# Halogen-Substitution of Benzotriazolium Cations Unlocks Enhanced Lewis Acidity

**DOI:** 10.1021/acsomega.6c09524

**Published:** 2026-10-27

**Authors:** Reece Lister-Roberts, John S. Wenger, Meera Mehta

**Affiliations:** 6396 University of Oxford, Department of Chemistry, 12 Mansfield Road, Oxford OX1 3QR, U.K.

## Abstract

Nitrenium cations have seen a surge of interest for catalytic applications as they are inexpensive, robust, and have high tunability. Thus far, this tunability has focused on modification of the wingtip, N-bound substituents or the employment of alternative aryl scaffolds. Here, we report the systematic modification of the benzotriazolium cation by installing a variety of halogen substitution patterns to the aryl backbone. Computational investigations suggested that these modifications would increase the Lewis acidity of the nitrenium center, which was supported experimentally by the Gutmann–Beckett test. The series of novel cations were employed as Lewis acid catalysts in a model reaction, and the most Lewis acidic derivative exhibited significantly enhanced catalytic performance relative to the parent, unsubstituted benzotriazolium cation. We further examined the relative stabilities of the neutral radical forms of these derivatives through a combined experimental and computational approach.

## Graphical abstract

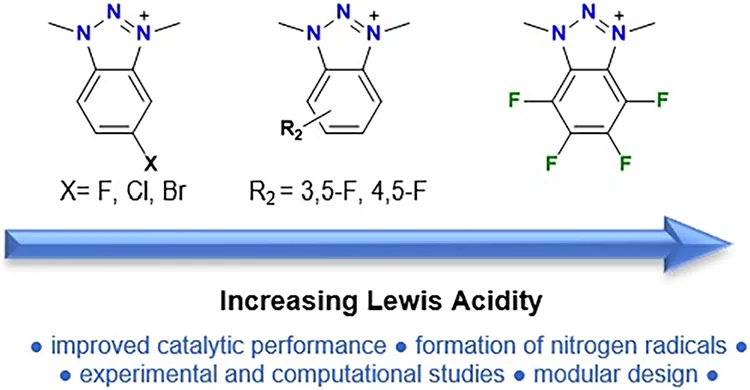

## Introduction

Transformations mediated by Earth-abundant p-block elements have seen a surge in interest amid growing environmental and fiscal concerns over the use of less abundant transition metals.^1^ Advances in the design of main group compounds which feature vacant, energetically accessible frontier orbitals have led to tremendous applications in small-molecule activation and Lewis acid catalysis.^2,3^ This research has largely focused on compounds featuring Group 13, Group 14, and heavier Group 15 elements as the locus of Lewis acidity, whereas the use of nitrogen catalysts in this area remains underdeveloped.^4,5^

*N*-heterocyclic nitrenium (NHN) cations represent the first examples of Lewis acid catalysts based on nitrogen. NHN cations are of fundamental interest due to their ease of synthesis, modularity, bench stability, and robustness.^6^ NHN cations were first shown to be capable π-acceptor ligands in transition metal complexes, with this property explored and developed in subsequent years.^7–11^ This led to a study on their coordination toward Lewis bases, and eventually their deployment as Lewis acid catalysts for five organic transformations by Mehta and Goicoechea, namely Friedel–Crafts dimerization of 1,1-diphenylethylene, hydrodefluorination of fluoroadamantane, deoxygenation of ketones, transfer hydrogenation of alkenes, and dehydrocoupling of phenol and triethylsilane.^12,13^ Since then, several generations of increasingly potent NHN cations in Lewis acid catalysis have been developed, capable of mediating the hydrosilylation of imines, reduction of acetophenone, and deoxygenation reduction of unsaturated ketones.^14–18^ Applications outside of Lewis acid catalysis have also recently been developed including uses in electrophilic amination, triazenolysis of alkenes, as a multimodal source of hydrogen leading to isolation of the triazinyl radical, and as electrolytes.^19–22^ To date, all of the modifications of these scaffolds have focused on the conjugation of the backbone or alteration of the wingtip, N-bound substituents (Figure 1a).^6,13,16^

**Figure 1:**
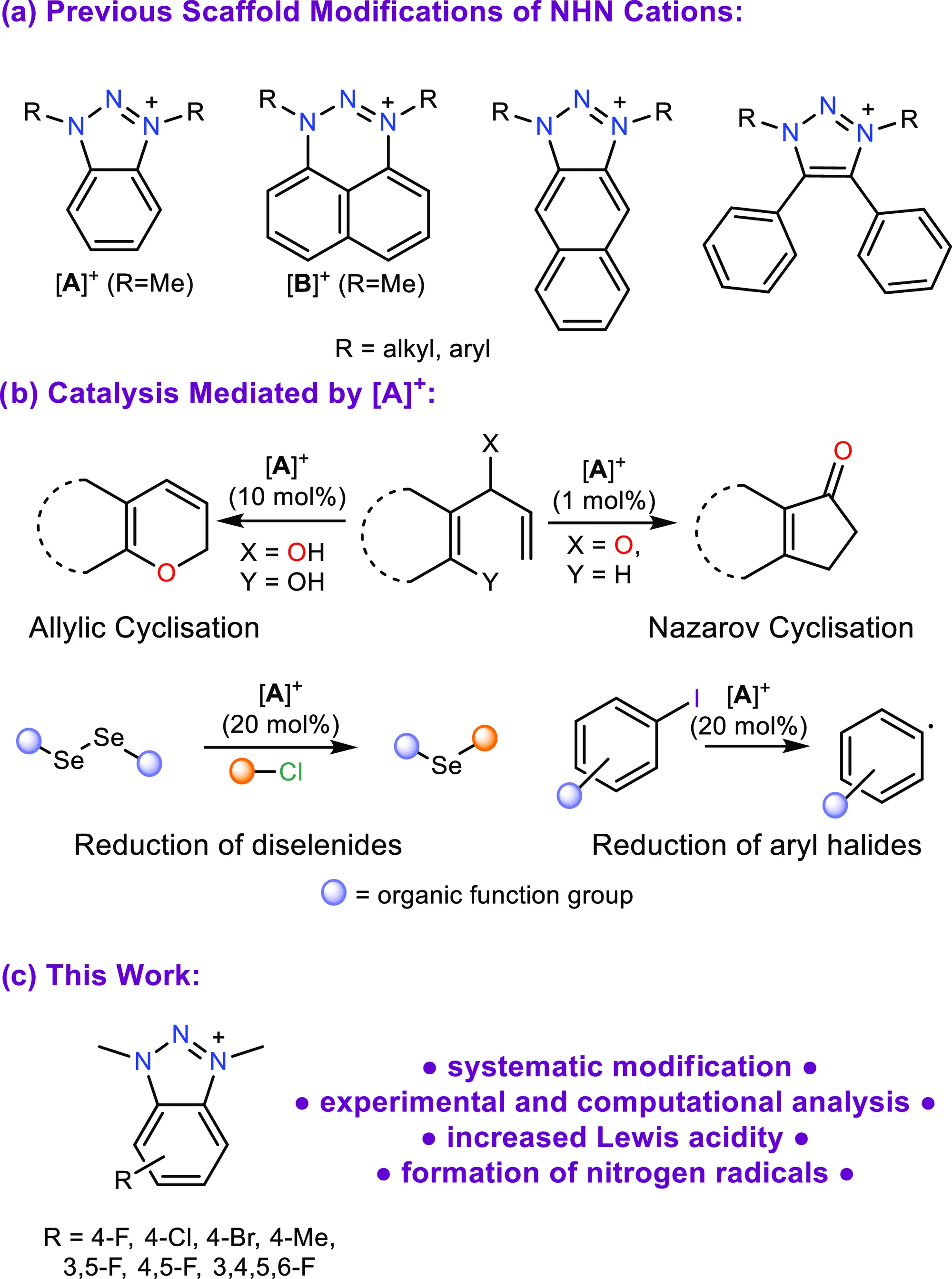
(a) Previous scaffold modifications to NHN cations. (b) Reactions catalyzed by NHN cation [**A**]^+^. (c) This work.

Recently, NHN cations based on benzotriazolium salts ([**A**])^+^, where R = Me; Figure 1(a) have been developed due to their ability to form charge-transfer complexes and enable distinct reactivity.^23^ In 2022, they were first deployed for the mediation of allylic and Nazarov cyclizations before they were reported as photocatalysts for the single electron reduction of aryl halides (Figure 1b).^24,25^ Later studies have shown that these visible light-mediated charge transfer complexes can be formed with both the anion or with an external base.^26^ Photocatalytic protocols with [**A**]^+^ are now better developed, with a range of organic transformations achieved including reduction of acyl fluorides and Se–Se bonds.^27–31^

Herein, we report a series of functionalized benzotriazolium salts and systematically investigate their properties. Unlike the more highly conjugated NHN cations, benzotriazolium cations have thus far remained unmodified within the literature; only the effect of varied counter-anions has been explored. We find that an increase in electron-withdrawing groups in the backbone leads to an increase in Lewis acidity, both experimentally and computationally, which materializes in increased catalytic competency of the most substituted derivative relative to the parent NHN cation. This increase in electron-withdrawing groups in the backbone is also shown to make the reduction potential of the NHN cations less negative, and make the corresponding radicals more reactive, which could be utilized in future catalytic applications.

## Results and Discussion

We targeted the synthesis of NHN cations [**1**]^+^–[**7**]^+^ (Figure 2a) to elucidate the effect of substitution with varied electron withdrawing groups on the active nitrenium center. Further, the aryl backbone was substituted with differing numbers of fluorine atoms, with different positional substitutions, to further study the effects of electron-withdrawing substituents. To this end, we synthesized compounds [**1**]­[BArF_20_], [**2**]­[BArF_20_], [**3**]­[BArF_20_], [**4**]­[BArF_20_], [**5**]­[BArF_20_]·(DCM)_0.5_ (DCM = dichloromethane), [**6**]­[BArF_20_], and [**7**]­[BArF_20_] ([BArF_20_] = tetrakis­(pentafluorophenyl)­borate), following an adapted literature protocol (Figure 2).^24^ In the first step of the synthesis, a substituted 1,2-diaminobenzene is treated with *tert*-butyl nitrite, to form the substituted benzotriazole. This intermediate is then methylated with methyl trifluoromethanesulfonate to form the backbone-functionalized 1,3-dimethyl-1*H*-benzo­[*d*]­[1,2,3]­triazol-3-ium trifluoromethanesulfonate salt. Finally, a salt metathesis reaction with [K]­[BArF_20_] allowed for the formation of the final products. We made many attempts to isolate the triflate salts for comparison; however, we were unsuccessful as the workup procedures employed for the parent benzotriazolium cation do not afford pure material for these derivatives, but rather intensely colored, crude oils. Methylation reactions conducted in this way are known to produce side products containing a methyl group at the central nitrogen and are therefore the likely main cause of the poor yields.^32^ Furthermore, the isolation of the novel benzotriazolium [BArF_20_]^−^ salts reported herein as analytically pure materials required multiple careful recrystallization and wash steps which resulted in quite low yields in each case (<5%). Each derivative was characterized fully by nuclear magnetic resonance (NMR) spectroscopy, single crystal X-ray diffraction (SC-XRD) studies, mass spectrometry, infrared spectroscopy, and elemental analysis. Unfortunately, elemental analyses of [**7**]­[BArF_20_] were unsuccessful, and thus, we did not continue investigating this compound.

**Figure 2:**
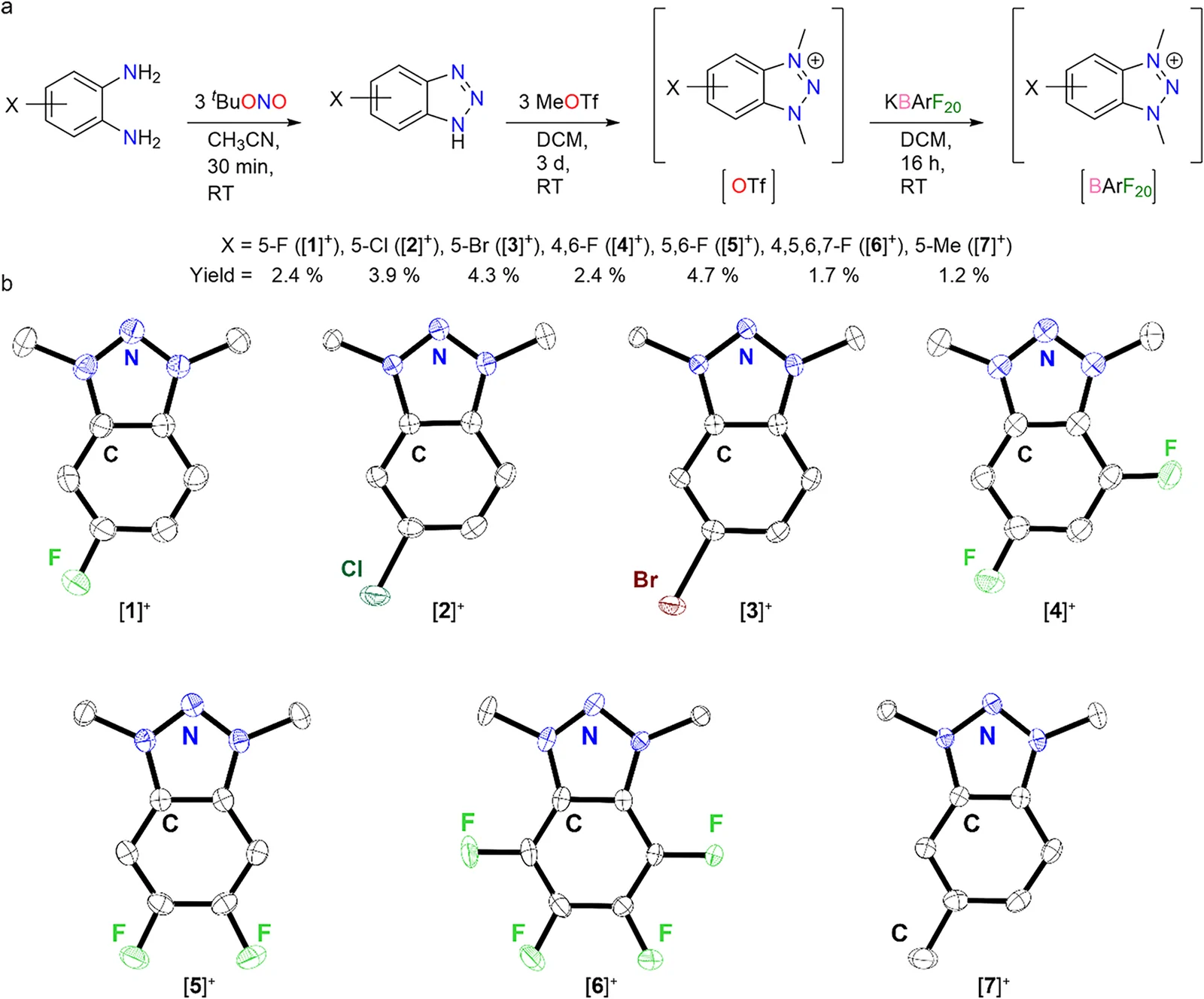
(a) Reaction scheme for the synthesis of [**1**]­[BArF_20_]–[**7**]­[BArF_20_]. (b) Thermal ellipsoid plots (50% probability) of [**1**]­[BArF_20_]–[**7**]­[BArF_20_]. H atoms, BArF_20_^–^ anions, and solvent molecules are omitted for clarity. Color code: C black, N blue, F light green, Cl dark green, Br maroon. Thermal ellipsoid plots containing the entire asymmetric unit are provided in the SI.

Crystals of each derivative suitable for X-ray diffraction could be readily obtained by slow evaporation of DCM solutions at room temperature. SC-XRD analysis of the different derivatives reveals little or no discernible structural deviations of the triazolium unit from one another. Interestingly, compounds [**2**]­[BArF_20_], [**3**]­[BArF_20_], [**6**]­[BArF_20_], and [**7**]­[BArF_20_] were characterized as crystallographic isomorphs, each crystallizing in the *Fdd*2 space group with a single molecule in the asymmetric unit. However, compound [**1**]­[BArF_20_] crystallized in the *P*1 space group with four molecules in the unit cell, and compound [**4**]­[BArF_20_] crystallized in the *Cc* space group, with the cation [**4**]^+^ being disordered about two orientations. In this regard, one or more of the benzotriazolium cations in the asymmetric unit of several of the derivatives were disordered about two positions related by “wagging” or “flipping” of the cation within its respective crystallographic pocket. Compound [**5**]­[BArF_20_]·(DCM)_0.5_ cocrystallized with half an equivalent of DCM, and the presence of the 0.5 equiv of DCM present in the solid-state structure was also identified in bulk material by NMR spectroscopy and elemental analysis.

Density functional theory (DFT) calculations were carried out on [**1**]^+^–[**6**]^+^ and [**A**]^+^ to probe the changes in electronic structure across the series. In each case, a singlet electronic configuration was dramatically more stable than a triplet state (singlet–triplet gap of >60 kcal mol^–1^), consistent with other previously reported NHNs in the literature (Supporting Table S13).^33,34^ The highest occupied molecular orbital (HOMO) and lowest unoccupied molecular orbital (LUMO) both decrease in energy from [**3**]^**+**^ > [**2**]^**+**^ > [**1**]^+^, consistent with the increasing electronegativity of the halogen substituent (Figure 3a). Increasing the number of fluorine substituents in the backbone further decreases the HOMO and LUMO energy relative to those of [**1**]^+^–[**3**]^+^. Among the new compounds, [**5**]^+^ features the lowest HOMO energy while [**6**]^+^ features the lowest LUMO energy. The relative energetic accessibility of the LUMO of [**6**]^+^ serves as an indication that the perfluorinated derivative may be the most competent Lewis acid. [**A**]­[BArF_20_] was found to have a higher LUMO energy than [**1**]­[BArF_20_]–[**6**]­[BArF_20_]. We visualized the HOMO and LUMO of the cation [**1**]^+^ as a representative example (Figure 3b,3c). Both the HOMO and LUMO of [**1**]^+^ are highly delocalized, consisting of π-bonding and π*-antibonding contributions throughout the fused ring system, respectively. Notably, the LUMO features a prominent contribution from the vacant N-centered p-orbital, which serves as the locus of Lewis acidity for these species.^13^ Frontier molecular orbitals of [**1**]^+^–[**6**]^+^ and [**A**]^+^ were also visualized (Supporting Figures S69–S82). Inspection of the HOMO of [**5**]^+^ and [**6**]^+^ suggests that the unexpected lower HOMO energy of [**5**]^+^ than [**6**]^+^ is a result of a crossover in orbital ordering rather than an anomalous stabilization effect. Natural population analysis of the central NHN cation's nitrogen atom (N1) finds only subtle deviations across the series, but notably the highest positive charge was found in the case of [**6**]^+^ (0.059 e^–^), followed by [**4**]^+^ (0.053 e^–^). Unexpectedly, [**5**]^+^ (0.046 e^–^) features a slightly lower Natural Charge on N1 than that of [**1**]^+^, [**2**]^+^, or [**3**]^**+**^ (0.047 e^–^).

**Figure 3:**
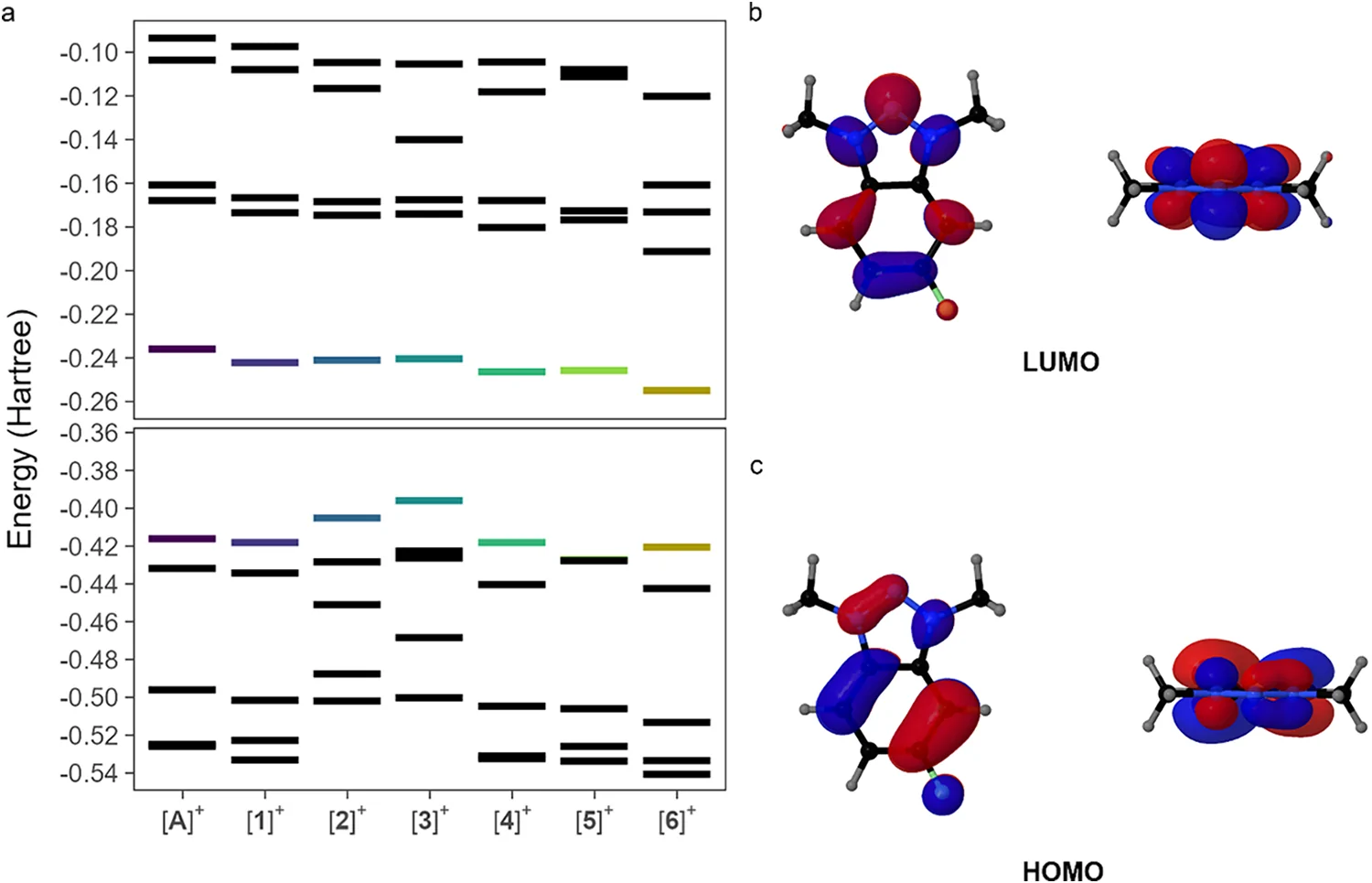
(a) Calculated orbital energies (B3LYP/def2-TZVP) for cations [**1**]^+^–[**6**]^+^ with frontier molecular orbitals shown in color. The upper panel contains the values for the LUMO, LUMO+1, LUMO+2, LUMO+3, and LUMO+4. The lower panel contains values for the HOMO, HOMO–1, HOMO–2, HOMO–3, and HOMO–4. Values are provided in Supporting Table S7. (b) LUMO and (c) HOMO of [**1**]^+^ (isosurface value = 0.04). Color code: C black, N blue, H gray, F green.

We then calculated the fluoride ion affinity (FIA; using the naked cations), hydride ion affinity (HIA; using the naked cations), and global electrophilicity index (GEI; using the [BArF_20_]^−^ salts; Table 1) to more directly probe the Lewis acidity of the series of derivatives (computed at the B3LYP/def2-TZVP level of theory).^35,36^ These methods were in agreement that an increase of electron-withdrawing groups in the backbone increases the Lewis acidity at the nitrenium center, with [**6**]^+^/[**6**]­[BArF_20_] predicted to be the most Lewis acidic (FIA = 469 kJ mol^–1^; GEI = 7.43 eV), and [**A**]^+^/[**A**]­[BArF_20_] predicted to be the least Lewis acidic of the series (FIA = 430 kJ mol^–1^; GEI = 5.25 eV). GEI studies suggest that [**2**]­[BArF_20_] (GEI = 5.70 eV) and [**3**]­[BArF_20_] (GEI = 5.71 eV) have roughly the same electrophilicity with [**1**]­[BArF_20_] slightly higher (GEI = 5.89 eV). However, FIA calculations indicate that all three of the monosubstituted derivatives studied would have the same Lewis acidity (FIA = 437 kJ mol^–1^). HIA values across the series are overall greater in magnitude, but follow a similar trend to the FIA values, with [**A**]^+^ exhibiting the lowest HIA at 645 kJ/mol and [**6**]^+^ exhibiting the highest HIA at 722 kJ/mol.

**Table 1:** Lewis Acidity Measures of Cations [**1**]­[BArF_20_]–[**6**]­[BArF_20_] and [**A**]­[BArF_20_]

Compound	FIA (kJ mol^–1^)^a^	HIA (kJ mol^–1^)	GEI (eV)	Gutmann–Beckett AN
[**1**][BArF_20_]	437	672	5.89	21.86
[**2**][BArF_20_]	437	674	5.70	22.87
[**3**][BArF_20_]	437	674	5.71	23.51
[**4**][BArF_20_]	449	692	6.38	23.34
[**5**][BArF_20_]	446	684	6.47	25.53
[**6**][BArF_20_]	469	722	7.43	21.50^b^
[**A**][BArF_20_]	430	645	5.25	23.67

a DFT was conducted on the naked cation.

b Compound exhibited poor solubility in DCM.

We calculated the interaction energies between [**1**]^+^–[**6**]^+^ and [**A**]^+^ with neutral donors, PMe_3_ and 1,3-dimethylimidazol-2-ylidene (IMe) in the gas phase (Supporting Information Section 3.4), as NHNs have previously been reported to form stable Lewis adducts in the presence of phosphines.^13^ In each case, binding of neutral donors is the most favorable in the case of [**6**]^+^ and the least favorable in the case of [**A**]^+^, suggesting that the addition of fluorine groups to the aryl backbone should enhance the Lewis acidity of the nitrenium cation. Coordination of IMe to [**4**]^+^–[**6**]^+^ is only slightly favorable, while coordination IMe to [**1**]^+^–[**3**]^+^ and [**A**]^+^ is slightly unfavorable. In contrast, PMe_3_ coordination to each NHN studied is predicted to be substantially exothermic. Unfortunately, attempts to isolate PMe_3_ adducts of these Lewis acids were unsuccessful, resulting in no reaction in the cases of [**1**]^+^–[**5**]^+^ and [**A**]^+^ and decomposition in the case of [**6**]^+^.

Finally, to interrogate the relative oxophilicity and azaphilicity of the cations presented herein, we performed an energy decomposition analysis of adducts of [**1**]^+^–[**6**]^+^ and [**A**]^+^ with OH_2_ and NH_3_ (Supporting Table S12).^37^ Δ*E*_int_ values are larger in magnitude for the OH_2_ adducts than the NH_3_ adducts, with [**6**]^+^ exhibiting the largest interaction energies among the cations. Interestingly, |Δ*E*_orb_| is slightly larger in the respective NH_3_ adducts, while |Δ*E*_elstat_| is larger for the OH_2_ adducts. Notably, Δ*E*_Pauli_ is also larger in the NH_3_ adducts. These results overall suggest that the nitrenium cations presented herein are slightly more oxophilic, although steric factors will invariably play a central role in any nitrenium-mediated process.

The Gutmann–Beckett test was undertaken to calculate the acceptor number (AN) of the series and experimentally validate the calculated trends in Lewis acidity (Table 1). Here we see the same general trend, with increasing the number of halogens in the backbone increasing Lewis acidity of the compound. Unlike the DFT-based methods, the positional effects in the bis-fluorinated derivatives are apparent with [**5**]­[BArF_20_] having an AN that is approximately 10% higher than [**4**]­[BArF_20_]. Unfortunately, [**6**]­[BArF_20_] shows limited solubility in dichloromethane leading to a lower than expected AN, with precipitate visible in the NMR tube.^38,39^ Despite having a lower predicted Lewis acidity than the rest of the series by computational analysis, [**A**]­[BArF_20_] had an AN of 23.67, better than that of [**1**]­[BArF_20_] and [**2**]­[BArF_20_] and close to that of [**3**]­[BArF_20_] and [**4**]­[BArF_20_].

With the literature precedence for using NHN cations as catalysts in single-electron processes, cyclic voltammetry (CV) studies were also undertaken to probe the effect of derivatization on their redox properties (Figure 4). First, we can see the reduction potential of the cation becomes less negative as additional fluorine atoms are introduced into the backbone, with [**1**]­[BArF_20_] having the most negative reduction potential (*E*_red_ = −1.29 V) and [**6**]­[BArF_20_] having the least negative potential (*E*_red_ = −1.03 V). Interestingly, [**3**]­[BArF_20_] showed a second reduction wave immediately after the first, leading to an irreversible reduction; attempts were made to independently study these reductions by CV but were unsuccessful due to their lack of separation. Both [**4**]­[BArF_20_] and [**6**]­[BArF_20_] only show quasi-reversible reductions, which become less reversible at slower scan rates. This suggests that the radical may be formed but decomposes quickly, and that substitution of the closest aromatic position to the nitrogen atoms may be responsible for the lack of stability of the corresponding radical (see Supporting Figures S40 and S60 for scan rate dependence). Compounds [**1**]­[BArF_20_], [**2**]­[BArF_20_], and [**5**]­[BArF_20_] show completely reversible reduction waves suggesting that the corresponding radicals may be stable. When comparing the parent species, [**A**]­[BArF_20_], to the series, we see that it has the most negative reduction potential and displays a reversible reduction, in line with the expected trend.

**Figure 4:**
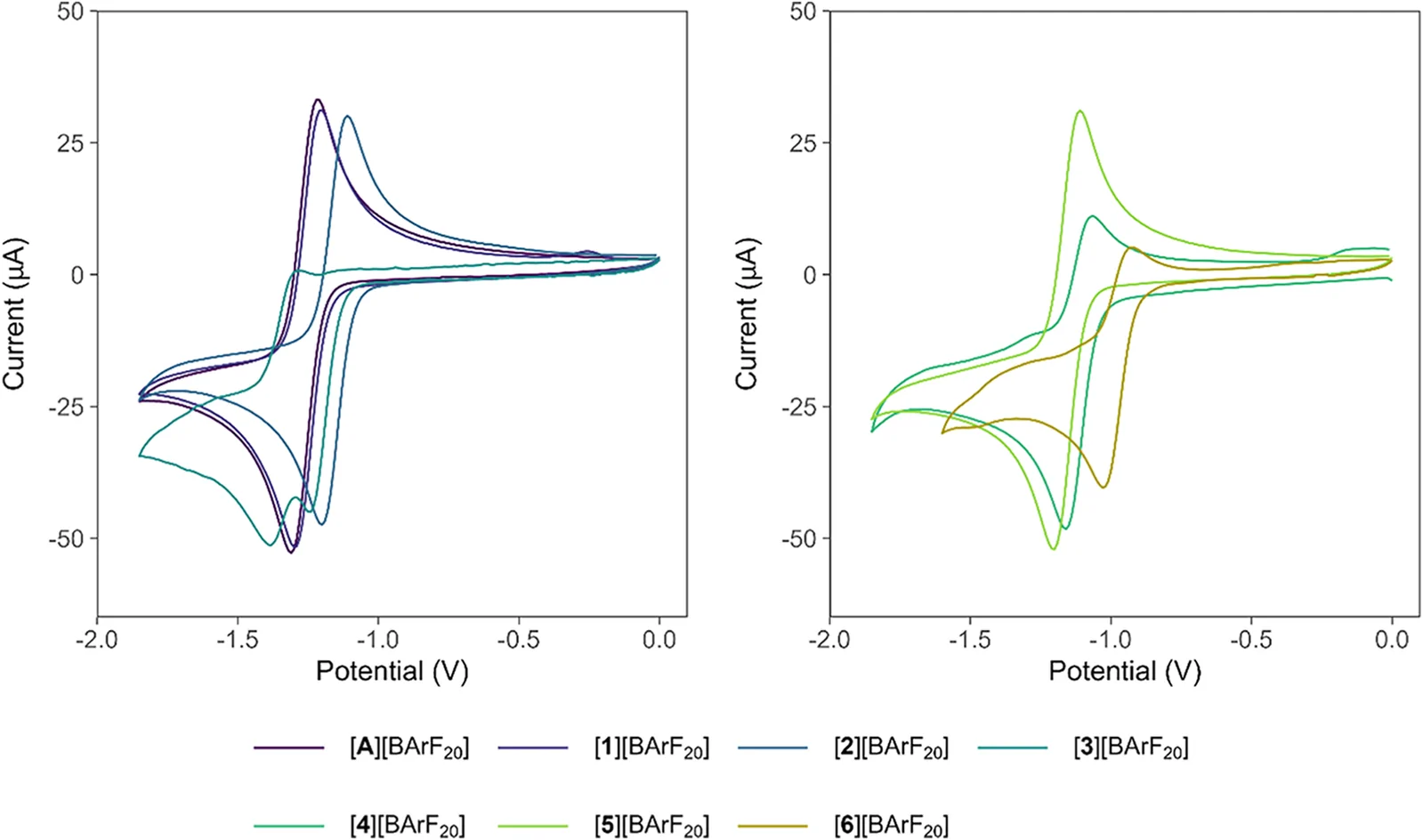
Cyclic voltammetry data for compounds [**A**]­[BArF_20_], [**1**]­[BArF_20_]–[**6**]­[BArF_20_]. Experimental details: analyte; 2 mM. Electrolyte; 100 equiv. [Bu_4_N]­[PF_6_]. Working electrode; glassy carbon. Counter electrode; platinum. Reference electrode; leak-proof Ag/AgCl. Scan rate; 0.1 V/s.

The suggested stability of **1** ^•^, **2** ^•^, and **5** ^•^ from the CV data led us to further study their solution state stability and properties. Cations [**1**]­[BArF_20_]–[**6**]­[BArF_20_] were each reduced with one equivalent of KC_8_ and the corresponding neutral radical was studied by electron paramagnetic resonance (EPR) spectroscopy. In line with the CV data, [**1**]­[BArF_20_], [**2**]­[BArF_20_], and [**5**]­[BArF_20_] turned blue upon reduction and were able to be studied by EPR spectroscopy yielding highly delocalized radicals (Figure 5b); these EPR data could not be simulated due to the high complexity of the spectra. Efforts to isolate radicals **1** ^•^, **2** ^•^, and **5** ^•^ have thus far been unsuccessful. The remaining compounds turned purple when exposed to KC_8_ or cobaltocene and did not show any resonance in EPR spectroscopy. For [**3**]­[BArF_20_], we believe that this is due to the additional reduction which occurs immediately after the first wave observed by CV, whereas for [**4**]­[BArF_20_] and [**6**]­[BArF_20_] we suggest that the radical does in fact form but decomposes quickly in line with the irreversible waves observed in the CV data.

**Figure 5:**
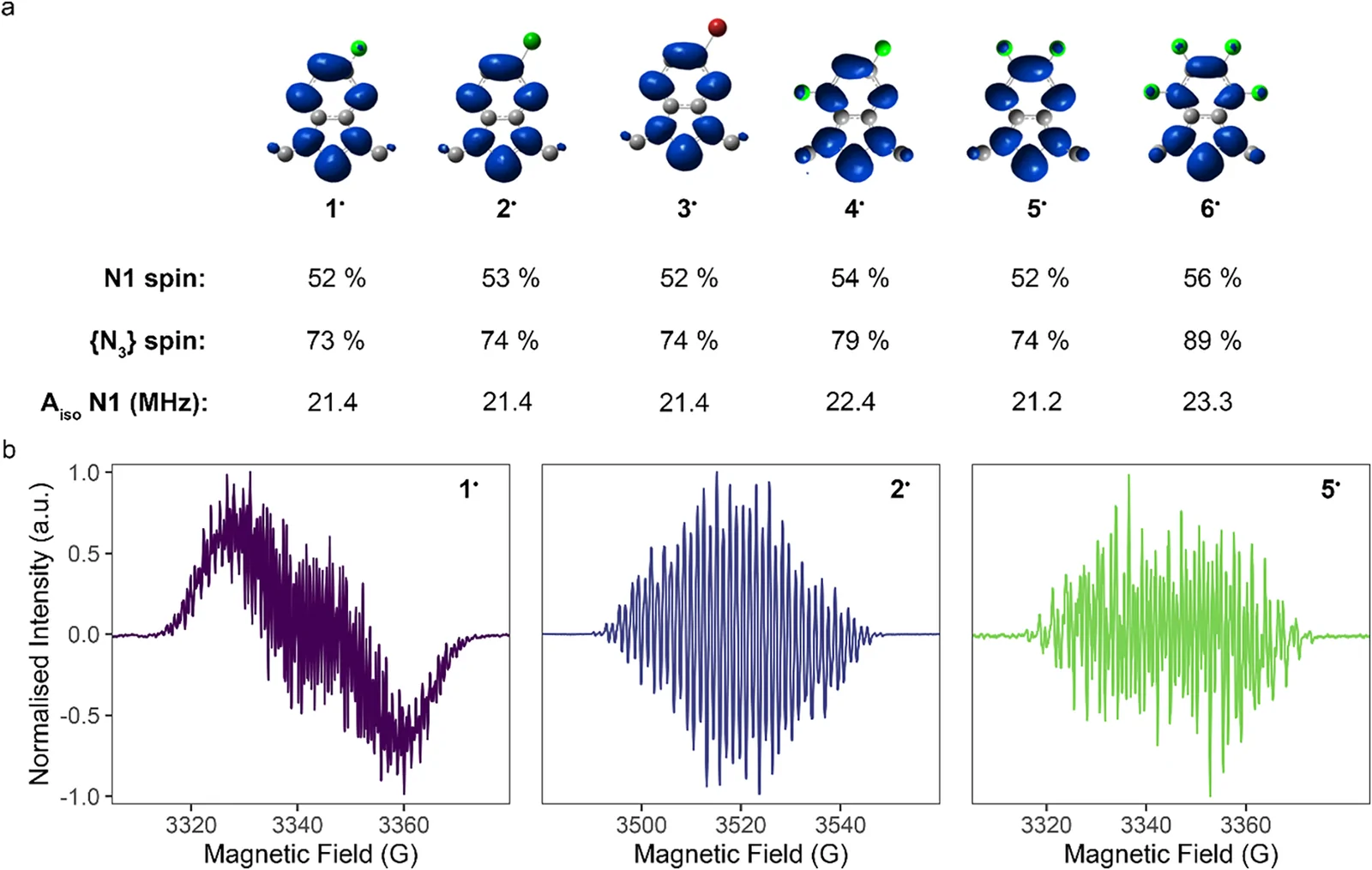
(a) Spin density plots for **1** ^•^–**6** ^•^ with spin density at the central nitrogen atom (N_1_) and the {N_3_} motif for each shown below. Calculated *A*_iso_ values for the N_1_ shown as another measure of N_1_ spin. (b) EPR spectra for **1**_,_^•^ **2** ^•^, and **5** ^•^ at X-band. Specific experimental parameters are given in the Supporting Information.

With the difficulty in modeling such complex EPR spectra, DFT calculations were undertaken to predict the isotropic hyperfine coupling constant (*A*_iso_; Figure 5a). The calculation confirmed that the radical is highly delocalized in all cases, but that the coupling to the central nitrogen atom (N_1_) is higher in the more substituted, less stable radicals **4** ^•^ and **6** ^•^. DFT studies were also conducted on **1** ^•^–**6** ^•^ to probe the spin density of the radicals. Compounds **1** ^•^, **2** ^•^, **3** ^•^, and **5** ^•^ had the least spin density on the {N_3_} unit while **4** ^•^ and **6** ^•^ were shown to have an additional 6% and 16% spin density, respectively, on the {N_3_} unit compared to **1** ^•^. The same trend can be observed in the N_1_ spin, albeit less pronounced. This is consistent with the radicals which were stable enough to be observed by EPR spectroscopy having the lowest spin density at the {N_3_} unit (with the exception of **3** ^•^ previously explained). The derivatives with the most spin density at the central nitrogen would be expected to be the most reactive, hence explaining why **4** ^•^ and **6** ^•^ cannot be observed by EPR spectroscopy.

Finally, a literature model reaction (allylic cyclization) was selected to probe the performance of the novel derivatives as Lewis acid catalysts compared to the parent compound, [**A**]­[BArF_20_] (Table 2).^24^ Compounds [**1**]­[BArF_20_]–[**6**]­[BArF_20_] were employed at 10 mol % loading under the same conditions as the model reaction to allow for direct comparison to the parent NHN salt, which only achieved 4% yield in the reaction as the [BArF_20_]^−^ salt. A transformation where [**A**]­[BArF_20_] performs poorly was purposefully selected to highlight even subtle changes in catalytic performance between the derivatives. Only one of the new derivatives, [**6**]­[BArF_20_], outperformed the parent NHN with an NMR yield of 31%. Consistent with its high FIA and GEI values, it is not surprising that [**6**]­[BArF_20_] had the best performance and demonstrates that the substitution in the backbone is actively increasing the potency of the catalyst in Lewis acid catalysis. Similar to [**A**]­[BArF_20_], compounds [**1**]­[BArF_20_]–[**5**]­[BArF_20_] should not be considered catalysts for this reaction as their turnover number is less than 1. Although it is worth noting that [**A**]^+^ with [BF_4_]^−^ anion is indeed catalytic in this transformation.^24^ Further, it should be noted that the increased yield obtained with [**6**]­[BArF_20_] is still lower than that of NHN cations based on different backbones and with different wingtip groups. This performance increase compared to [**A**]­[BArF_20_] highlights that halogenation of the backbone can have a large effect on catalyst performance. This new approach to NHN modification adds another layer of tunability to these catalysts which can be employed alongside the backbone and wingtip modification methods reported in the literature.

**Table 2:**
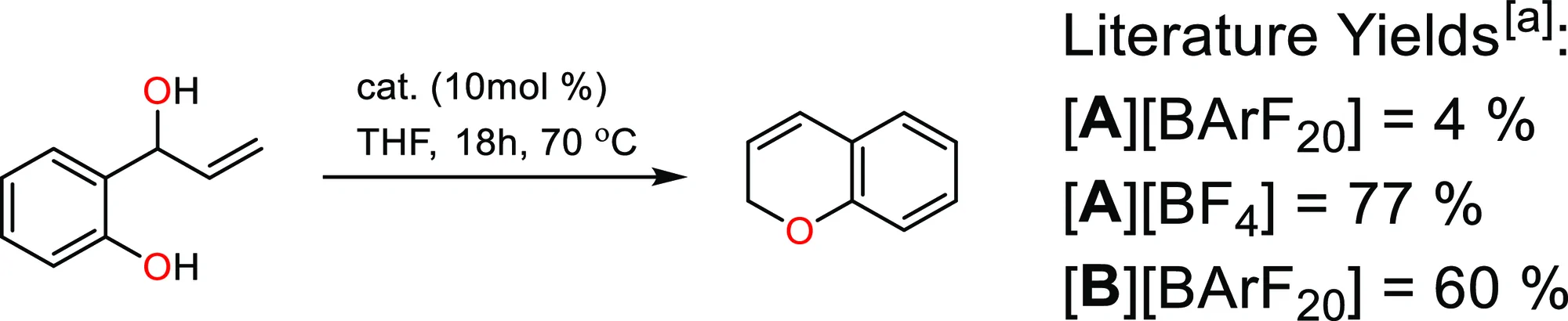
Performance of [**A**]­[BArF_20_], [**B**]­[BArF_20_], and [**1**]­[BArF_20_]–[**6**]­[BArF_20_] as catalysts for an Allylic Cyclisation Reaction^a^

Catalyst	^1^H NMR Yield (%, vs CH_2_Br_2_ Internal Standard)				
	Run 1	Run 2	Run 3	Average	esd
[**1**][BArF_20_]	3	3.4	3.5	3.3	0.3
[**2**][BArF_20_]	3.7	5.4	5.2	4.8	0.9
[**3**][BArF_20_]	2.4	5.1	3.6	3.7	1.4
[**4**][BArF_20_]	3.5	4.1	3.5	3.7	0.3
[**5**][BArF_20_]·(DCM)_0.5_	3.3	5.0	3.3	3.9	1
[**6**][BArF_20_]	34.2	32.1	25.1	30.5	4.8

a Previously reported value.^24^

## Conclusion

In summary, we have developed six new NHN cations with varying halogen substituents in the backbone of the molecule. Computational Lewis acidity tests indicate that adding more electron-withdrawing groups to the backbone increases the Lewis acidity and electrophilicity at the central nitrogen atom. However, experimental studies indicate that only [**6**]­[BArF_20_] significantly outperforms the parent compound [**A**]­[BArF_20_] in catalysis, where it achieved 31% yield of *2H*-chromene from 2-(1-hydroxyallyl)­phenol compared to 4% yield to *2H*-chromene with [**A**]­[BArF_20_] under similar conditions. Increasing the number of electron-withdrawing groups in the backbone was also shown to make these NHN cations easier to reduce, but in turn makes the corresponding radical less stable. Efforts are currently underway to expand the role of halogen-functionalized nitrenium cations in catalysis and to install more strongly mesomerically electron-withdrawing groups, such as ester, cyano, and nitro.

## Methods

All manipulations were carried out under a nitrogen atmosphere using standard Schlenk-line and glovebox methodology. All glassware was flame-dried before use. Innovative Technologies anhydrous engineering solvent purification system was used to obtain dry tetrahydrofuran, diethyl ether, toluene, acetonitrile (MeCN), DCM and pentane. Solvents obtained in this way were subsequently degassed and stored over 3 Å molecular sieves. All starting materials (unless otherwise stated below) were purchased from commercial sources and used without further purification.

KC_8_ and [K]­[BArF_20_] (BArF_20_ = B­(C_6_F_5_)_4_) were made according to literature preparations.^40,41^

### Procedure for Synthesis of 1,3-Dimethyl Benzotriazolium Tetrakis­(pentafluorophenyl)­borate Derivatives

The respective 1,2-diaminobenzene derivative (1.0 g, 1 equiv) was dissolved in MeCN (50 mL) before addition of *tert*-butyl nitrite (3 equiv). The solution was stirred for 30 min before the solvent was removed under dynamic vacuum yielding the 1,2,3-triazole which was weighed and then dried overnight under high vacuum in a Schlenk flask. The next step of the procedure was completed under an inert atmosphere with dry solvents. The solid was dissolved/suspended in DCM and methyl triflate (3 equiv) was added dropwise. The reaction was stirred for 3 days. Bench stored diethyl ether was added (50 mL) and stirred for 1 h causing a precipitate to form. The supernatant was poured off and the remaining solid/oil washed twice more with diethyl ether. The crude material was pushed through a short silica plug in pure acetonitrile before removing the solvent and drying under dynamic vacuum yielding the crude nitrenium triflate salt. In the glovebox, the crude nitrenium triflate salt was stirred with K­[BArF_20_] (1.1 equiv) in DCM for 16 h. The product was filtered with the insoluble solid extracted 3 tismes with DCM before the layers were combined and solvent removed in vacuo. The solid was then washed with chloroform (3 × 10 mL) before drying and recrystallization from hot DCM. The isolated solids were then washed with chloroform and recrystallized from hot DCM a second time. The supernatant was then decanted and the solids were washed with chloroform before being dried under vacuum.

#### General Procedure for Reduction of NHN Cations with KC_8_

All EPR spectroscopy samples were prepared according to the following procedure:

In a vial in the glovebox, [**1**]­[BArF_20_]–[**6**]­[BArF_20_] (10 mg) and KC_8_ (1 equiv) were suspended in toluene (2 mL) and shaken for 1 min. The solution was filtered and an aliquot was taken into a J. Young EPR tube for analysis by EPR spectroscopy.

## Supplementary Material



## Notes

Experimental procedures; ^1^H NMR spectrum (CD_3_CN, 500 MHz) of [1]­[BArF_20_] at room temperature; ^13^C­{^1^H} NMR spectrum (CD_3_CN, 126 MHz) of [1]­[BArF_20_] at room temperature; ^1^B­{^1^H} NMR spectrum (CD_3_CN, 160 MHz) of [1]­[BArF_20_] at room temperature (PDF)
