# Supplementary material for: Halogen-Substitution of Benzotriazolium Cations Unlocks Enhanced Lewis Acidity
Source: ACS Omega. 2026 Oct 27;11(44):69302–10. doi: 10.1021/acsomega.6c09524 (PMC13756702; doi:10.1021/acsomega.6c09524)
Supplement: Supplementary file 1 [file ao6c09524_si_008.pdf]

Supporting Information

Halogen-substitution of Benzotriazolium Cations Unlocks Enhanced Lewis Acidity

Reece Lister-Roberts,[a]† *John S. Wenger*,[a]† *and Meera Mehta**[a]

[a] Department of Chemistry, University of Oxford, 12 Mansfield Road, Oxford, OX1 3QR, U.K.

meera.mehta@chem.ox.ac.uk

† These authors contributed equally.

Contents

1. Methods and Materials ... 4
Experimental Considerations ... 4
Analytical Considerations ... 4
General computational considerations ... 5
2. Experimental and Characterisation Data ... 5
2.1 General Synthetic Procedures ... 5
2.1.1 Procedure for Synthesis of 1,3-dimethyl benzotriazolium tetrakis(pentafluorophenyl)borate derivatives:[9] ... 5
2.1.2 General Procedure for Reduction of NHN Cations with KC_8 ... 6
2.2 5-Fluoro-1,3-dimethyl-1H-benzo[d][1,2,3]triazol-3-ium tetrakis(pentafluorophenyl)borate - [1][$BArF_{20}$] ... 6
2.2.1 Synthesis ... 6
2.2.2 Cyclic Voltammetry ... 13
2.2.3 Electron Paramagnetic Resonance and Spin Density Data ... 14
2.3 5-Chloro-1,3-dimethyl-1H-benzo[d][1,2,3]triazol-3-ium tetrakis(pentafluorophenyl)borate - [2][$BArF_{20}$] ... 15
2.3.1 Synthesis ... 15
2.3.2 Cyclic Voltammetry ... 21
2.3.3 Electron Paramagnetic Resonance and Spin Density Data ... 22
2.4 5-Bromo-1,3-dimethyl-1H-benzo[d][1,2,3]triazol-3-ium tetrakis(pentafluorophenyl)borate - [3][$BArF_{20}$] ... 24
2.4.1 Synthesis ... 24
2.4.2 Cyclic Voltammetry ... 30
2.4.3 Spin Density Data ... 30
2.5 4,6-Difluoro-1,3-dimethyl-1H-benzo[d][1,2,3]triazol-3-ium tetrakis(pentafluorophenyl)borate - [4][$BArF_{20}$] ... 31
2.5.1 Synthesis ... 31
2.5.2 Cyclic Voltammetry ... 38
2.5.3 Spin Density ... 38
2.6 5,6-Difluoro-1,3-dimethyl-1H-benzo[d][1,2,3]triazol-3-ium tetrakis(pentafluorophenyl)borate - [5][$BArF_{20}$]•$(DCM)_{0.5}$ ... 40
2.6.1 Synthesis ... 40
2.6.2 Cyclic Voltammetry ... 47
2.6.3 Electron Paramagnetic Resonance and Spin Density Data ... 48
2.7 4,5,6,7-Tetrafluoro-1,3-dimethyl-1H-benzo[d][1,2,3]triazol-3-ium tetrakis(pentafluorophenyl)borate - [6][$BArF_{20}$] ... 49
2.7.1 Synthesis ... 49
2.7.2 Cyclic Voltammetry ... 55
2.7.3 Spin Density Data ... 56
2.8 5-Methyl-1,3-dimethyl-1H-benzo[d][1,2,3]triazol-3-ium tetrakis(pentafluorophenyl)borate - [7][$BArF_{20}$] ... 57

2.8.1 Synthesis 57
3. Lewis Acidity Studies 63
3.1 Canonical Molecular Orbital energies (Hartree) of $[1]^{+}$-$[6]^{+}$ (B3LYP/def2-TZVP). 63
3.2 Fluoride Ion Affinity 77
3.3 Hydride Ion Affinity 78
3.4 Binding Towards Neutral Donors 79
3.5 Singlet – Triplet Gaps 80
3.6 Global Electrophilicity Index 80
3.7 Gutmann-Beckett 81
3.8 Model catalytic reaction[9] 83
4. Crystallography Tables 87
5. Optimised coordinates 90
6. References 139

1. **Methods and Materials**

Experimental Considerations

All manipulations were carried out under a nitrogen atmosphere using standard Schlenk-line and glovebox methodology. All glassware was flame-dried before use. Innovative Technologies anhydrous engineering solvent purification system was used to obtain dry THF, Et_2O, toluene, acetonitrile (MeCN), DCM and pentane. Solvents obtained in this way were subsequently degassed and stored over 3 Å molecular sieves. All starting materials (unless otherwise stated below) were purchased from commercial sources and used without further purification.

KC_8 and $KBArF_{20}$ ($BArF_{20} = B(C_6F_5)_4$) were made according to literature preparations.[1,2]

Analytical Considerations

NMR spectroscopy. ^1H, $^{11}B\{^1H\}$, $^{13}C\{^1H\}$, ^{19}F, and $^{19}F\{^1H\}$ NMR spectra for characterisation were recorded on a Bruker AVIII 500 spectrometer (operating frequencies: 499.94 MHz, 160 MHz, 125.7 MHz, and 470.6 MHz respectively). ^1H and $^{31}P\{^1H\}$ NMR spectra collected for analysis of the catalytic runs and Gutmann-Beckett test were recorded on a Bruker AVIII 400 with an operating frequency of 399.78 MHz and 161.99 MHz respectively. ^1H chemical shifts were internally referenced to the residual solvent resonances. All NMR data were analysed using MestReNova V15.1.0.

Electron paramagnetic resonance. Electron paramagnetic resonance (EPR) spectra were recorded at X band (9.3–9.8 GHz) with a Bruker EMXmicro spectrometer at 298 K.

Cyclic voltammetry. Cyclic voltammetry (CV) was carried out in the glovebox under inert conditions with EMStat4s. Electrodes: Working – glassy carbon. Counter – platinum wire. Reference – Ag /AgCl (leak proof).

Infrared spectroscopy. ATR-IR spectra were recorded on microcrystalline powders using a Bruker Alpha II under an inert atmosphere.

Mass spectrometry. Mass spectrometry samples were analysed by the mass spectrometry service at the University of Oxford using an electrospray ionization (ESI) equipped Waters RDa bench-top time of flight mass spectrometer. Samples were prepared under a nitrogen atmosphere and directly injected into the ionization source of the mass spectrometer.

X-ray diffraction studies. X-ray diffraction data were collected for compounds [**1**][$BArF_{20}$]-[**5**][$BArF_{20}$] and [**7**][$BArF_{20}$] on a dual source Rigaku XtaLAB Synergy-DW VHF equipped with a PhotonJet-R dual wavelength rotating anode and HyPix-Arc 150° detector at 100 K using Cu Kα (1.54184 Å) radiation. X-ray diffraction data for [**6**][$BArF_{20}$] were collected using a dual source Rigaku FR-X rotating anode diffractometer with a Hypix-6000HE detector and an Oxford Cryosystems nitrogen flow gas system at 100 K using Cu Kα (1.54184 Å) radiation. X-ray data were collected using CrysAlisPro software.

Crystal structure determination and refinements: X-ray data were processed and reduced using CrysAlisPro. Absorption correction was performed using empirical methods (SCALE3 ABSPACK)

based upon symmetry-equivalent reflections combined with measurements at different azimuthal angles. The crystal structure was solved and refined against all F2 values using the SHELX and Olex2 suite of programmes. All atoms were refined anisotropically. Hydrogen atoms were placed in calculated positions and refined using idealised geometries and assigned fixed isotropic displacement parameters. Disordered atoms were treated with same-distance (SADI), similarity (SIMU), and rigid-bond (RIGU) restraints. In the case of [**1**][$BArF_{20}$], inspection of the Fourier difference map identified some residual density that likely corresponds to the presence of a fraction of an equivalent of DCM, which could not be reliably modelled. The solvent mask applied to [**1**][$BArF_{20}$] identified two small electron-containing voids in the unit cell with a total electron count of 6 electrons in a volume of 58.5 Å^3. Crystallographic data for [**1**][$BArF_{20}$], [**2**][$BArF_{20}$], [**3**][$BArF_{20}$], [**4**][$BArF_{20}$], [**5**][$BArF_{20}$]•$(DCM)_{0.5}$, [**6**][$BArF_{20}$], and [**7**][$BArF_{20}$] have been deposited via the joint CCDC/FIZ Karlsruhe deposition service under 2564493, 2564490, 2564489, 2564488, 2564492, 2564487, and 2564491, respectively.

General computational considerations

DFT geometry optimizations, frequency calculations, and single point energy calculations were carried out using the Gaussian 16 package, revision C.01.[3] All calculations were carried out using the B3LYP functional and the Ahlrichs def2 basis set of polarised triple-ζ quality (def2-TZVP).[4–6] Geometry optimisations were performed, with default settings, starting from crystallographic coordinates where possible. Analysis of the harmonic vibrational frequencies confirmed the optimised geometries as energetic minima. For each compound, the gas phase cation was used for all calculations except for the global electrophilicity index calculations which also included the $[BArF_{20}]^-$ counter anion. Canonical Molecular Orbitals were visualised in JMOL (version 16.3.49). Spin distribution plots were visualised in GaussView 16. Geometry optimizations and energy decomposition analysis of adducts of [**A**], [**1**]$^+$-[**6**]$^+$ with OH_2 and NH_3 were performed using *ORCA 6.1*.[7,8] Energy decomposition analysis was performed *via* a fragmentation scheme in which the first fragment was comprised of the nitrenium cation and the second fragment was comprised of the neutral donor OH_2 or NH_3.

2. Experimental and Characterisation Data

2.1 General Synthetic Procedures

2.1.1 Procedure for Synthesis of 1,3-dimethyl benzotriazolium tetrakis(pentafluorophenyl)borate derivatives:[9]

The respective 1,2-diaminobenzene derivative (1.0 g, 1 equiv.) was dissolved in MeCN (50 mL) before addition of tert-butyl nitrite (3 equiv.). The solution was stirred for 30 min before the solvent was removed under dynamic vacuum yielding the 1,2,3-triazole which was weighed and then dried overnight under high vacuum in a Schlenk flask. The next step of the procedure was completed under an inert atmosphere with dry solvents. The solid was dissolved / suspended in DCM and methyl triflate (3 equiv.) was added dropwise. The reaction was stirred for 3 days. Bench stored diethyl ether was added (50 mL) and stirred for one hour causing a precipitate to form. The supernatant was poured off and the remaining solid / oil washed twice more with diethyl ether. The crude material was pushed through a

short silica plug in pure acetonitrile before removing the solvent and drying under dynamic vacuum yielding the crude nitrenium triflate salt. In the glovebox, the crude nitrenium triflate salt was stirred with K[$BArF_{20}$] (1.1 equiv.) in DCM for 16 hours. The product was filtered with the insoluble solid extracted 3 times with DCM before the layers were combined and solvent removed in vacuo. The solid was then washed with chloroform (3 × 10 mL) before drying and recrystallisation from hot DCM. The isolated solids were then washed with chloroform and recrystallised from hot DCM a second time. The supernatant was then decanted and the solids were washed with chloroform before being dried under vacuum.

2.1.2 General Procedure for Reduction of NHN Cations with KC_8

All EPR spectroscopy samples were prepared according to the following procedure:

In a vial in the glovebox, [**1**][$BArF_{20}$]-[**6**][$BArF_{20}$] (10 mg) and KC_8 (1 equiv.) were suspended in toluene (2 mL) and shaken for 1 min. The solution was filtered and an aliquot was taken into a J Young EPR tube for analysis by EPR spectroscopy.

2.2 5-Fluoro-1,3-dimethyl-1H-benzo[d][1,2,3]triazol-3-ium tetrakis(pentafluorophenyl)borate - [1][$BArF_{20}$]

2.2.1 Synthesis

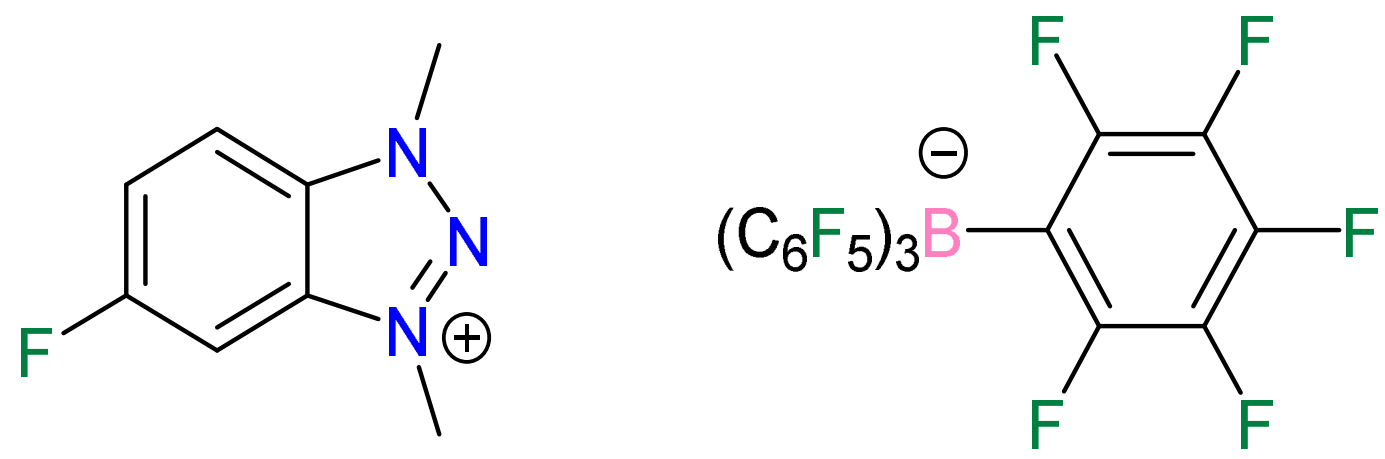

Compound [**1**][$BArF_{20}$] was prepared following the general procedure.

Yield: 0.171 g (2.4 %).

Elemental analysis, Found: C, 45.13; H, 0.85; N, 4.70%. **Calc.** for $C_{32}H_9BF_{21}N_3$: C, 45.47; H, 1.07; N, 4.97%.

HR-ESI-MS [$C_8H_9FN_3$]$^+$: 166.0771 (calc. 166.0776).

^{1}H NMR (500 MHz, CD_3CN): δ = 8.12 (dd, J = 9.4, 4.1 Hz, 1H), 7.84 (dd, J = 7.6, 2.2 Hz, 1H), 7.77 (td, J = 9.3, 2.2 Hz, 1H), 4.51 (s, 3H), 4.46 (s, 3H) ppm.

^{13}C{^{1}H} NMR (126 MHz, CD_3CN): δ = 164.43 (d, $^1J_{CF}$ = 254.0 Hz, Ar*C*F), 149.11 (br d, J = 237.4 Hz, *o*-C_6F_5), 139.30 (br d, J = 245.9 Hz, *p*-C_6F_5), 137.35 (br d, J = 244.4 Hz, *m*-C_6F_5), 137.34 (d, $^3J_{CF}$ = 14.6 Hz), 133.73 (s, Ar*C*N), 125.83 (s, *ipso*-C_6F_5), 122.84 (d, $^2J_{CF}$ = 28.6 Hz), 117.03 (d, $^3J_{CF}$ = 11.0 Hz, Ar*C*N), 100.28 (d, $^2J_{CF}$ = 29.6 Hz), 39.14 (s, N*C*H_3), 38.94 (s, N*C*H_3) ppm.

$^{11}B\{^{1}H\}$ NMR (160 MHz, CD_3CN): δ = –16.69 ppm.

$^{19}F\{^{1}H\}$ NMR (470 MHz, CD_3CN): δ = –106.40 (s, 1F), –133.78 (s, 8F), –163.94 (t, *J* = 19.5 Hz, 4F), –168.36 (t, *J* = 18.2 Hz, 8F) ppm.

^{19}F NMR (470 MHz, CD_3CN): δ = –106.36 - –106.41 (m, 1F), –133.76 (s, 8F), –163.96 (t, *J* = 19.6 Hz, 4F), –168.38 (t, *J* = 18.7 Hz, 8F) ppm.

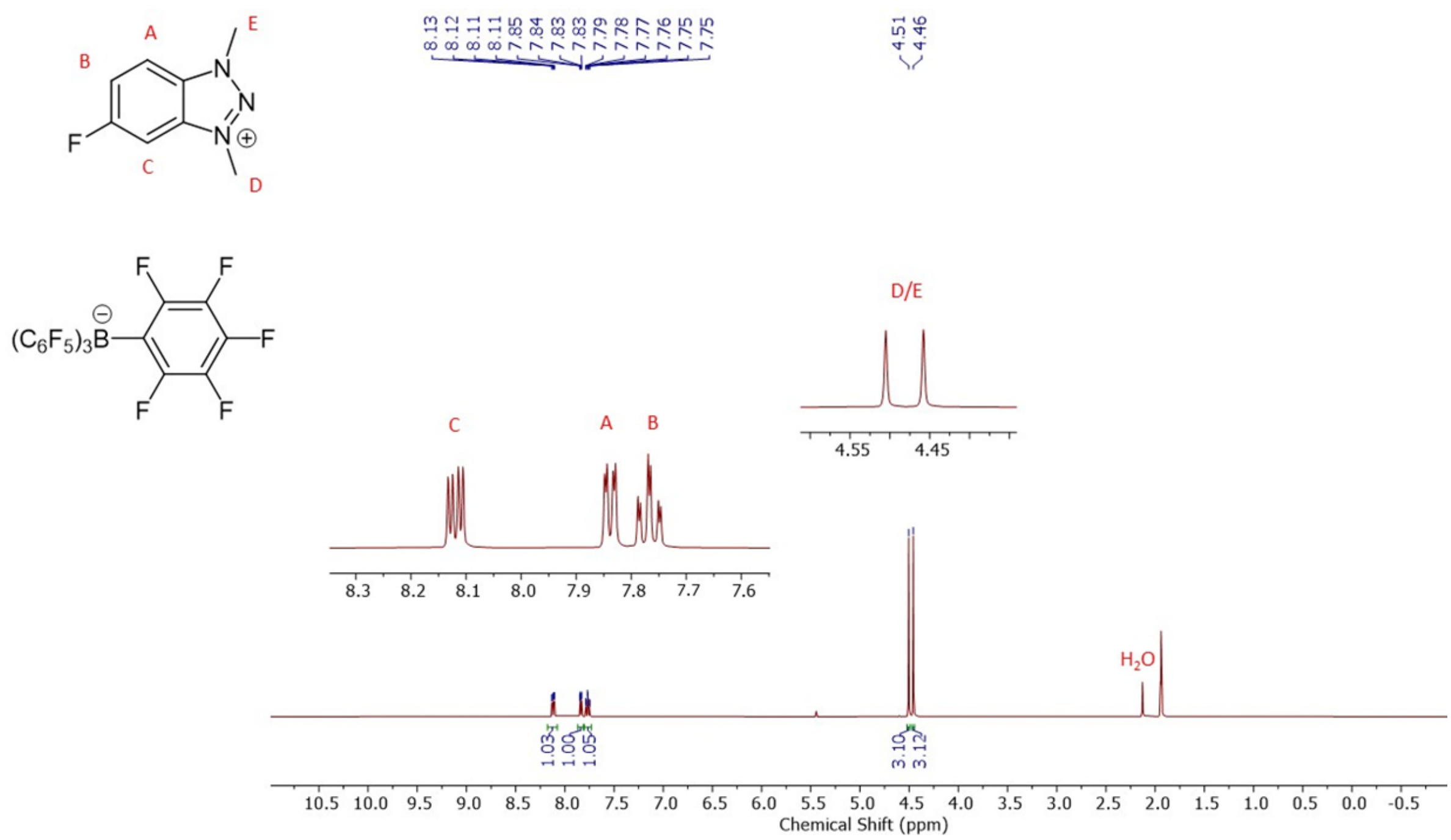

Figure S1. ^{1}H NMR spectrum (CD_3CN, 500 MHz) of [**1**][$BArF_{20}$] at room temperature.

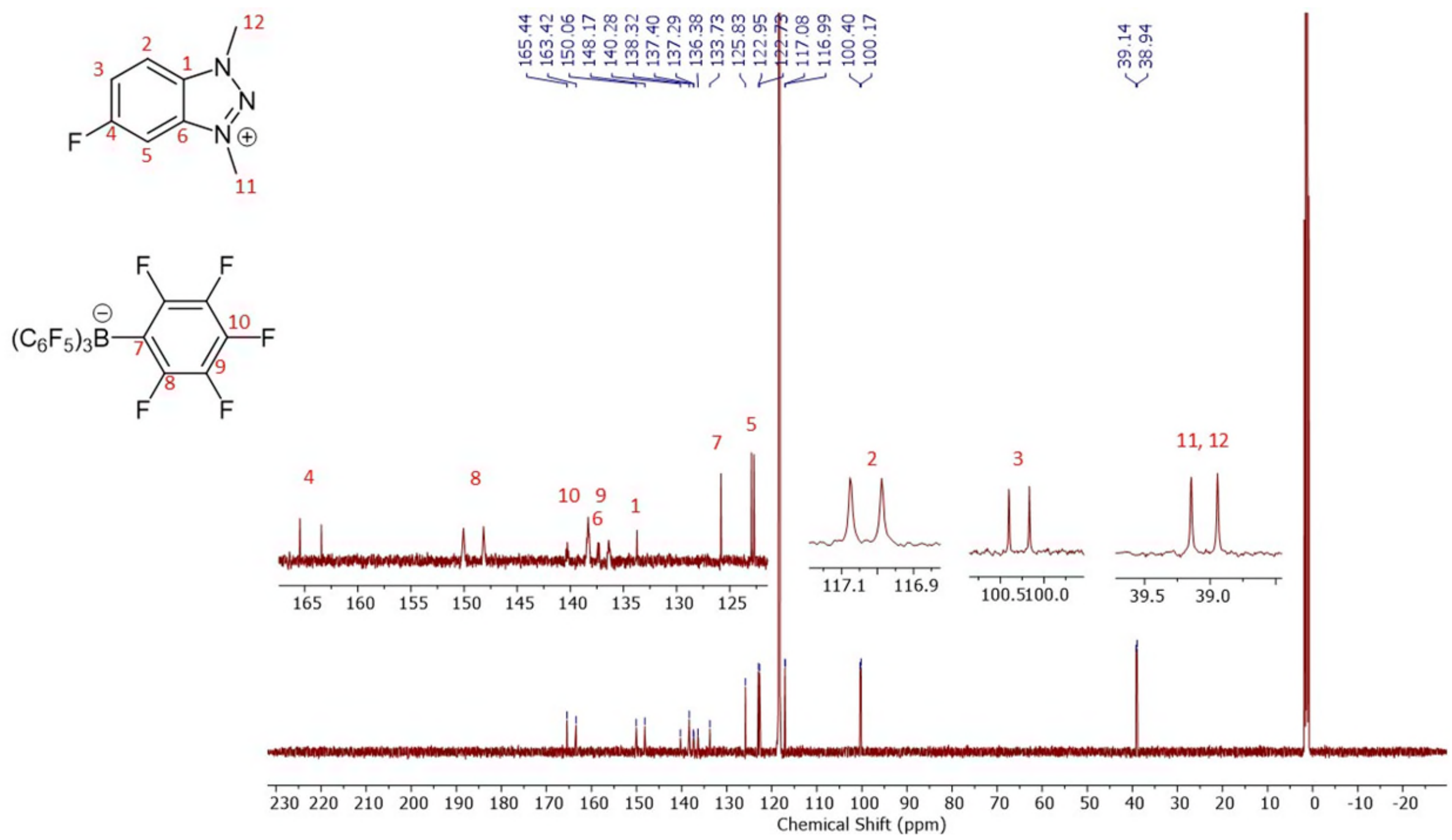

Figure S2. $^{13}C\{^{1}H\}$ NMR spectrum (CD_3CN, 126 MHz) of [**1**][$BArF_{20}$] at room temperature.

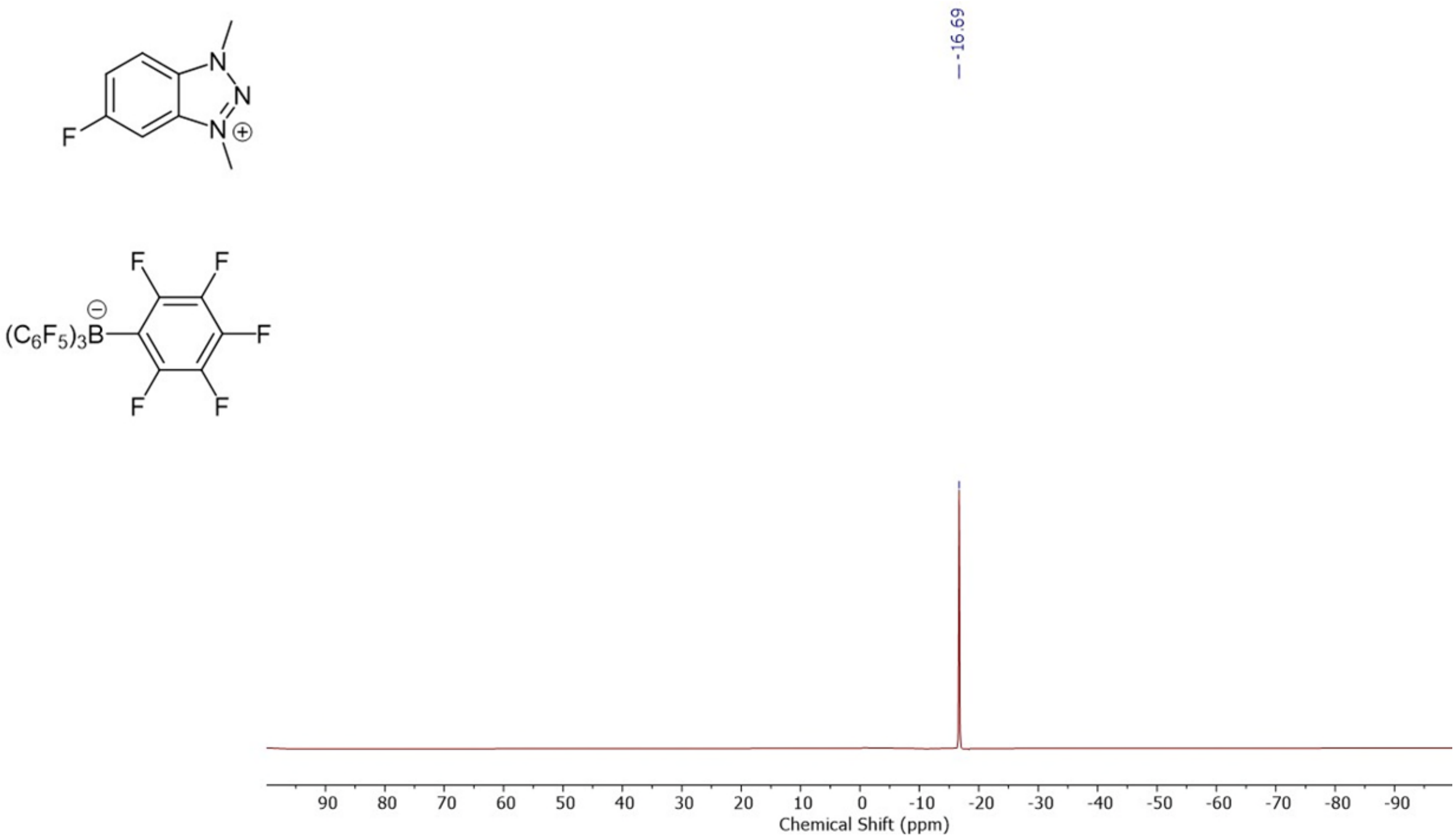

Figure S3. $^{11}B\{^{1}H\}$ NMR spectrum (CD_3CN, 160 MHz) of [**1**][$BArF_{20}$] at room temperature.

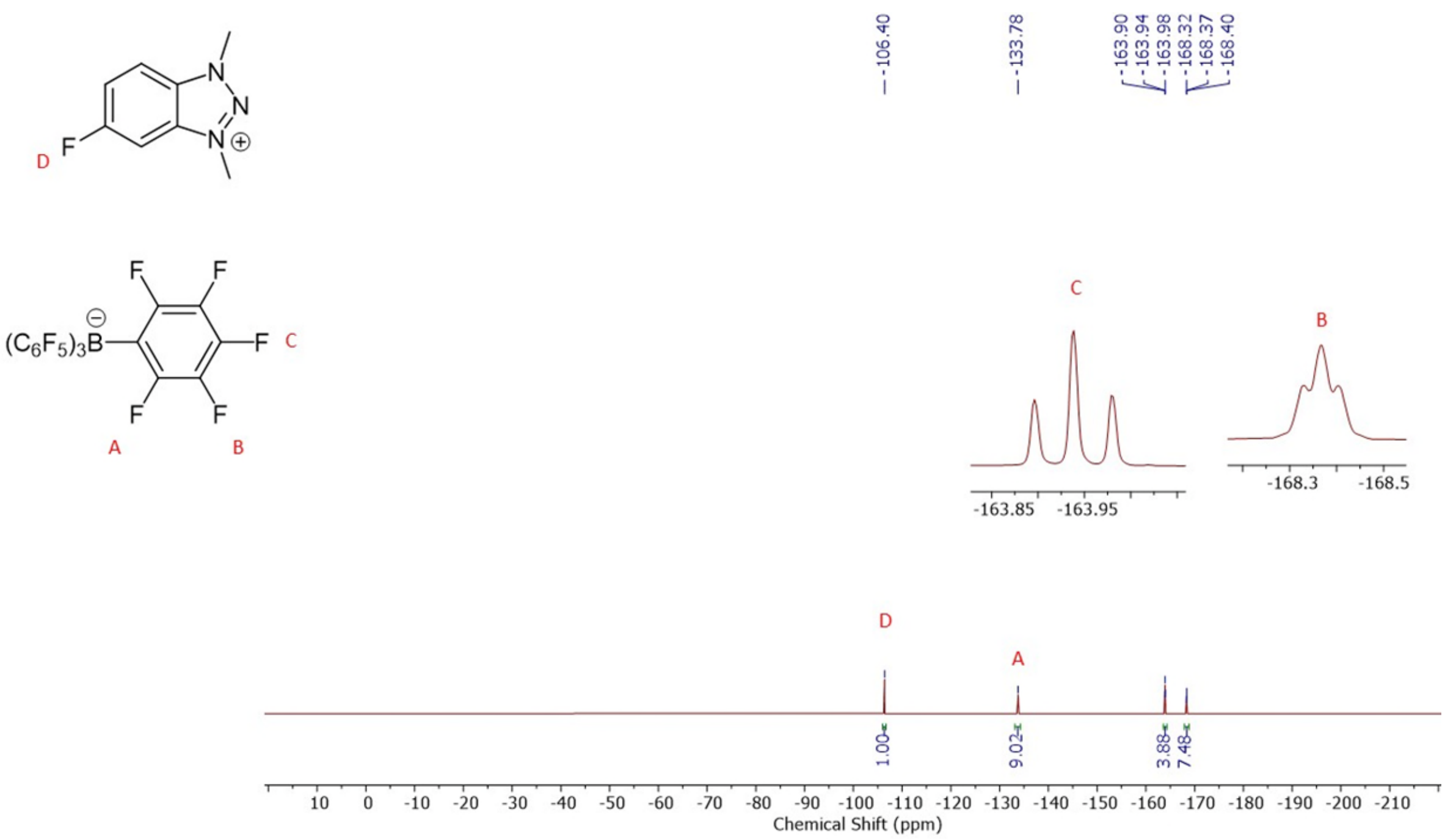

Figure S4. $^{19}F\{^{1}H\}$ NMR spectrum (CD_3CN, 470 MHz) of [**1**][$BArF_{20}$] at room temperature.

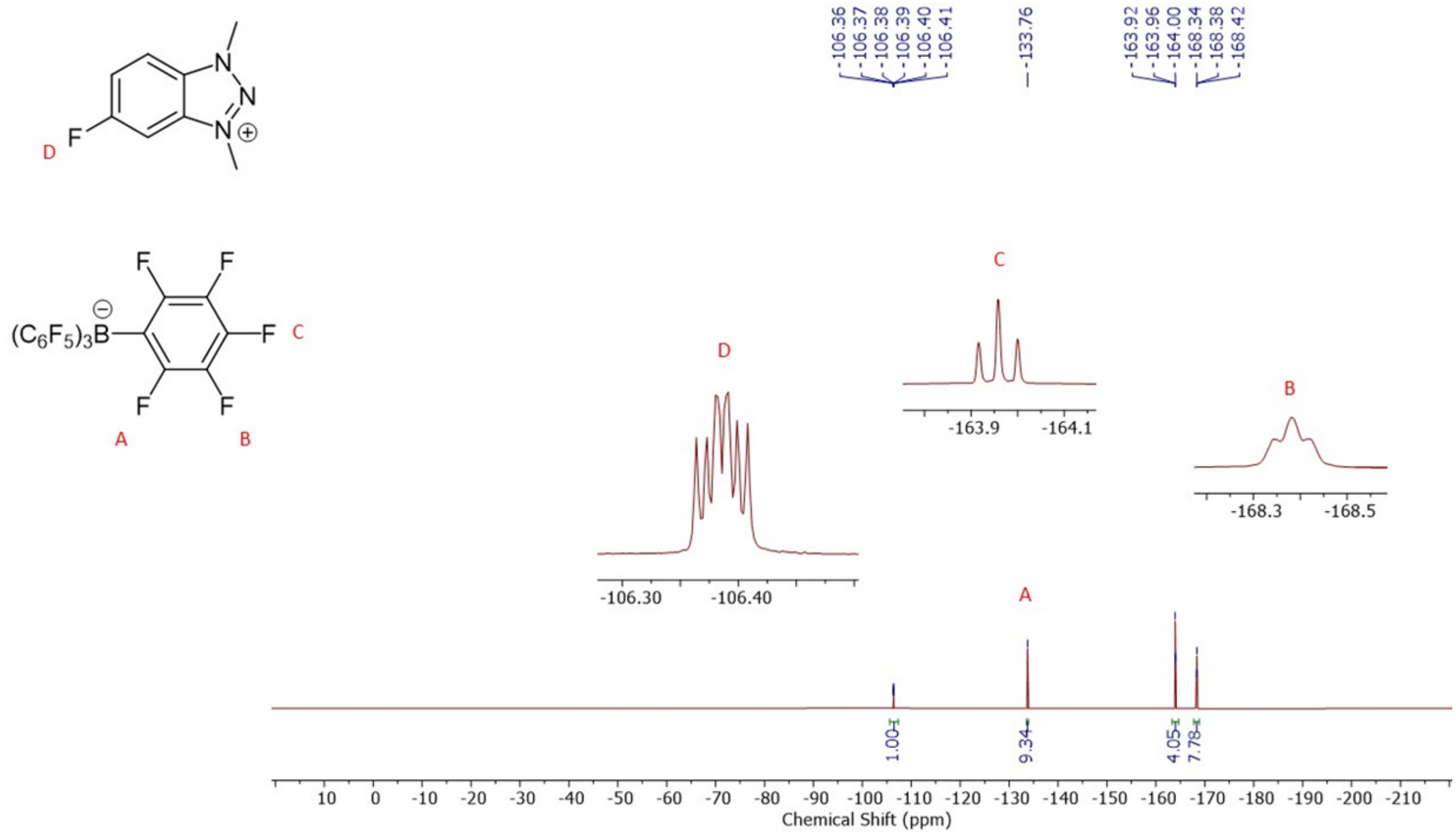

Figure S5. ^{19}F NMR spectrum (CD_3CN, 470 MHz) of [**1**][$BArF_{20}$] at room temperature.

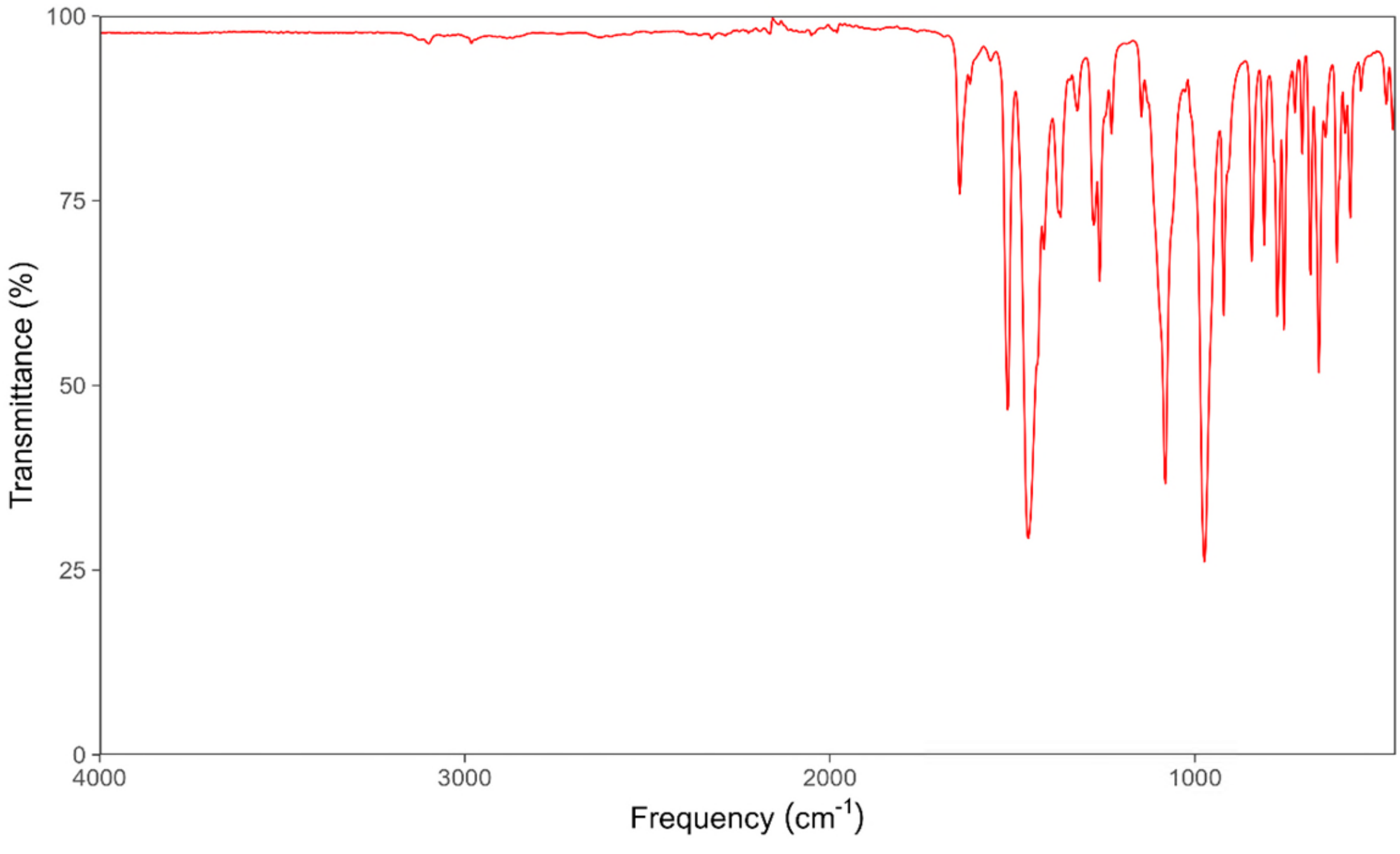

Figure S6. Experimental IR spectrum of [**1**][$BArF_{20}$].

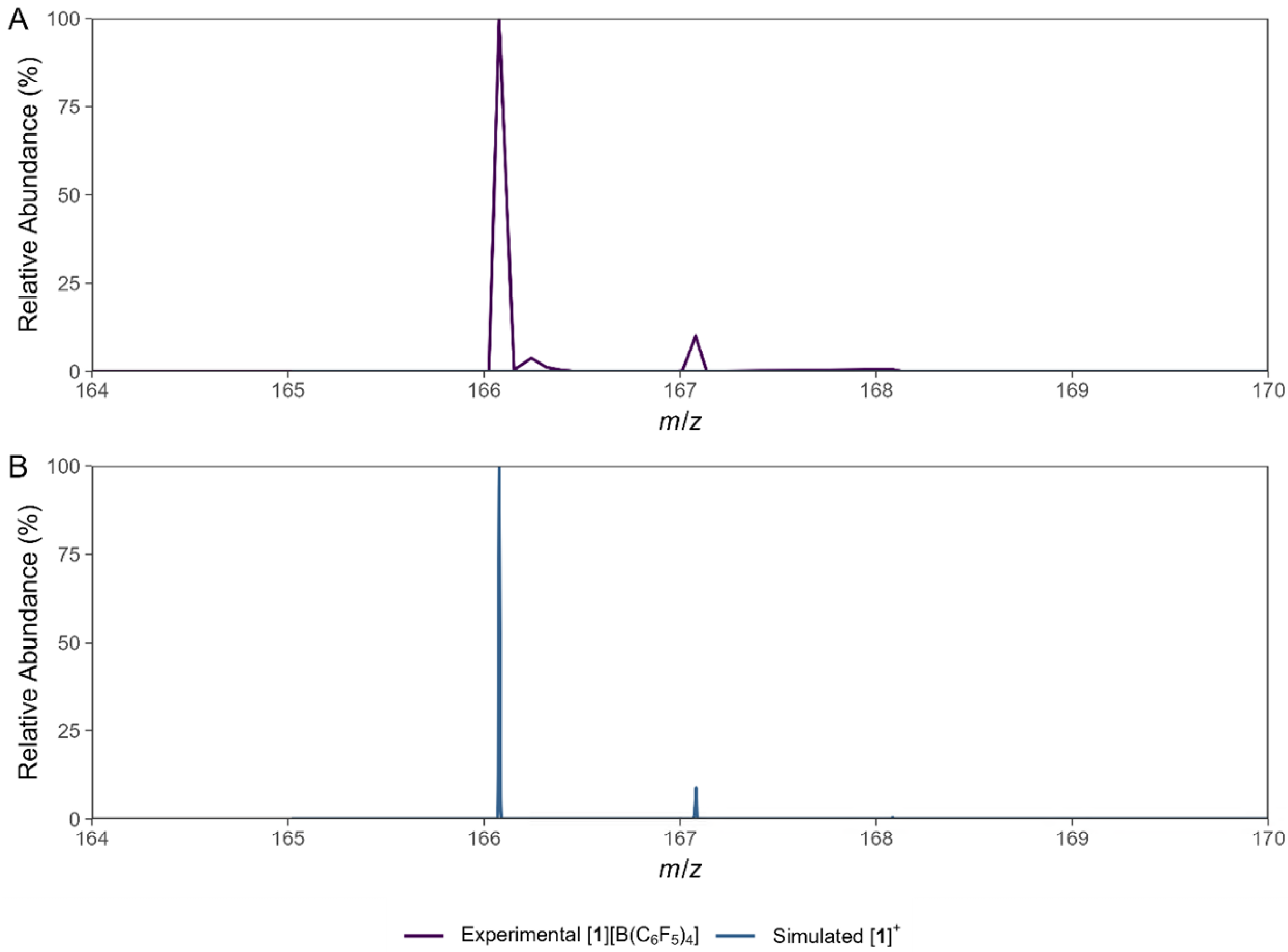

Figure S7. (A) Experimental HR-ESI-MS spectrum for [**1**][BArF$_{20}$]. (B) Simulated HR-ESI-MS spectrum for [**1**]$^{+}$.

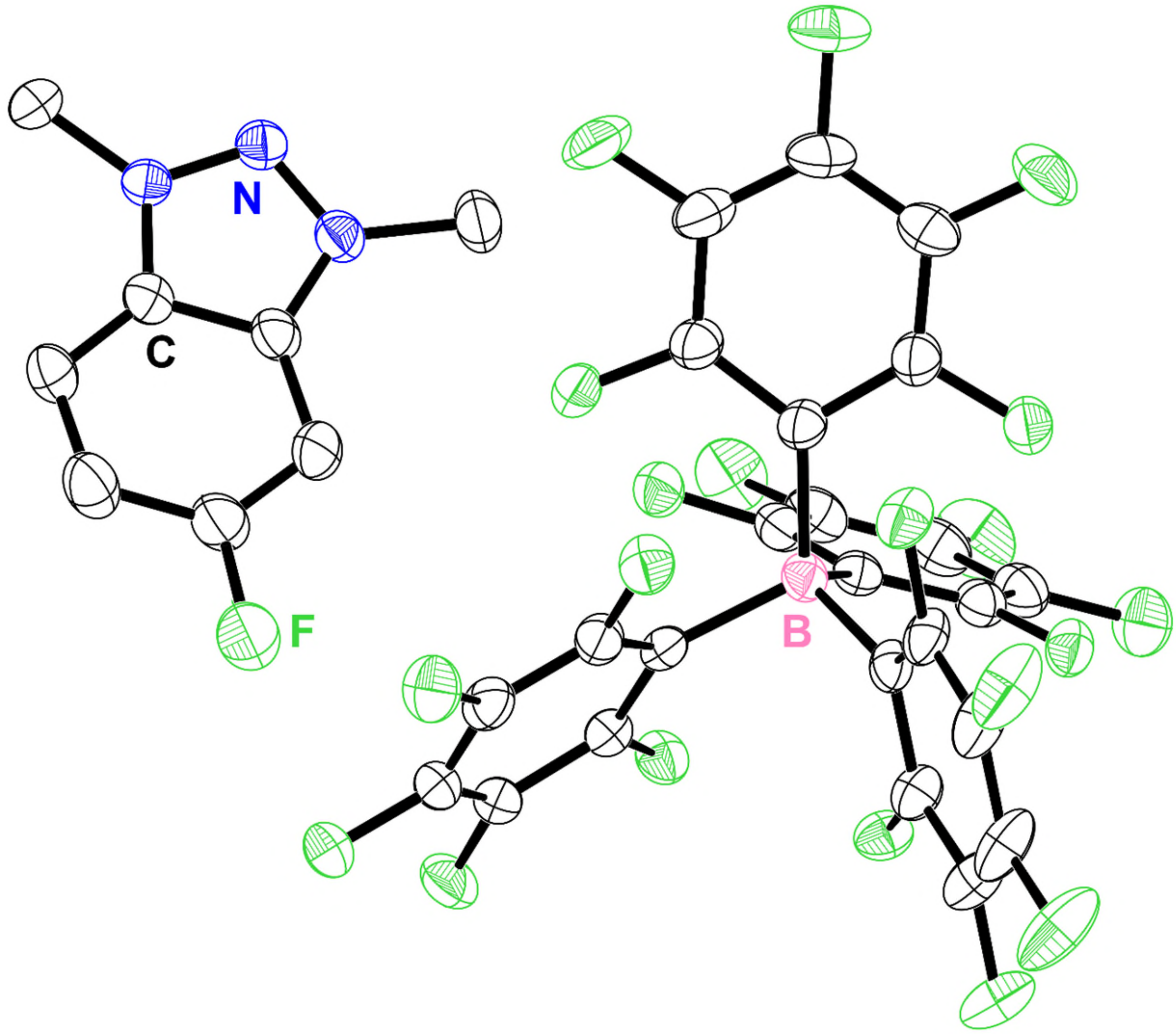

Figure S8. Thermal ellipsoid plot (50% probability) of [**1**][$BArF_{20}$]. H atoms, disordered components, and solvent molecules are omitted for clarity. Only one molecular unit from the asymmetric unit is displayed. Colour code: N blue, B pink, C black, F light green.

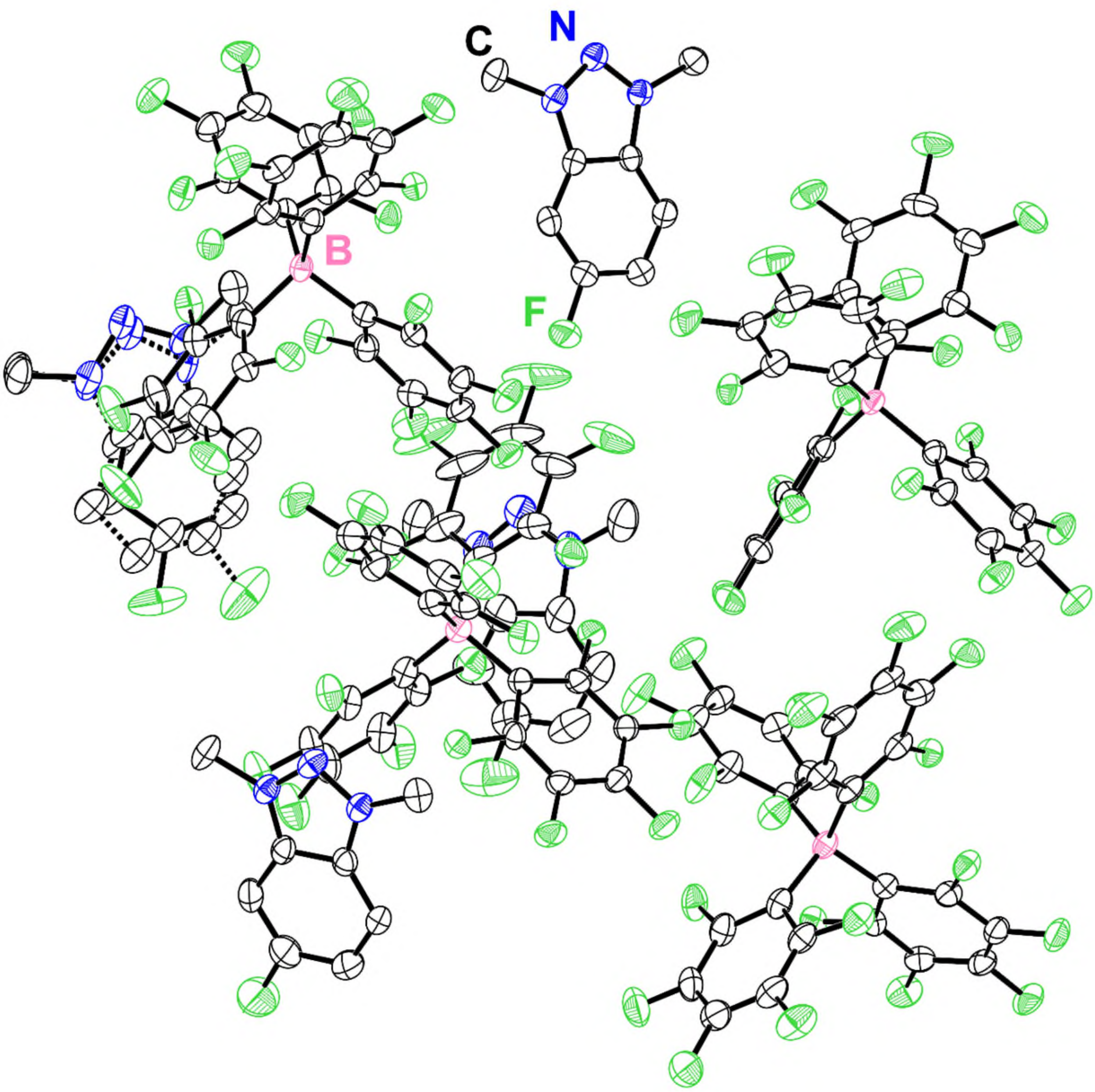

Figure S9. Thermal ellipsoid plot (50% probability) of [**1**][BArF$_{20}$]. H atoms are omitted for clarity. Colour code: N blue, B pink, C black, F light green.

2.2.2 Cyclic Voltammetry

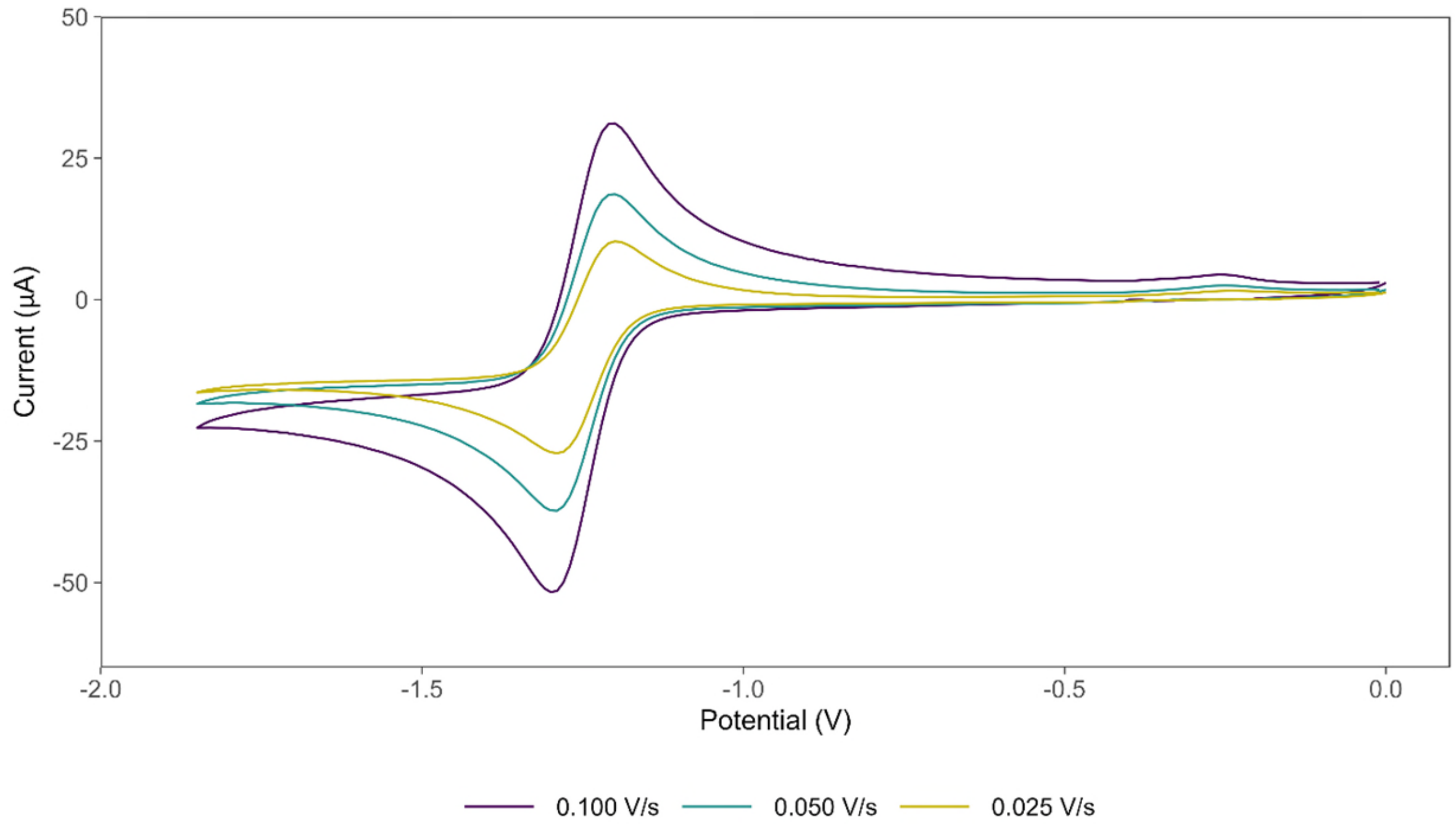

Figure S10. Cyclic voltammetry of [**1**][$BArF_{20}$]. Experimental details: Analyte; 2 mM. Electrolyte; 100 equiv. [Bu_4N][PF_6]. Solvent; acetonitrile. Working electrode; glassy carbon. Counter electrode; platinum. Reference electrode; leak-proof Ag/AgCl.

2.2.3 Electron Paramagnetic Resonance and Spin Density Data

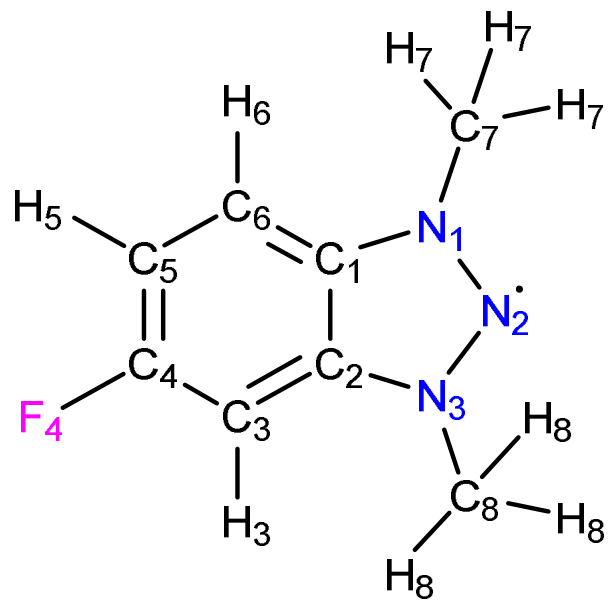

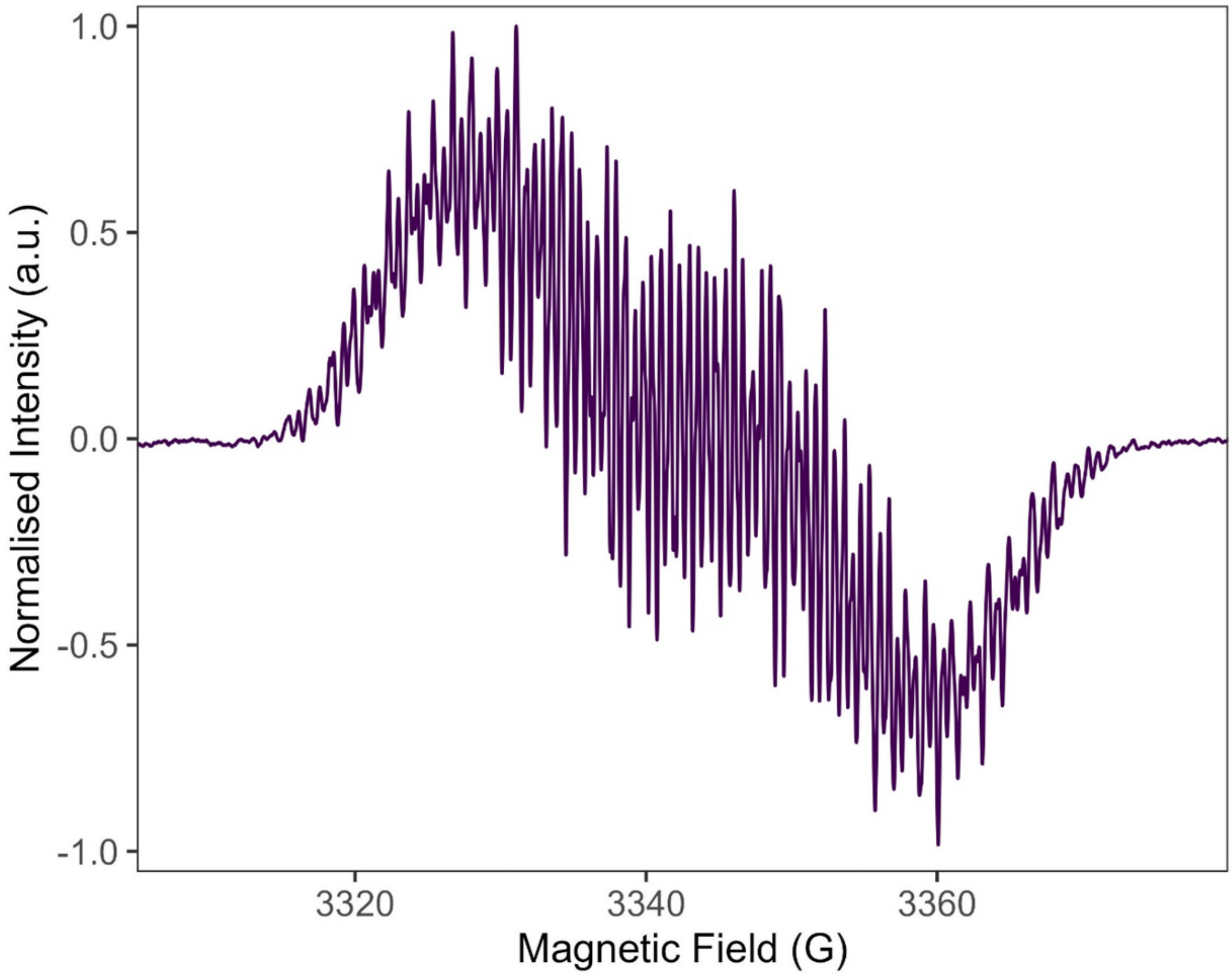

Figure S11. Electron paramagnetic resonance spectrum of [**1**][$BArF_{20}$] after reduction by KC_8. Parameters: Solvent: toluene. Scans: 50. Modulation amplitude: 0.1 G. Frequency: 9.377 GHz. Temperature: 298 K. Sample was prepared according to the general procedure in Section 2.1.2.

Table S1. Spin densities (B3LYP/def2-TZVP) of **1**$^{\bullet}$.

Atom	Mulliken Spin Density	Calculated A_{iso}
N1	0.103	6.554
N2	0.524	21.389
N3	0.107	4.490
C1	–0.052	NA
C2	–0.046	NA
C3	0.137	NA
C4	0.063	NA
C5	0.037	NA
C6	0.145	NA
C7	–0.007	NA
C8	–0.007	NA
F4	–0.009	2.929
H3	–0.006	9.712
H5	–0.001	6.501
H6	–0.007	12.618
3 × H7	0.003	6.213
3 × H8	0.003	4.276

2.3 5-Chloro-1,3-dimethyl-1H-benzo[d][1,2,3]triazol-3-ium tetrakis(pentafluorophenyl)borate - [2][BArF$_{20}$]

2.3.1 Synthesis

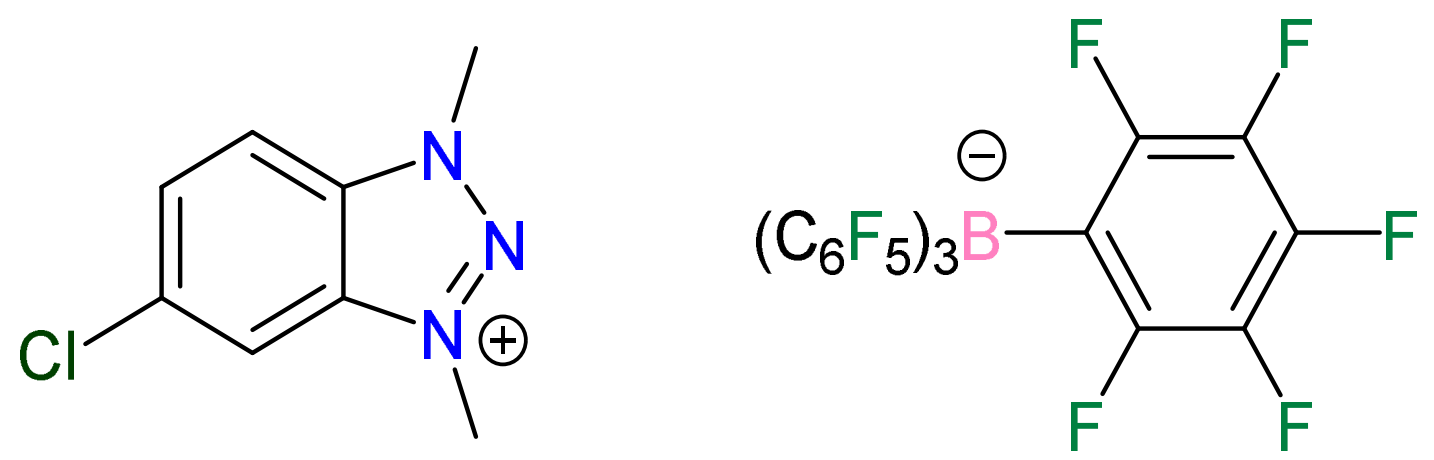

Compound [**2**][BArF$_{20}$] was prepared following the general procedure.

Yield: 0.244 g (3.9 %).

Elemental analysis, Found: C, 45.13; H, 0.89; N, 4.39%. **Calc.** for $C_{32}H_9BF_{20}N_3Cl$: C, 44.61; H, 1.05; N, 4.88%.

HR-ESI-MS [$C_8H_9ClN_3$]$^+$: 182.0474 (calc. 182.0480).

^{1}H NMR (500 MHz, CD_3CN): δ = 8.20 (d, *J* = 1.7 Hz, 1H), 8.07 (d, *J* = 9.1 Hz, 1H), 7.92 (dd, *J* = 9.1, 1.7 Hz, 1H), 4.50 (s, 3H), 4.47 (s, 3H) ppm.

^{13}C{^{1}H} NMR (126 MHz, CD_3CN): δ = 149.10 (d, $^{1}J_{CF}$ = 243.9 Hz, *o*-C_6F_5), 139.30 (d, $^{1}J_{CF}$ = 245.9 Hz, *p*-C_6F_5), 138.24 (s, Ar*C*Cl), 137.29 (d, $^{1}J_{CF}$ = 238.4 Hz, *m*-C_6F_5), 137.08 (s, Ar*C*N), 135.40 (s, Ar*C*N), 133.29 (s, Ar*C*H), 125.82 (s, *ipso*-C_6F_5), 116.09 (s, Ar*C*H), 114.34 (s, Ar*C*H), 39.14 (s, N*C*H_3), 39.01 (s, N*C*H_3) ppm.

^{11}B{^{1}H} NMR (160 MHz, CD_3CN): δ = –16.69 ppm.

^{19}F NMR (470 MHz, CD_3CN): δ = –133.78 (s, 8F), –163.93 (t, *J* = 19.8 Hz, 4F), –168.36 (t, *J* = 18.7 Hz, 8F) ppm.

^{19}F{^{1}H} NMR (470 MHz, CD_3CN): δ = –133.77 (s, 8F), –163.95 (t, *J* = 19.8 Hz, 4F), –168.37 (t, *J* = 18.7 Hz, 8F) ppm.

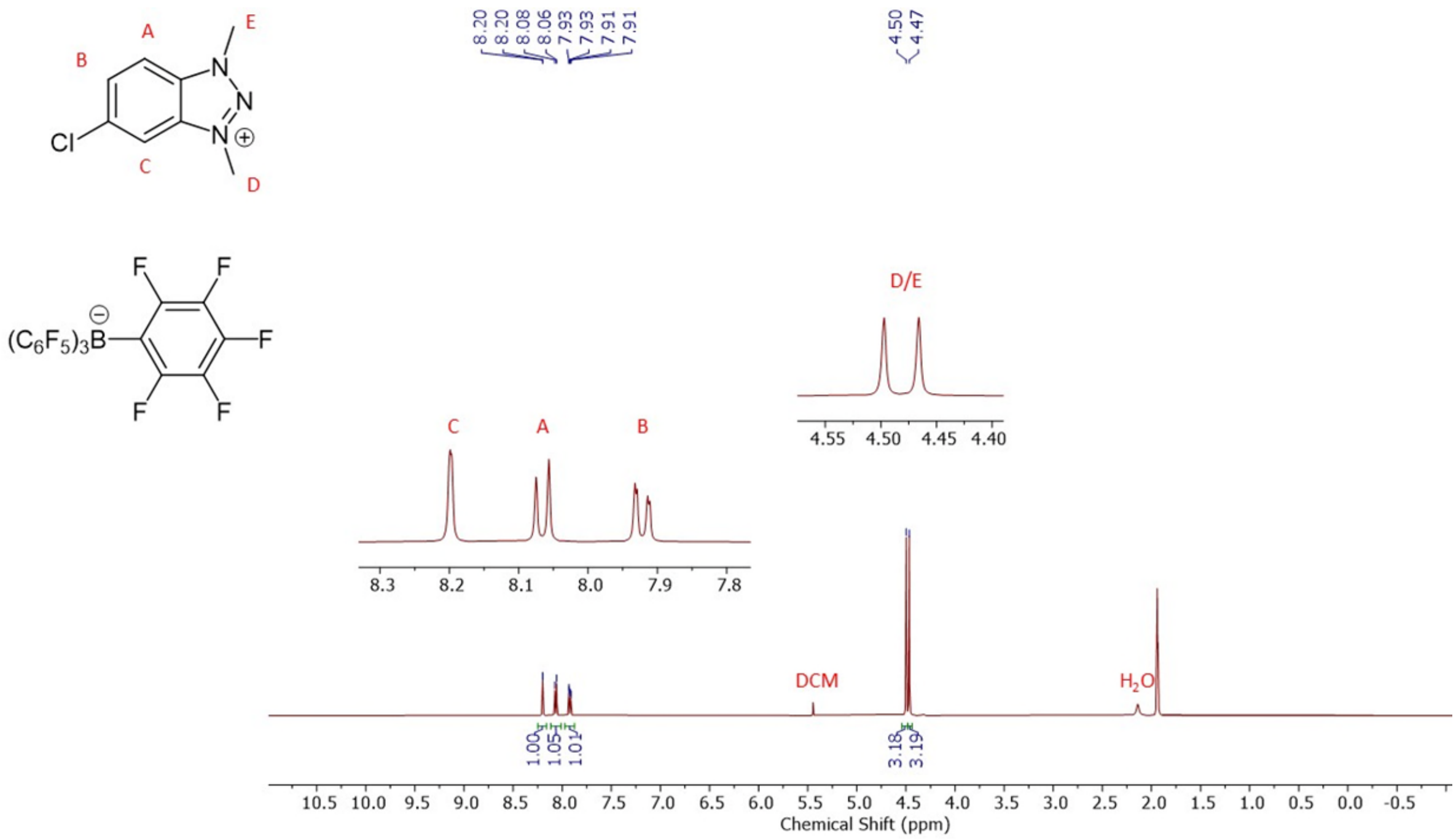

Figure S12. ^{1}H NMR spectrum (CD_3CN, 500 MHz) of [**2**][$BArF_{20}$] at room temperature.

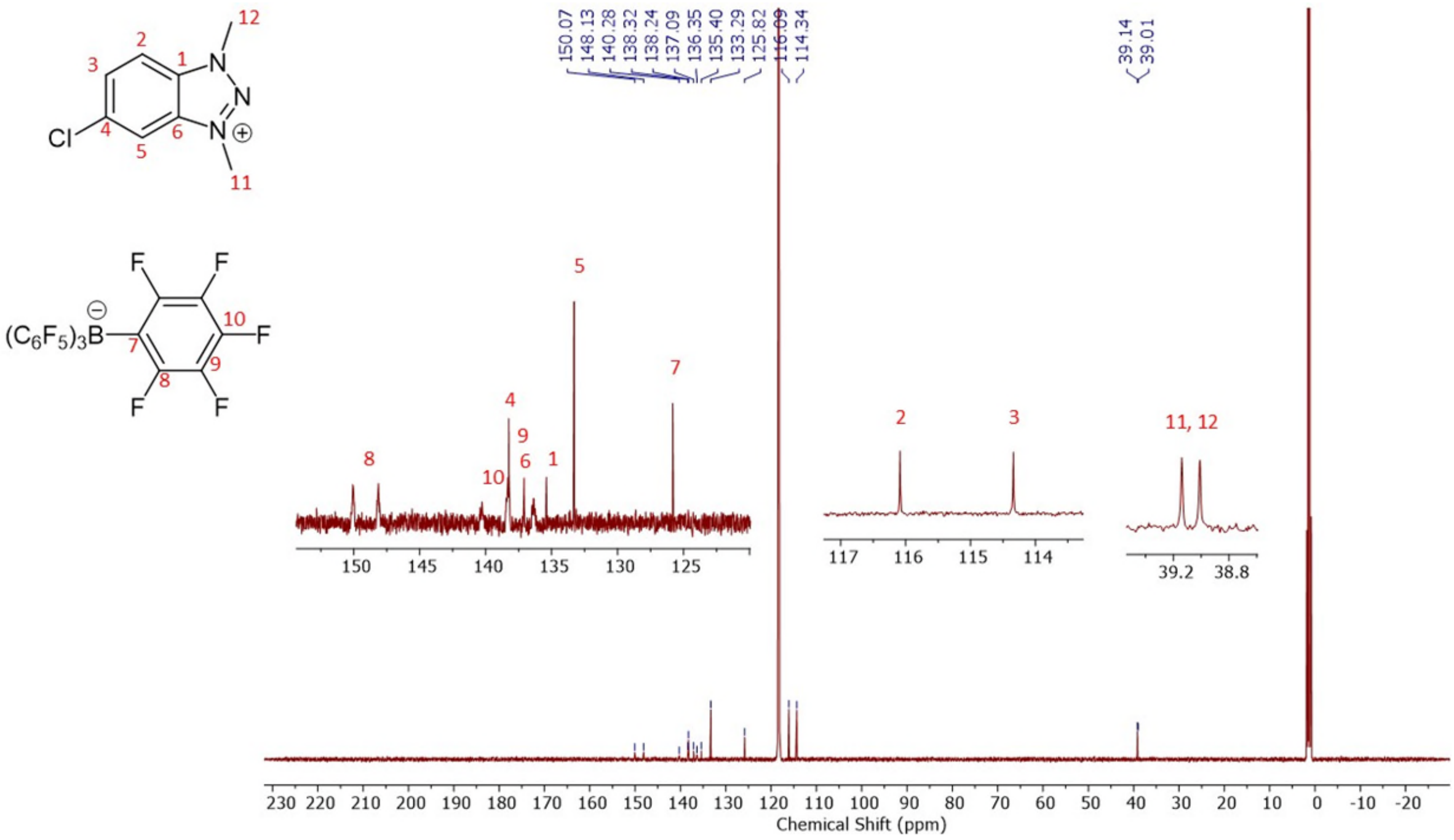

Figure S13. $^{13}C\{^{1}H\}$ NMR spectrum (CD_3CN, 126 MHz) of [**2**][$BArF_{20}$] at room temperature.

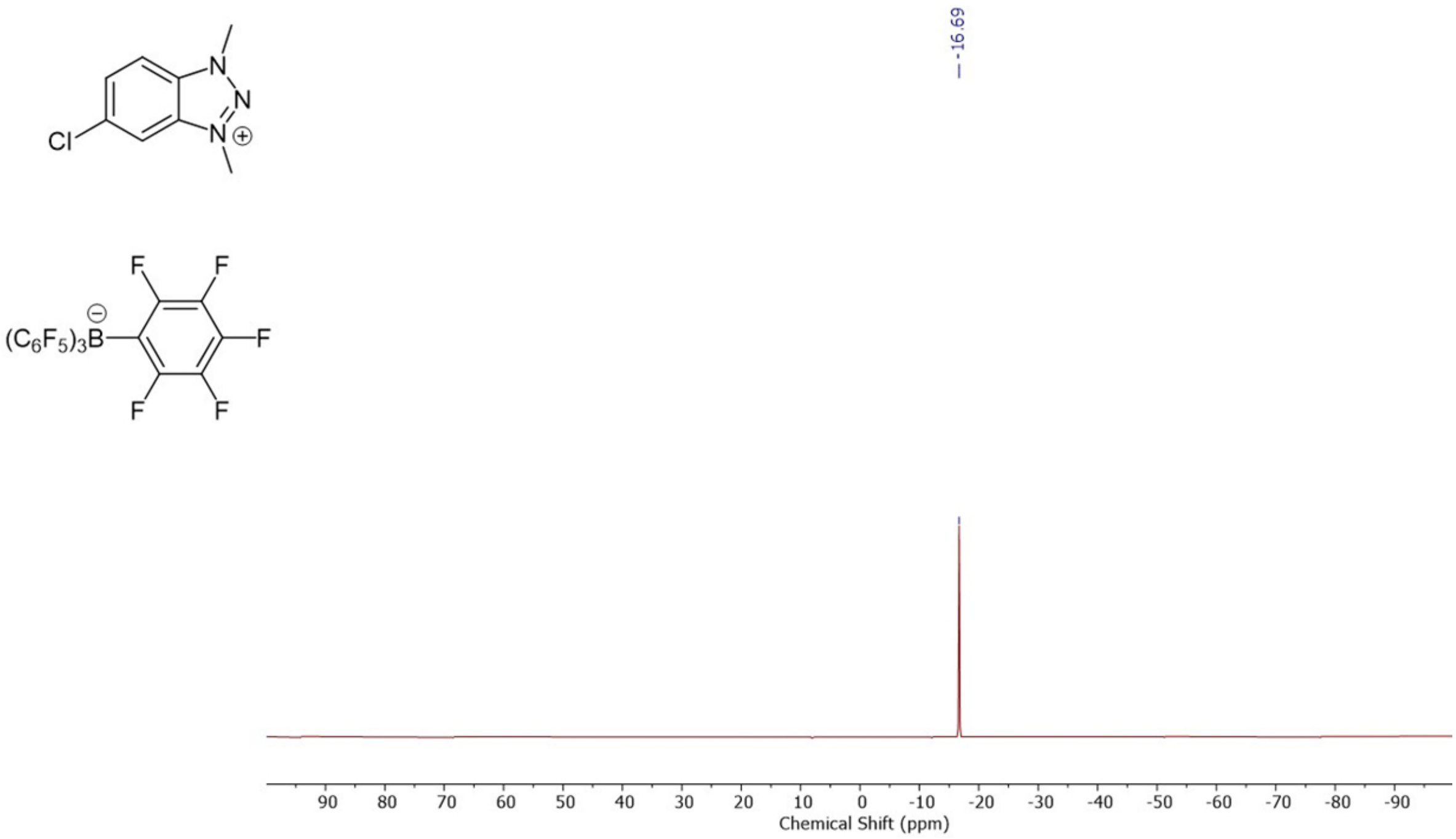

Figure S14. $^{11}B\{^{1}H\}$ NMR spectrum (CD_3CN, 160 MHz) of [**2**][$BArF_{20}$] at room temperature.

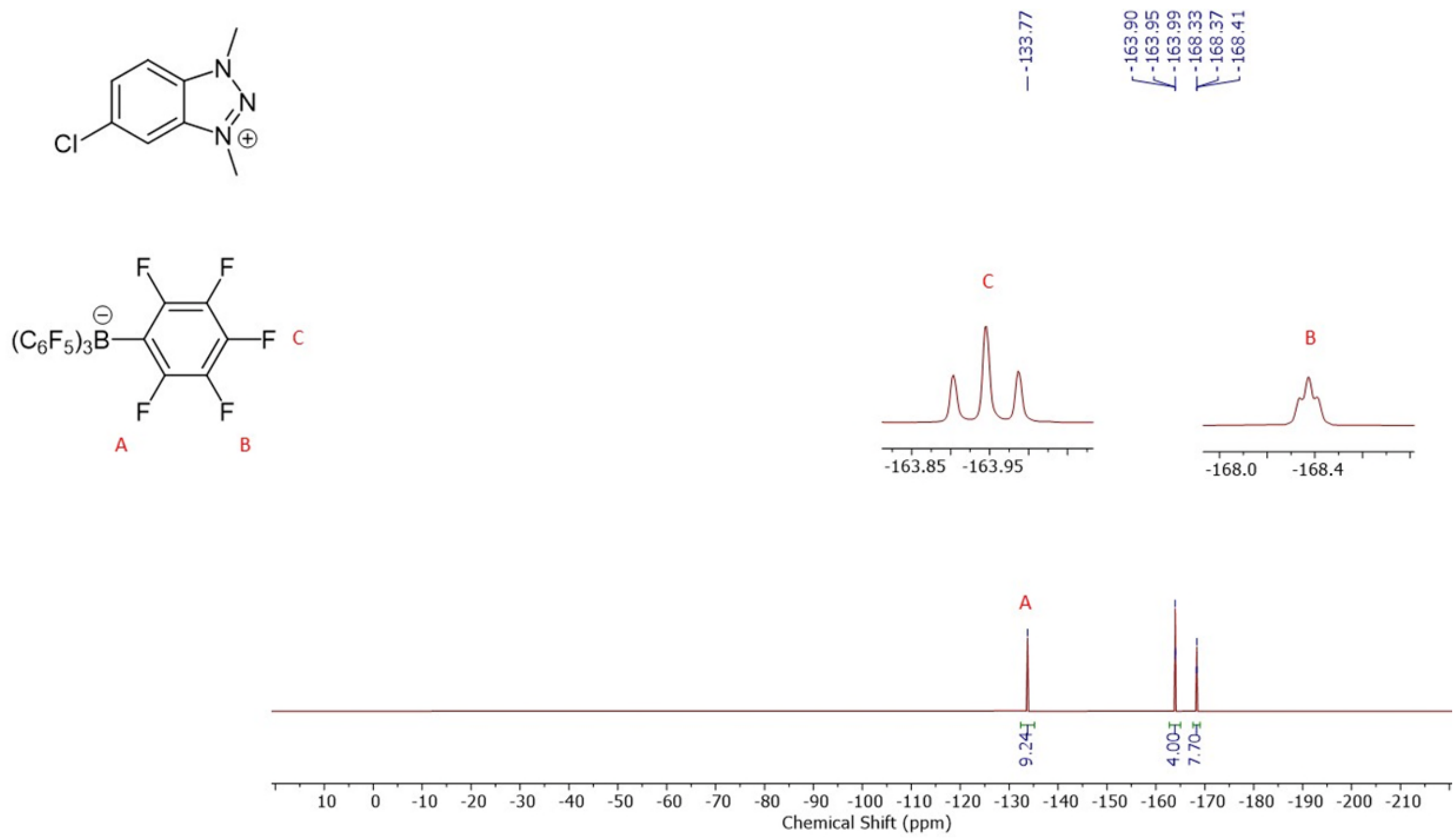

Figure S15. $^{19}F\{^{1}H\}$ NMR spectrum (CD_3CN, 470 MHz) of [**2**][$BArF_{20}$] at room temperature.

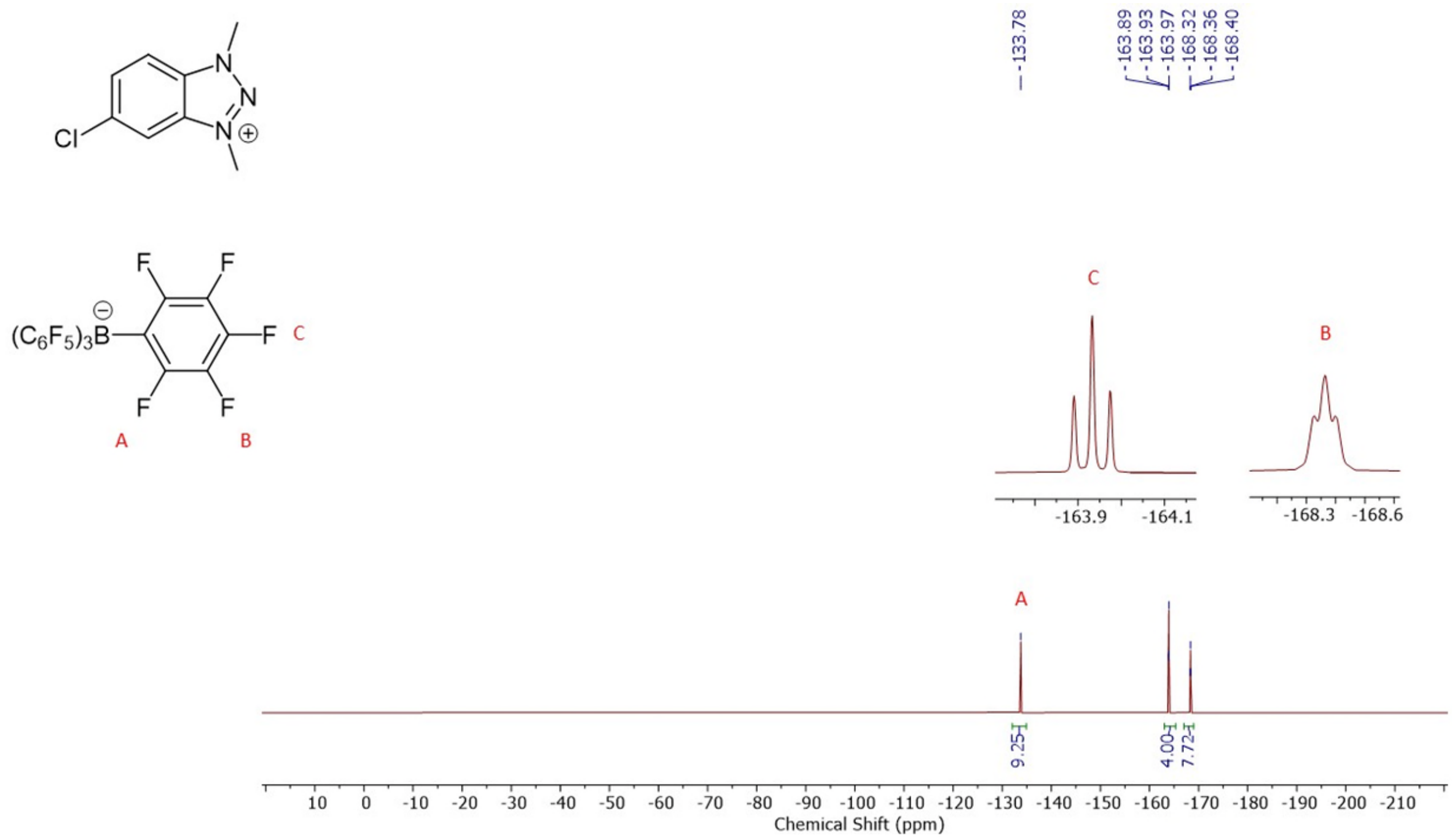

Figure S16. ^{19}F NMR spectrum (CD_3CN, 470 MHz) of [**2**][$BArF_{20}$] at room temperature.

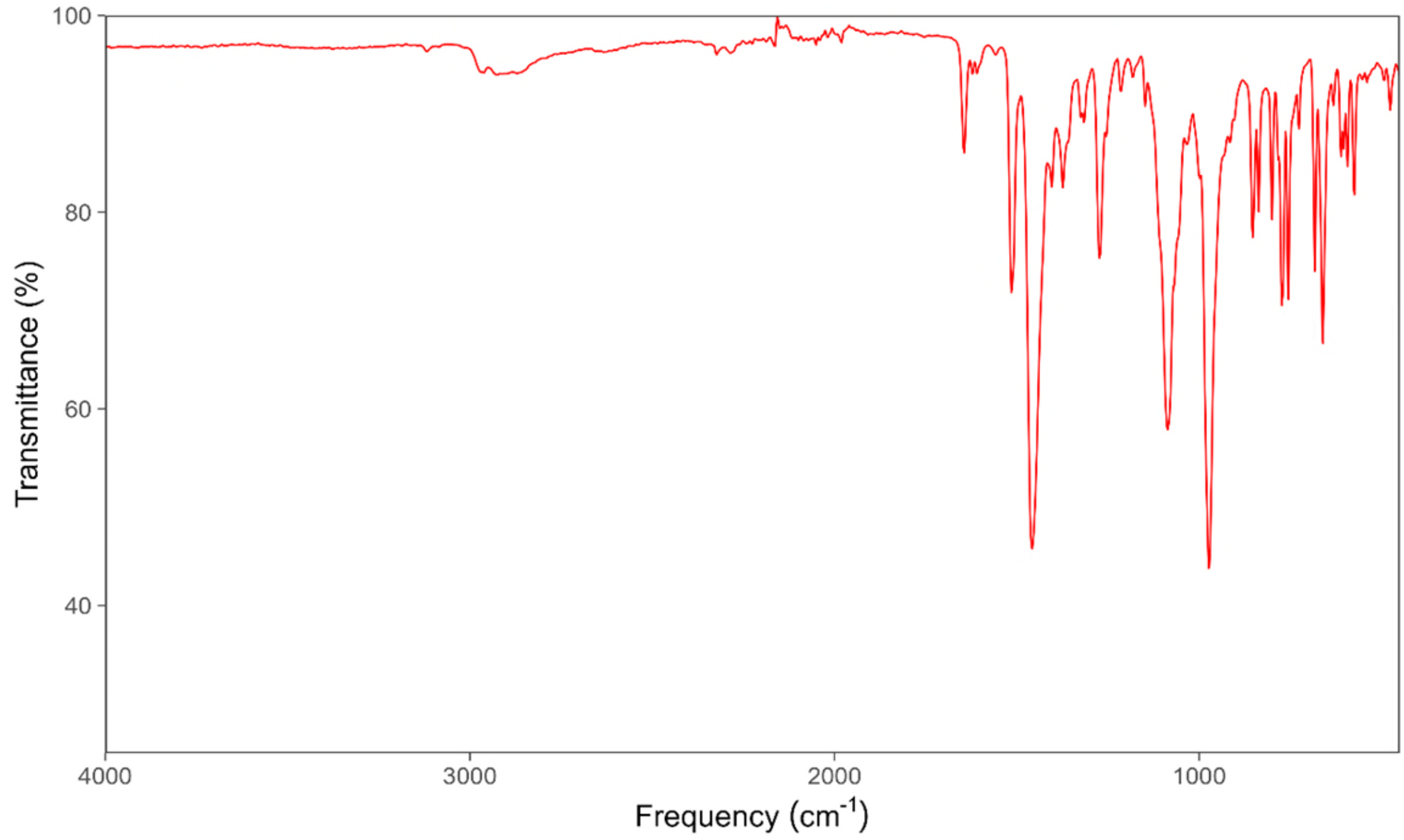

Figure S17. Experimental IR spectrum of [**2**][BArF20].

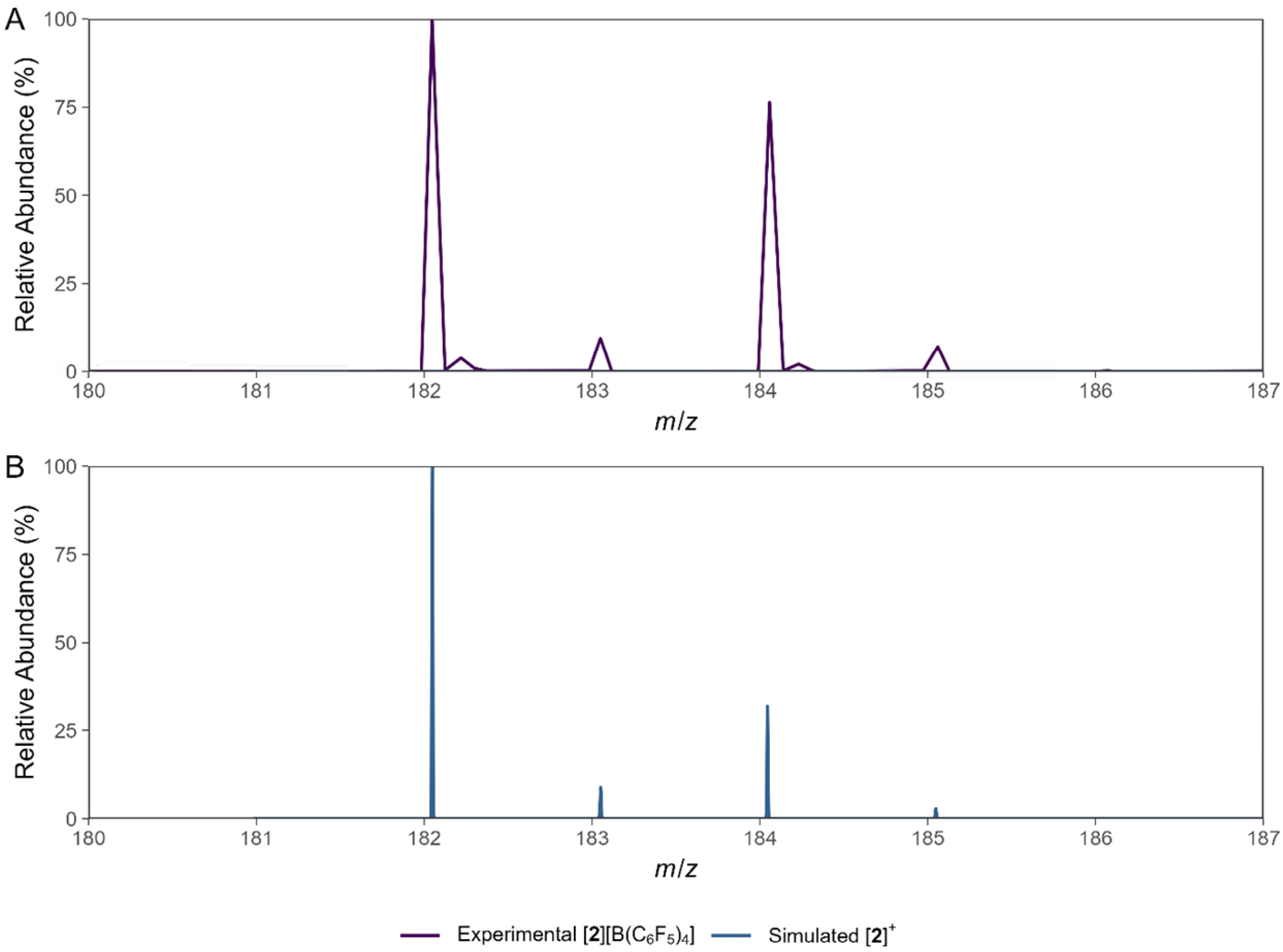

Figure S18. (A) Experimental HR-ESI-MS spectrum for [**2**][BArF20]. (B) Simulated HR-ESI-MS spectrum for [**2**]$^+$.

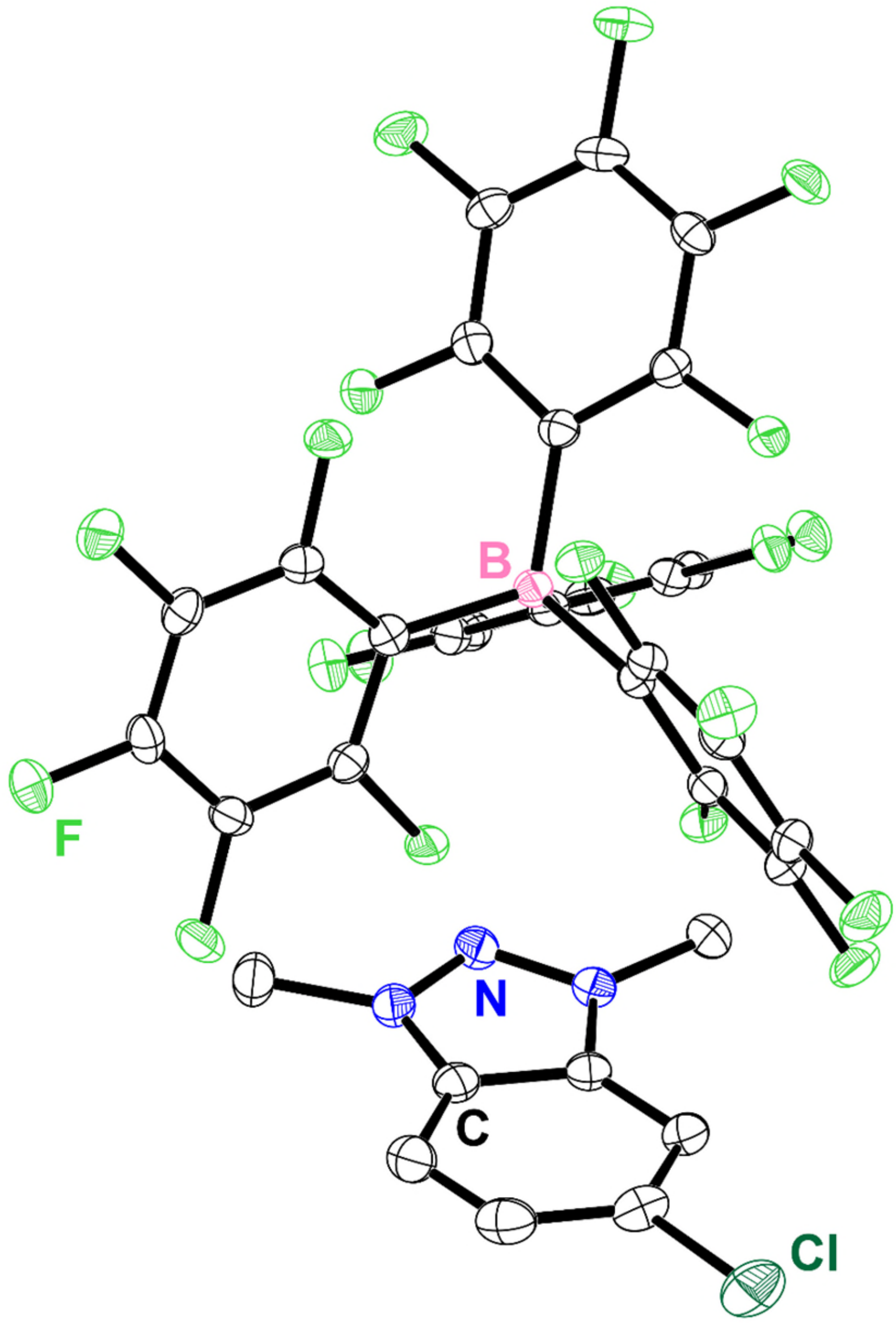

Figure S19. Thermal ellipsoid plot (50% probability) of [**2**][BArF20]. H atoms are omitted for clarity. Colour code: N blue, B pink, C black, F light green, Cl dark green.

2.3.2 Cyclic Voltammetry

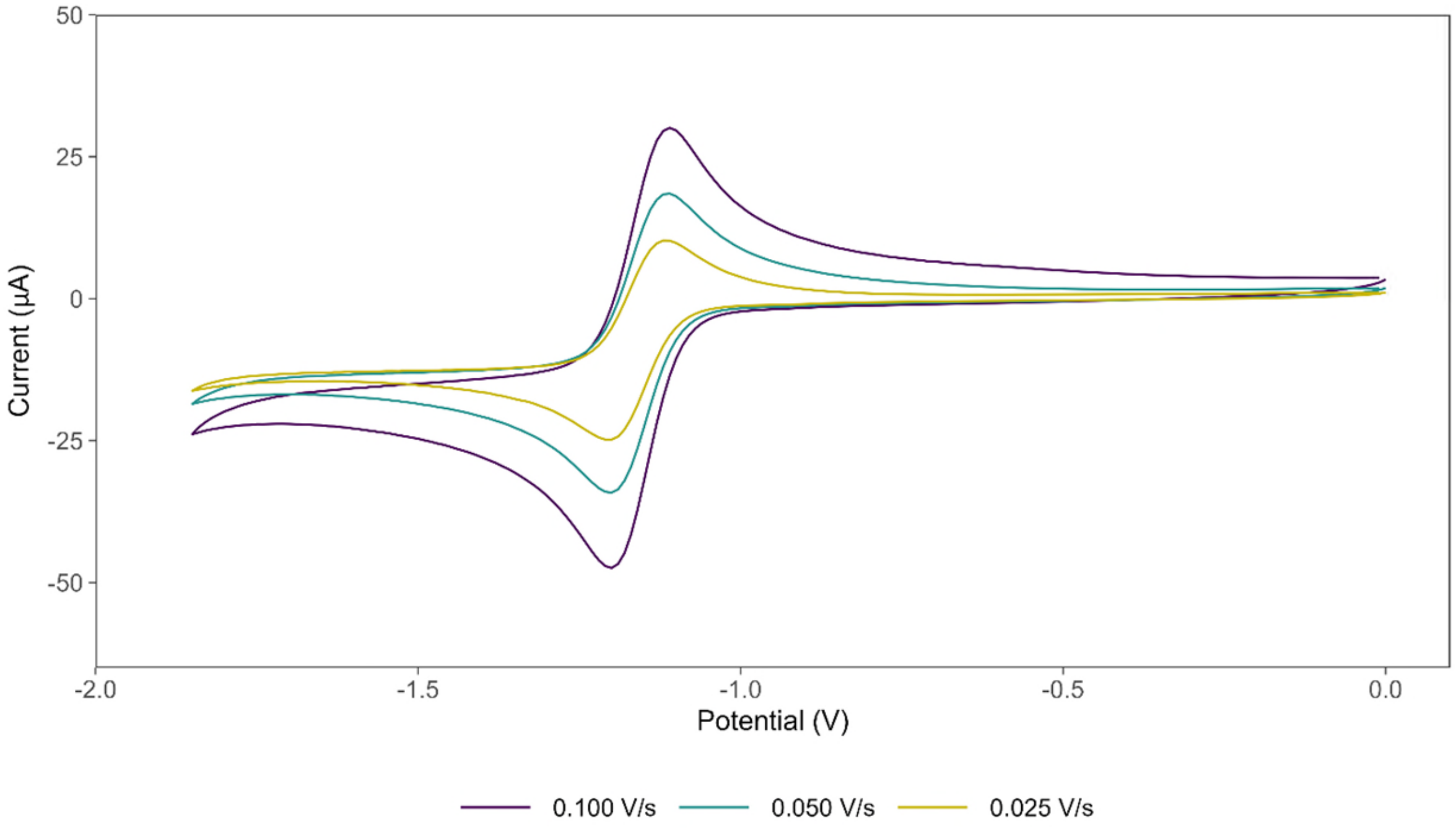

Figure S20. Cyclic voltammetry of [**2**][BArF$_{20}$]. Experimental details: Analyte; 2 mM. Electrolyte; 100 equiv. [Bu_4N][PF_6]. Solvent; acetonitrile. Working electrode; glassy carbon. Counter electrode; platinum. Reference electrode; leak-proof Ag/AgCl.

2.3.3 Electron Paramagnetic Resonance and Spin Density Data

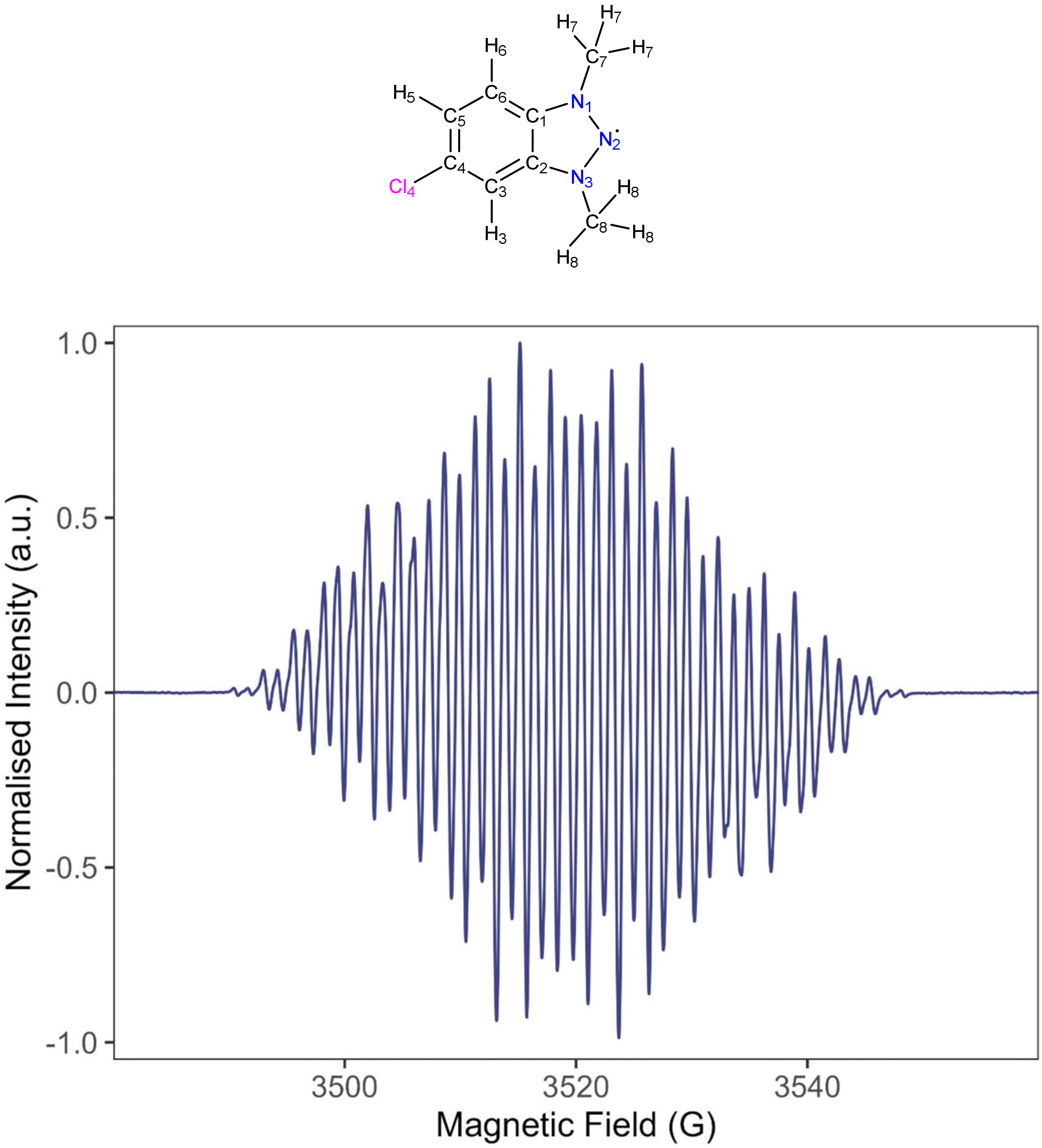

Figure S21. Electron paramagnetic resonance spectrum of [**2**][BArF$_{20}$] after reduction by KC_8. Parameters: Solvent: toluene. Scans: 10. Modulation amplitude: 0.2 G. Frequency: 9.867 GHz. Temperature: 298 K. Sample was prepared according to the general procedure in Section 2.1.2.

Table S2. Spin densities (B3LYP/def2-TZVP) of **2•**.

Atom	Mulliken Spin Density	Calculated A_{iso}
N1	0.108	5.424
N2	0.525	21.439
N3	0.107	5.044
C1	–0.051	NA
C2	–0.049	NA
C3	0.135	NA
C4	0.052	NA
C5	0.040	NA
C6	0.140	NA
C7	–0.007	NA
C8	–0.007	NA
Cl4	0.000	0.131
H3	–0.006	11.460
H5	–0.002	4.179
H6	–0.006	12.305
3 × H7	0.004	5.161
3 × H8	0.004	4.841

2.4 5-Bromo-1,3-dimethyl-1H-benzo[d][1,2,3]triazol-3-ium tetrakis(pentafluorophenyl)borate - [3][BArF$_{20}$]

2.4.1 Synthesis

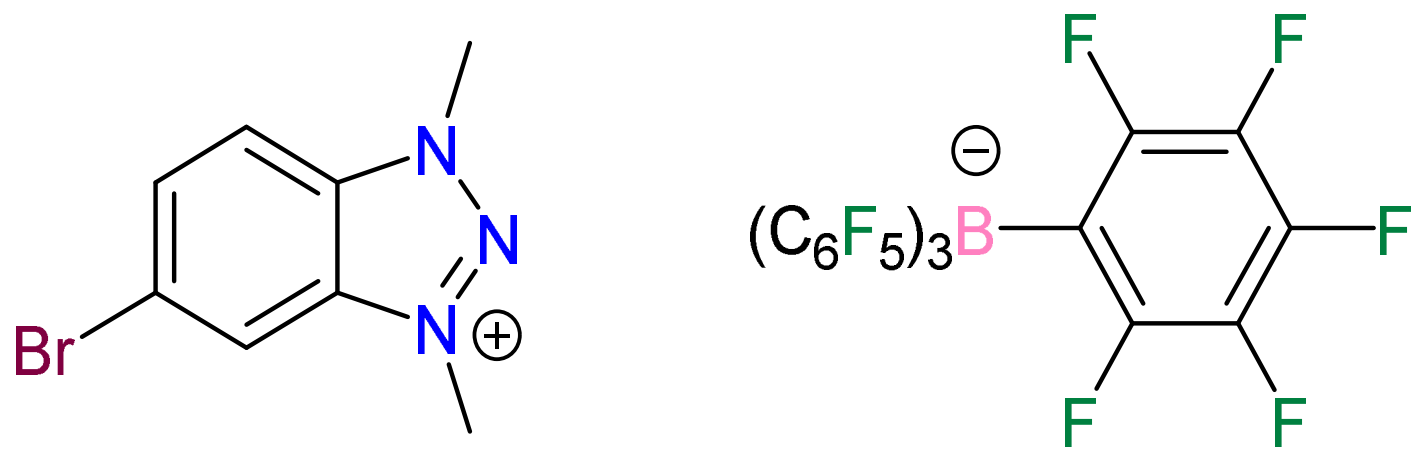

Compound [**3**][BArF$_{20}$] was prepared following the general procedure.

Yield: 0.217 g (4.3 %).

Elemental analysis, Found: C, 42.90; H, 1.14; N, 4.46%. **Calc.** for $C_{32}H_9BF_{20}N_3Br$: C, 42.42; H, 1.00; N, 4.64%.

HR-ESI-MS [$C_8H_9BrN_3$]$^+$: 225.9997 (calc. 225.9975).

^{1}H NMR (500 MHz, CD_3CN): δ = 8.38 (dd, *J* = 1.6, 0.8 Hz, 1H), 8.05 (dd, *J* = 9.1, 1.6 Hz, 1H), 7.99 (dd, *J* = 9.2, 0.8 Hz, 1H), 4.49 (s, 3H), 4.46 (s, 3H) ppm.

^{13}C{^{1}H} NMR (126 MHz, CD_3CN): δ = 149.10 (d, $^1J_{CF}$ = 250.0 Hz, *o*-C_6F_5), 139.29 (d, $^1J_{CF}$ = 244.1 Hz, *p*-C_6F_5), 137.37 (s, Ar*C*Br), 137.35 (d, $^1J_{CF}$ = 243.4 Hz, *m*-C_6F_5), 137.07 (s, Ar*C*N), 135.78 (s, Ar*C*H), 135.65 (s, Ar*C*N), 125.89 (s, *ipso*-C_6F_5), 117.56 (s, Ar*C*H), 116.08 (s, Ar*C*H), 39.10 (s, N*C*H_3), 39.01 (s, N*C*H_3) ppm.

^{11}B{^{1}H} NMR (160 MHz, CD_3CN): δ = –16.69 ppm.

^{19}F NMR (470 MHz, CD_3CN): δ = –133.77 (s, 8F), –163.93 (t, J = 19.7 Hz, 4F), –168.36 (t, J = 19.0 Hz, 8F) ppm.

^{19}F{^{1}H} NMR (470 MHz, CD_3CN): δ = –133.77 (s, 8F), –163.93 (t, J = 19.7 Hz, 4F), –168.36 (t, J = 19.0 Hz, 8F) ppm.

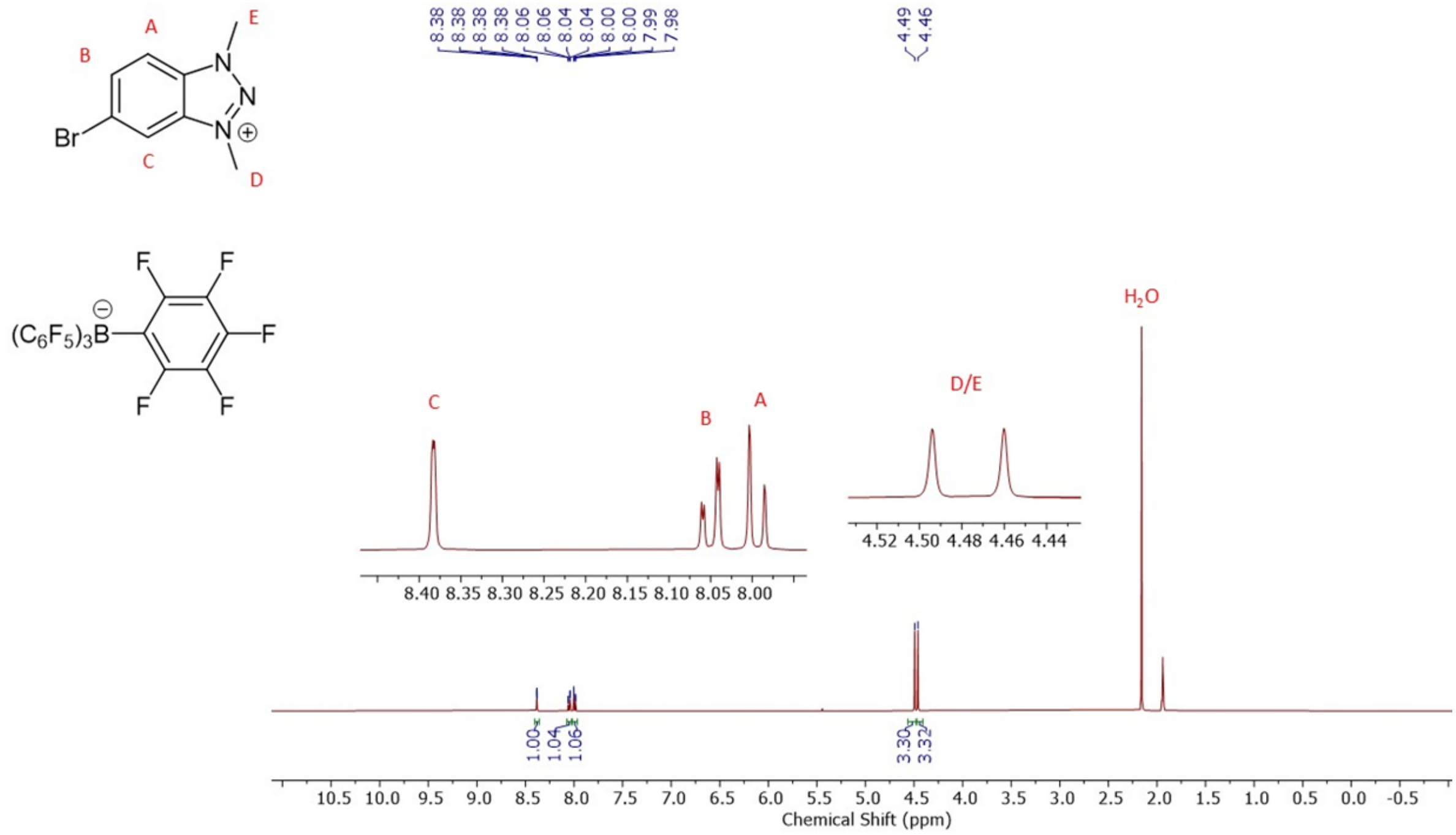

Figure S22. ^{1}H NMR spectrum (CD_3CN, 500 MHz) of [**3**][$BArF_{20}$] at room temperature.

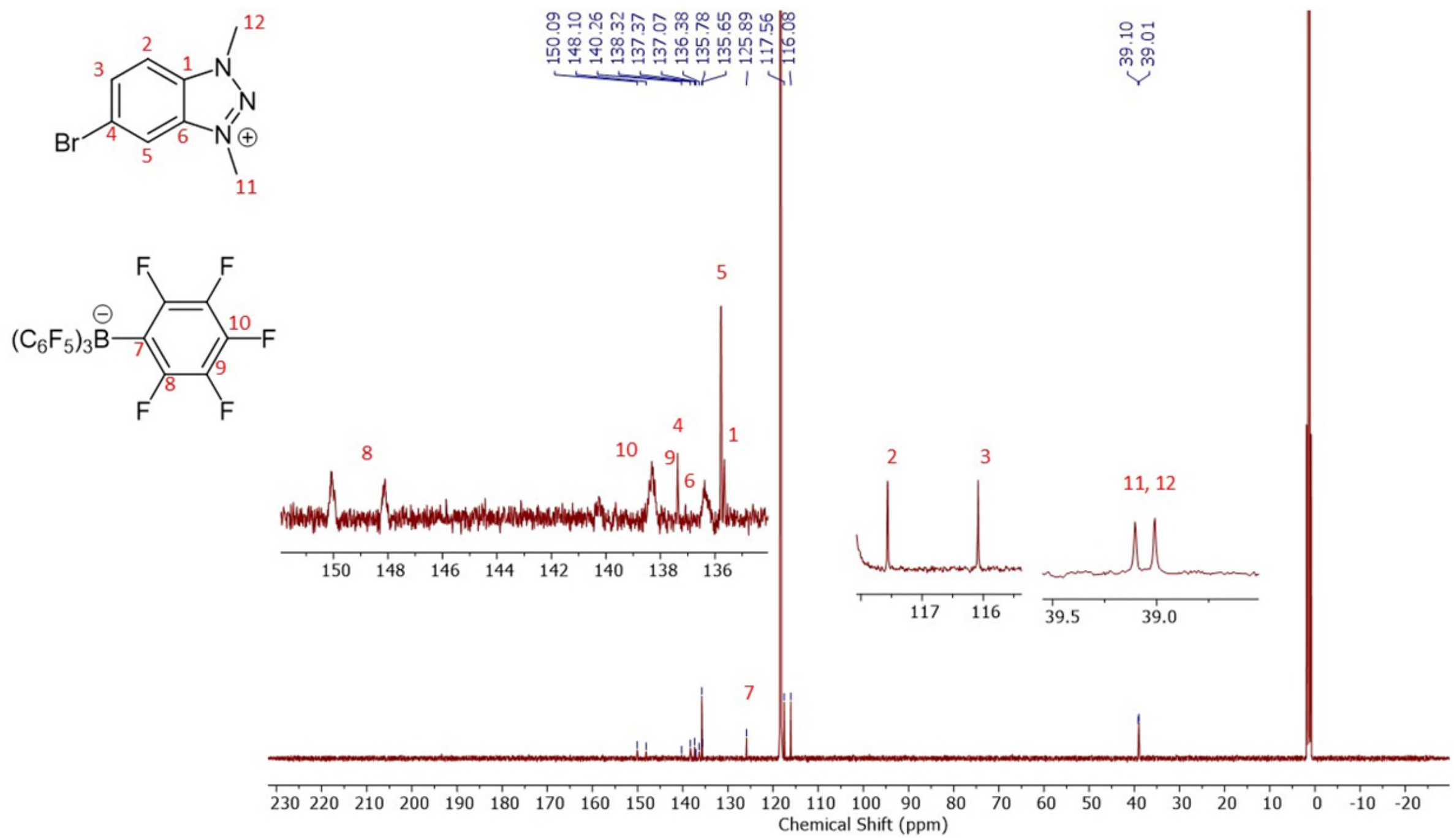

Figure S23. $^{13}C\{^1H\}$ NMR spectrum (CD_3CN, 126 MHz) of [**3**][$BArF_{20}$] at room temperature.

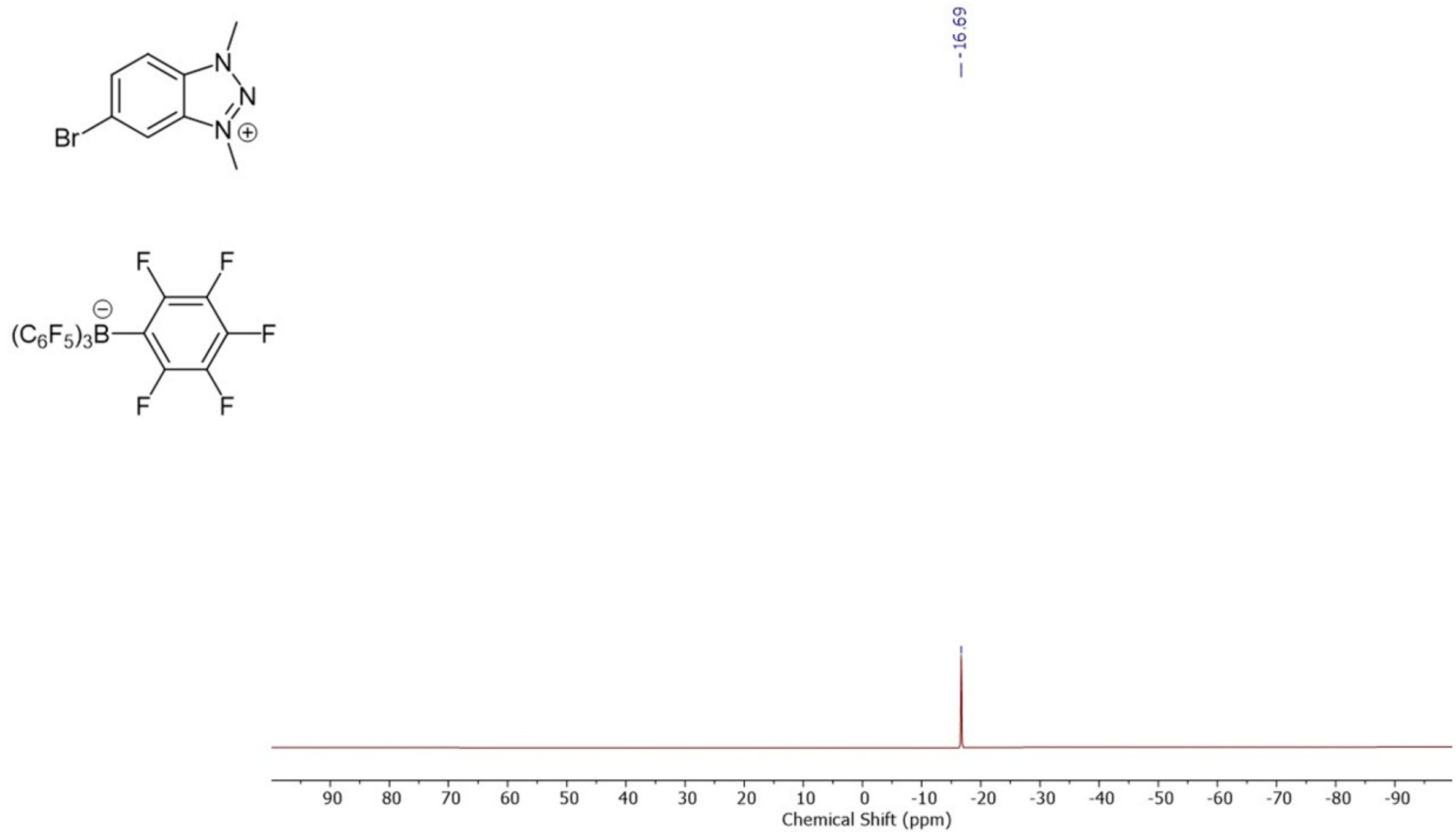

Figure S24. $^{11}B\{^{1}H\}$ NMR spectrum (CD_3CN, 160 MHz) of [**3**][$BArF_{20}$] at room temperature.

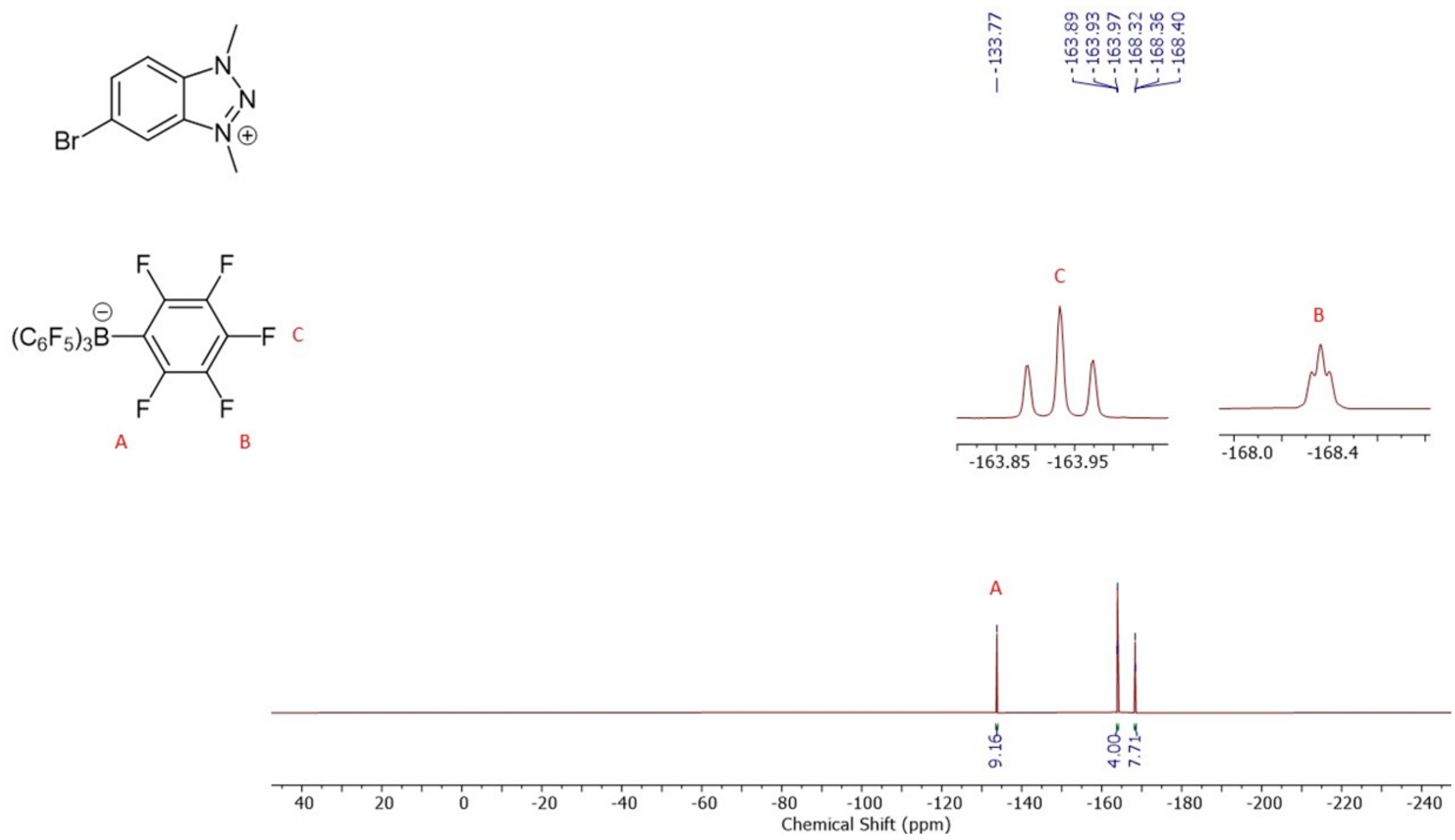

Figure S25. $^{19}F\{^{1}H\}$ NMR spectrum (CD_3CN, 470 MHz) of [**3**][$BArF_{20}$] at room temperature.

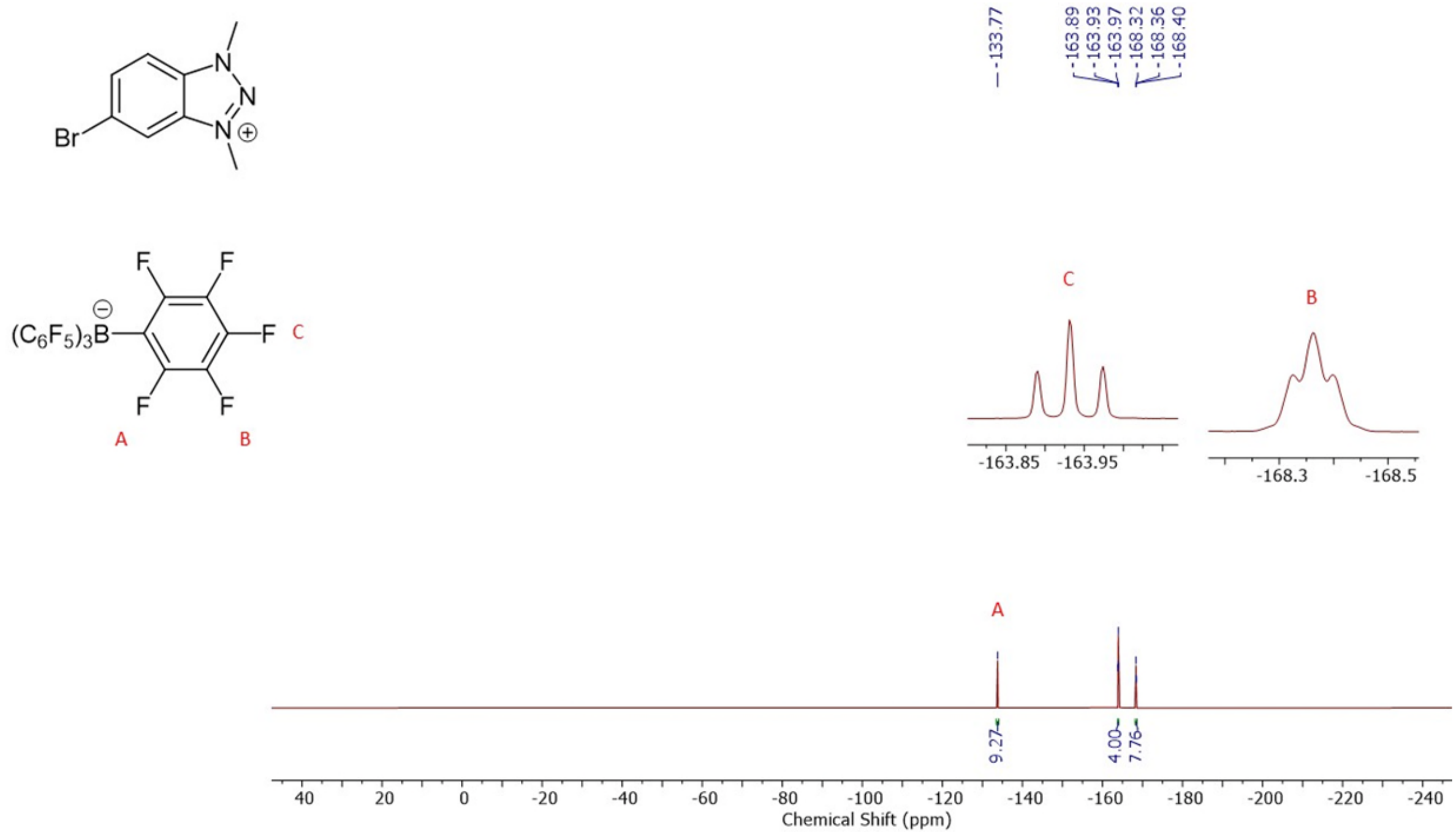

Figure S26. ^{19}F NMR spectrum (CD_3CN, 470 MHz) of [**3**][$BArF_{20}$] at room temperature.

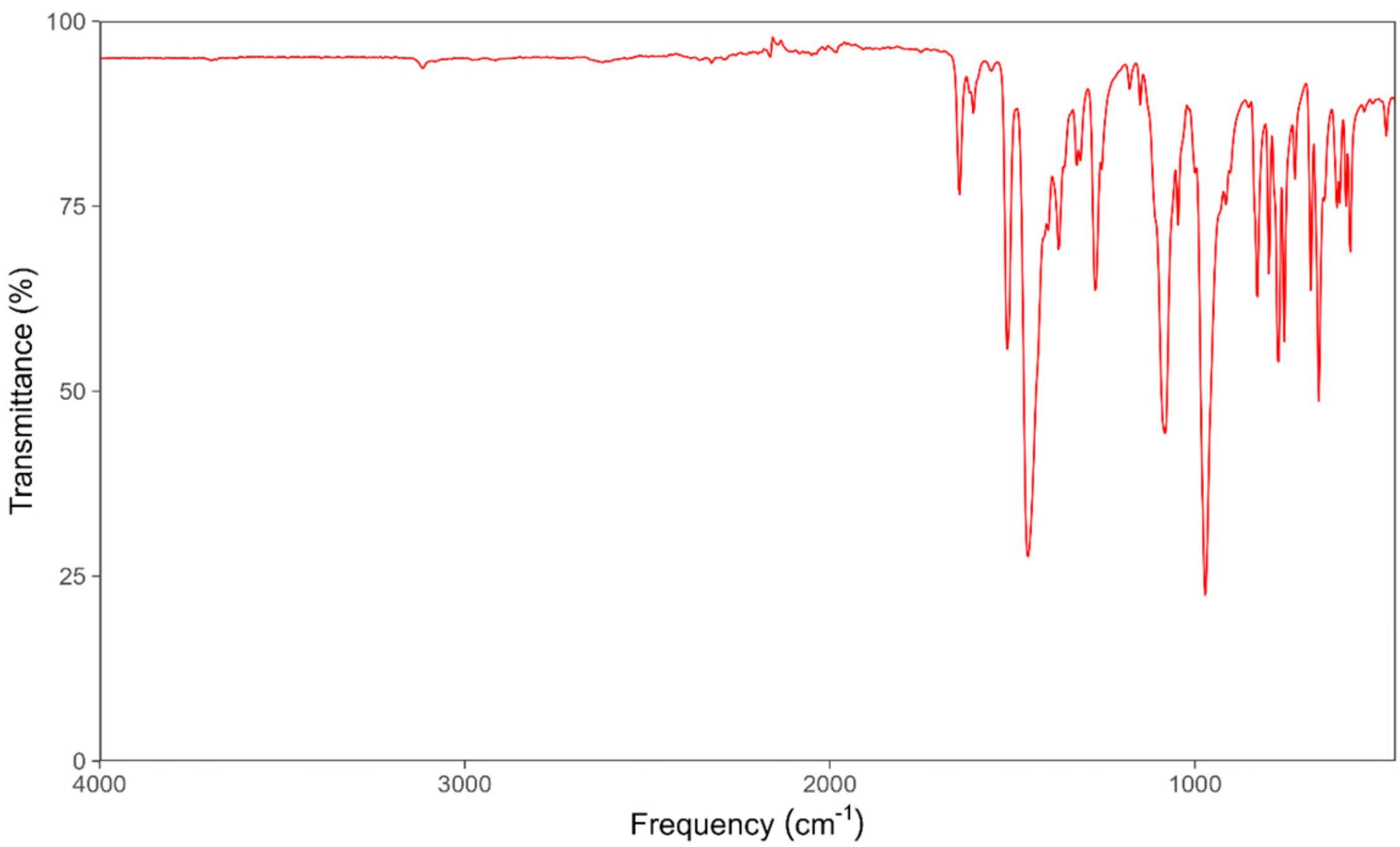

Figure S27. Experimental IR spectrum of [**3**][$BArF_{20}$].

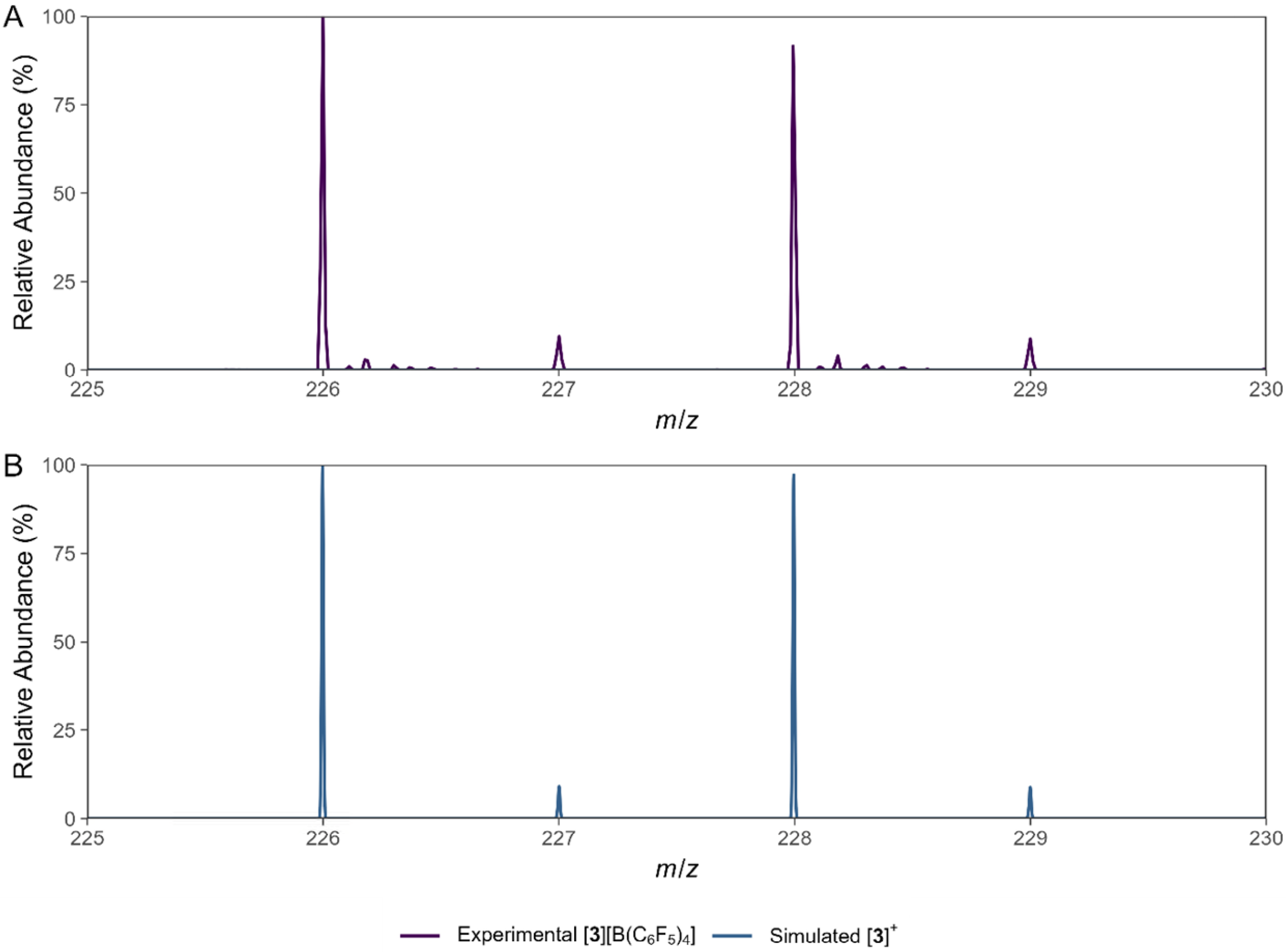

Figure S28. (A) Experimental HR-ESI-MS spectrum for [**3**][$BArF_{20}$]. (B) Simulated HR-ESI-MS spectrum for [**3**]$^+$.

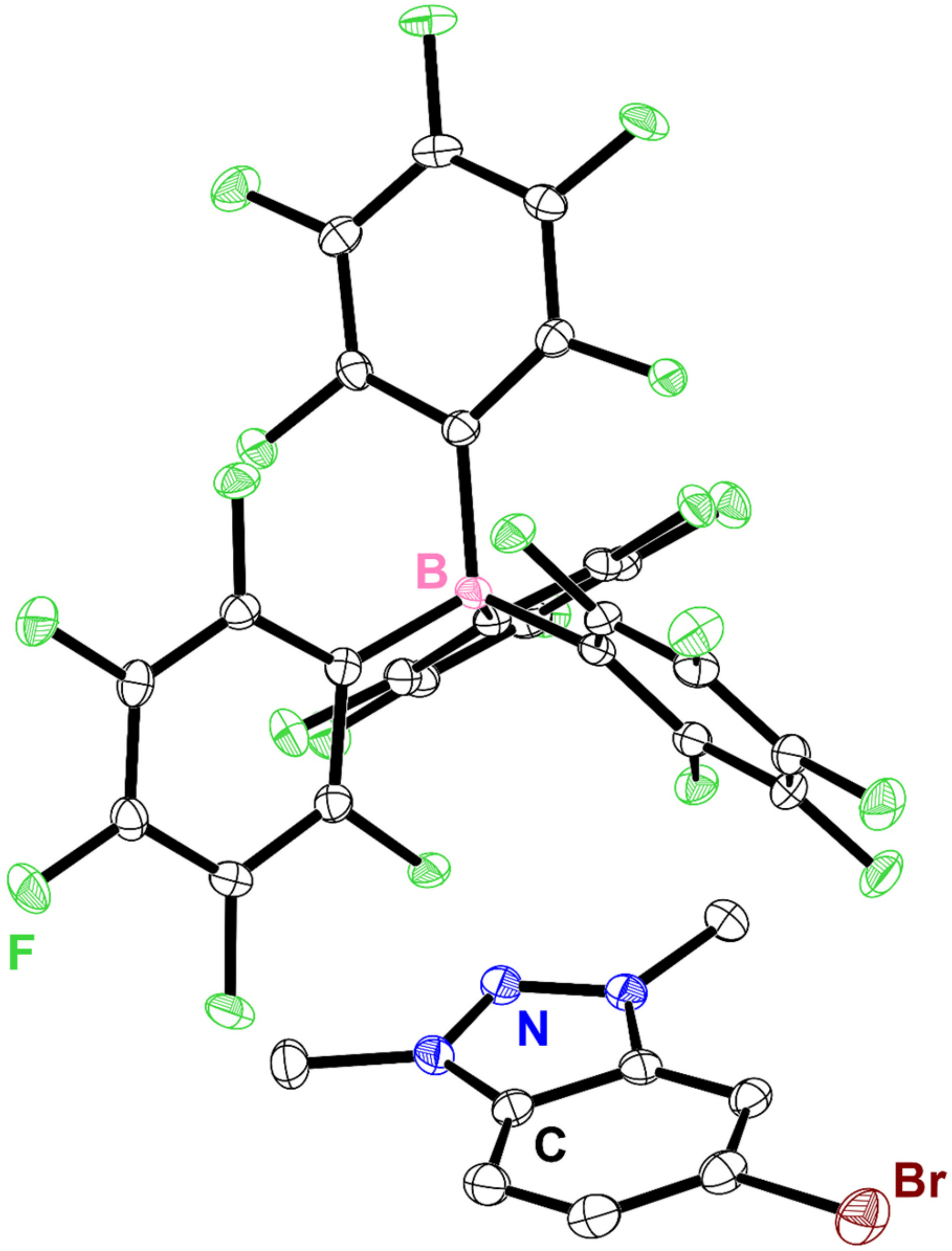

Figure S29. Thermal ellipsoid plot (50% probability) of [**3**][BArF$_{20}$]. H atoms are omitted for clarity. Colour code: N blue, B pink, C black, F light green, Br maroon.

2.4.2 Cyclic Voltammetry

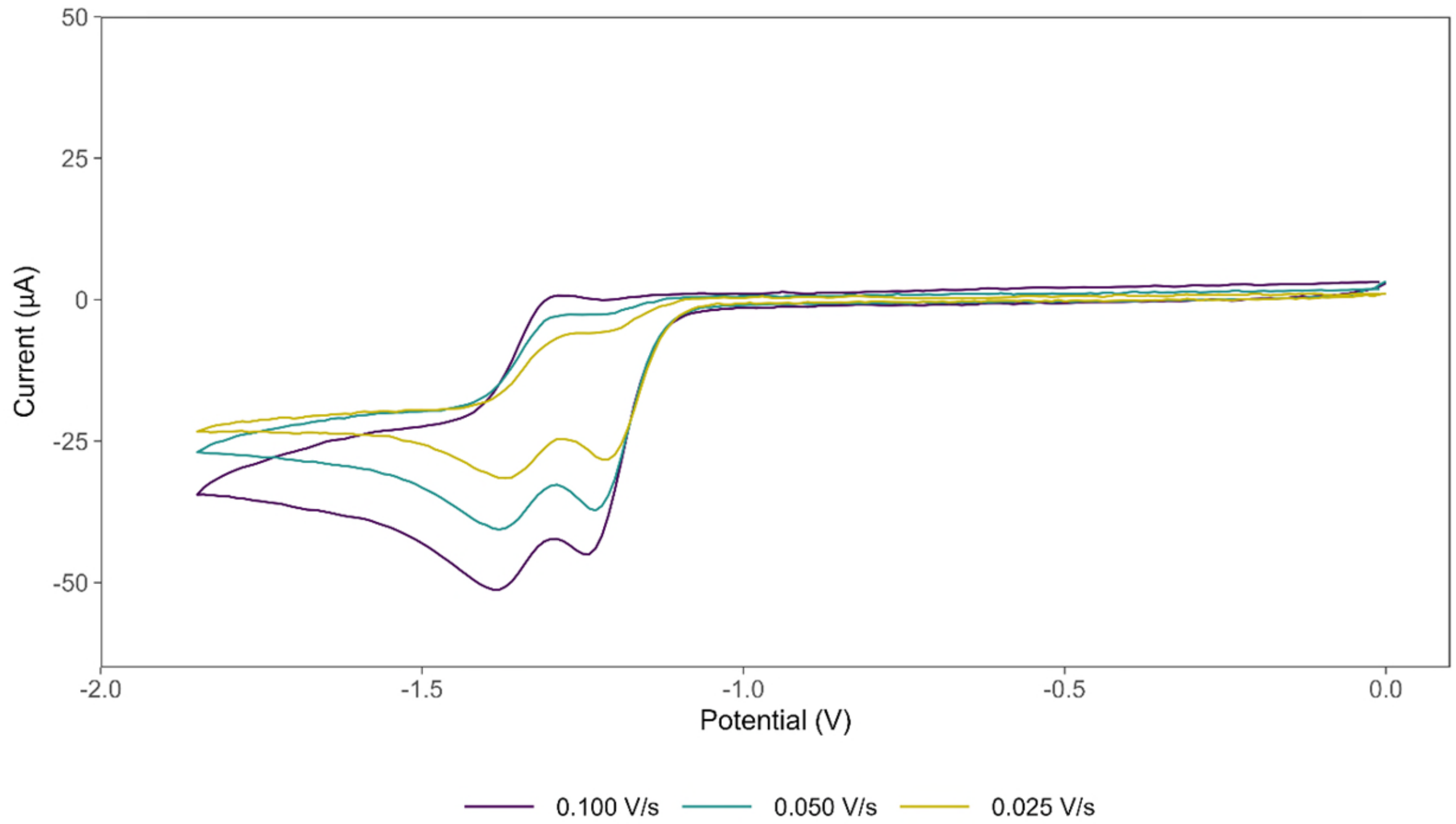

Figure S30. Cyclic voltammetry of [**3**][$BArF_{20}$]. Experimental details: Analyte; 2 mM. Electrolyte; 100 equiv. [Bu_4N][PF_6]. Solvent; acetonitrile. Working electrode; glassy carbon. Counter electrode; platinum. Reference electrode; leak-proof Ag/AgCl.

2.4.3 Spin Density Data

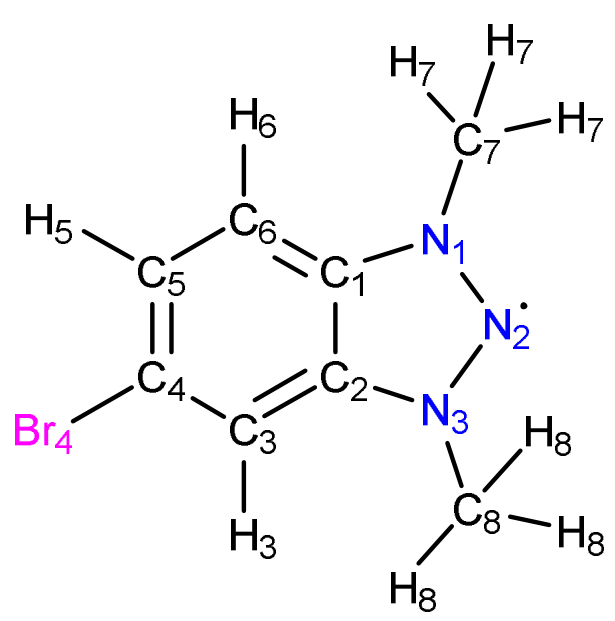

Table S3. Spin densities (B3LYP/def2-TZVP) of **3˙**.

Atom	Mulliken Spin Density
N1	0.108
N2	0.524
N3	0.106
C1	–0.052
C2	–0.049
C3	0.137
C4	0.051
C5	0.039
C6	0.141
C7	–0.007
C8	–0.007
Br4	0.003
H3	–0.006
H5	–0.002
H6	–0.006
3 × H7	0.004
3 × H8	0.004

2.5 4,6-Difluoro-1,3-dimethyl-1H-benzo[d][1,2,3]triazol-3-ium tetrakis(pentafluorophenyl)borate - [4][BArF$_{20}$]

2.5.1 Synthesis

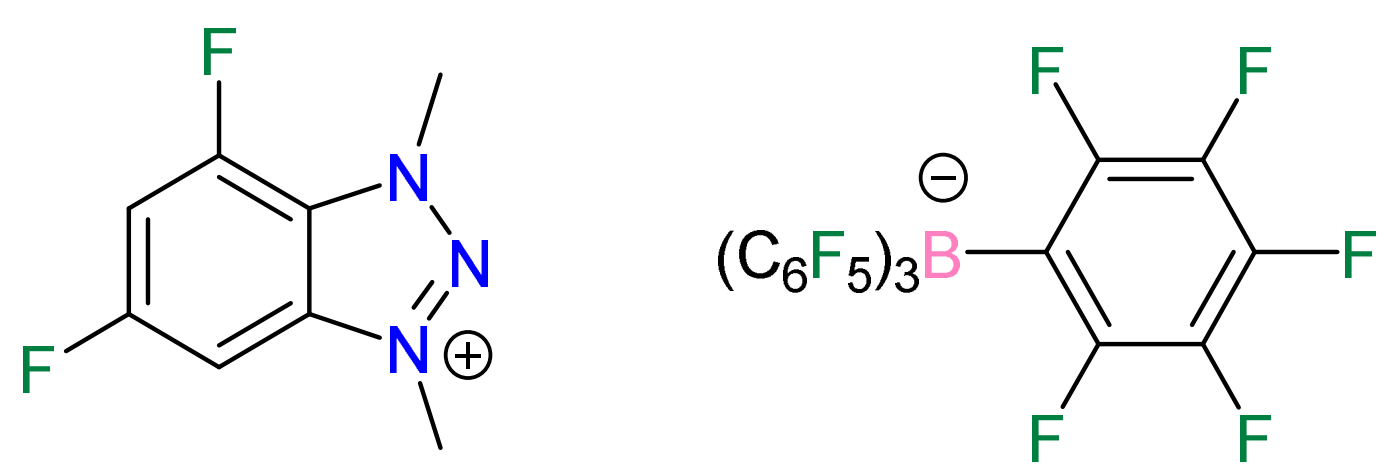

Compound [**4**][BArF$_{20}$] was prepared following the general procedure.

Yield: 0.153 g (2.4 %).

Elemental analysis, Found: C, 44.51; H, 0.96; N, 4.77%. **Calc.** for $C_{32}H_8BF_{22}N_3$: C, 44.52; H, 0.93; N, 4.87%.

HR-ESI-MS [$C_8H_8F_2N_3$]$^+$: 184.0681 (calc. 184.0681).

^{1}H NMR (500 MHz, CD_3CN): δ = 7.74–7.68 (m, 1H), 7.65–7.56 (m, 1H), 4.60 (s, 3H), 4.47 (s, 3H) ppm.

$^{13}C\{^1H\}$ NMR (126 MHz, CD_3CN): δ = 164.27 (dd, $^1J_{CF}$ = 255.8 Hz, $^3J_{CF}$ = 9.3 Hz, Ar*C*F), 149.67 (dd, $^1J_{CF}$ = 260.7 Hz, $^3J_{CF}$ = 14.9 Hz, Ar*C*F), 149.09 (d, $^1J_{CF}$ = 238.4 Hz, *o*-C_6F_5), 139.30 (d, $^1J_{CF}$ = 245.4 Hz, *p*-C_6F_5), 138.83 (d, $^3J_{CF}$ = 17.0 Hz, Ar*C*N), 137.36 (d, $^1J_{CF}$ = 243.1 Hz, *m*-C_6F_5), 125.83 (s, *ipso*-C_6F_5), 125.01 (d, $^2J_{CF}$ = 17.8 Hz, Ar*C*N), 108.76 (dd, $^2J_{CF}$ = 32.1 Hz, $^2J_{CF}$ = 20.1 Hz, Ar*C*H), 97.24 (dd, $^2J_{CF}$ = 29.1 Hz, $^4J_{CF}$ = 6.0 Hz, Ar*C*H), 41.41 (d, $^4J_{CF}$ = 3.5 Hz, N*C*H_3), 39.45 (s, N*C*H_3) ppm.

$^{11}B\{^1H\}$ NMR (160 MHz, CD_3CN): δ = –16.69 ppm.

^{19}F NMR (470 MHz, CD_3CN): δ = –101.81 (d, *J* = 8.4 Hz, 1F), –123.04 (t, *J* = 9.5 Hz, 1F), –133.77 (dt, *J* = 16.5, 7.8 Hz, 8F), –163.95 (t, *J* = 19.8 Hz, 4F), –168.38 (t, *J* = 18.8 Hz, 8F) ppm.

$^{19}F\{^1H\}$ NMR (470 MHz, CD_3CN): δ = –101.82 (d, *J* = 8.7 Hz, 1F), –123.04 (d, *J* = 7.8 Hz, 1F), –133.78 (s, 8F), –163.94 (t, *J* = 19.9 Hz, 4F), –168.39 (t, *J* = 18.8 Hz, 8F) ppm.

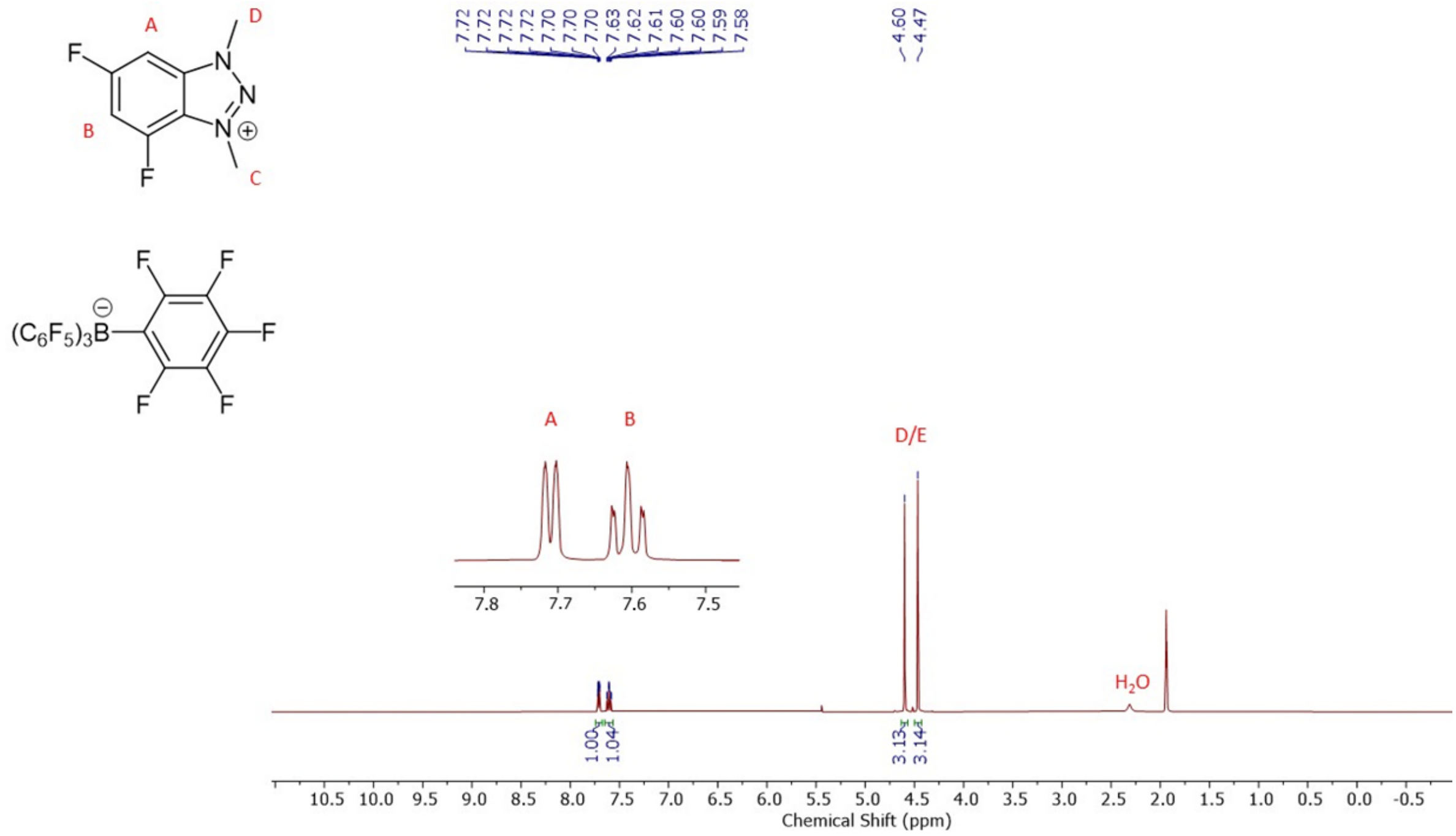

Figure S31. ^1H NMR spectrum (CD_3CN, 500 MHz) of [**4**][$BArF_{20}$] at room temperature.

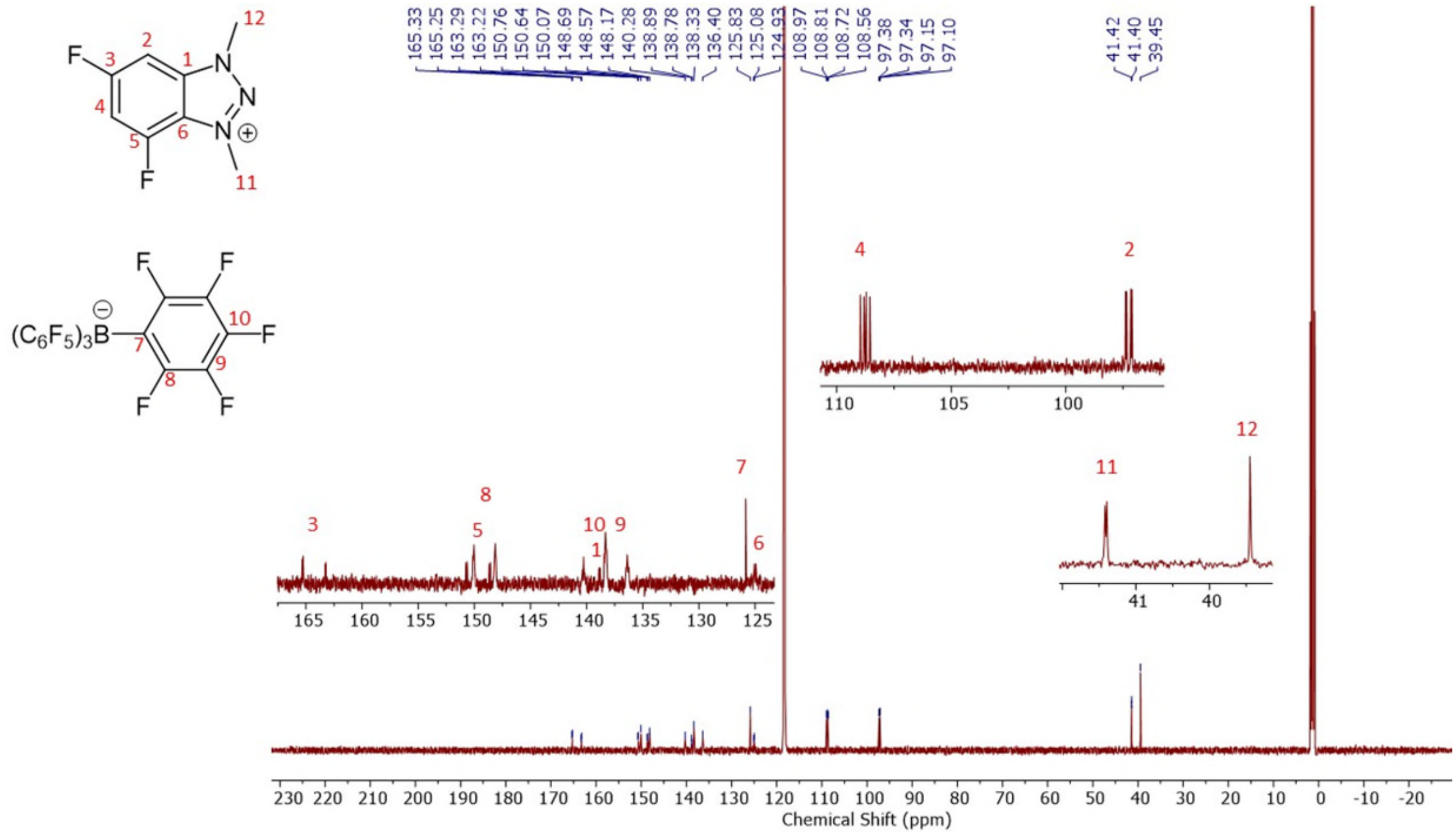

Figure S32. $^{13}C\{^{1}H\}$ NMR spectrum (CD_3CN, 126 MHz) of [**4**][BArF$_{20}$] at room temperature.

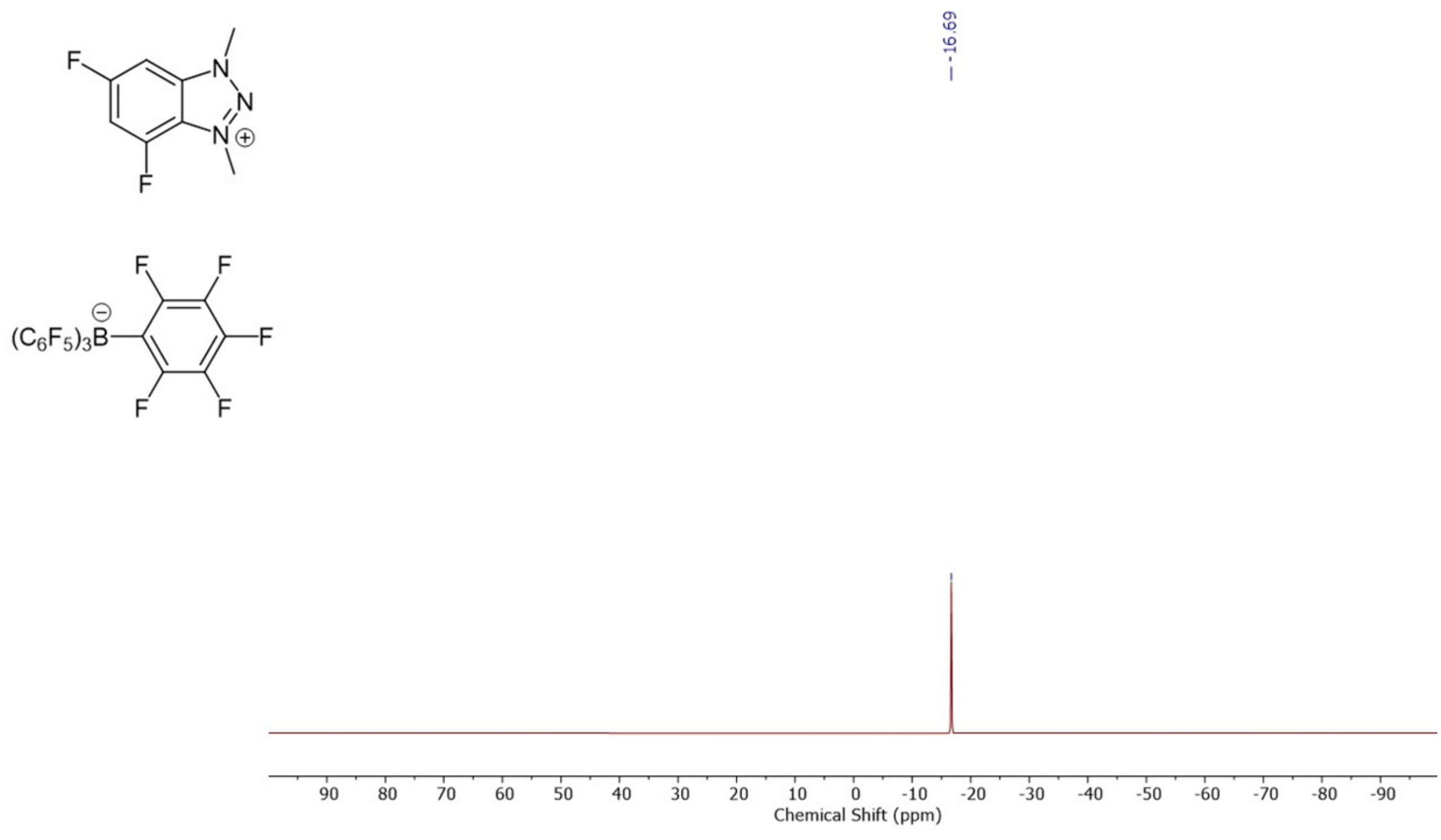

Figure S33. $^{11}B\{^{1}H\}$ NMR spectrum (CD_3CN, 160 MHz) of [**4**][BArF$_{20}$] at room temperature.

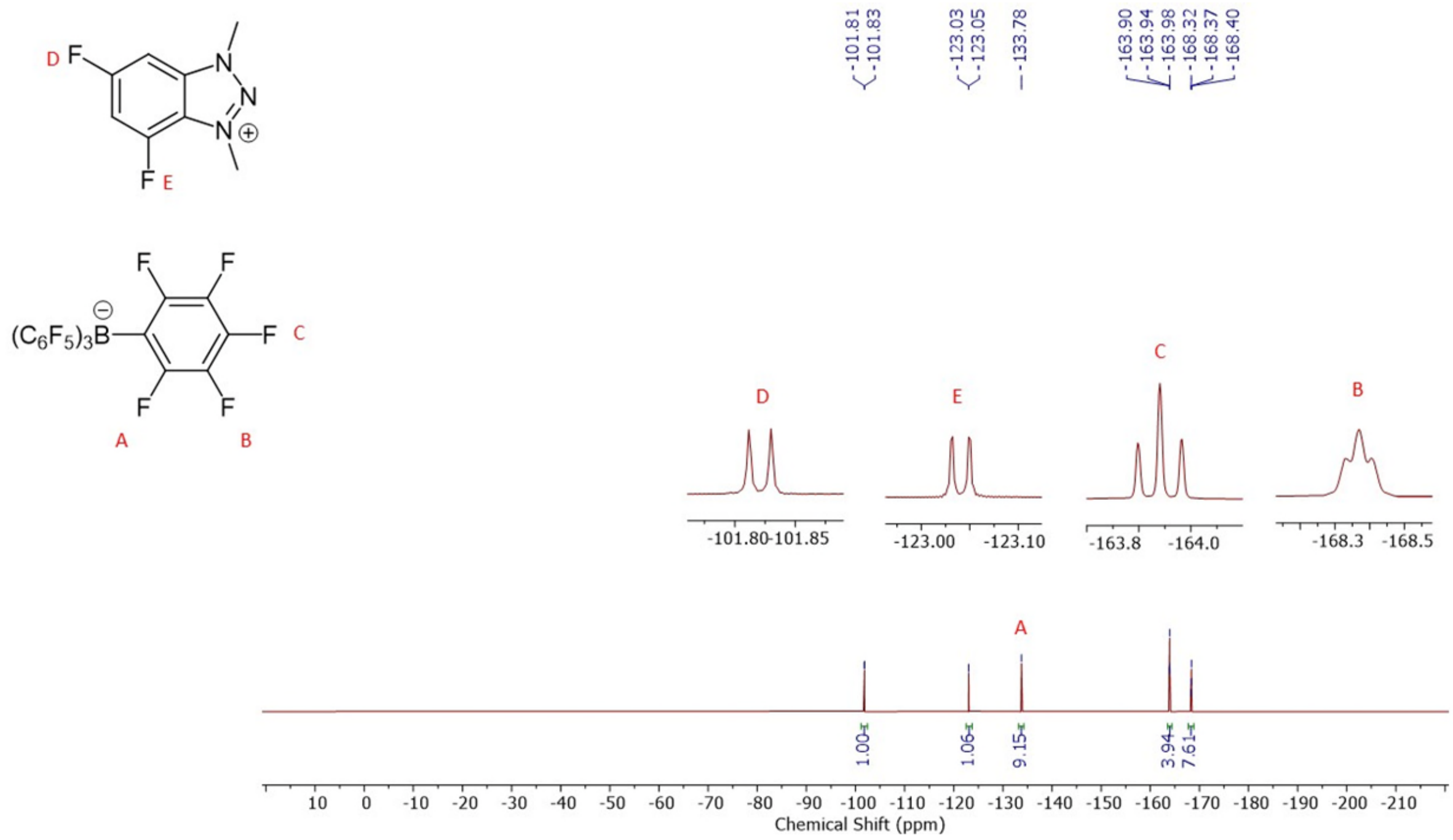

Figure S34. $^{19}F\{^{1}H\}$ NMR spectrum (CD_3CN, 470 MHz) of [**4**][$BArF_{20}$] at room temperature.

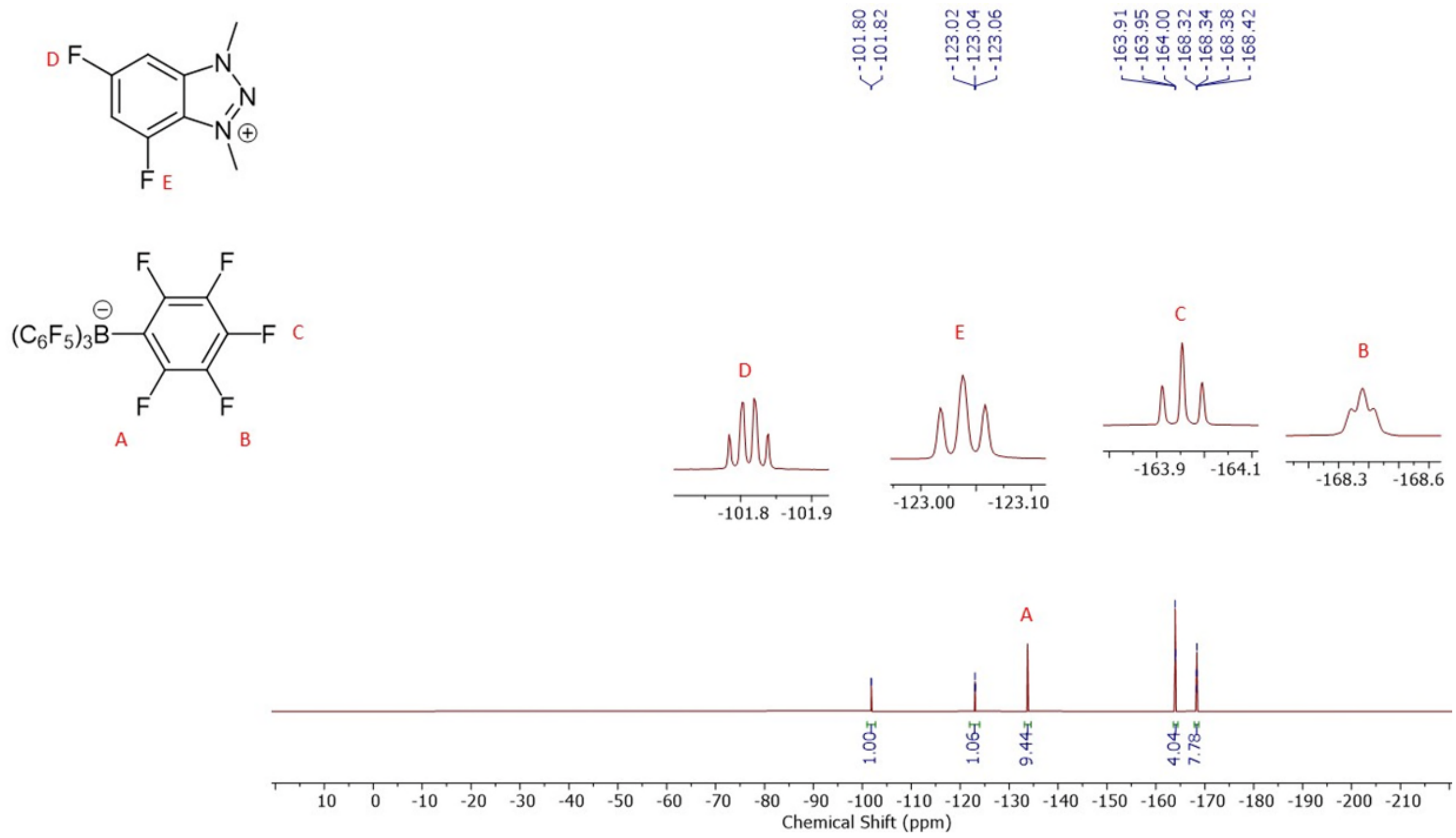

Figure S35. ^{19}F NMR spectrum (CD_3CN, 470 MHz) of [**4**][$BArF_{20}$] at room temperature.

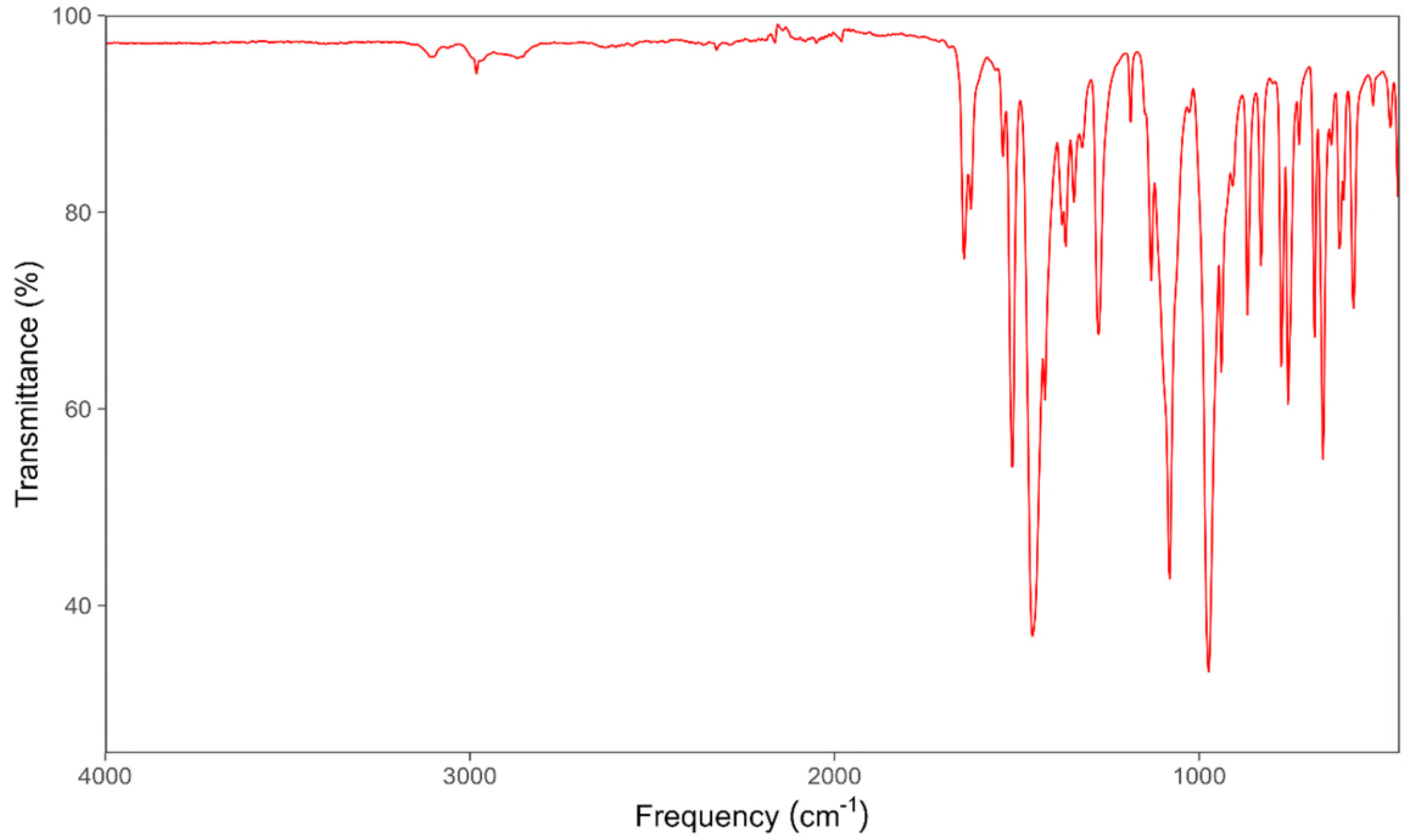

Figure S36. Experimental IR spectrum of [**4**][BArF20].

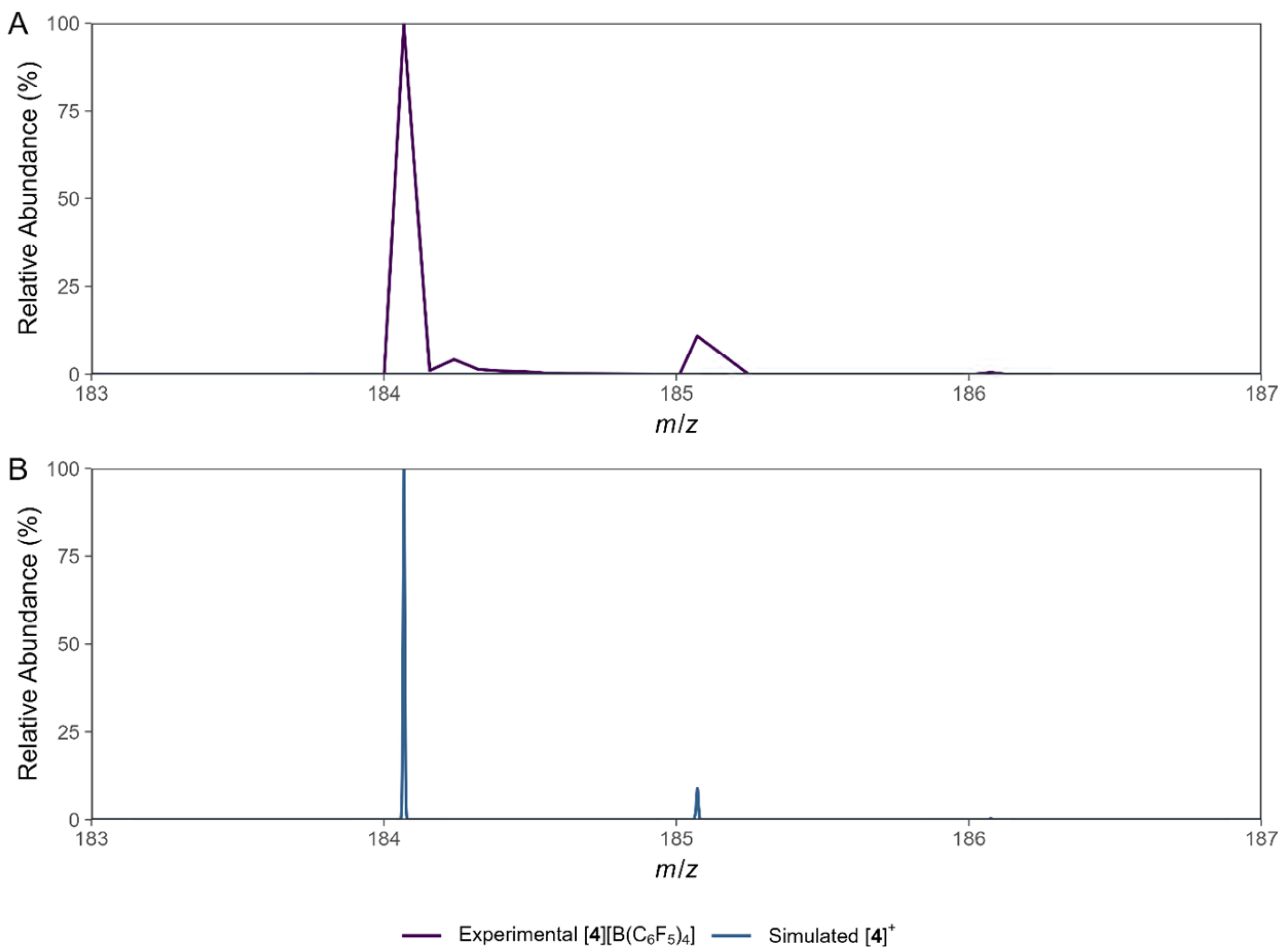

Figure S37. (A) Experimental HR-ESI-MS spectrum for [**4**][BArF20]. (B) Simulated HR-ESI-MS spectrum for [**4**]+.

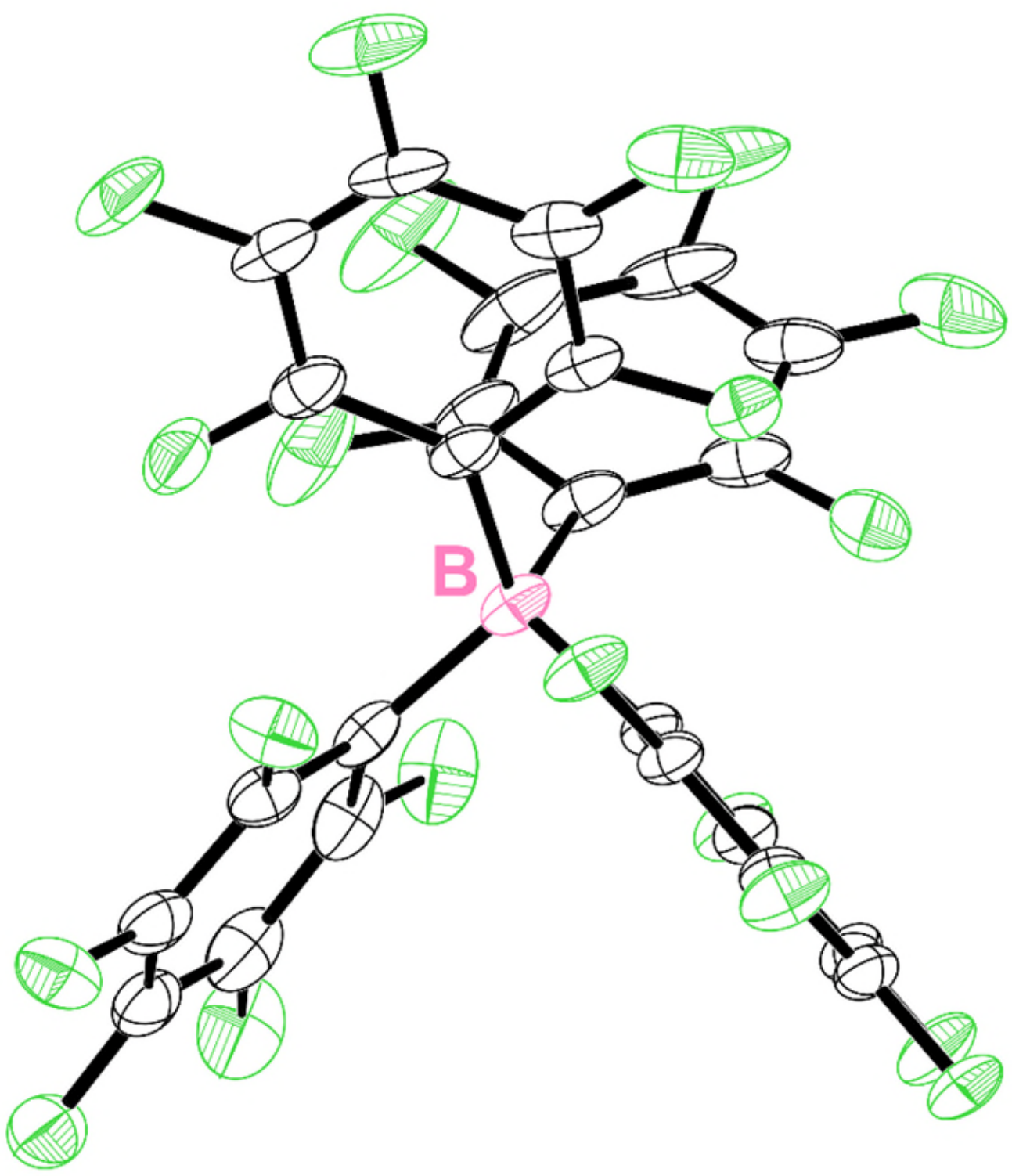

Figure S38. Thermal ellipsoid plot (50% probability) of [**4**][$BArF_{20}$]. H atoms and disordered components are omitted for clarity. Colour code: N blue, B pink, C black, F light green.

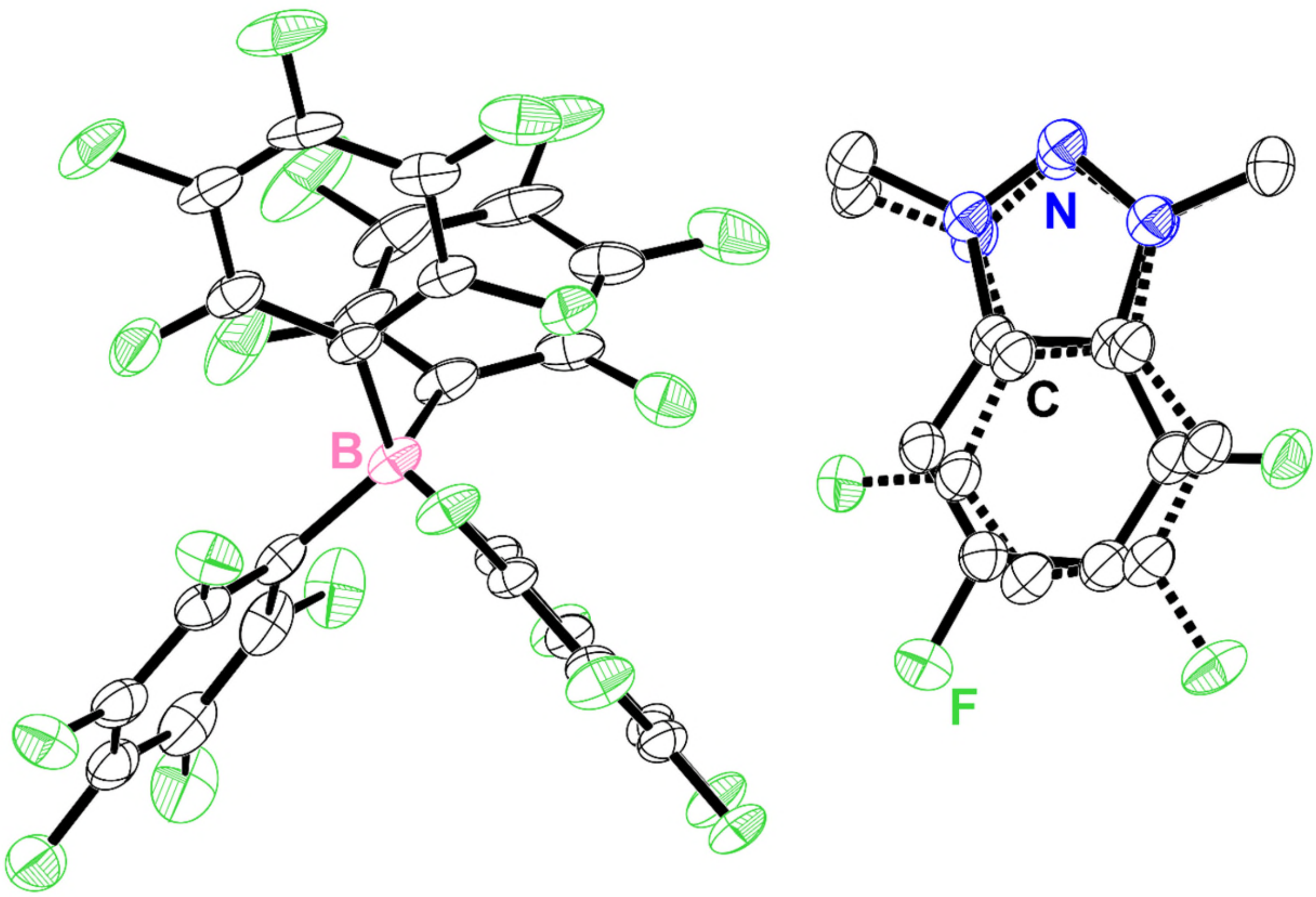

Figure S39. Thermal ellipsoid plot (50% probability) of [**4**][BArF$_{20}$]. H atoms omitted for clarity. Colour code: N blue, B pink, C black, F light green.

2.5.2 Cyclic Voltammetry

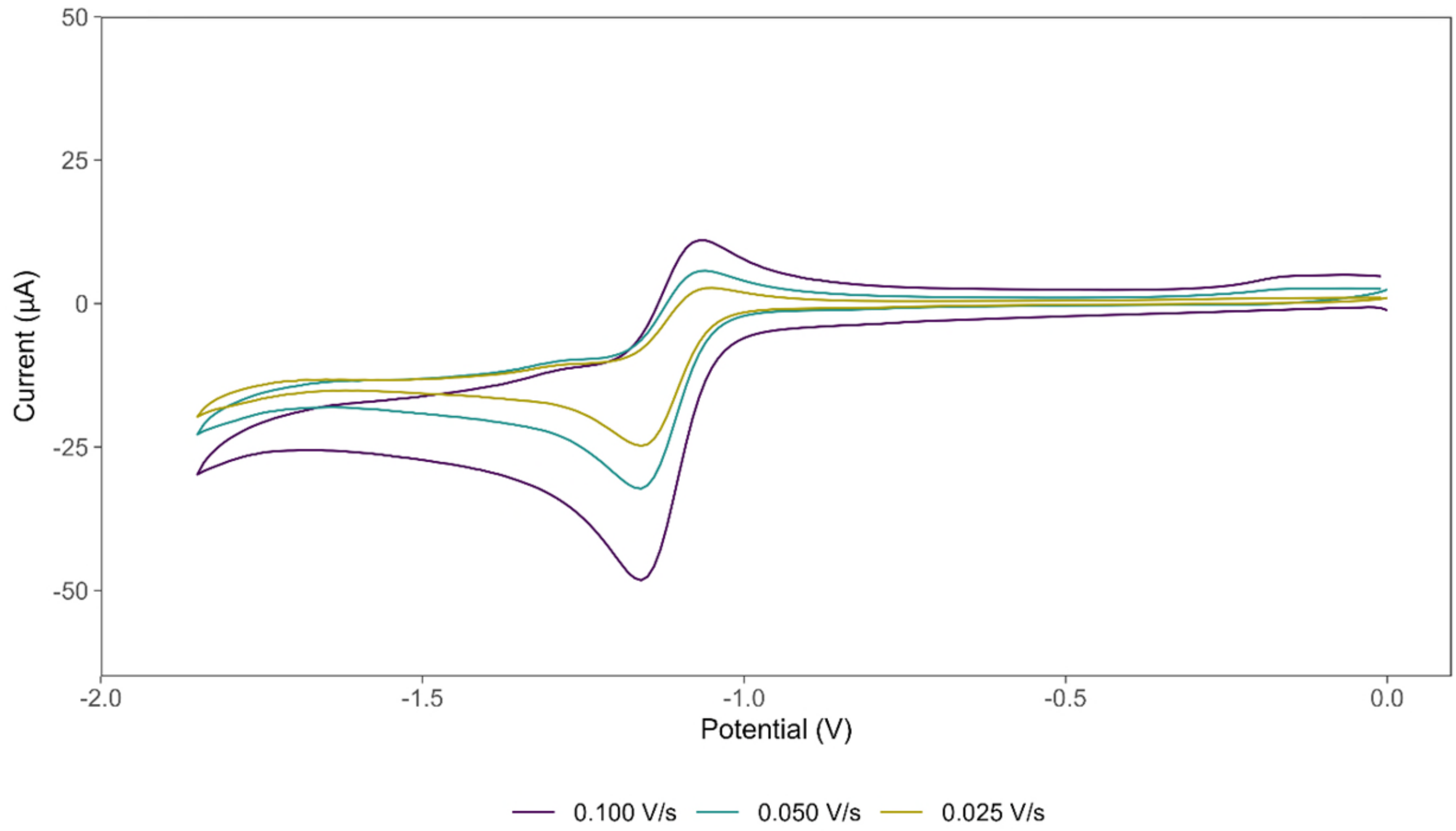

Figure S40: Cyclic voltammetry of [**4**][$BArF_{20}$]. Experimental details: Analyte; 2 mM. Electrolyte; 100 equiv. [Bu_4N][PF_6]. Solvent; acetonitrile. Working electrode; glassy carbon. Counter electrode; platinum. Reference electrode; leak-proof Ag/AgCl.

2.5.3 Spin Density

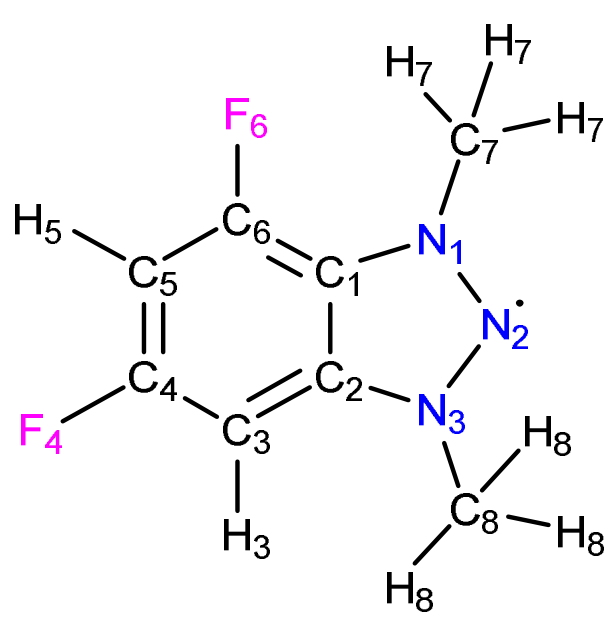

Table S4. Spin densities (B3LYP/def2-TZVP) of **4**$^{\bullet}$.

Atom	Mulliken Spin Density
N1	0.144
N2	0.541
N3	0.100
C1	–0.030
C2	–0.044
C3	0.080
C4	0.033
C5	0.047
C6	0.117
C7	–0.009
C8	–0.006
F4	0.002
F6	0.005
H3	–0.003
H5	–0.003
3 × H7	0.005
3 × H8	0.005

2.6 5,6-Difluoro-1,3-dimethyl-1H-benzo[d][1,2,3]triazol-3-ium tetrakis(pentafluorophenyl)borate - [5][BArF$_{20}$]•(DCM)$_{0.5}$

2.6.1 Synthesis

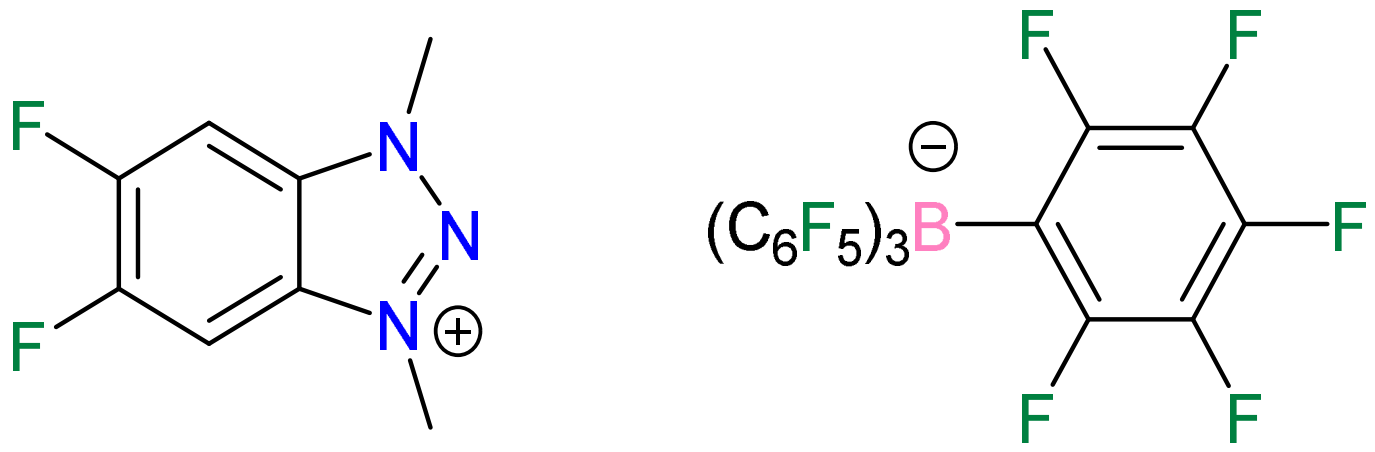

Compound [**5**][BArF$_{20}$]•(DCM)$_{0.5}$ was prepared following the general procedure.

Yield: 293 mg (4.7 %).

Elemental analysis, Found: C, 43.43; H, 0.91; N, 4.48%. **Calc.** for $C_{32.5}H_9BClF_{22}N_3$: C, 43.10; H, 1.00; N, 4.64%.

HR-ESI-MS: [$C_8H_8F_2N_3$]$^+$: 184.0694 (calc. 184.0681).

^{1}H NMR (500 MHz, CD_3CN): δ = 8.05 (t, *J* = 7.6 Hz, 2H), 5.44 (s, 1H), 4.46 (s, 6H) ppm.

^{13}C{^{1}H} NMR (126 MHz, CD_3CN): δ = 154.27 (dd, $^1J_{CF}$ = 260.0, $^3J_{CF}$ = 18.1 Hz, Ar*C*F), 149.09 (d, $^1J_{CF}$ = 242.4 Hz, *o*-C_6F_5), 139.31 (d, $^1J_{CF}$ = 241.1 Hz, *p*-C_6F_5), 137.32 (d, $^1J_{CF}$ = 240.1 Hz, *m*-C_6F_5), 133.07 (t, $^3J_{CF}$ = 6.5 Hz, Ar*C*N), 124.96 (br s, *ipso*-C_6F_5), 103.74–101.48 (m, Ar*C*H), 55.34 (DCM), 39.29 (s, N*C*H_3) ppm.

^{11}B{^{1}H} NMR (160 MHz, CD_3CN): δ = –16.69 ppm.

^{19}F NMR (470 MHz, CD_3CN): δ = –126.56 (t, *J* = 7.4 Hz, 2F), –133.77 (s, 8F), –163.95 (t, *J* = 19.6 Hz, 4F), –168.37 (t, *J* = 18.0 Hz, 8F) ppm.

^{19}F{^{1}H} NMR (470 MHz, CD_3CN): δ = –126.56 (s, 2F), –133.77 (s, 8F), –163.95 (t, *J* = 19.6 Hz, 4F), –168.37 (t, *J* = 18.0 Hz, 8F) ppm.

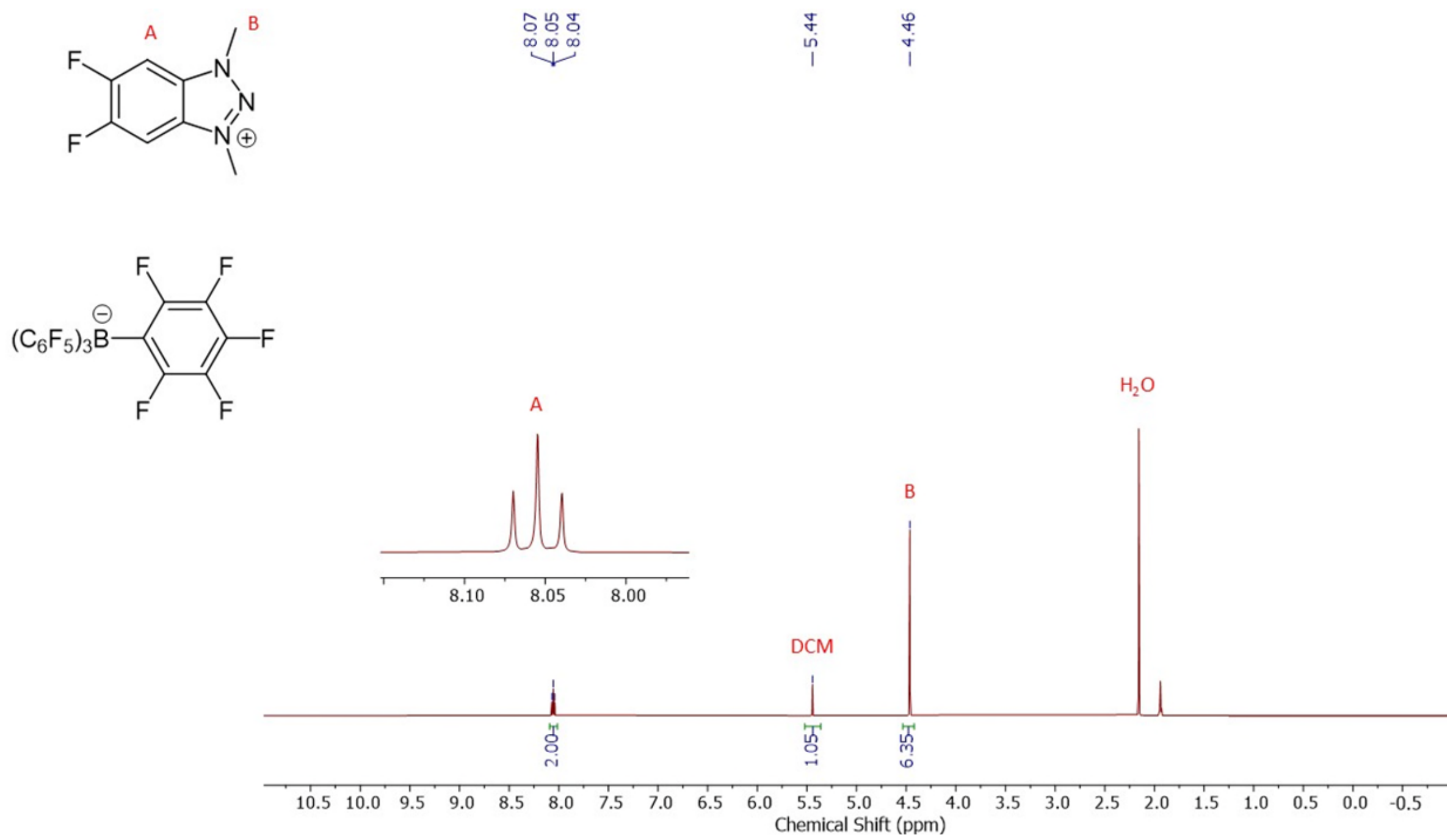

Figure S41. ^{1}H NMR spectrum (CD_3CN, 500 MHz) of [**5**][BArF$_{20}$]•(DCM)$_{0.5}$ at room temperature.

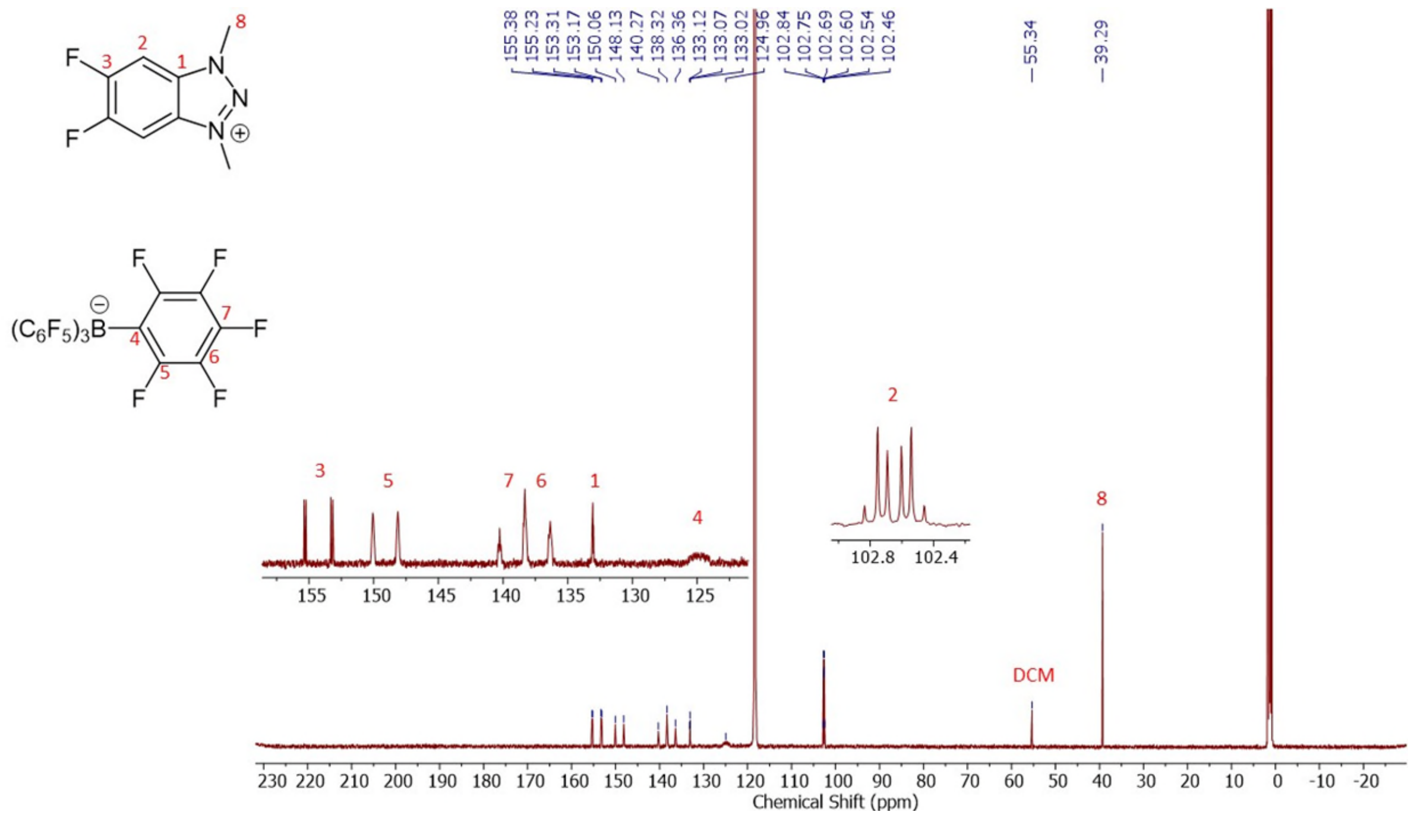

Figure S42. $^{13}C\{^{1}H\}$ NMR spectrum (CD_3CN, 126 MHz) of [**5**][BArF$_{20}$]•(DCM)$_{0.5}$ at room temperature.

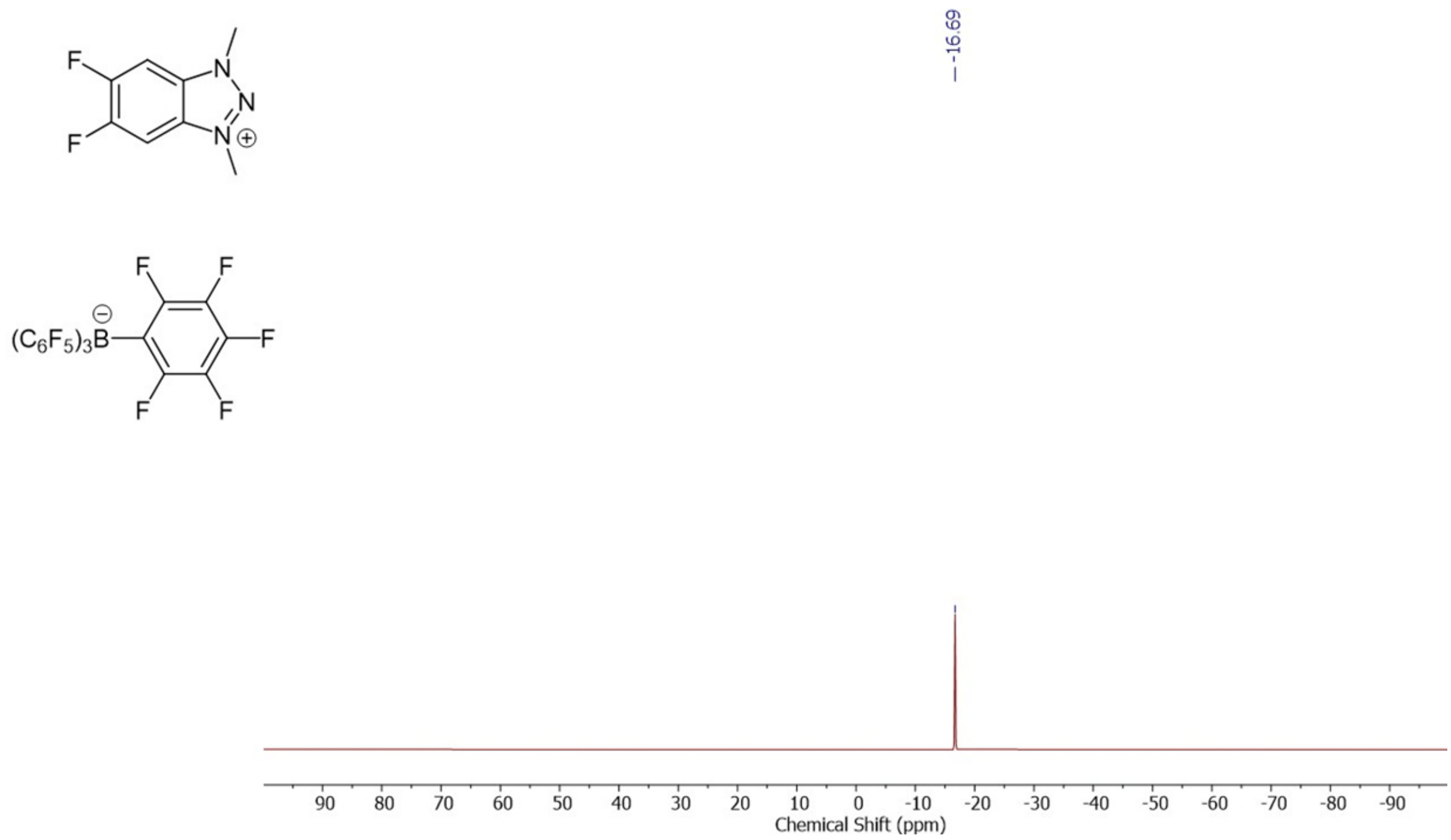

Figure S43. $^{11}B\{^1H\}$ NMR spectrum (CD_3CN, 160 MHz) of [**5**][$BArF_{20}$]•$(DCM)_{0.5}$ at room temperature.

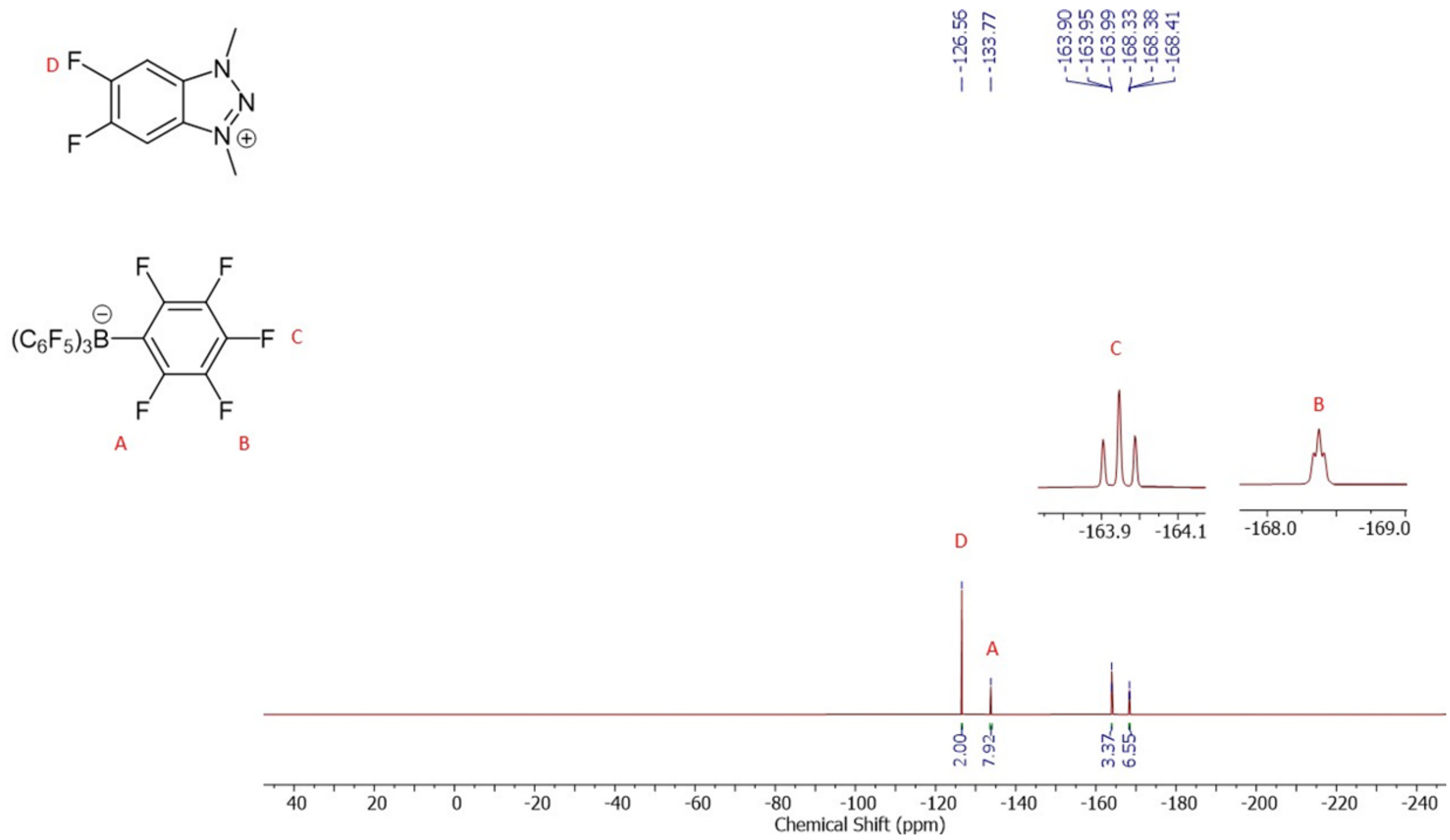

Figure S44. $^{19}F\{^1H\}$ NMR spectrum (CD_3CN, 470 MHz) of [**5**][$BArF_{20}$]•$(DCM)_{0.5}$ at room temperature.

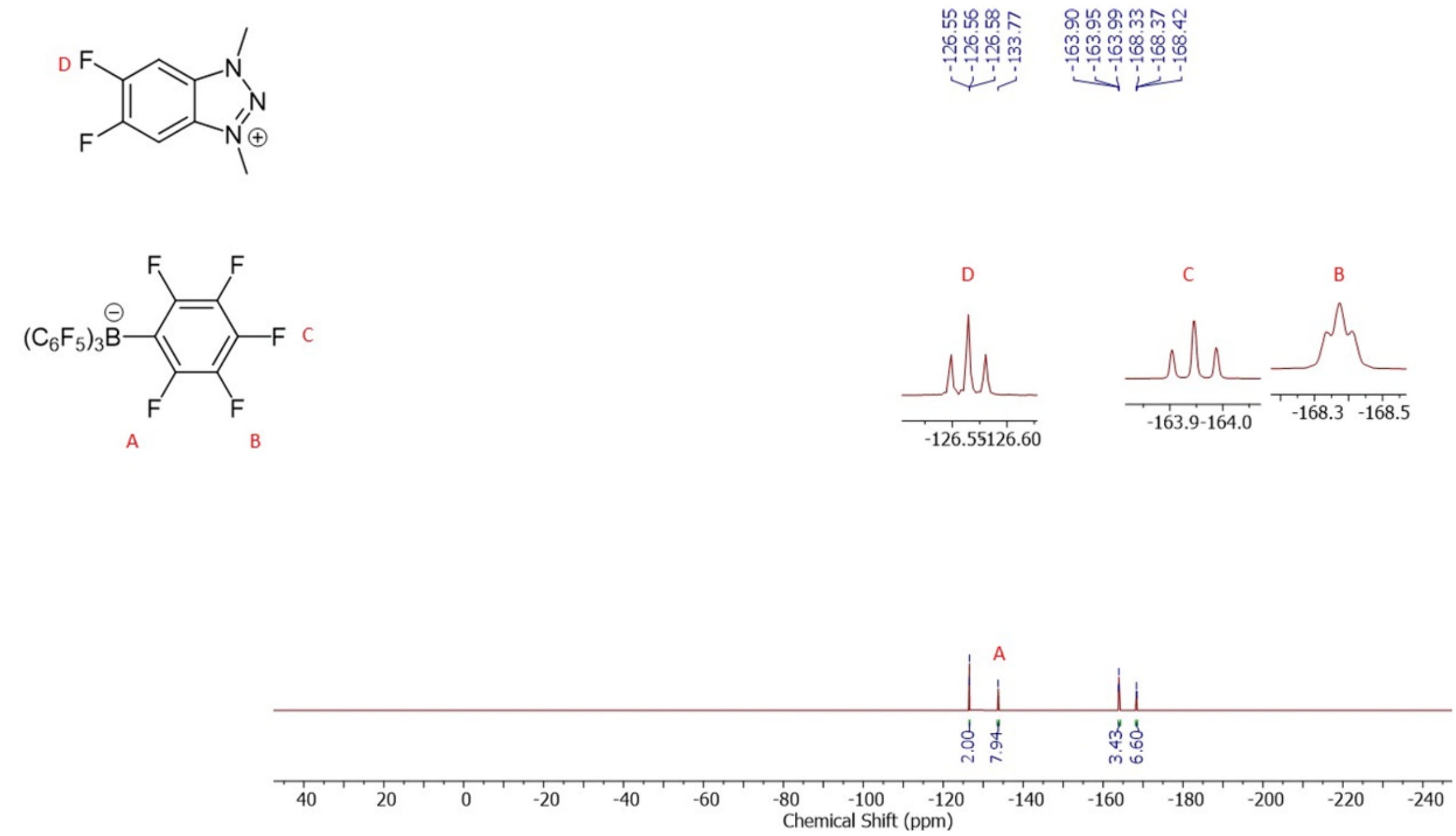

Figure S45. ^{19}F NMR spectrum (CD_3CN, 470 MHz) of [**5**][$BArF_{20}$]•$(DCM)_{0.5}$ at room temperature.

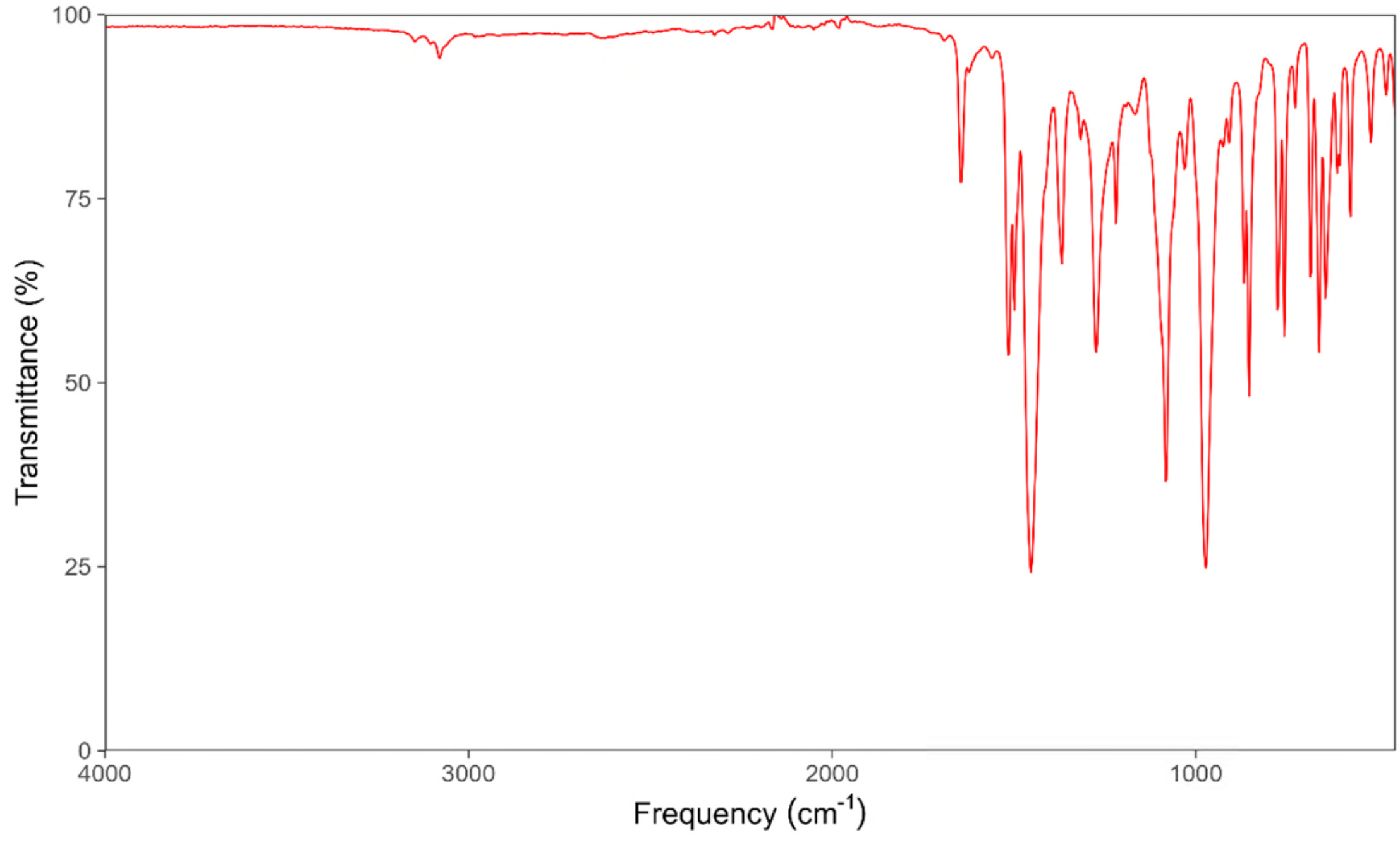

Figure S46. Experimental IR spectrum of [**5**][$BArF_{20}$]•$(DCM)_{0.5}$.

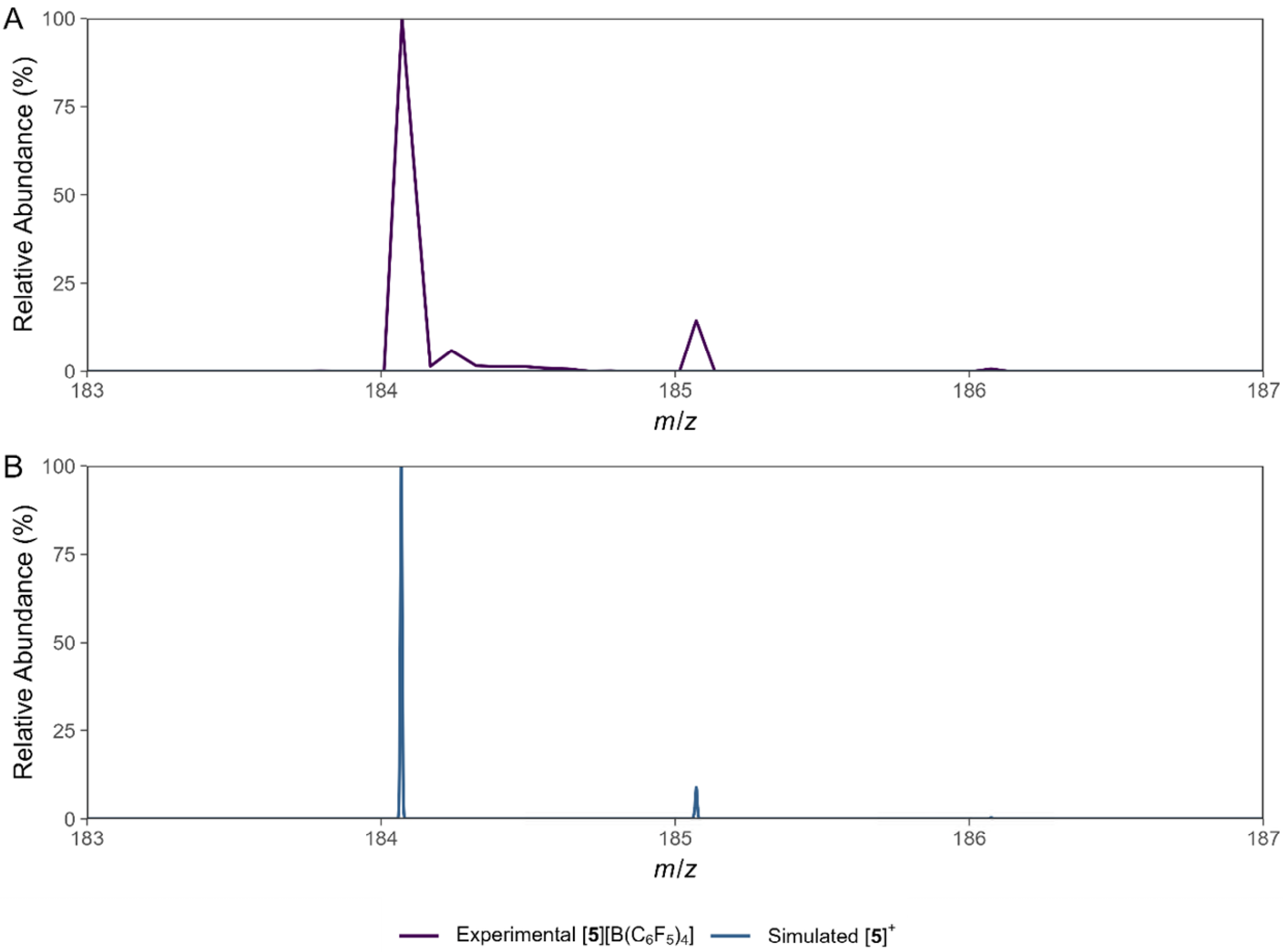

Figure S47. (A) Experimental HR-ESI-MS spectrum for [**5**][BArF$_{20}$]•(DCM)$_{0.5}$. (B) Simulated HR-ESI-MS spectrum for [**5**]$^{+}$.

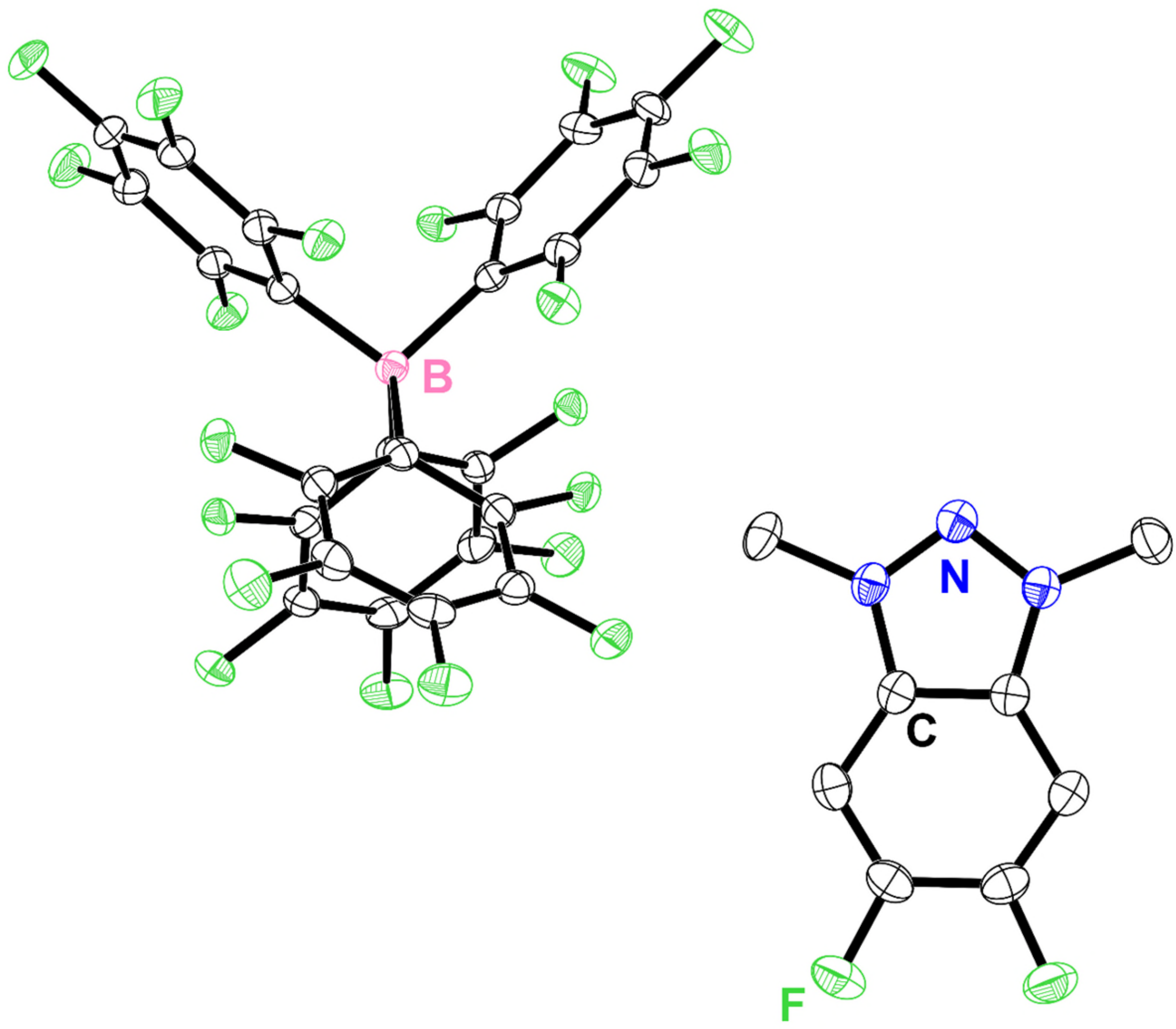

Figure S48. Thermal ellipsoid plot (50% probability) of [**5**][BArF$_{20}$]. H atoms, disordered components, and solvent molecules are omitted for clarity. Only one molecular unit from the asymmetric unit is displayed. Colour code: N blue, B pink, C black, F light green.

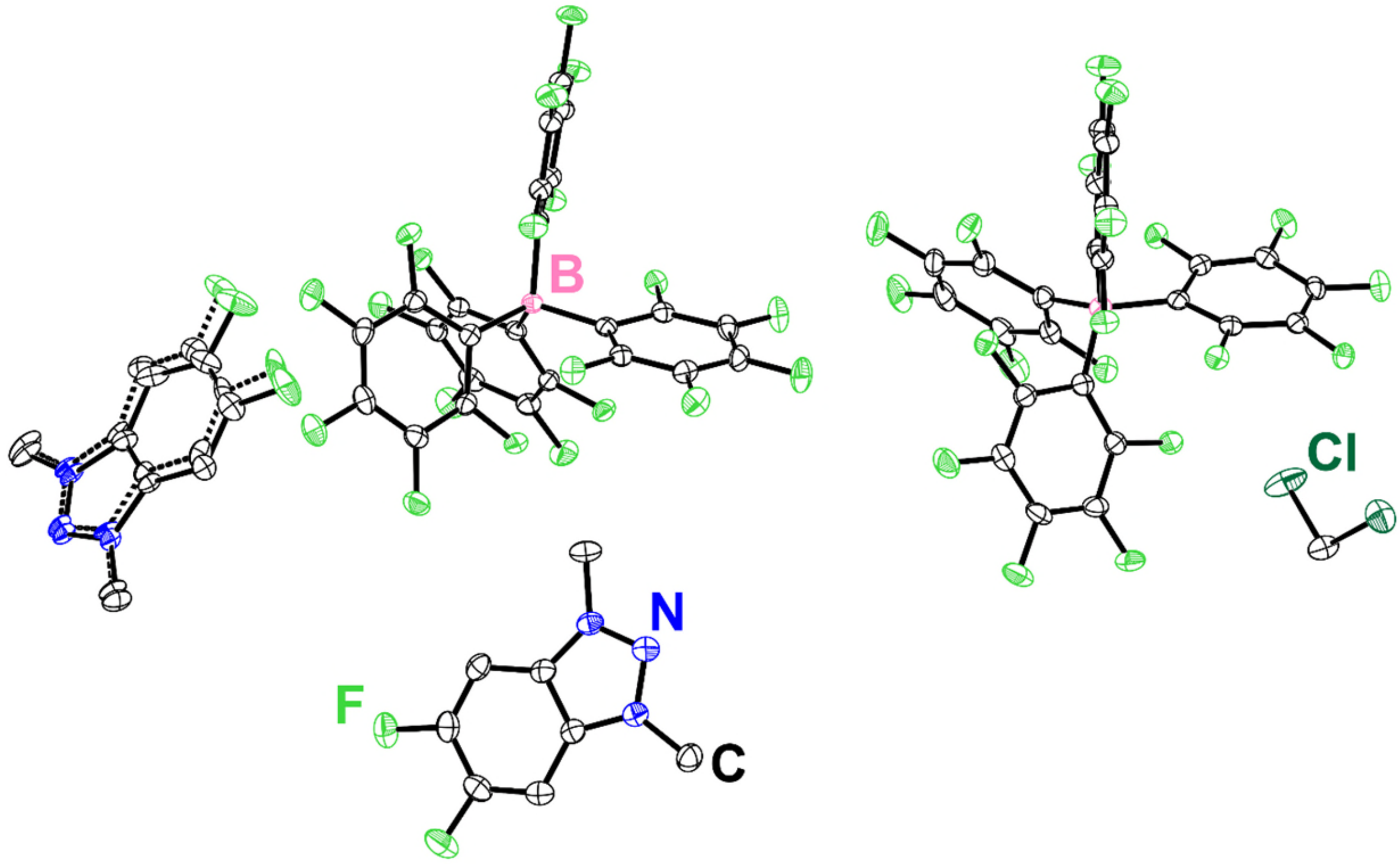

Figure S49. Thermal ellipsoid plot (50% probability) of [**5**][BArF$_{20}$]•(DCM)$_{0.5}$. H atoms omitted for clarity. Colour code: N blue, B pink, C black, F light green.

2.6.2 Cyclic Voltammetry

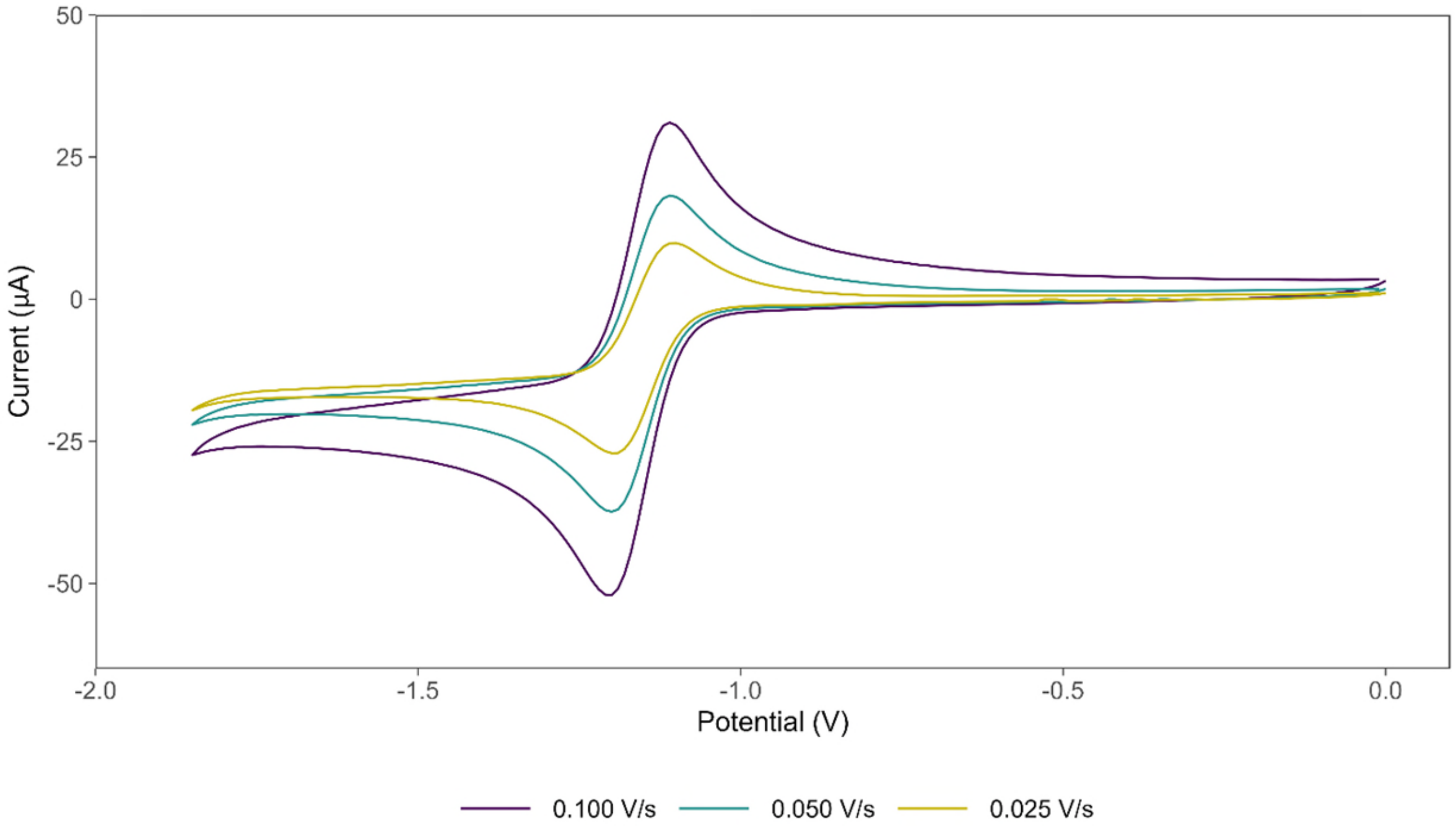

Figure S50: Cyclic voltammetry of [**5**][BArF$_{20}$]•(DCM)$_{0.5}$. Experimental details: Analyte; 2 mM. Electrolyte; 100 equiv. [Bu$_4$N][PF$_6$]. Solvent; acetonitrile. Working electrode; glassy carbon. Counter electrode; platinum. Reference electrode; leak-proof Ag/AgCl.

2.6.3 Electron Paramagnetic Resonance and Spin Density Data

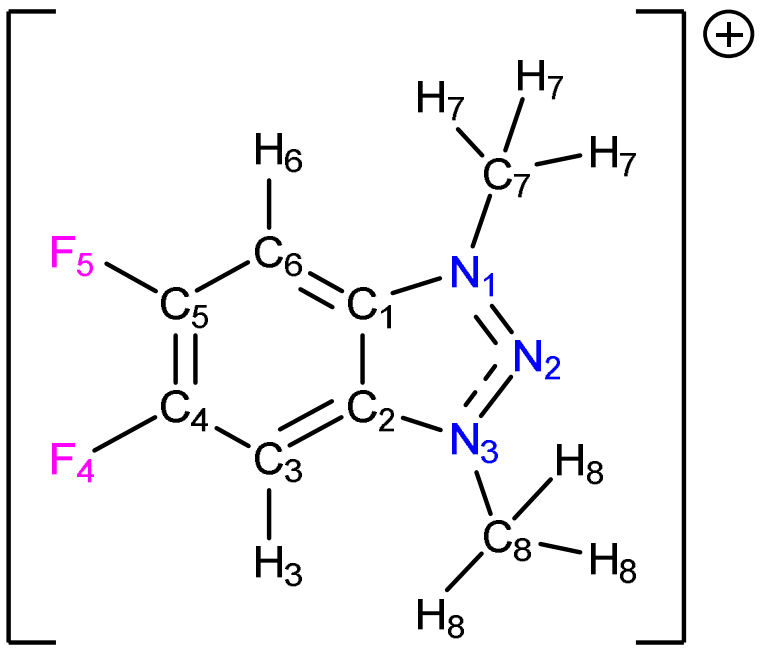

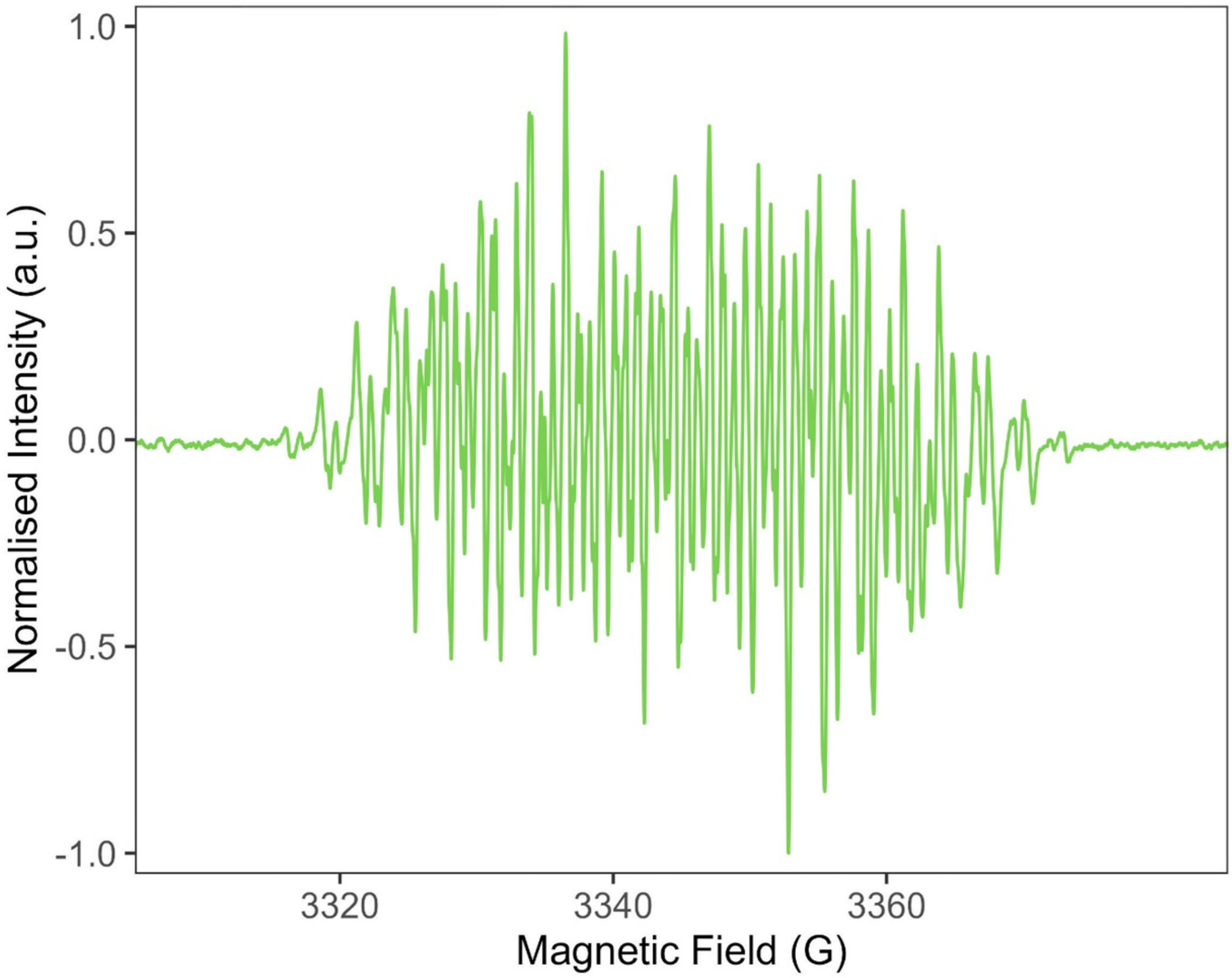

Figure S51. Electron paramagnetic resonance spectrum of [**5**][BArF$_{20}$]•(DCM)$_{0.5}$ after reduction by KC$_8$. Parameters: Solvent: toluene. Scans: 10. Modulation amplitude: 0.2 G. Frequency: 9.867 GHz. Temperature: 298 K. Sample was prepared according to the general procedure in Section 2.1.2.

Table S5. Spin densities (B3LYP/def2-TZVP) of **5˙**.

Atom	Mulliken Spin Density	Calculated A_{iso}
N1	0.112	5.443
N2	0.520	21.253
N3	0.110	5.448
C1	–0.048	NA
C2	–0.047	NA
C3	0.113	NA
C4	0.059	NA
C5	0.057	NA
C6	0.115	NA
C7	–0.007	NA
C8	–0.007	NA
F4	0.003	5.387
F5	0.003	5.390
H3	–0.005	11.184
H6	–0.005	11.185
3 × H7	0.004	5.112
3 × H8	0.004	5.110

2.7 4,5,6,7-Tetrafluoro-1,3-dimethyl-1H-benzo[d][1,2,3]triazol-3-ium tetrakis(pentafluorophenyl)borate - [6][BArF$_{20}$]

2.7.1 Synthesis

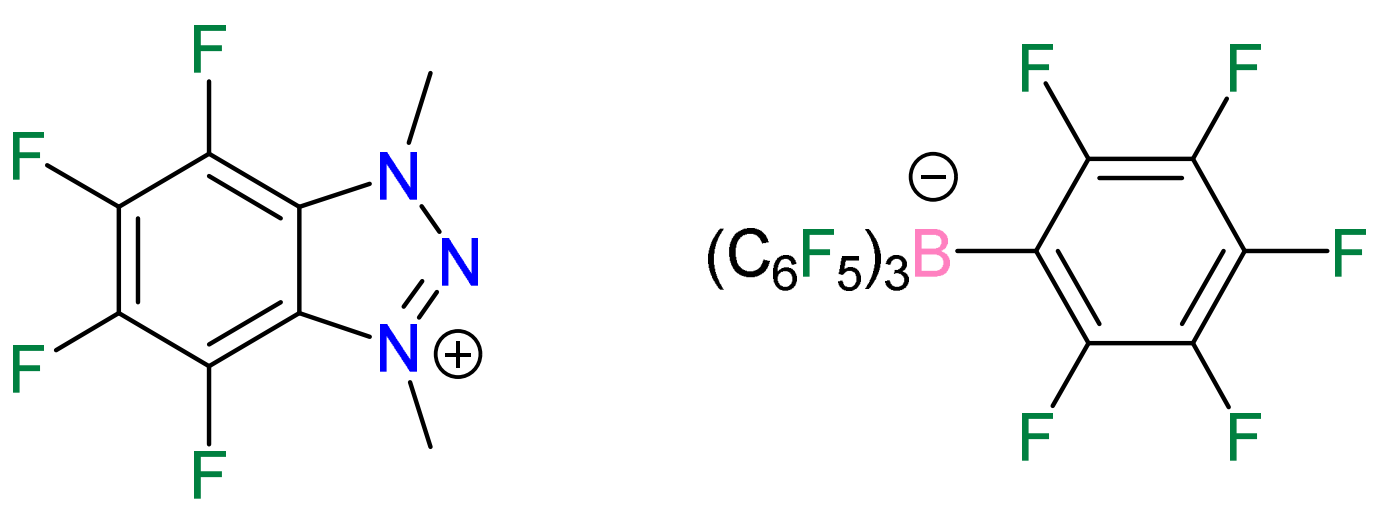

Compound [**6**][BArF$_{20}$] was prepared following the general procedure.

Yield: 91 mg (1.7 %).

Elemental analysis, Found: C, 42.97; H, 0.77; N, 4.51%. **Calc.** for $C_{32}H_6BF_{24}N_3$: C, 42.74; H, 0.67; N, 4.67%.

HR-ESI-MS: $[C_8H_6F_4N_3]^+$: 220.0489 (calc. 220.0493).

^{1}H NMR (500 MHz, CD_3CN): δ = 4.62 (s, 6H) ppm.

$^{13}C\{^1H\}$ NMR (126 MHz, CD_3CN): δ = 149.13 (d, $^1J_{CF}$ = 240.4 Hz, *o*-C_6F_5), 143.35 (d, $^1J_{CF}$ = 271.0 Hz, Ar*C*F), 139.32 (d, $^1J_{CF}$ = 245.4 Hz, *p*-C_6F_5), 137.37 (d, $^1J_{CF}$ = 245.5 Hz, *m*-C_6F_5), 134.94 (d, $^1J_{CF}$ = 271.7 Hz, Ar*C*F), 124.78 (s, Ar*C*N), 41.84 (s, N*C*H_3) ppm. The C atom at the *ipso*-C_6F_5 position was not observed.

$^{11}B\{^1H\}$ NMR (160 MHz, CD_3CN): δ = –16.69 ppm.

^{19}F NMR (470 MHz, CD_3CN): δ = –133.76 (s, 8F), –148.26 (dt, J = 13.9, 3.9 Hz, 2F), –153.91 (dt, J = 13.4, 3.7 Hz, 2F), –163.97 (t, J = 19.6 Hz, 4F), –168.38 (q, J = 13.0 Hz, 8F) ppm.

$^{19}F\{^1H\}$ NMR (470 MHz, CD_3CN): δ = –133.78 (s, 8F), –148.26 (dt, J = 13.9, 3.9 Hz, 2F), –153.91 (dt, J = 13.4, 3.7 Hz, 2F), –163.97 (t, J = 19.6 Hz, 4F), –168.40 (q, J = 13.0 Hz, 8F) ppm.

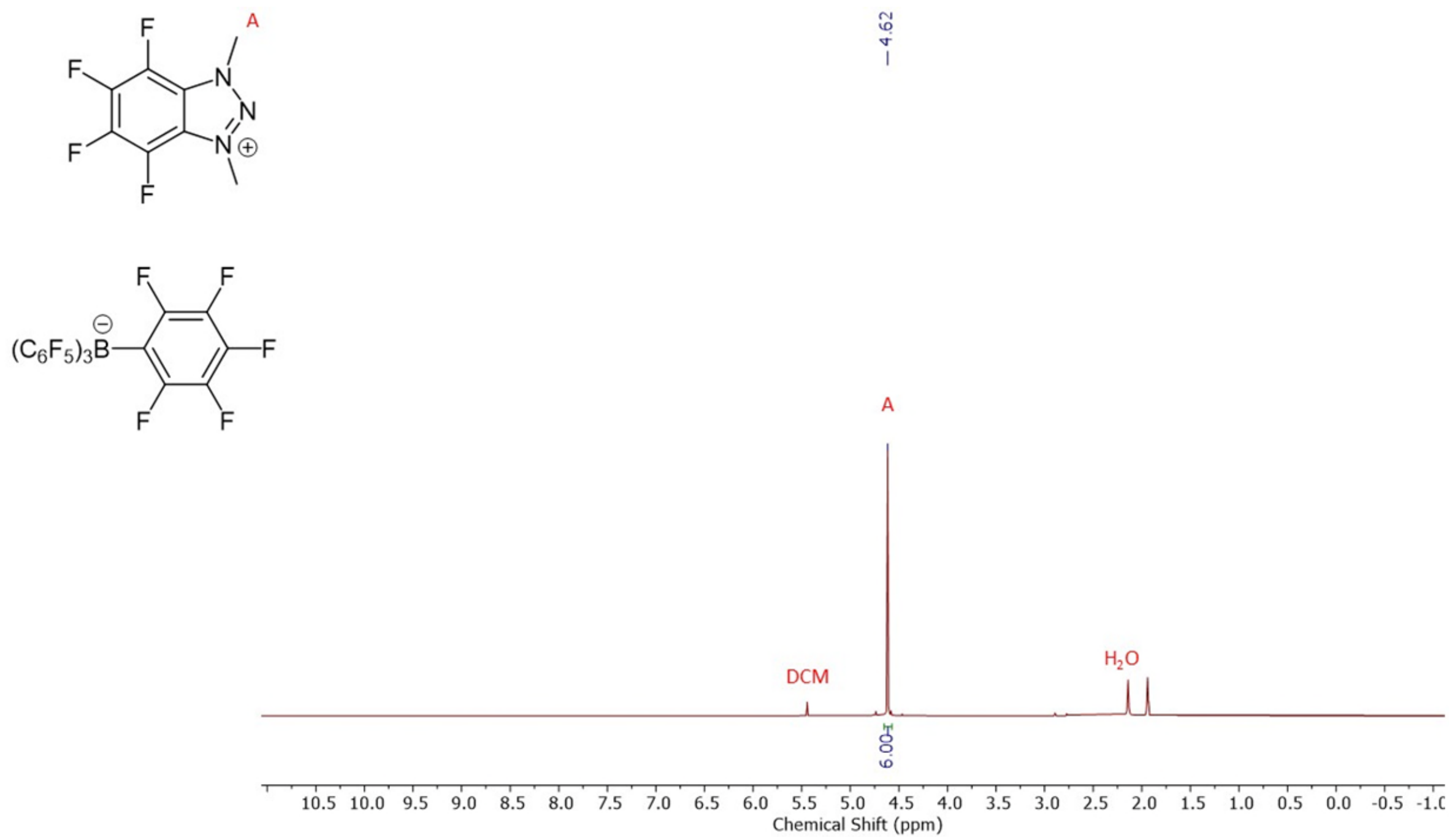

Figure S52. ^1H NMR spectrum (CD_3CN, 500 MHz) of [**6**][$BArF_{20}$] at room temperature.

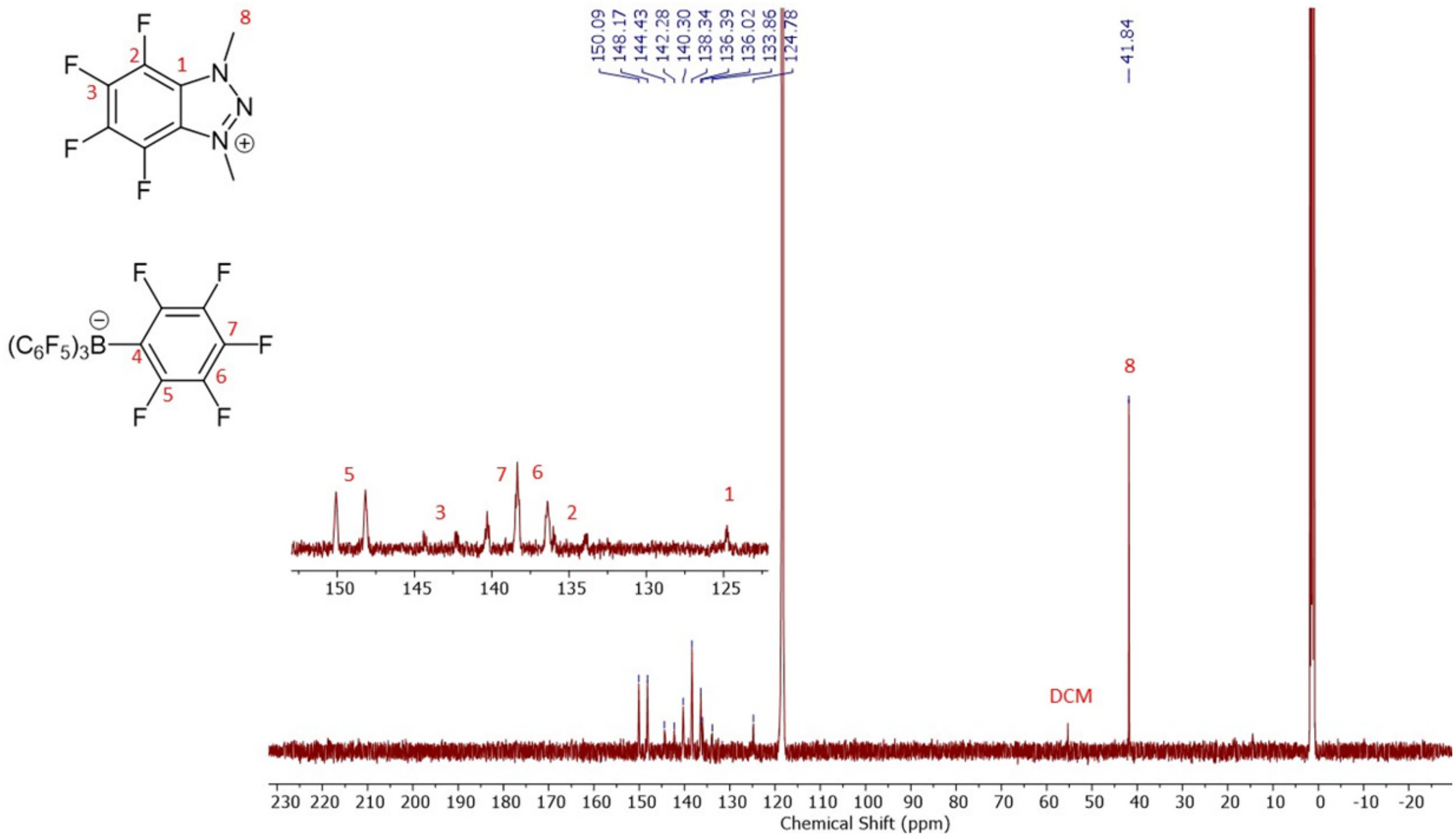

Figure S53. $^{13}C\{^1H\}$ NMR spectrum (CD_3CN, 126 MHz) of [**6**][$BArF_{20}$] at room temperature. The C atom at the *ipso*-C_6F_5 position was not observed.

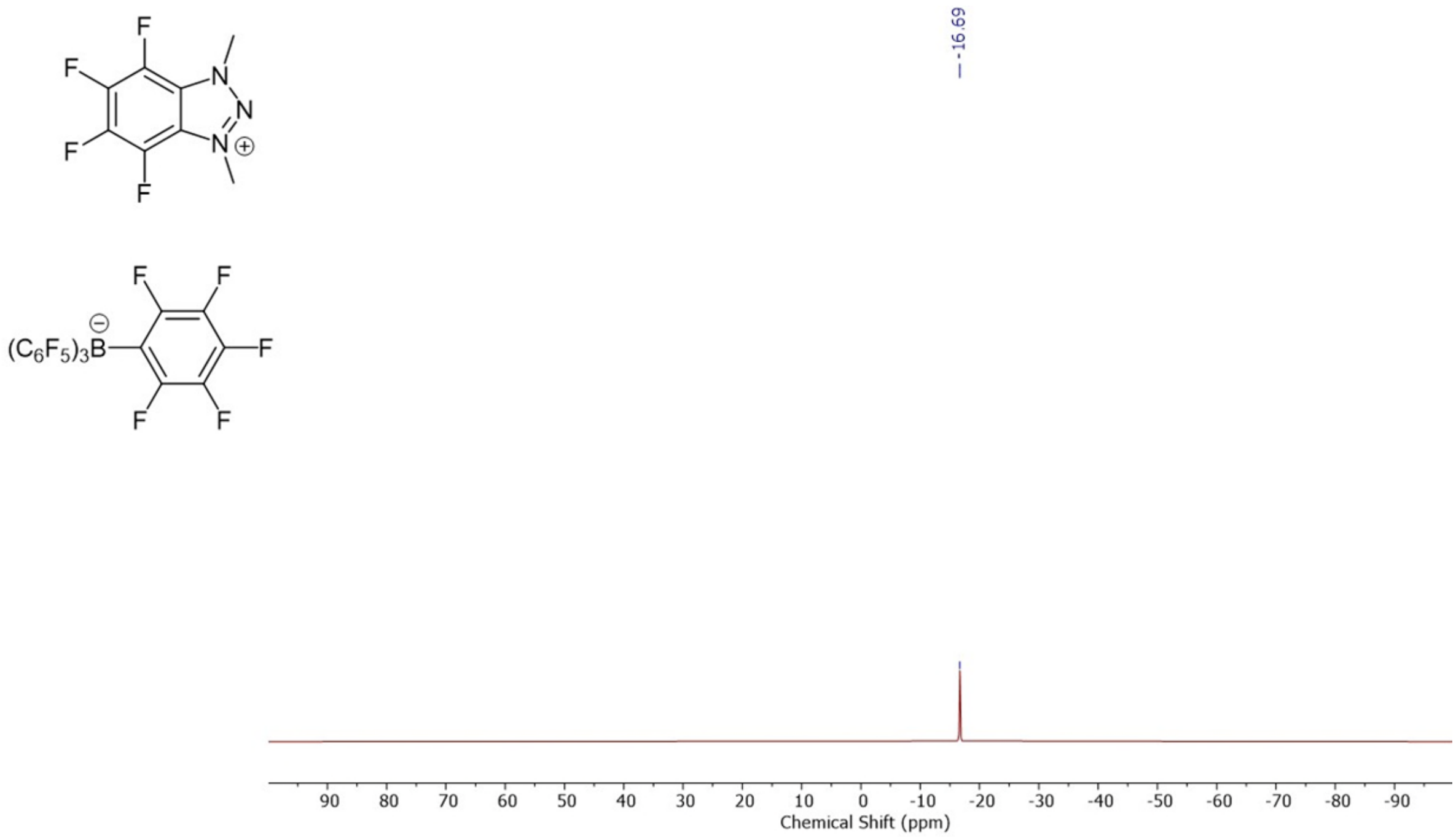

Figure S54. $^{11}B\{^1H\}$ NMR spectrum (CD_3CN, 160 MHz) of [**6**][$BArF_{20}$] at room temperature.

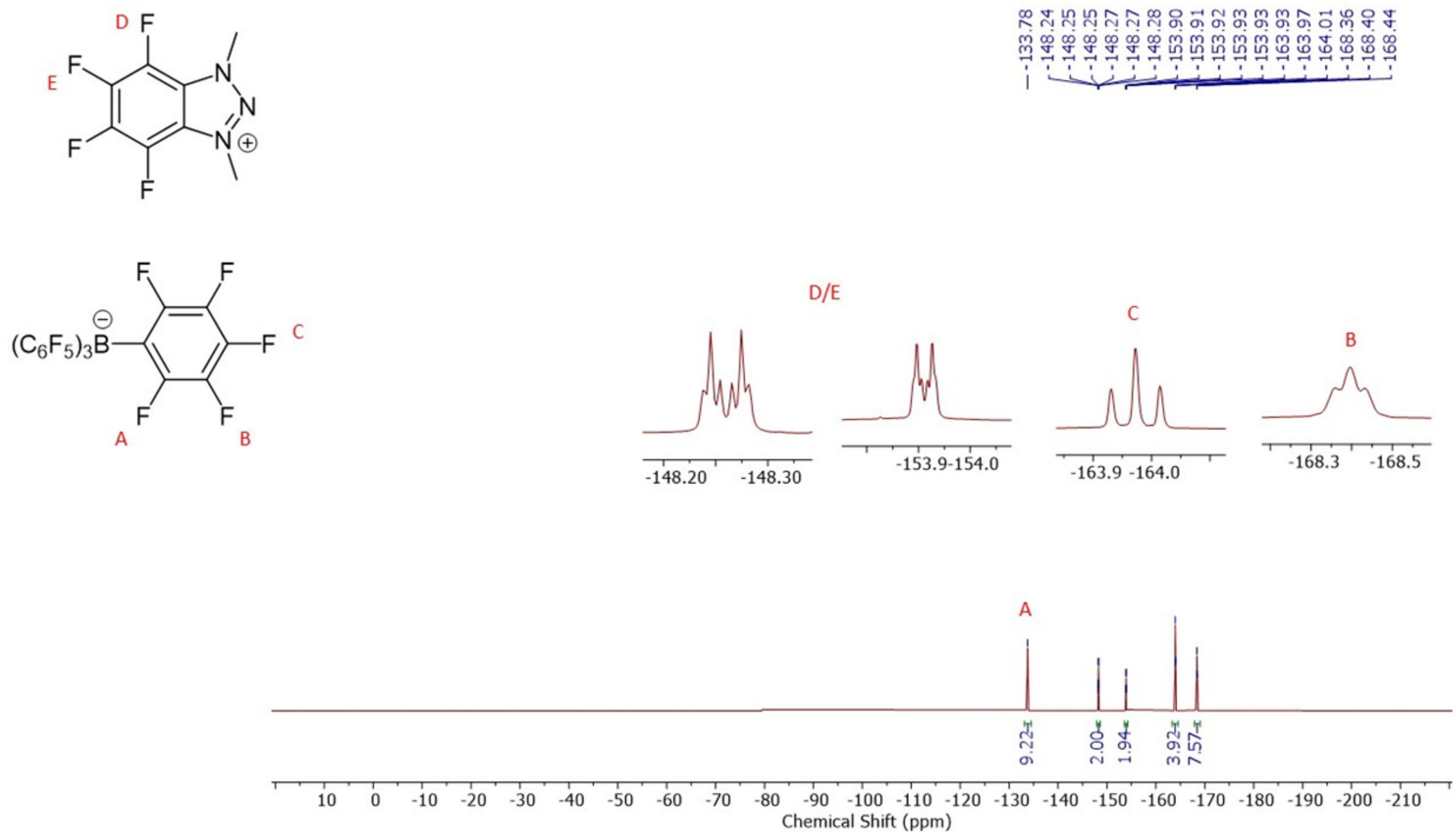

Figure S55. $^{19}F\{^{1}H\}$ NMR spectrum (CD_3CN, 470 MHz) of [**6**][$BArF_{20}$] at room temperature.

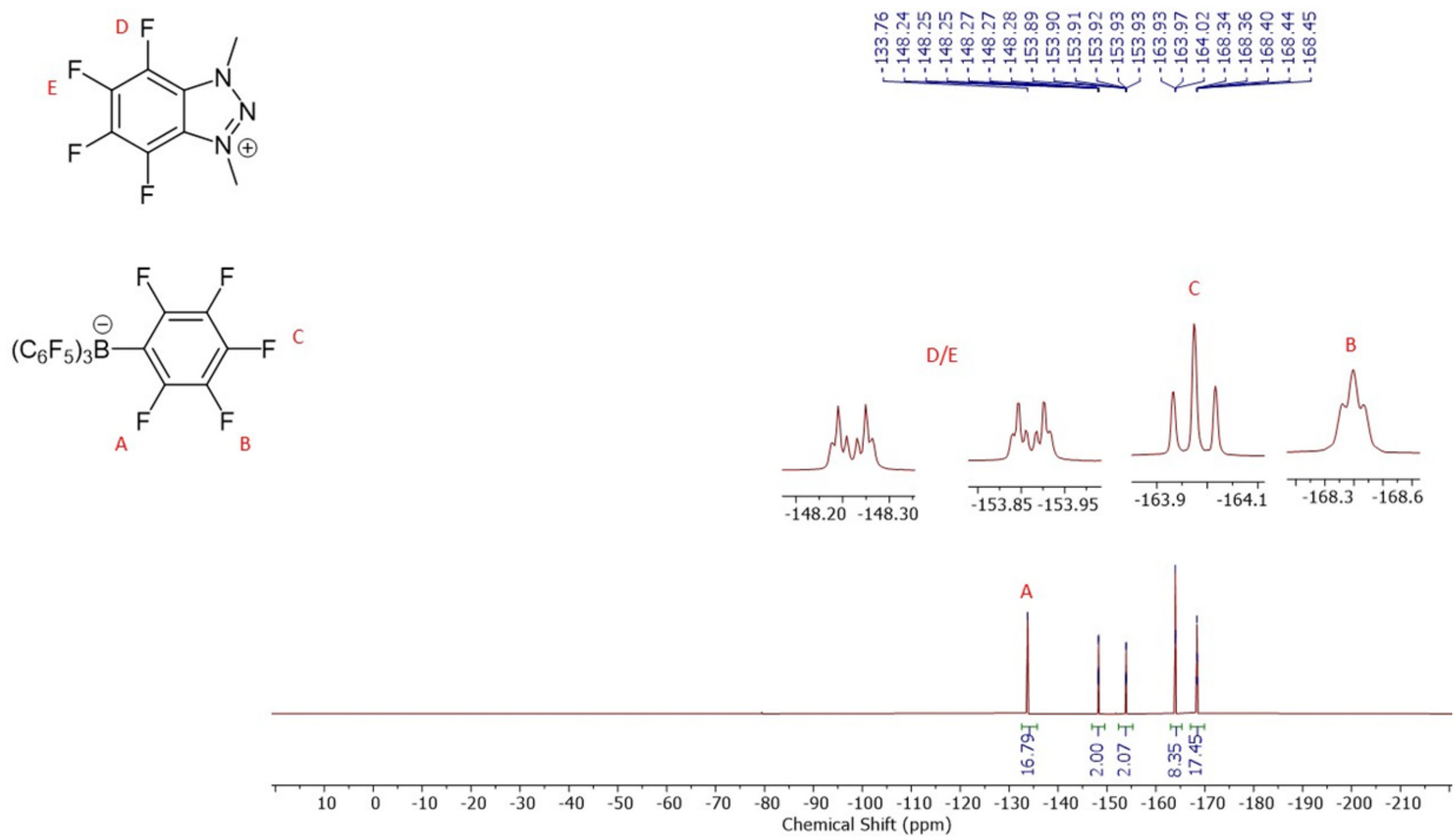

Figure S56. ^{19}F NMR spectrum (CD_3CN, 470 MHz) of [**6**][$BArF_{20}$] at room temperature.

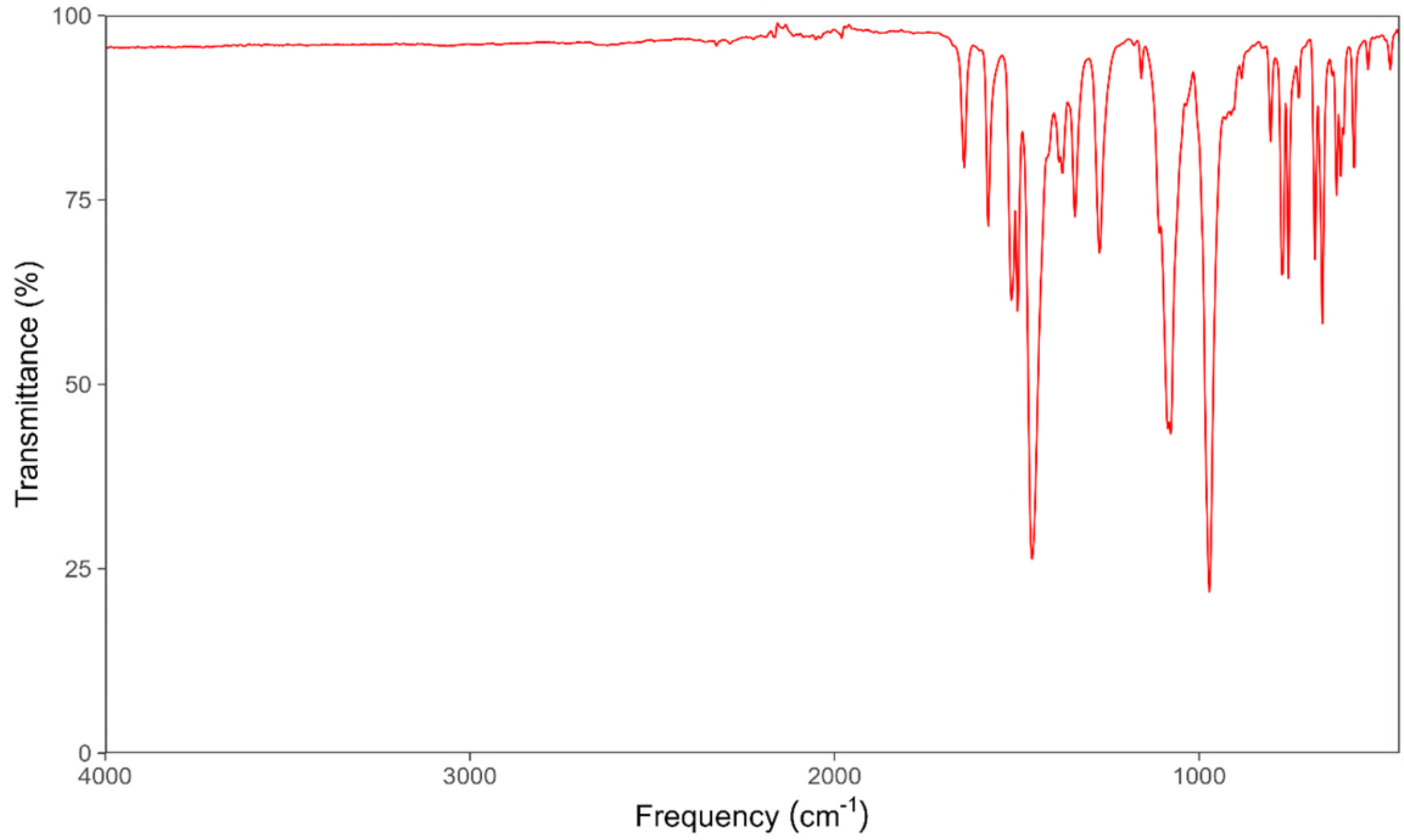

Figure S57. Experimental IR spectrum of [**6**][BArF20].

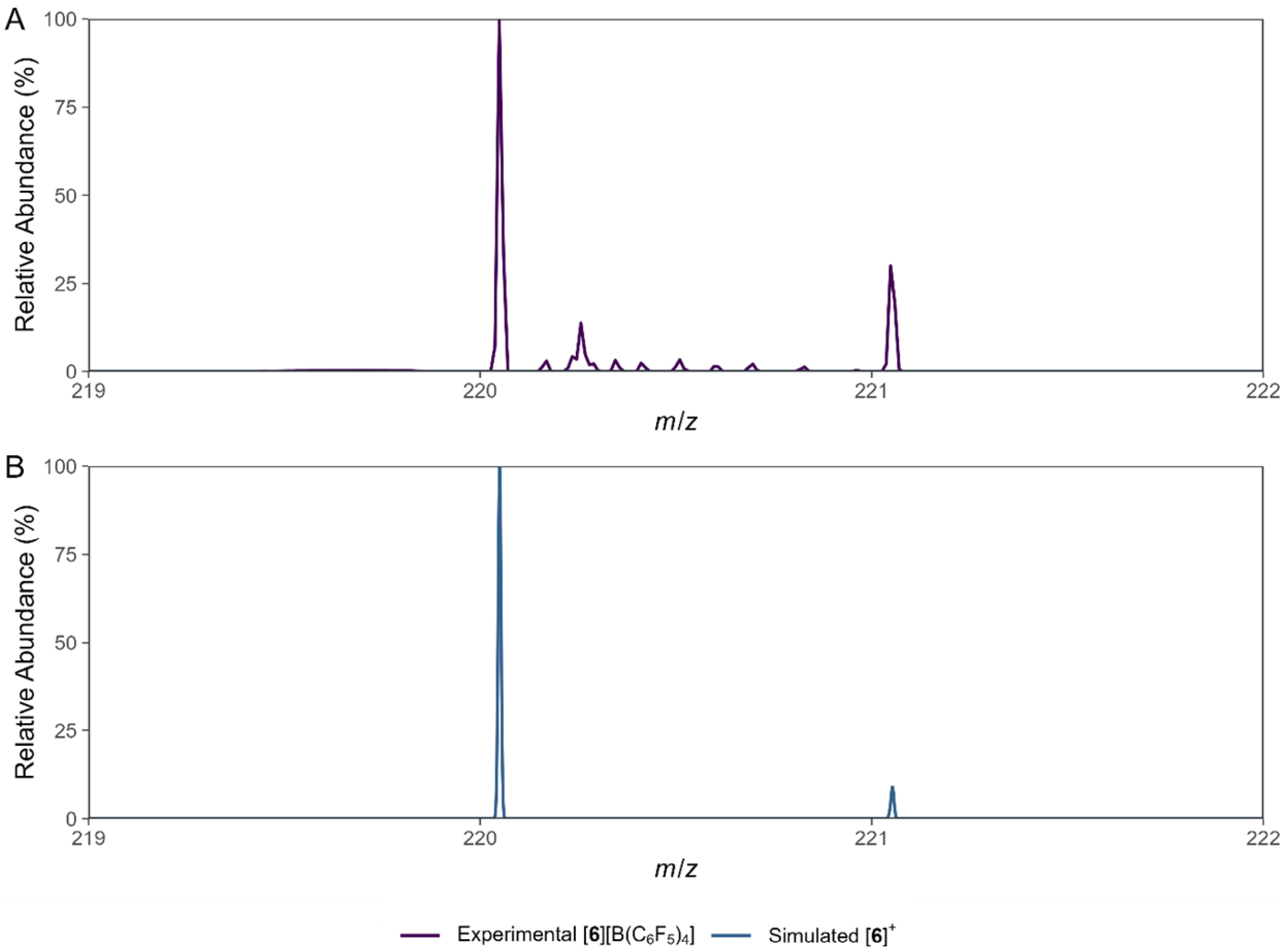

Figure S58. (A) Experimental HR-ESI-MS spectrum for [**6**][BArF20]. (B) Simulated HR-ESI-MS spectrum for [**6**]$^{+}$.

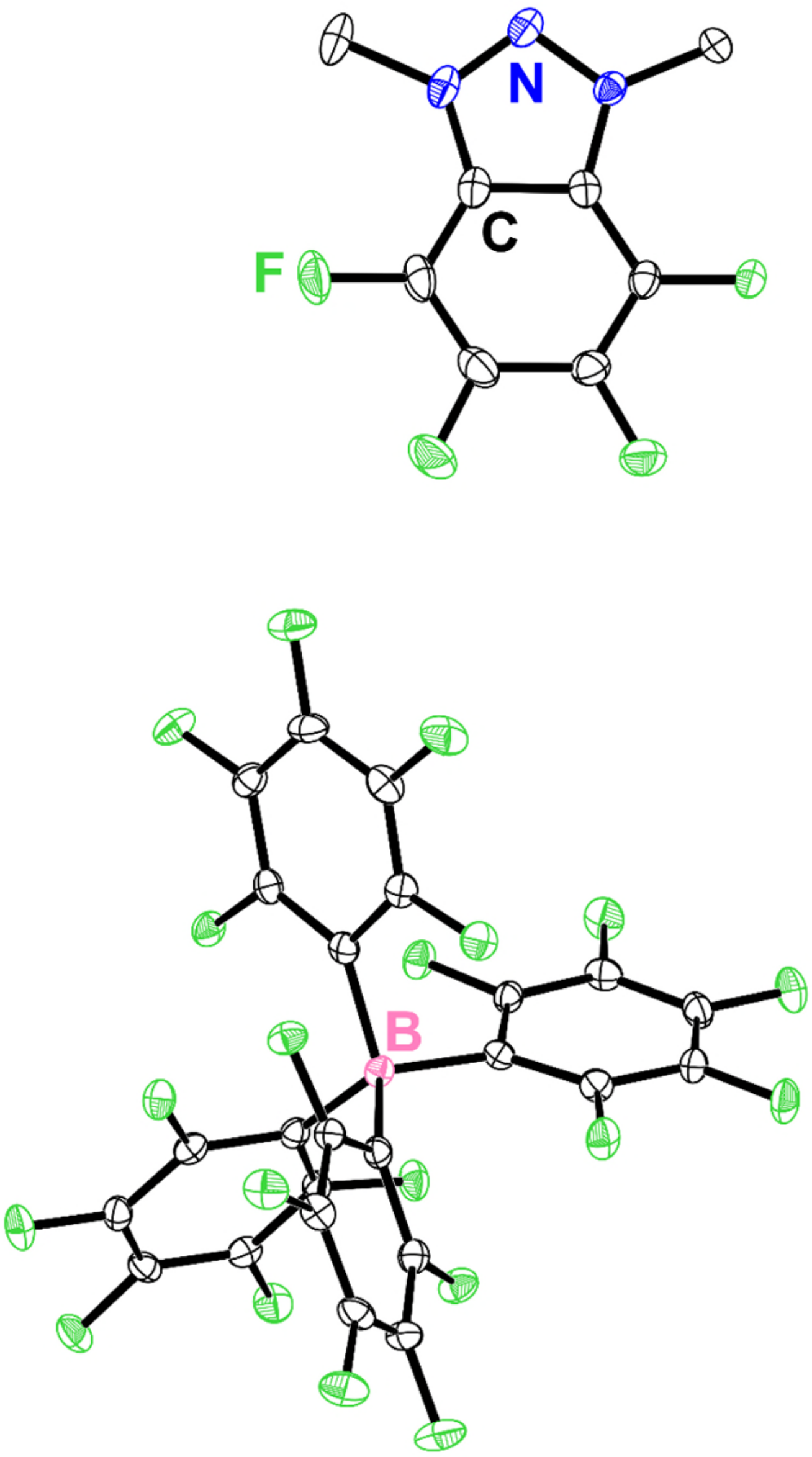

Figure S59. Thermal ellipsoid plot (50% probability) of [**6**][$BArF_{20}$]. H atoms omitted for clarity. Colour code: N blue, B pink, C black, F light green.

2.7.2 Cyclic Voltammetry

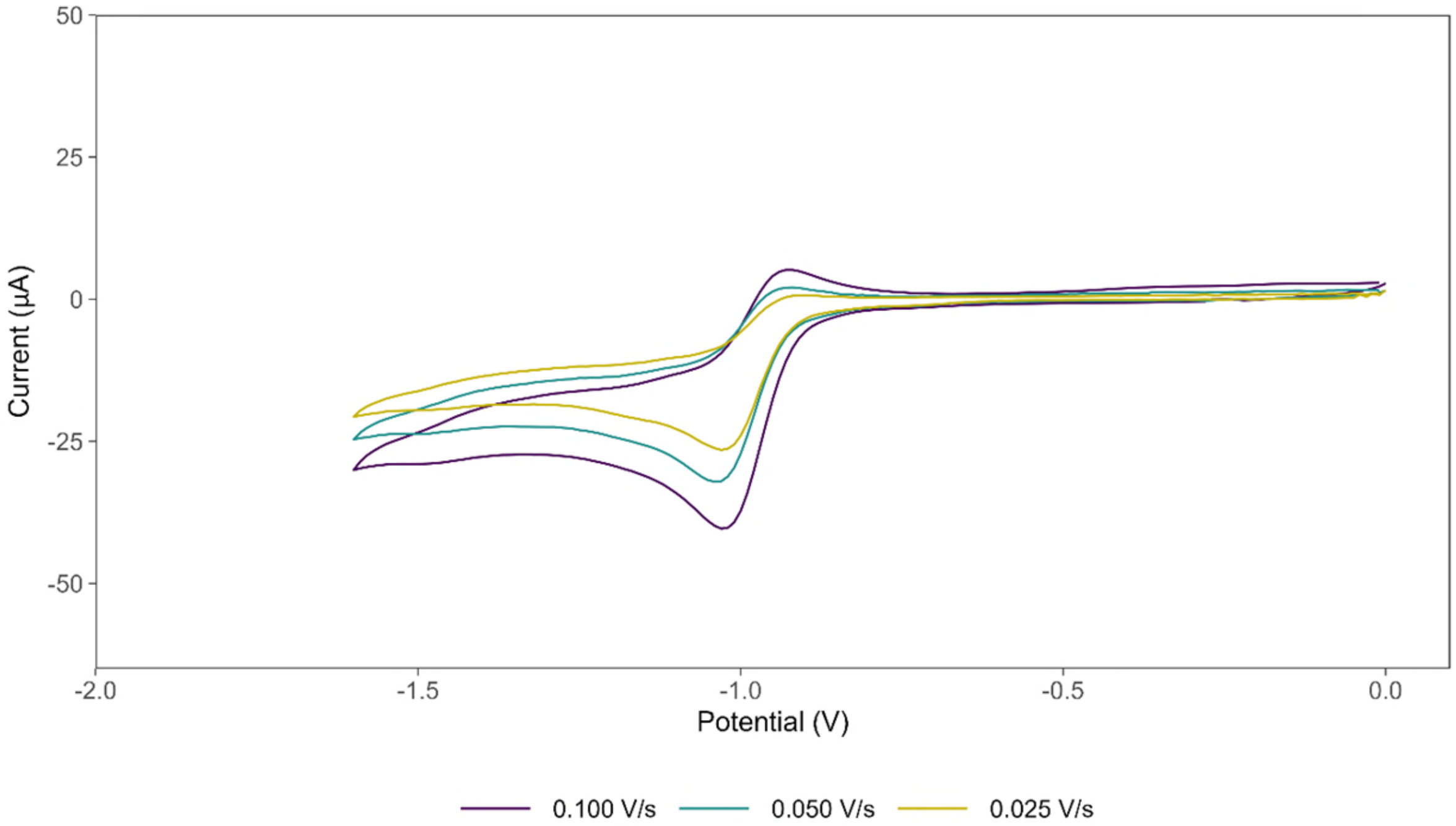

Figure S60: Cyclic voltammetry of [**6**][BArF$_{20}$]. Experimental details: Analyte; 2 mM. Electrolyte; 100 equiv. [Bu_4N][PF_6]. Solvent; acetonitrile. Working electrode; glassy carbon. Counter electrode; platinum. Reference electrode; leak-proof Ag/AgCl.

2.7.3 Spin Density Data

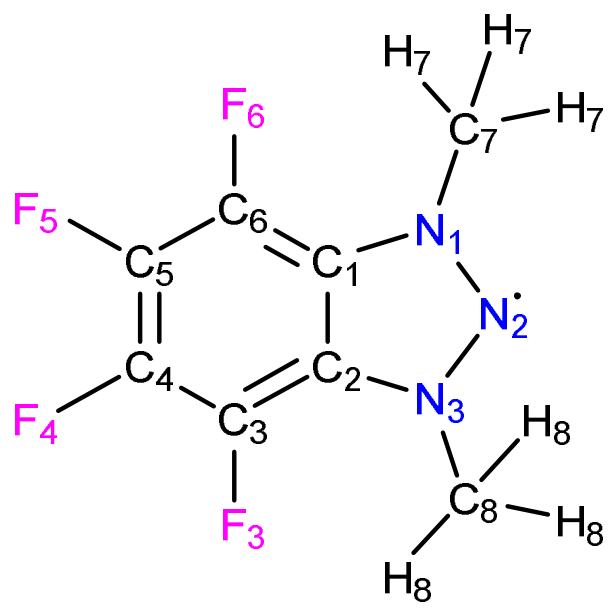

Table S6. Spin densities (B3LYP/def2-TZVP) of **6•**.

Atom	Mulliken Spin Density
N1	0.130
N2	0.556
N3	0.129
C1	–0.034
C2	–0.032
C3	0.078
C4	0.037
C5	0.033
C6	0.081
C7	–0.007
C8	–0.008
F3	0.004
F4	0.002
F5	0.002
F6	0.003
3 × H7	0.004
3 × H8	0.004

2.8 5-Methyl-1,3-dimethyl-1H-benzo[d][1,2,3]triazol-3-ium tetrakis(pentafluorophenyl)borate - [7][BArF$_{20}$]

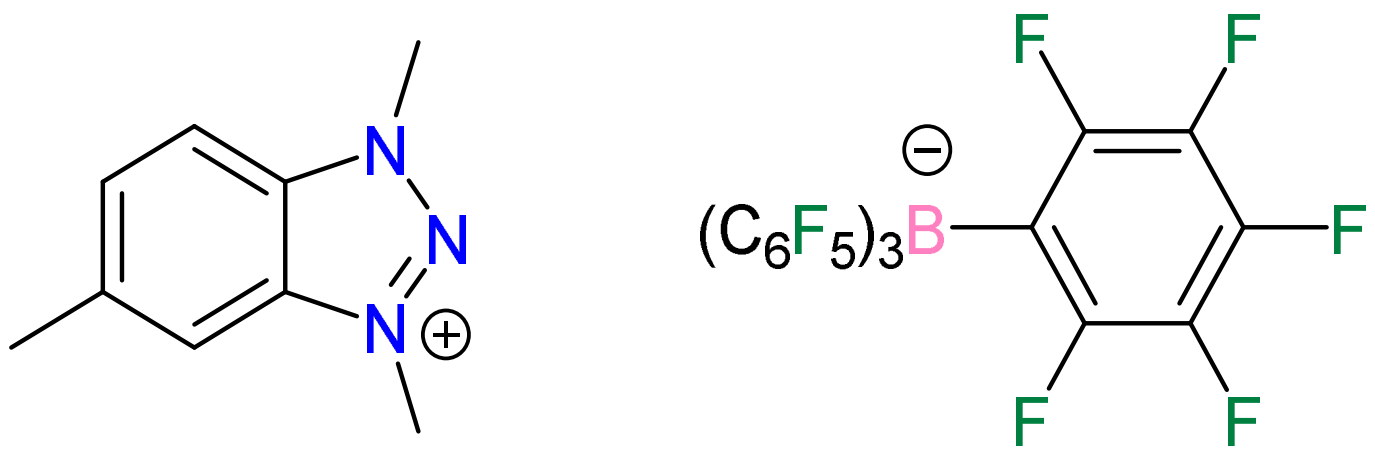

2.8.1 Synthesis

Compound [**7**][BArF$_{20}$] was prepared following the general procedure.

Yield: 86 mg (1.2 %).

Elemental analysis, Found: C, 48.02; H, 1.43; N, 4.82%. **Calc.** for $C_{33}H_{12}BF_{20}N_3$: C, 47.12; H, 1.44; N, 5.00%. Elemental analyses for [**7**][BArF$_{20}$] were repeatedly unsuccessful; the best results obtained are provided.

HR-ESI-MS: [$C_9H_{12}N_3$]$^+$: 162.1038 (calc. 162.1026).

^{1}H NMR (500 MHz, CD_3CN): δ = 7.94 (d, J = 8.9 Hz, 1H), 7.85 (s, 1H), 7.78 (d, J = 8.9 Hz, 1H), 4.47 (s, 3H), 4.44 (s, 3H), 2.63 (d, J = 0.9 Hz, 3H) ppm.

^{13}C{^{1}H} NMR (126 MHz, CD_3CN): δ = 149.11 (d, $^1J_{CF}$ = 239.8 Hz, *o*-C_6F_5), 144.17 (s, Ar*C*CH$_3$), 139.31 (d, $^1J_{CF}$ = 241.4 Hz, *p*-C_6F_5), 137.37 (d, $^1J_{CF}$ = 245.9 Hz, *m*-C_6F_5), 136.89 (s, Ar*C*N), 135.07 (s, Ar*C*N), 134.57 (s, Ar*C*H), 125.83 (s, *ipso*-C_6F_5), 113.89 (s, Ar*C*H), 112.96 (s, Ar*C*H), 38.68 (s, N*C*H$_3$), 38.45 (s, N*C*H$_3$), 22.07 (s, ArC*C*H$_3$) ppm.

^{11}B{^{1}H} NMR (160 MHz, CD_3CN): δ = –16.69 ppm.

^{19}F NMR (470 MHz, CD_3CN): δ = –133.76 (s, 8F), –163.95 (t, *J* = 19.5 Hz, 4F), –168.37 (t, *J* = 19.5 Hz, 8F) ppm.

^{19}F NMR{^{1}H} (470 MHz, CD_3CN): δ = –133.76 (s, 8F), –163.95 (t, *J* = 19.5 Hz, 4F), –168.38 (t, *J* = 19.5 Hz, 8F) ppm.

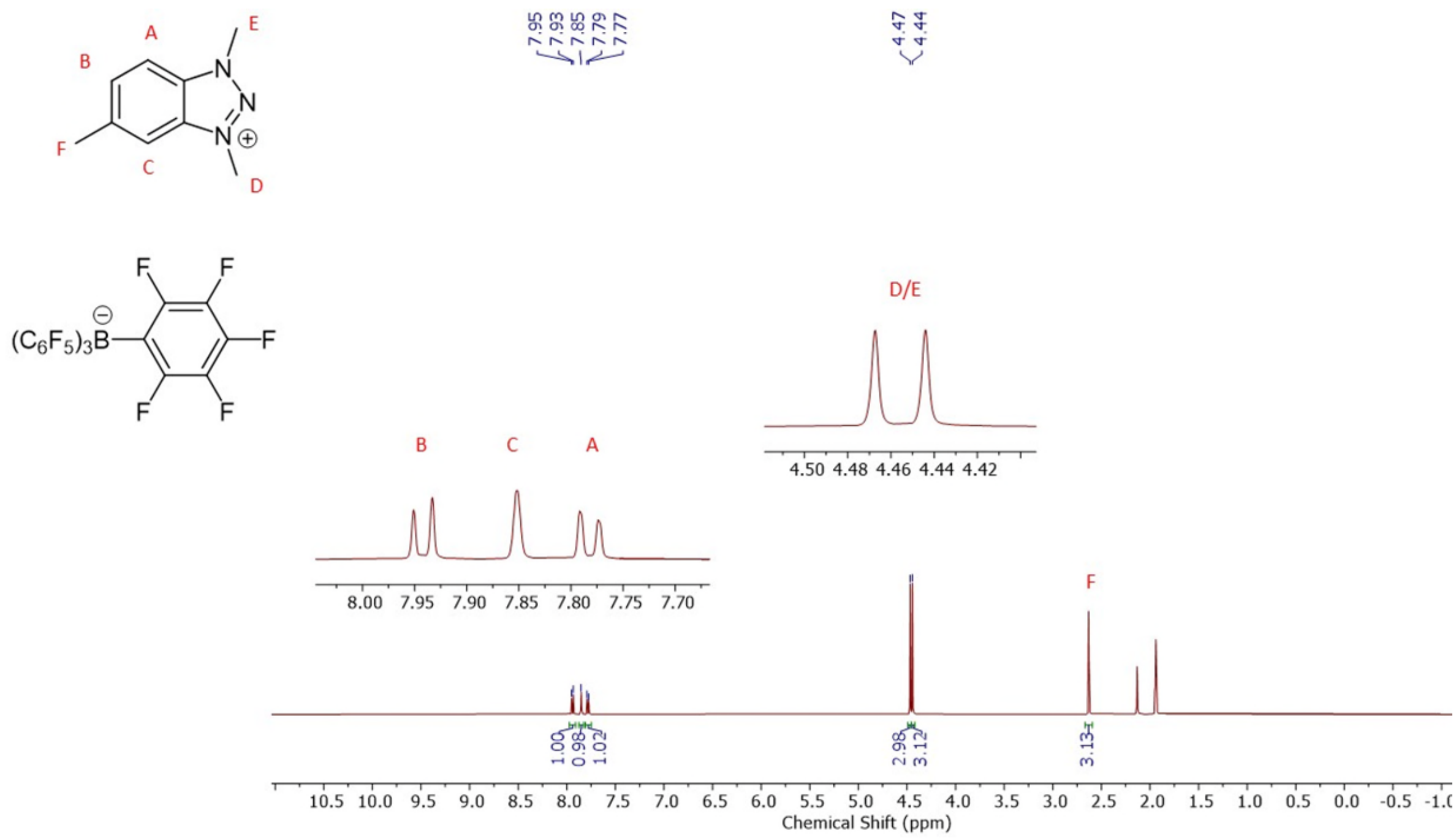

Figure S61. ^{1}H NMR spectrum (CD_3CN, 500 MHz) of [**7**][$BArF_{20}$] at room temperature.

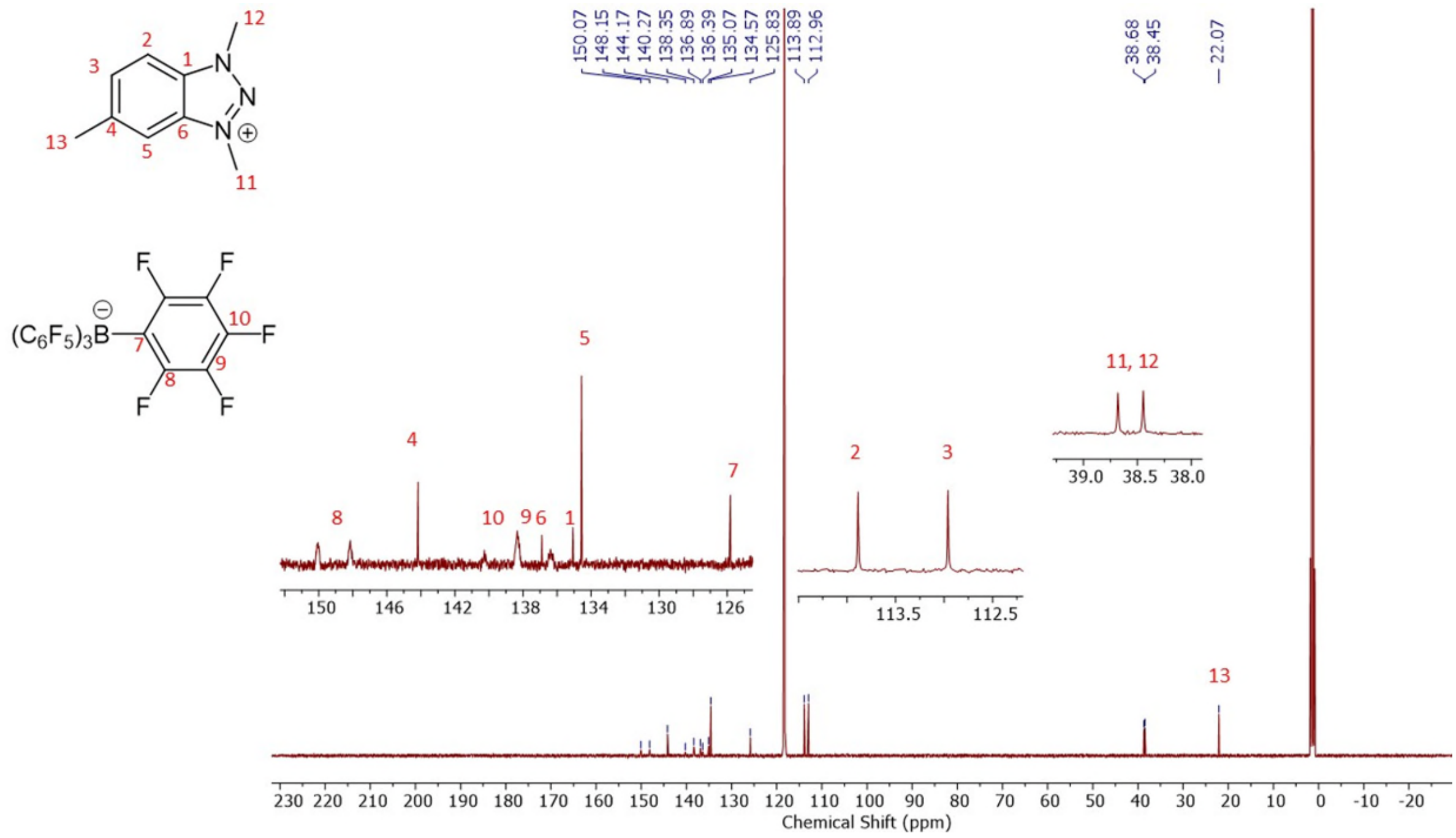

Figure S62. $^{13}C\{^{1}H\}$ NMR spectrum (CD_3CN, 126 MHz) of [**7**][$BArF_{20}$] at room temperature.

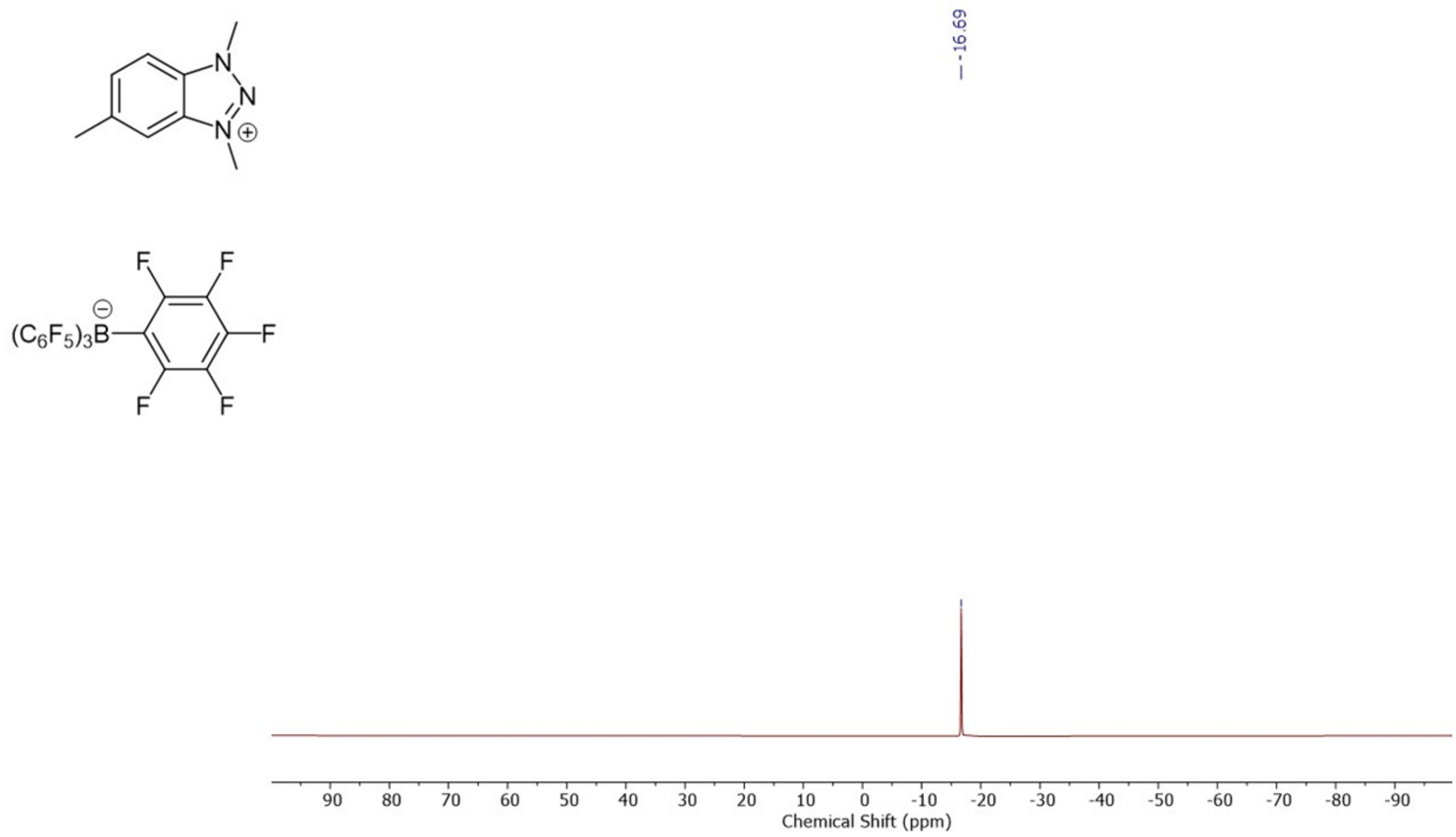

Figure S63. $^{11}B\{^1H\}$ NMR spectrum (CD_3CN, 160 MHz) of [**7**][$BArF_{20}$] at room temperature.

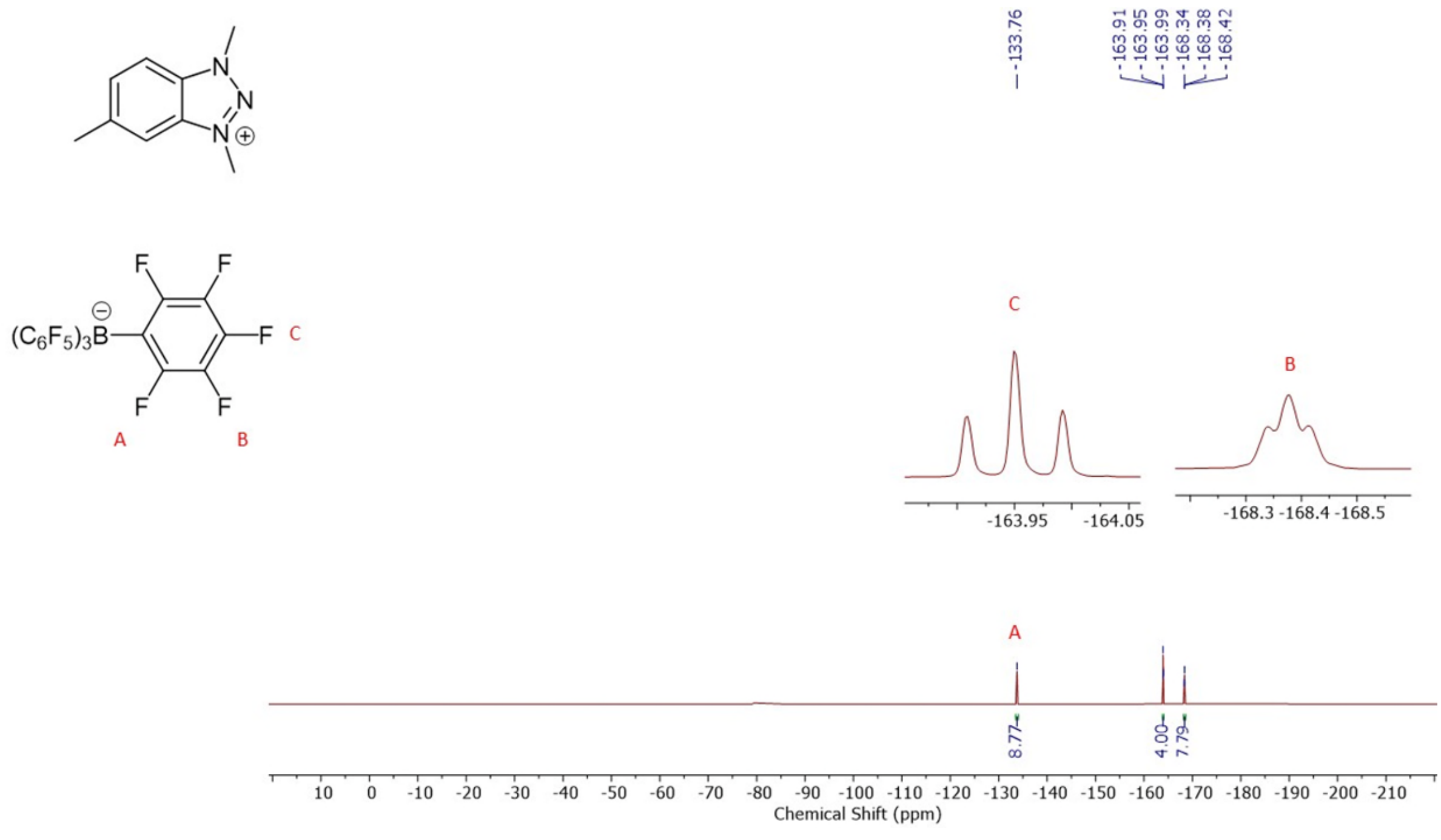

Figure S64. $^{19}F\{^1H\}$ NMR spectrum (CD_3CN, 470 MHz) of [**7**][$BArF_{20}$] at room temperature.

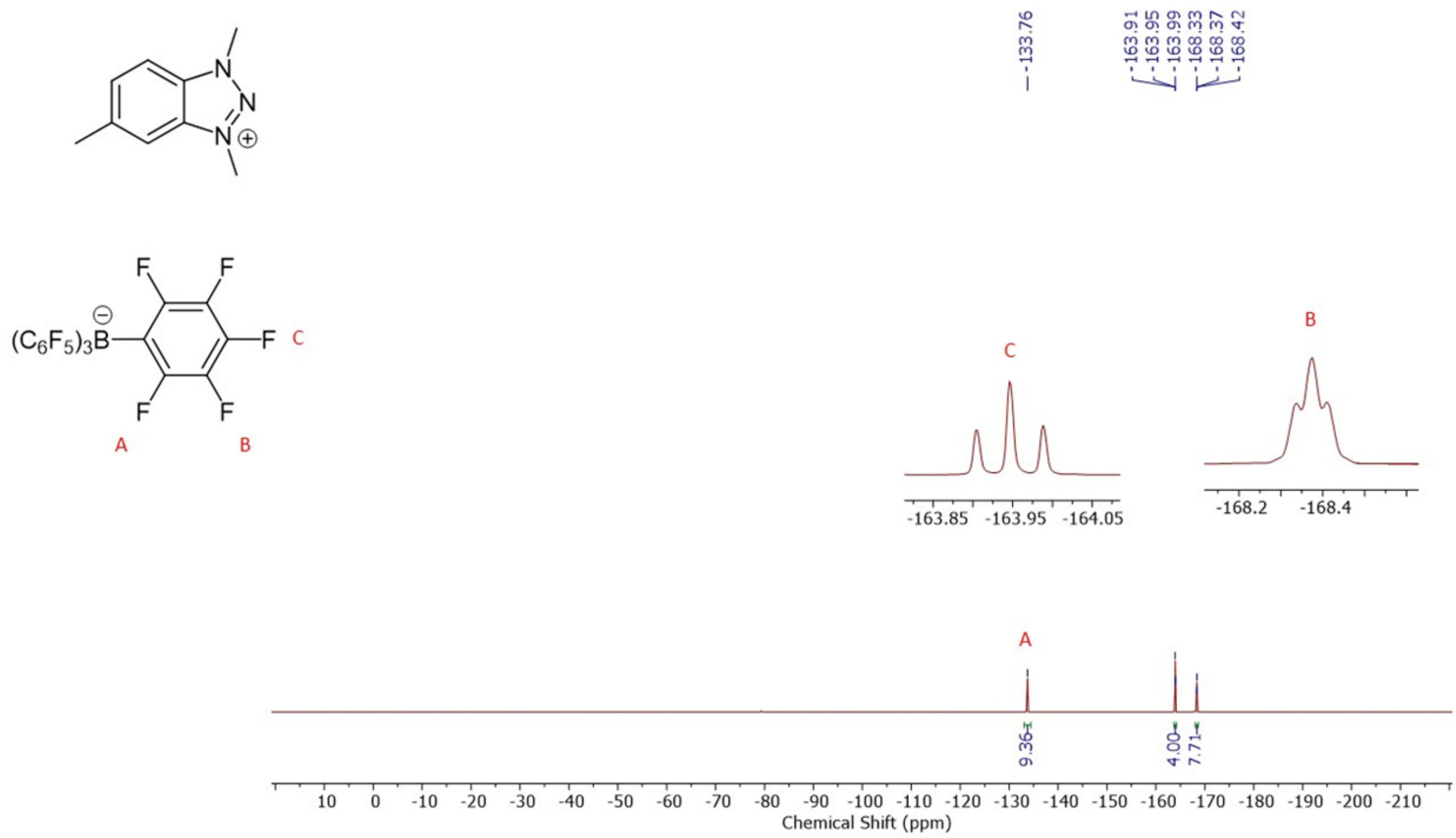

Figure S65. ^{19}F NMR spectrum (CD_3CN, 470 MHz) of [**7**][$BArF_{20}$] at room temperature.

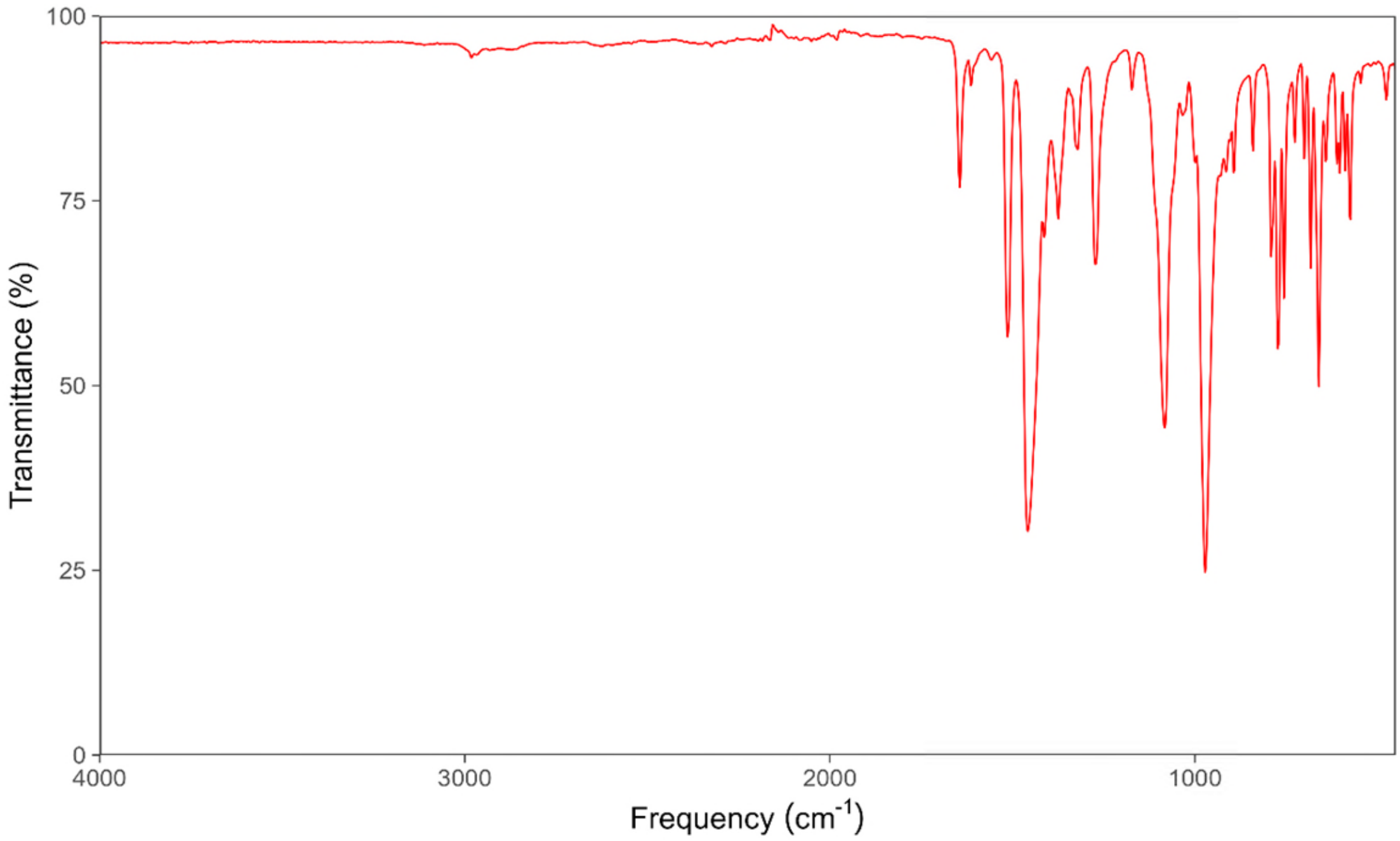

Figure S66. Experimental IR spectrum of [**7**][$BArF_{20}$].

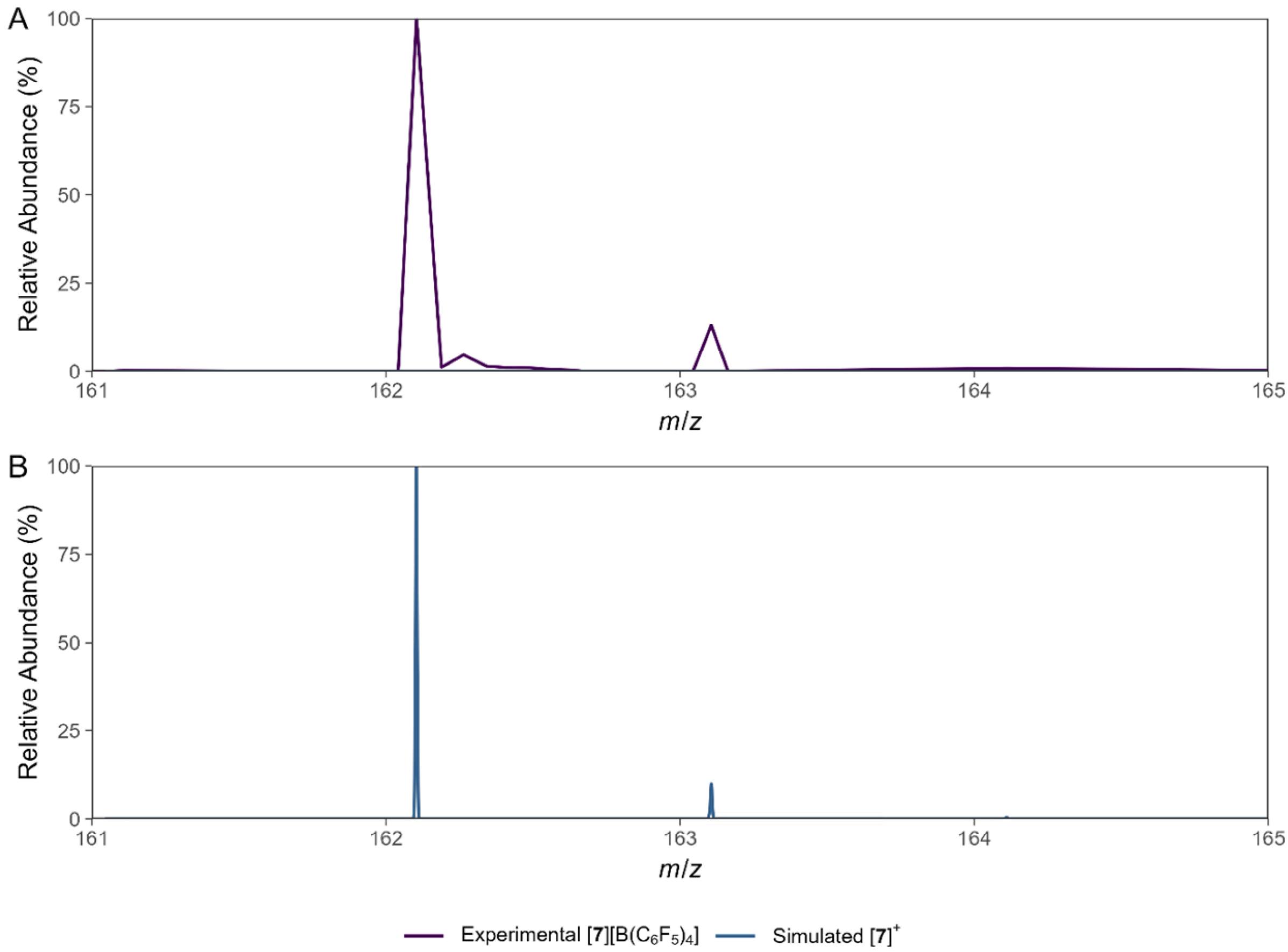

Figure S67. (A) Experimental HR-ESI-MS spectrum for [**7**][$BArF_{20}$]. (B) Simulated HR-ESI-MS spectrum for [**7**]$^{+}$.

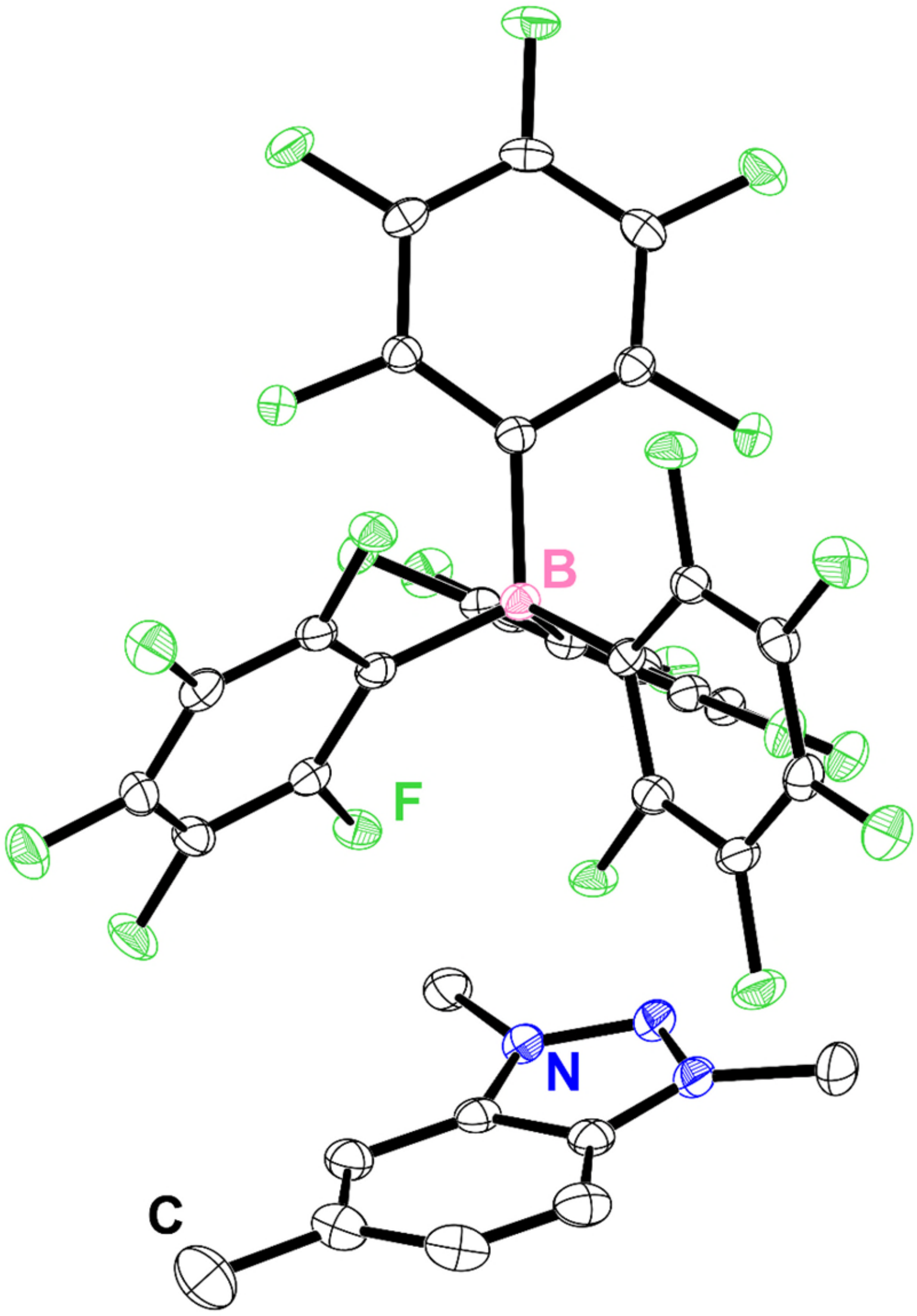

Figure S68. Thermal ellipsoid plot (50% probability) of [**7**][$BArF_{20}$]. H atoms omitted for clarity. Colour code: N blue, B pink, C black, F light green.

3. Lewis Acidity Studies

3.1 Canonical Molecular Orbital energies (Hartree) of [1]$^+$-[6]$^+$ (B3LYP/def2-TZVP).

Table S7. Canonical Molecular Orbital energies (Hartree) of [**1**]$^+$-[**6**]$^+$ (B3LYP/def2-TZVP).

	[**1**]$^+$	[**2**]$^+$	[**3**]$^+$	[**4**]$^+$	[**5**]$^+$	[**6**]$^+$	[**A**]$^+$
HOMO−4	–0.533	–0.502	–0.500	–0.532	–0.534	–0.541	−0.525
HOMO−3	–0.523	–0.488	–0.469	–0.531	–0.526	–0.534	−0.505
HOMO−2	–0.502	–0.451	–0.427	–0.505	–0.506	–0.513	−0.497
HOMO−1	–0.434	–0.429	–0.423	–0.440	–0.428	–0.443	−0.432
HOMO	–0.418	–0.405	–0.396	–0.418	–0.427	–0.421	−0.417
LUMO	–0.242	–0.241	–0.241	–0.247	–0.246	–0.255	−0.236
LUMO+1	–0.173	–0.175	–0.174	–0.180	–0.177	–0.191	−0.167
LUMO+2	–0.167	–0.169	–0.168	–0.168	–0.173	–0.173	−0.161
LUMO+3	–0.108	–0.117	–0.140	–0.118	–0.112	–0.161	−0.109
LUMO+4	–0.098	–0.105	–0.105	–0.105	–0.108	–0.120	−0.100

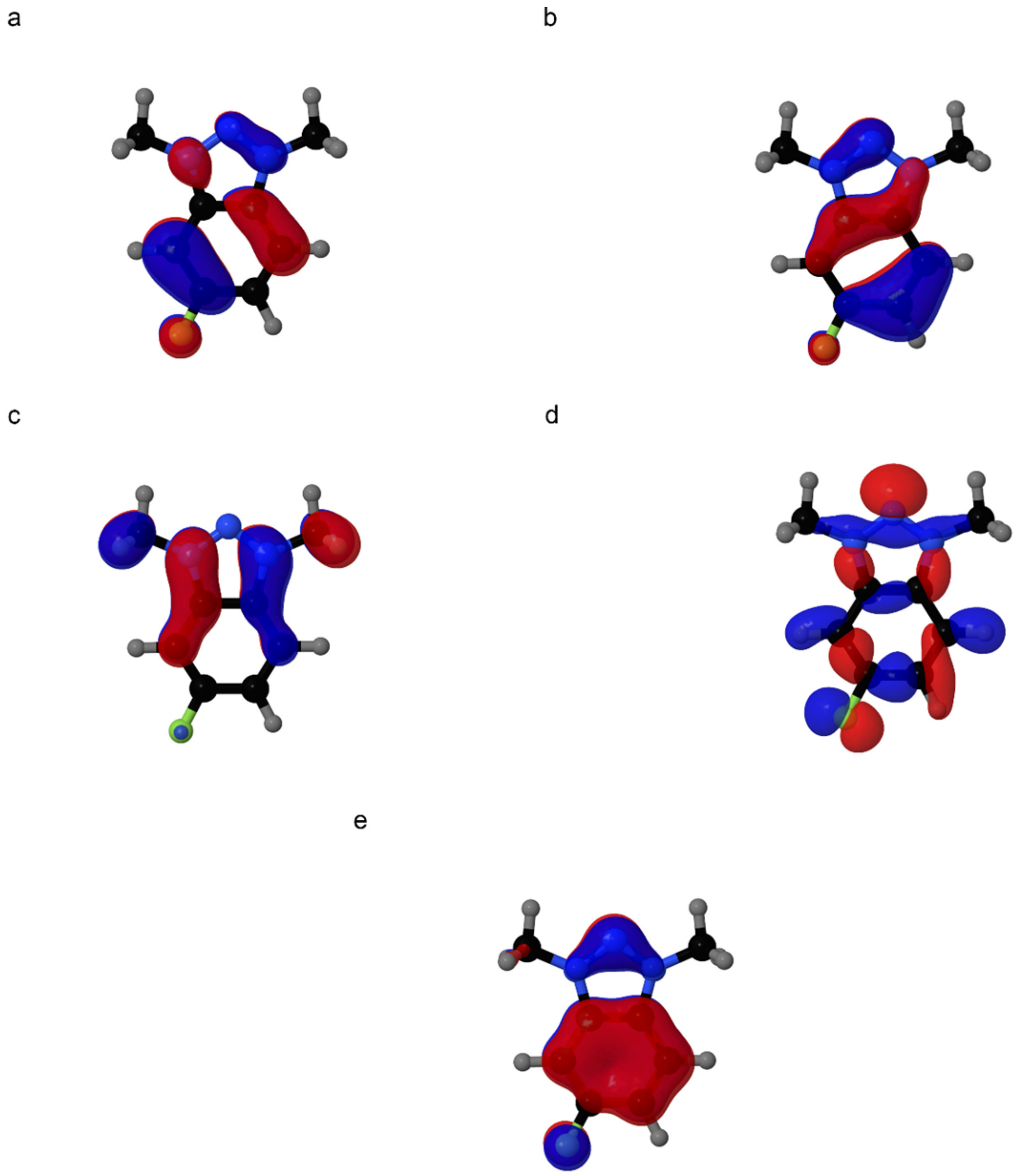

Figure S69. (a) HOMO, (b) HOMO–1, (c) HOMO–2, (d) HOMO–3, and (e) HOMO–4 of [**1**]$^{+}$. Canonical molecular orbitals were calculated at the B3LYP/def2-TZVP level of theory (isosurface value = 0.04).

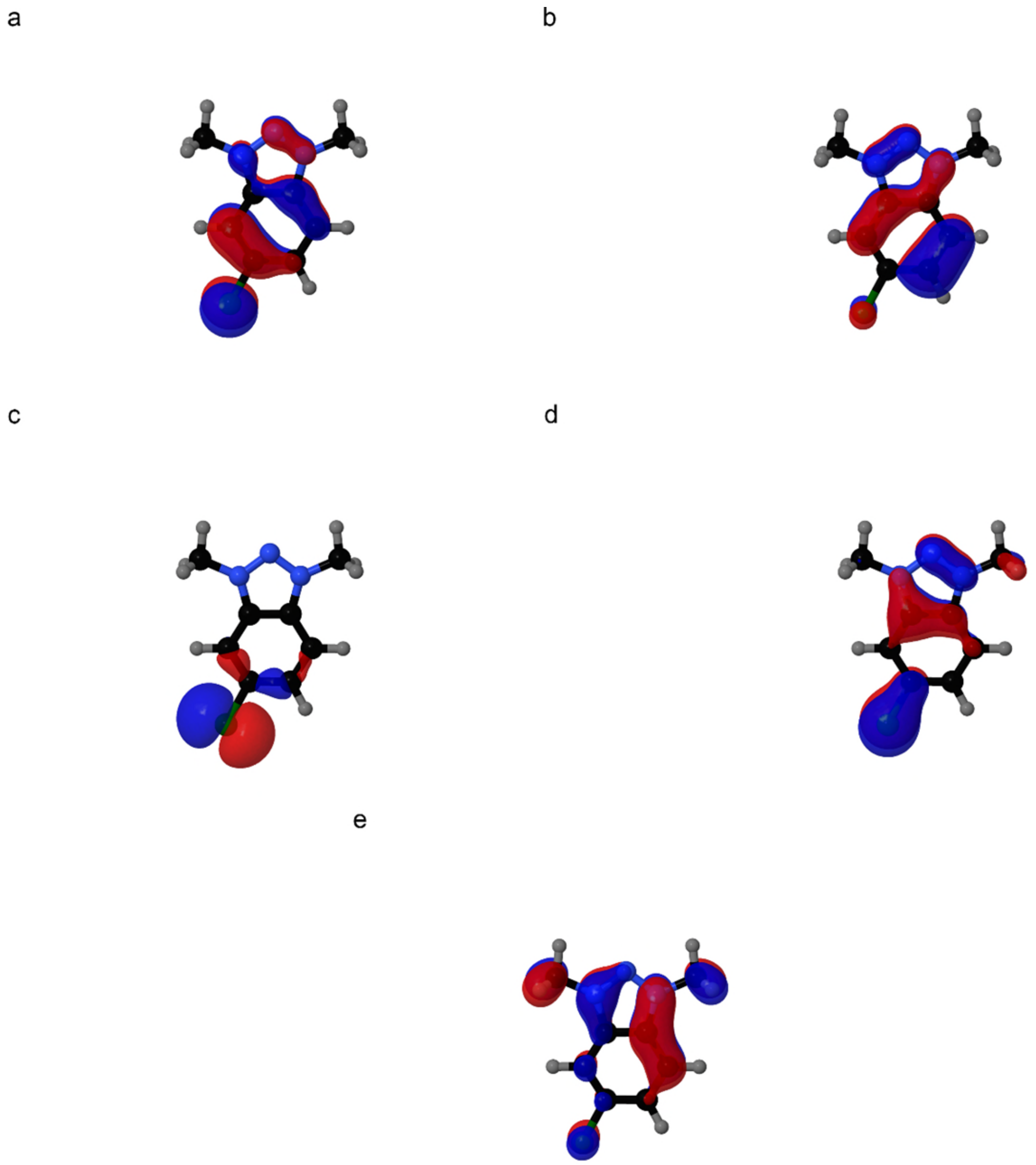

Figure S70. (a) HOMO, (b) HOMO–1, (c) HOMO–2, (d) HOMO–3, and (e) HOMO–4 of [**2**]$^+$. Canonical molecular orbitals were calculated at the B3LYP/def2-TZVP level of theory (isosurface value = 0.04).

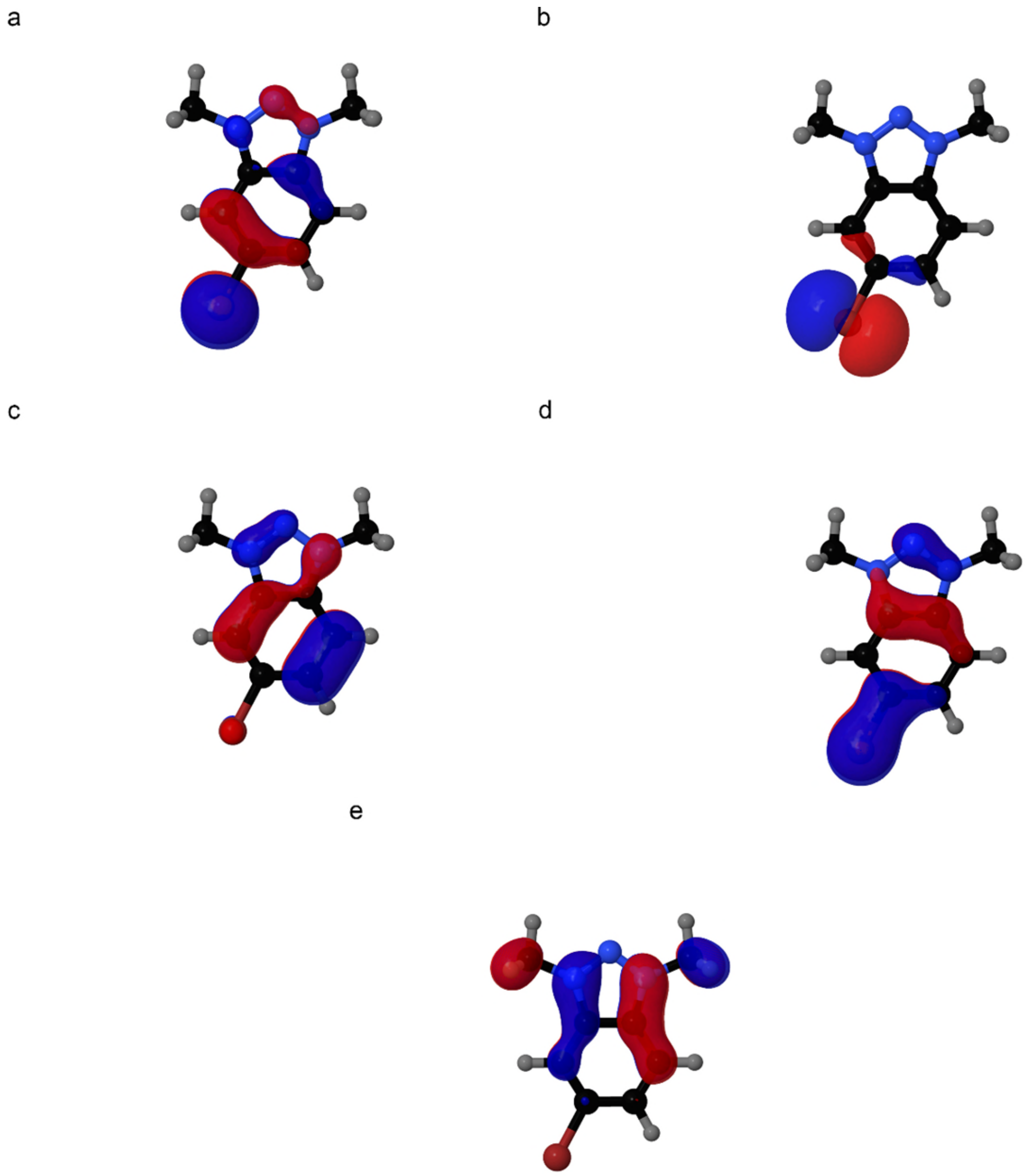

Figure S71. (a) HOMO, (b) HOMO–1, (c) HOMO–2, (d) HOMO–3, and (e) HOMO–4 of [**3**]$^{+}$. Canonical molecular orbitals were calculated at the B3LYP/def2-TZVP level of theory (isosurface value = 0.04).

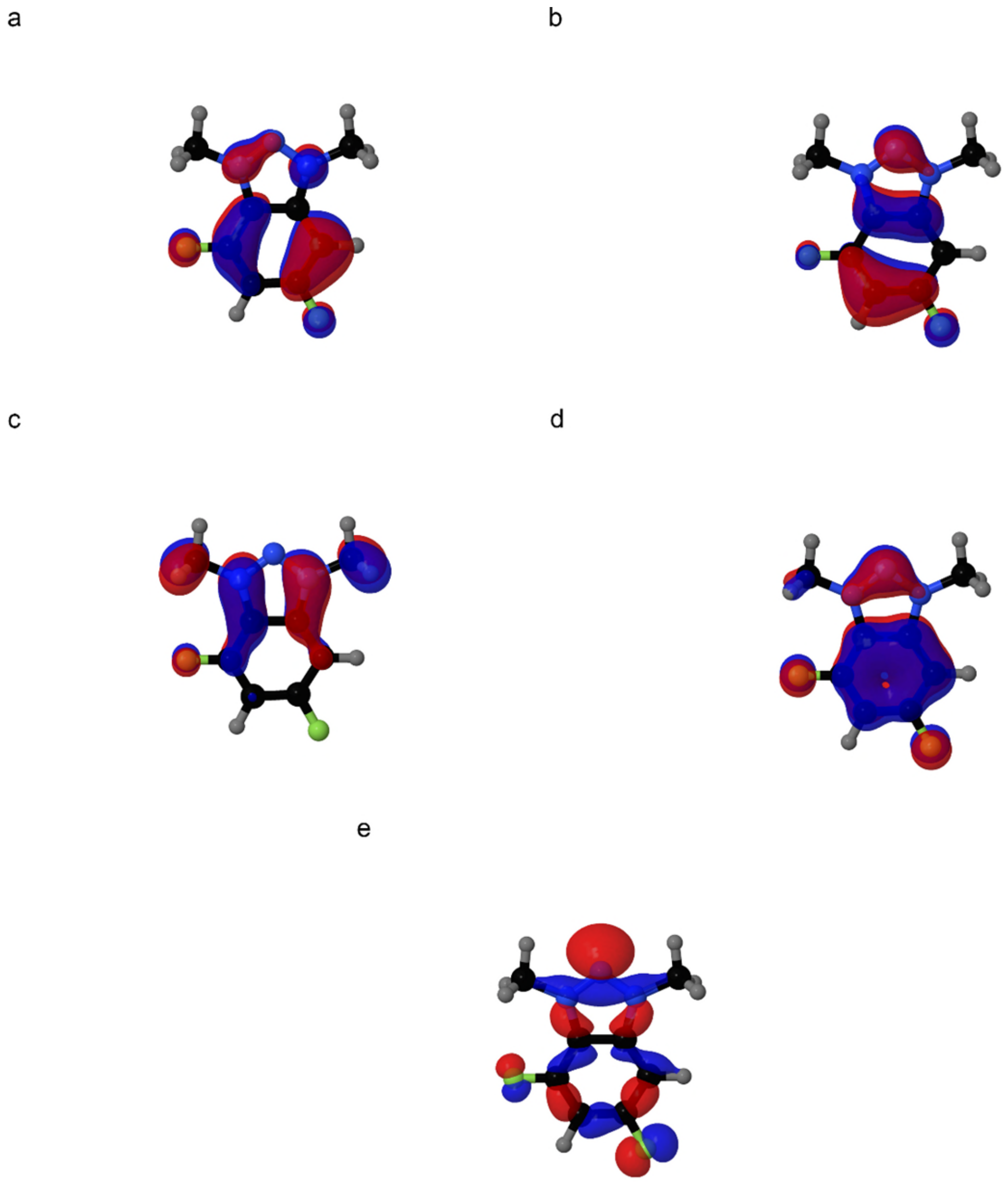

Figure S72. (a) HOMO, (b) HOMO–1, (c) HOMO–2, (d) HOMO–3, and (e) HOMO–4 of [**4**]$^+$. Canonical molecular orbitals were calculated at the B3LYP/def2-TZVP level of theory (isosurface value = 0.04).

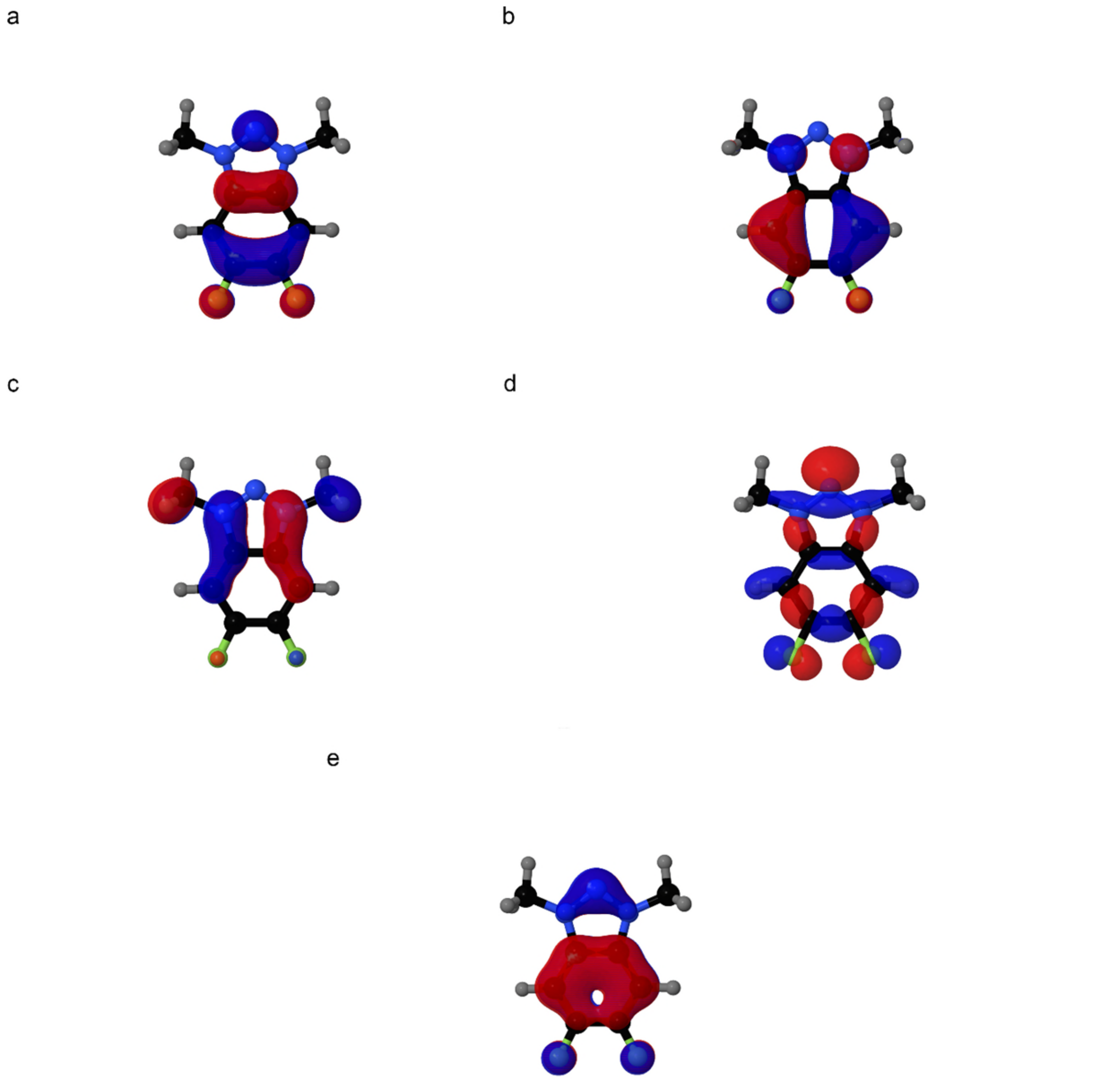

Figure S73. (a) HOMO, (b) HOMO–1, (c) HOMO–2, (d) HOMO–3, and (e) HOMO–4 of [**5**]$^{+}$. Canonical molecular orbitals were calculated at the B3LYP/def2-TZVP level of theory (isosurface value = 0.04).

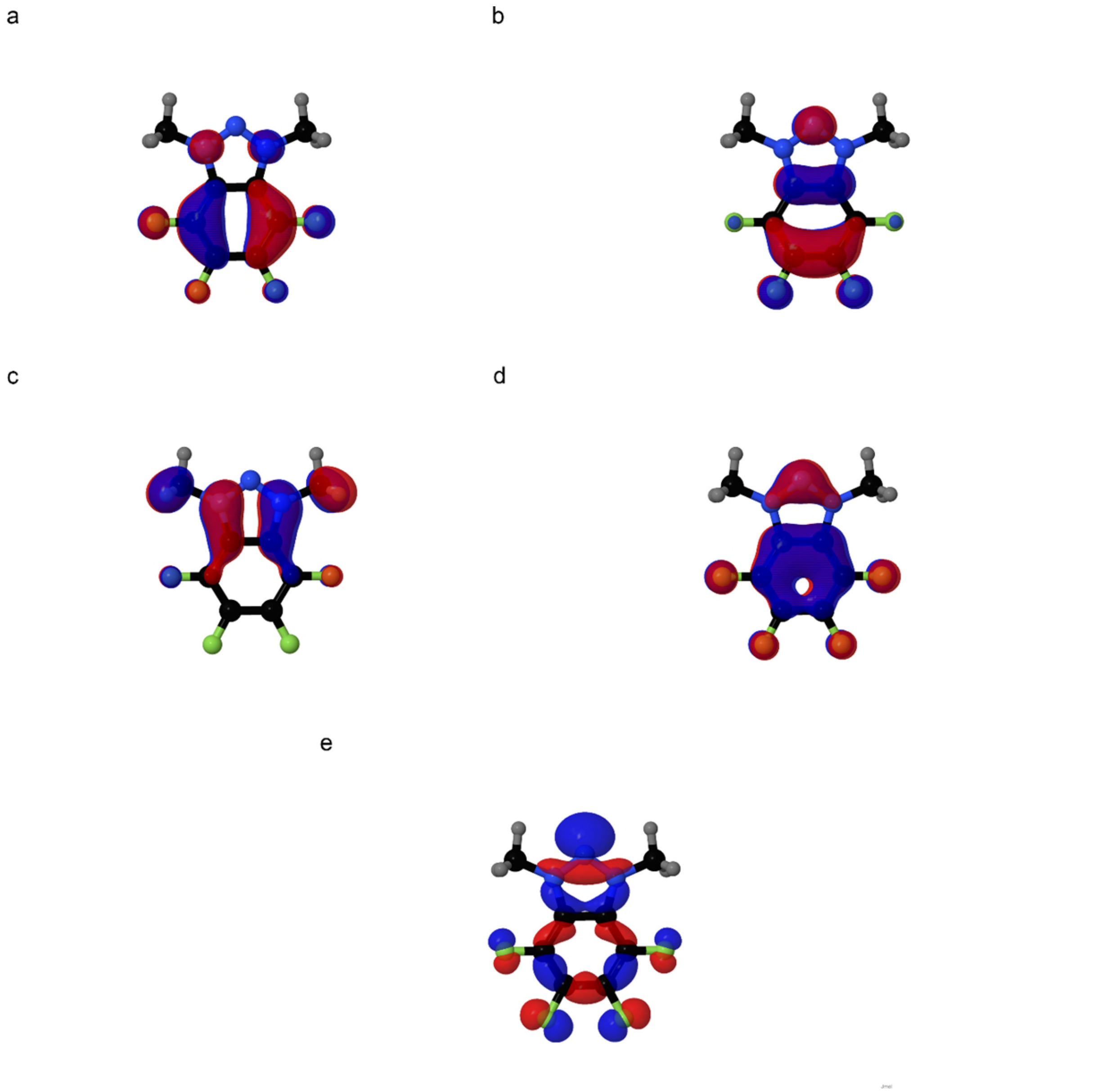

Figure S74. (a) HOMO, (b) HOMO–1, (c) HOMO–2, (d) HOMO–3, and (e) HOMO–4 of [**6**]$^{+}$. Canonical molecular orbitals were calculated at the B3LYP/def2-TZVP level of theory (isosurface value = 0.04).

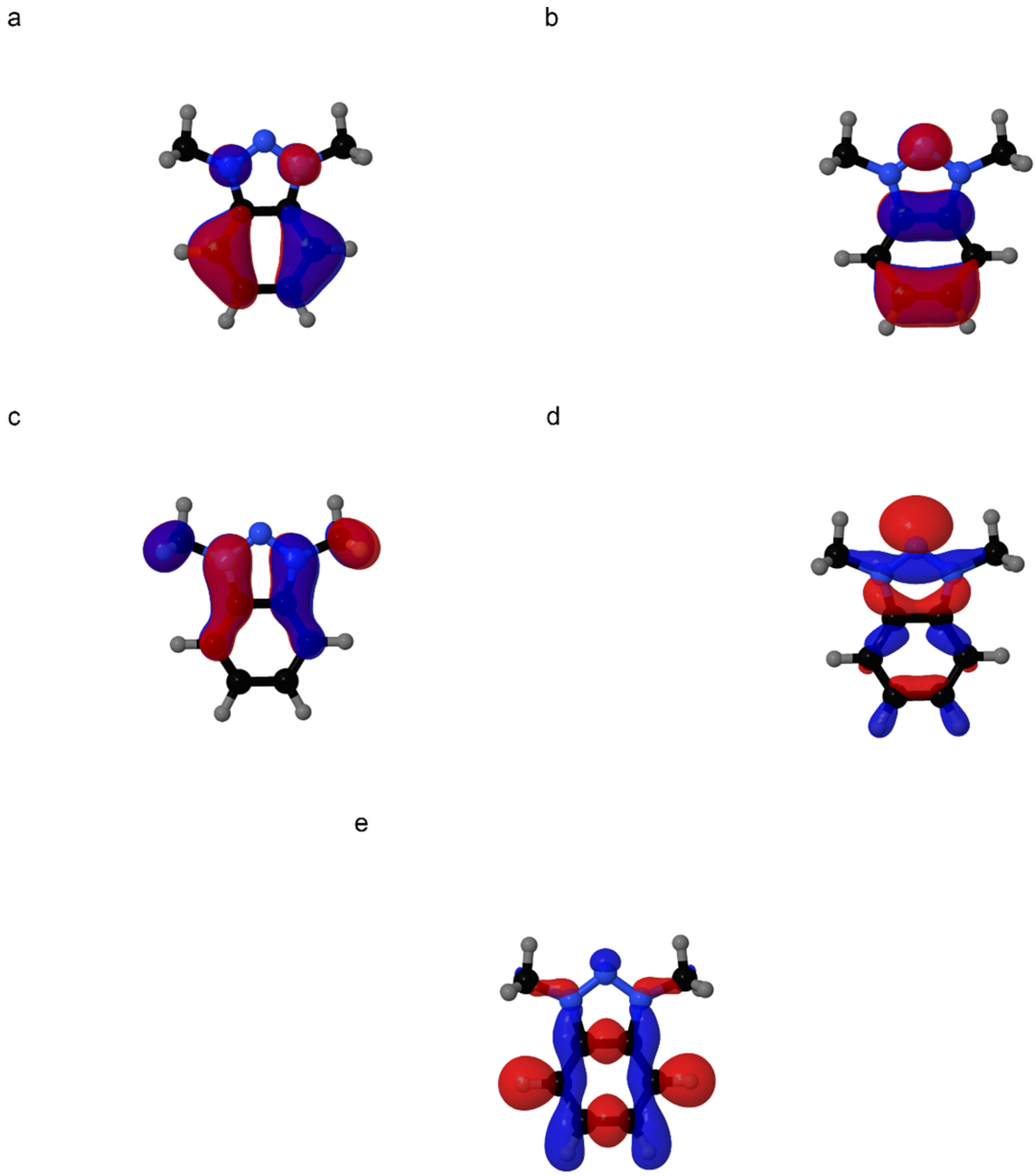

Figure S75. (a) HOMO, (b) HOMO–1, (c) HOMO–2, (d) HOMO–3, and (e) HOMO–4 of [**A**]$^+$. Canonical molecular orbitals were calculated at the B3LYP/def2-TZVP level of theory (isosurface value = 0.04).

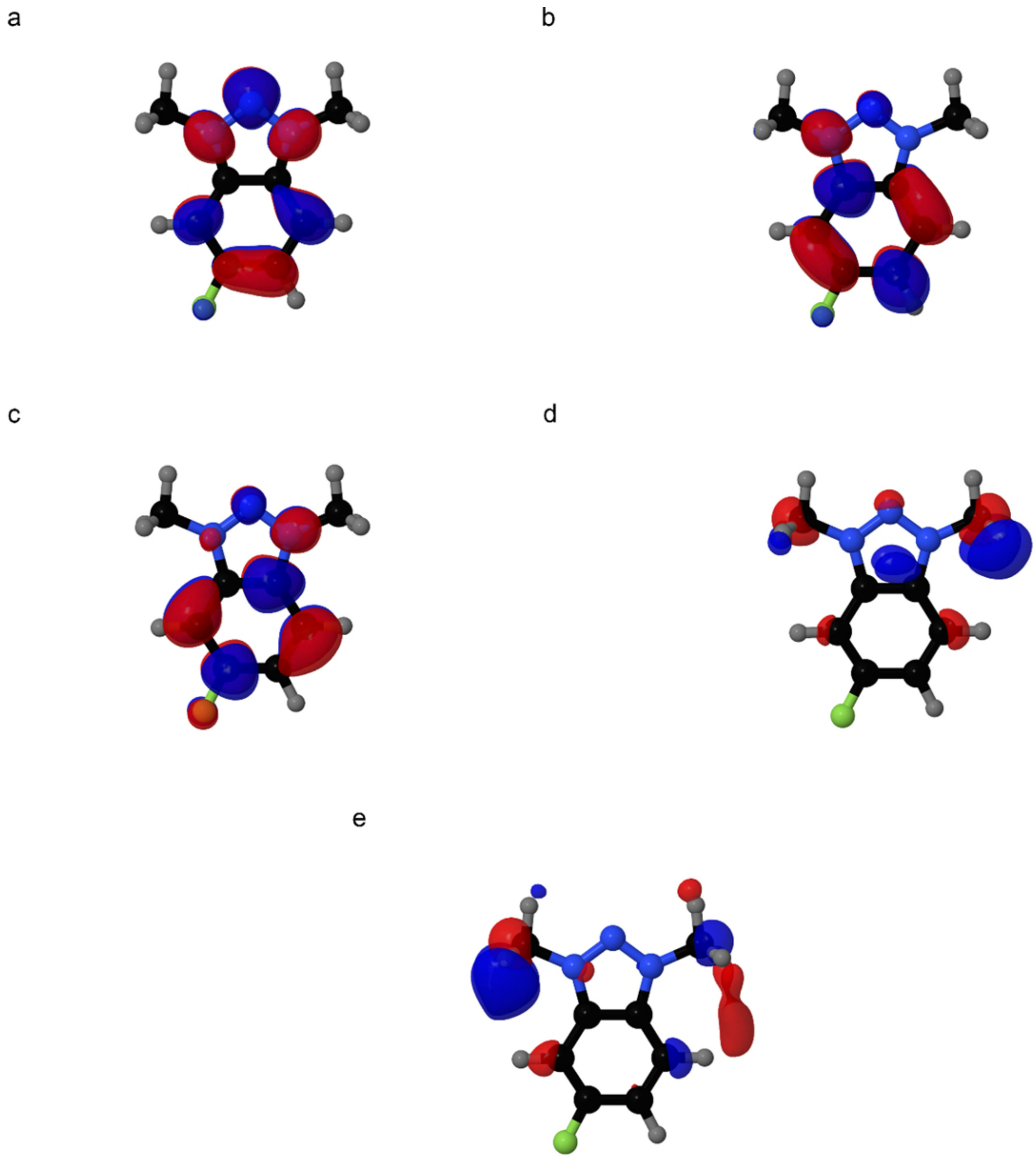

Figure S76. (a) LUMO, (b) LUMO+1, (c) LUMO+2, (d) LUMO+3, and (e) LUMO+4 of [**1**]$^{+}$. Canonical molecular orbitals were calculated at the B3LYP/def2-TZVP level of theory (isosurface value = 0.04).

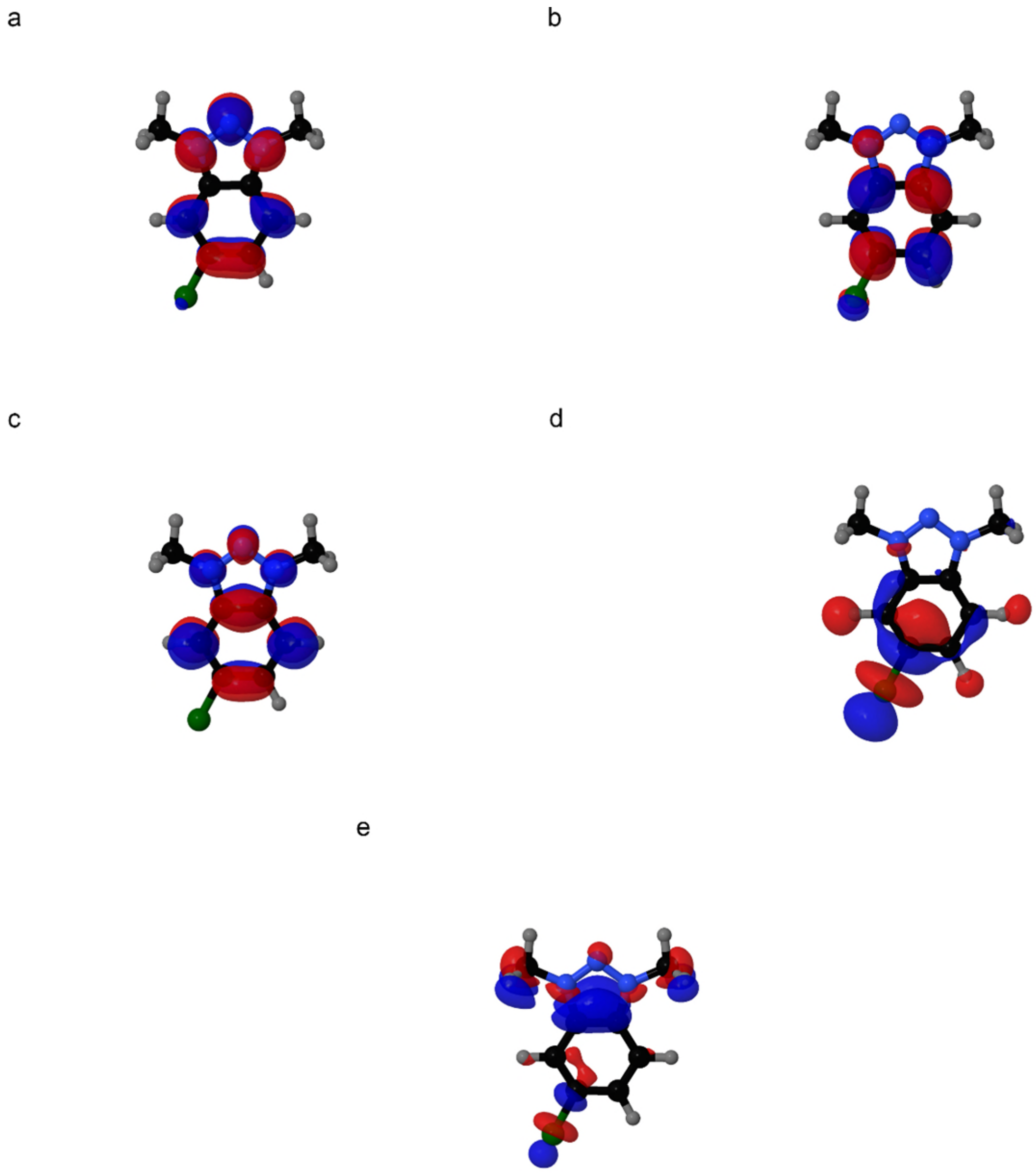

Figure S77. (a) LUMO, (b) LUMO+1, (c) LUMO+2, (d) LUMO+3, and (e) LUMO+4 of [**2**]$^+$. Canonical molecular orbitals were calculated at the B3LYP/def2-TZVP level of theory (isosurface value = 0.04).

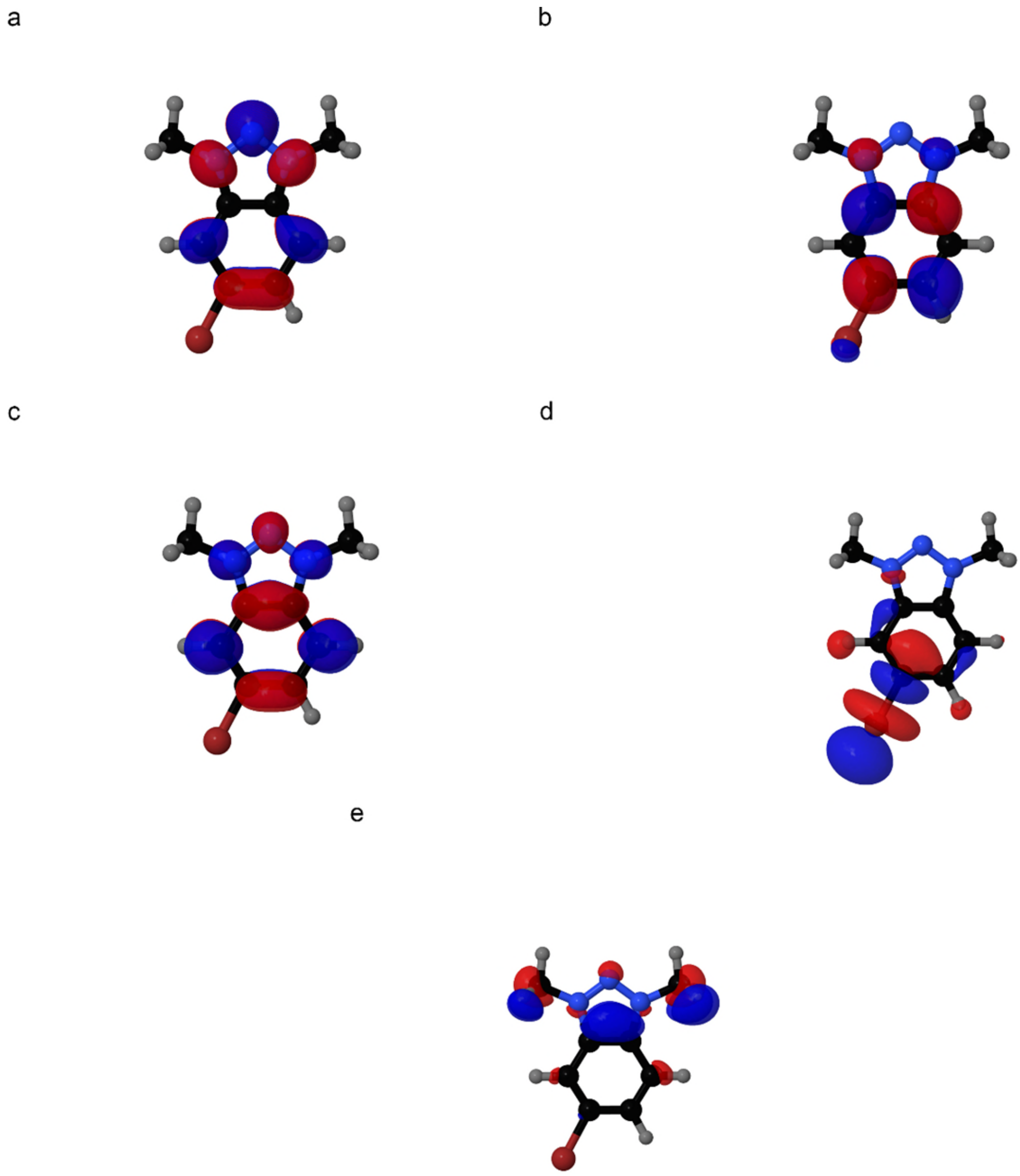

Figure S78. (a) LUMO, (b) LUMO+1, (c) LUMO+2, (d) LUMO+3, and (e) LUMO+4 of [**3**]$^{+}$. Canonical molecular orbitals were calculated at the B3LYP/def2-TZVP level of theory (isosurface value = 0.04).

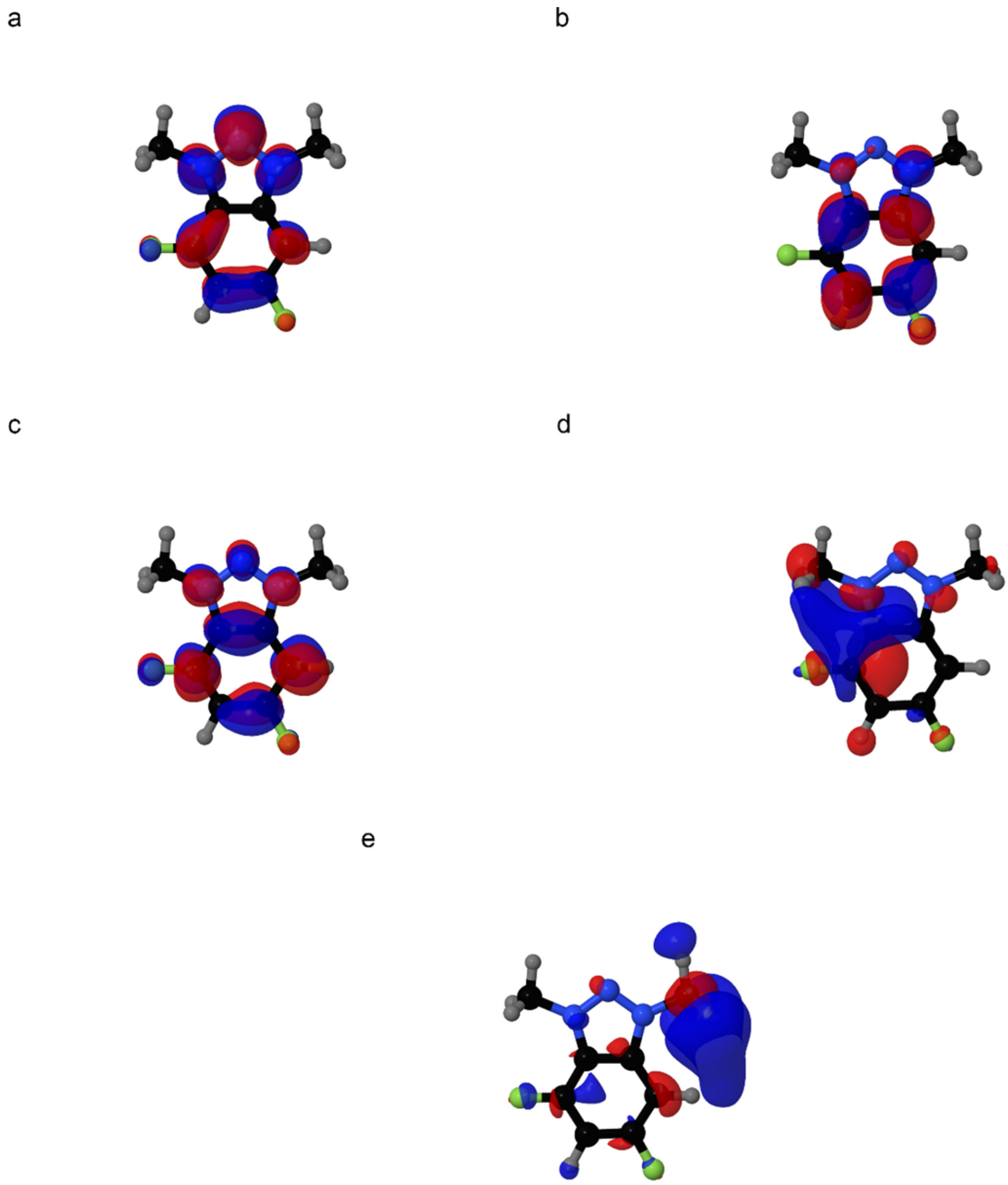

Figure S79. (a) LUMO, (b) LUMO+1, (c) LUMO+2, (d) LUMO+3, and (e) LUMO+4 of [**4**]$^+$. Canonical molecular orbitals were calculated at the B3LYP/def2-TZVP level of theory (isosurface value = 0.04).

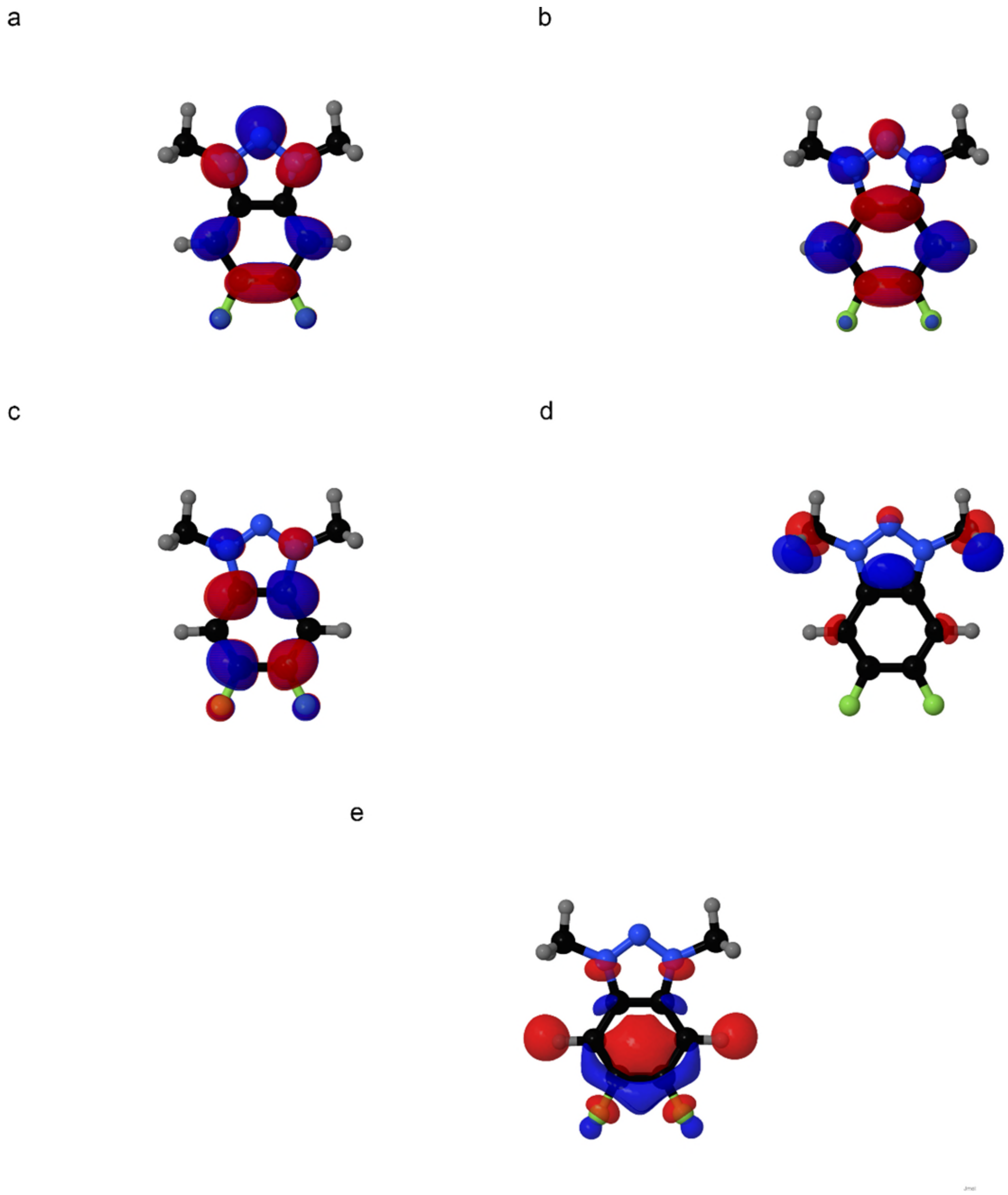

Figure S80. (a) LUMO, (b) LUMO+1, (c) LUMO+2, (d) LUMO+3, and (e) LUMO+4 of [**5**]$^{+}$. Canonical molecular orbitals were calculated at the B3LYP/def2-TZVP level of theory (isosurface value = 0.04).

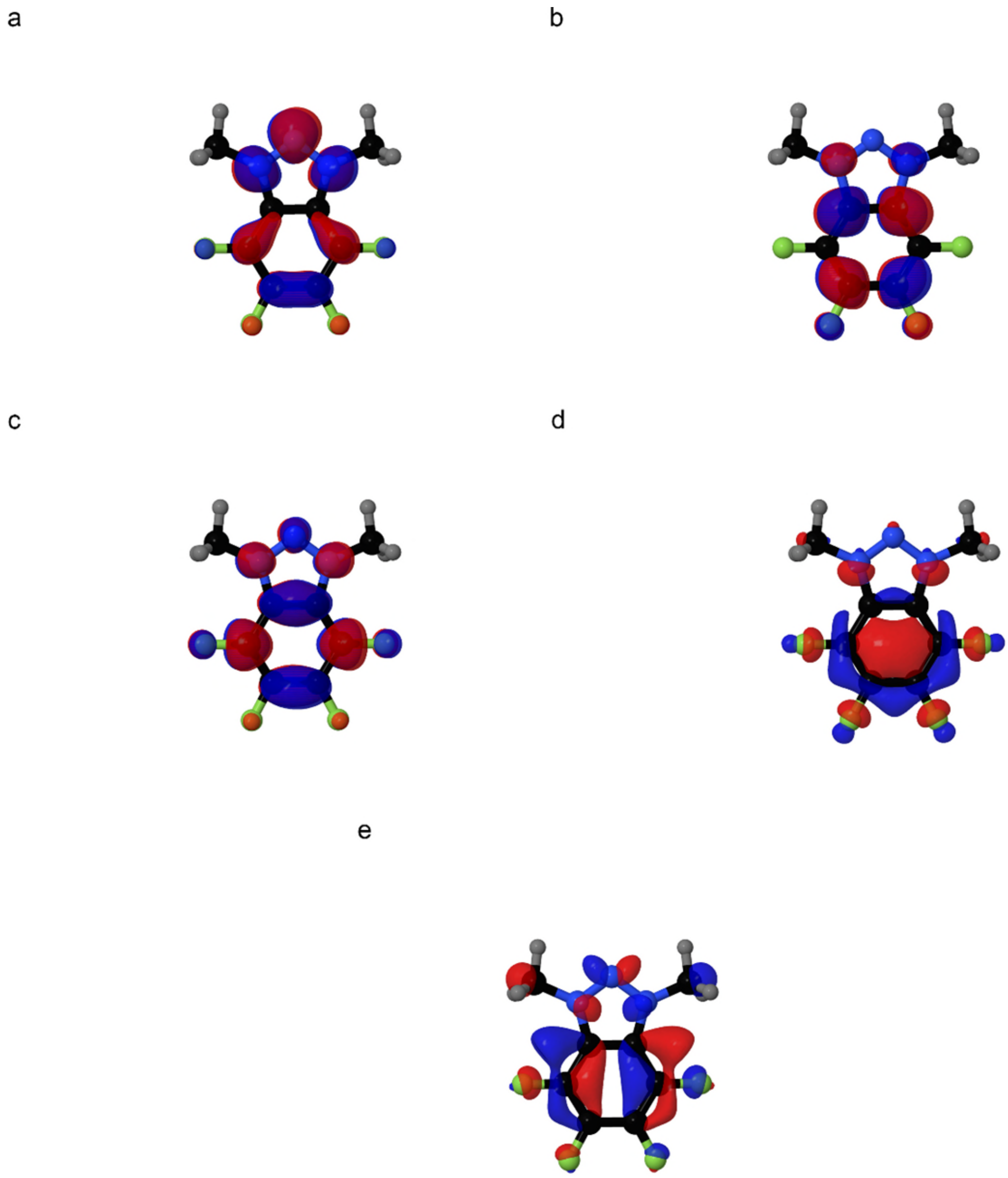

Figure S81. (a) LUMO, (b) LUMO+1, (c) LUMO+2, (d) LUMO+3, and (e) LUMO+4 of [**6**]$^{+}$. Canonical molecular orbitals were calculated at the B3LYP/def2-TZVP level of theory (isosurface value = 0.04).

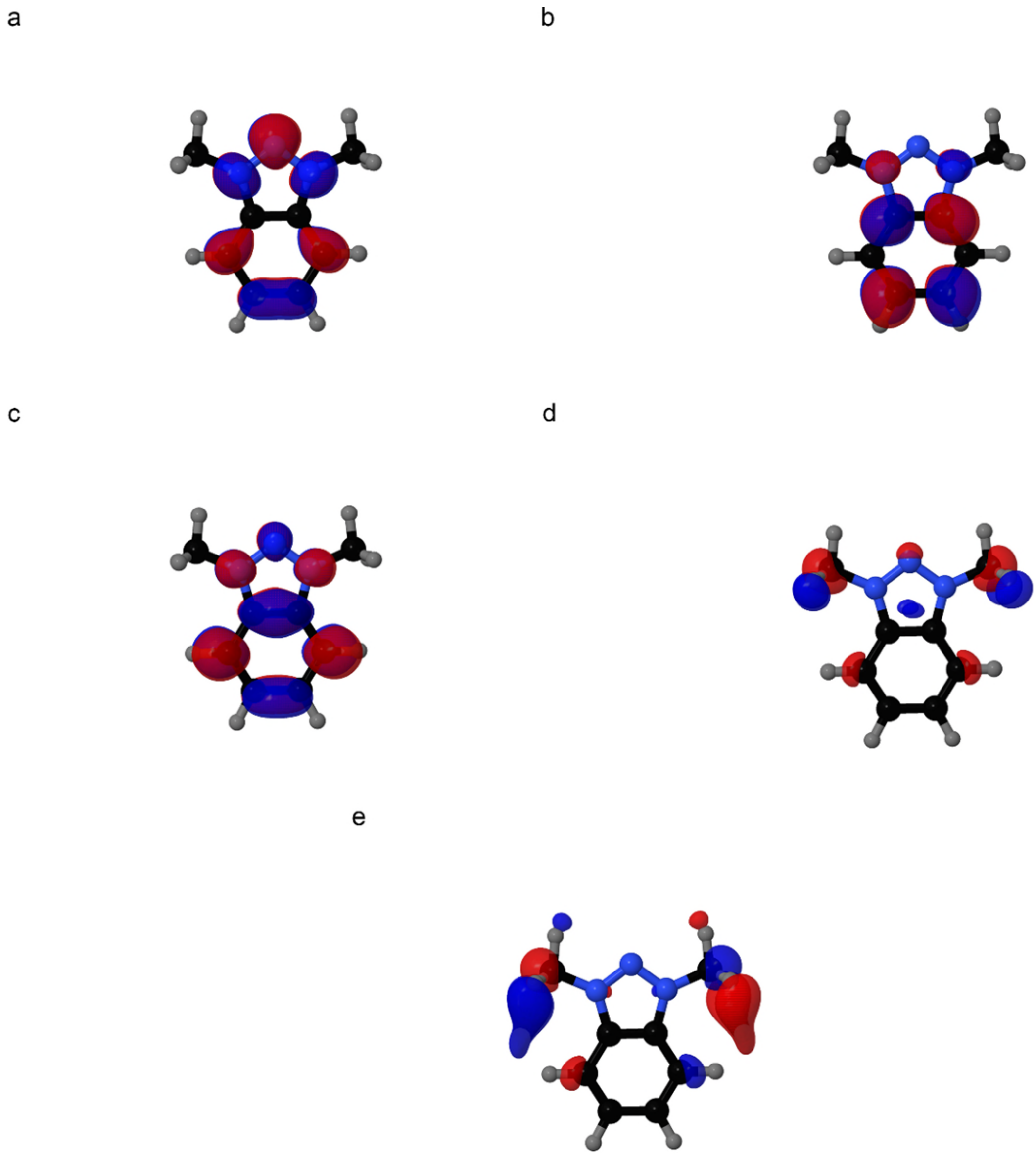

Figure S82. (a) LUMO, (b) LUMO+1, (c) LUMO+2, (d) LUMO+3, and (e) LUMO+4 of [**A**]$^+$. Canonical molecular orbitals were calculated at the B3LYP/def2-TZVP level of theory (isosurface value = 0.04).

3.2 Fluoride Ion Affinity

The Lewis acidity of selected Lewis acids was probed by calculation of their fluoride ion affinity (FIA; B3LYP/def2-TZVP) using the CF_2O-CF_3O^- reference system (also known as Christe's method).[10]

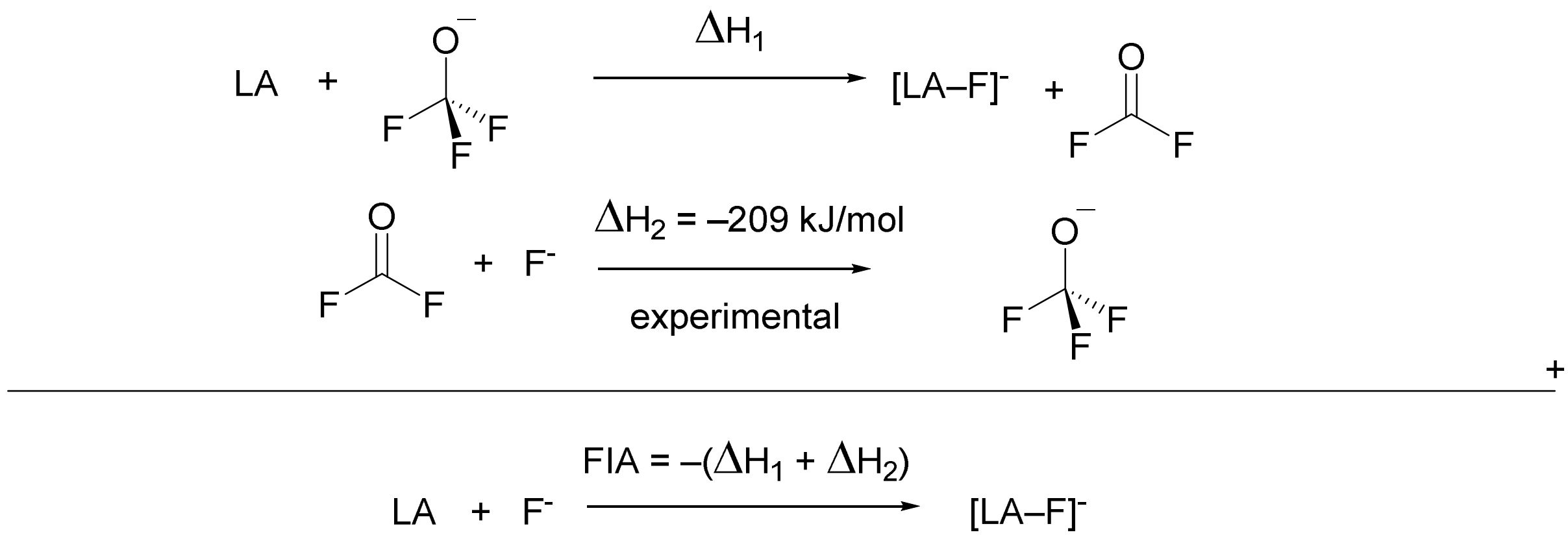

Table S8. FIA (kJ/mol) of [**1**-**6**]$^+$ (B3LYP/def2-TZVP).

**Compound**	**FIA (kJ mol^{-1})** [a]
[**A**]$^+$	**430**
[**1**]$^+$	437
[**2**]$^+$	437
[**3**]$^+$	437
[**4**]$^+$	449
[**5**]$^+$	446
[**6**]$^+$	469

[a] DFT was conducted on the naked cation.

3.3 Hydride Ion Affinity

The Lewis acidity of the Lewis acids was also probed by calculating hydride ion affinity, using the Me_3SiH / Me_3Si^+ reference system.[11]

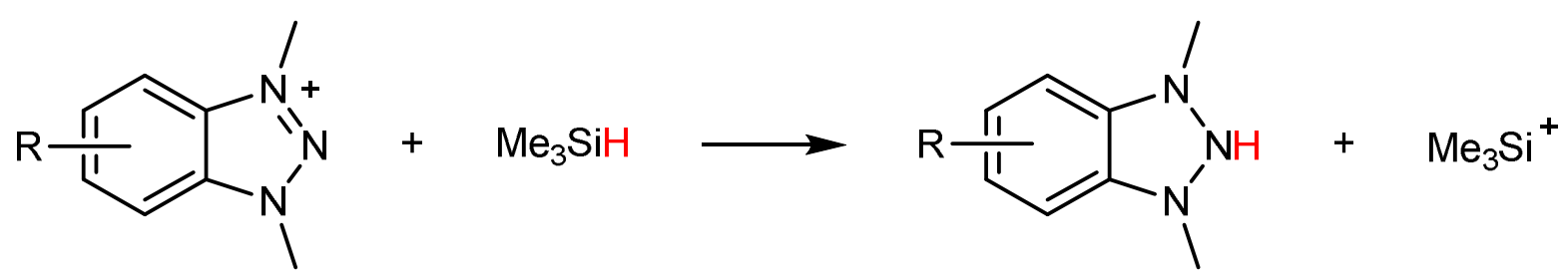

Table S9. HIA (kJ/mol) of [**1**]$^+$-[**6**]$^+$ (B3LYP/def2-TZVP).

**Compound**	**HIA (kJ mol^{-1})**
[**A**]$^+$	645
[**1**]$^+$	672
[**2**]$^+$	674
[**3**]$^+$	674
[**4**]$^+$	692

[**5**]$^+$	684
[**6**]$^+$	722

3.4 Binding Towards Neutral Donors

The coordination of the series towards neutral donors was also studied. Electronic binding energies for the binding with the carbene 1,3-dimethylimidazol-2-ylidene (IMe) and trimethylphosphine (PMe_3) were calculated in the gas phase.

Table S10: Electronic binding energies of [**A**]$^+$, [**1**]$^+$-[**6**]$^+$ with IMe in the gas phase (B3LYP/def2-TZVP).

Adduct	ΔE (Hartree)	ΔE (kcal/mol)	ΔE (kJ/mol)
[**A**]$^+$–IMe	+0.002404	+1.51	+6.31
[**1**]$^+$–IMe	+0.000207	+0.13	+0.54
[**2**]$^+$–IMe	+0.000267	+0.17	+0.70
[**3**]$^+$–IMe	+0.000348	+0.22	+0.91
[**4**]$^+$–IMe	–0.004256	–2.67	–11.17
[**5**]$^+$–IMe	−0.000659	−0.41	−1.73
[**6**]$^+$–IMe	−0.009939	−6.24	−26.10

Table S11. Electronic binding energies of [**A**]$^+$, [**1**]$^+$-[**6**]$^+$ with PMe_3 in the gas phase (B3LYP/def2-TZVP).

Adduct	ΔE (Hartree)	ΔE (kcal/mol)	ΔE (kJ/mol)
[**A**]$^+$–PMe_3	−0.053446751	−33.54	−140.34
[**1**]$^+$–PMe_3	–0.054996444	–34.51	–144.39
[**2**]$^+$–PMe_3	–0.055166481	–34.62	–144.84
[**3**]$^+$–PMe_3	–0.055182235	–34.63	–144.88
[**4**]$^+$–PMe_3	–0.059561489	–37.38	–156.38
[**5**]$^+$–PMe_3	–0.056086175	–35.19	–147.25
[**6**]$^+$–PMe_3	–0.065793730	–41.29	–172.74

Table S12. Energy decomposition analysis of adducts of [**A**]$^+$, [**1**]$^+$-[**6**]$^+$ with OH_2 and NH_3 (B3LYP/def2-TZVP). Values are provided in kcal/mol. ΔE_{disp} = 0 in all cases.

Adduct	ΔE_{int}	ΔE_{orb}	ΔE_{elstat}	ΔE_{Pauli}	ΔE^0_{XC}
[**A**]$^+$–OH_2	–6.79	–1.17	–8.34	5.36	–2.68
[**1**]$^+$–OH_2	–7.04	–1.19	–8.51	5.21	–2.61
[**2**]$^+$–OH_2	–6.96	–1.19	–8.43	5.23	–2.62
[**3**]$^+$–OH_2	–6.95	–1.19	–8.41	5.24	–2.62
[**4**]$^+$–OH_2	–7.43	–1.27	–8.91	5.41	–2.70

[**5**]$^+$–OH_2	–7.26	–1.23	–8.68	5.22	–2.61
[**6**]$^+$–OH_2	–8.00	–1.39	–9.43	5.54	–2.76
[**A**]$^+$–NH_3	–6.05	–1.32	–7.60	5.74	–2.78
[**1**]$^+$–NH_3	–6.19	–1.38	–7.84	5.77	–2.80
[**2**]$^+$–NH_3	–6.11	–1.38	–7.77	5.83	–2.83
[**3**]$^+$–NH_3	–6.11	–1.39	–7.77	5.85	–2.84
[**4**]$^+$–NH_3	–6.62	–1.47	–8.25	5.90	–2.85
[**5**]$^+$–NH_3	–6.44	–1.43	–8.02	5.80	–2.81
[**6**]$^+$–NH_3	–7.33	–1.65	–8.98	6.32	–3.05

3.5 Singlet – Triplet Gaps

Table S13. Singlet-Triplet gap of [**A**]$^+$, [**1**]$^+$-[**6**]$^+$ calculated using SCF energies (B3LYP/def2-TZVP).

Compound	E_T - E_S (kcal/mol)	E_T - E_S (kJ/mol)
[**A**]$^+$	66.32	277.46
[**1**]$^+$	65.09	272.35
[**2**]$^+$	62.53	261.61
[**3**]$^+$	61.47	257.18
[**4**]$^+$	62.82	262.84
[**5**]$^+$	67.72	283.33
[**6**]$^+$	60.72	254.04

3.6 Global Electrophilicity Index

The global electrophilicity index (GEI; B3LYP/def2-TZVP) was calculated for each of the [$BArF_{20}$] salts according to literature procedures.[12]

Table S14. GEI of [**A**][$BArF_{20}$], [**1**][$BArF_{20}$]-[**6**][$BArF_{20}$] (B3LYP/def2-TZVP).

Compound	GEI (eV)
[**1**][$BArF_{20}$]	5.89
[**2**][$BArF_{20}$]	5.70
[**3**][$BArF_{20}$]	5.71
[**4**][$BArF_{20}$]	6.38
[**5**][$BArF_{20}$]	6.47
[**6**][$BArF_{20}$]	7.43
[**A**][$BArF_{20}$]	5.25

3.7 Gutmann-Beckett

The Gutmann-Beckett method was used to experimentally assess the Lewis acidity across the series. This method analyses the difference in chemical shift of Et_3PO in the ^{31}P NMR when each Lewis acid is complexed to it. From this difference in chemical shift, an Acceptor Number (AN) can be calculated using Equation 1, where a higher number indicates a greater Lewis acidity.

In a J Young NMR tube, triethylphosphine oxide (1 mg, 0.007 mmol, 1 equiv.; Et_3PO) was reacted with the Lewis acid (0.022 mmol, 3 equiv.) in CD_2Cl_2 and analysed by $^{31}P\{^1H\}$ NMR spectroscopy. The chemical shift of the resulting phosphorus resonance was input into Equation 1 to calculate AN.

Equation 1. $AN = 2.21 \times (\delta_{P(LA - Et_3PO)} - 41)$

Table S15. AN for [**1**][BArF20], [**2**][BArF20], [**3**][BArF20], [**4**][BArF20], [**5**][BArF20]•(DCM)0.5, [**6**][BArF20], and [**A**][BArF20].

Compound	Δ $^{31}P\{^1H\}$ (ppm)	Acceptor Number
[**1**][BArF20]	9.89	21.86
[**2**][BArF20]	10.35	22.87
[**3**][BArF20]	10.64	23.51
[**4**][BArF20]	10.56	23.34
[**5**][BArF20]•(DCM)0.5	11.55	25.53
[**6**][BArF20][a]	9.73	21.50
[**A**][BArF20]	10.71	23.67

[a] [6][BArF20] is poorly soluble in CD_2Cl_2 with a large amount suspended during the test.

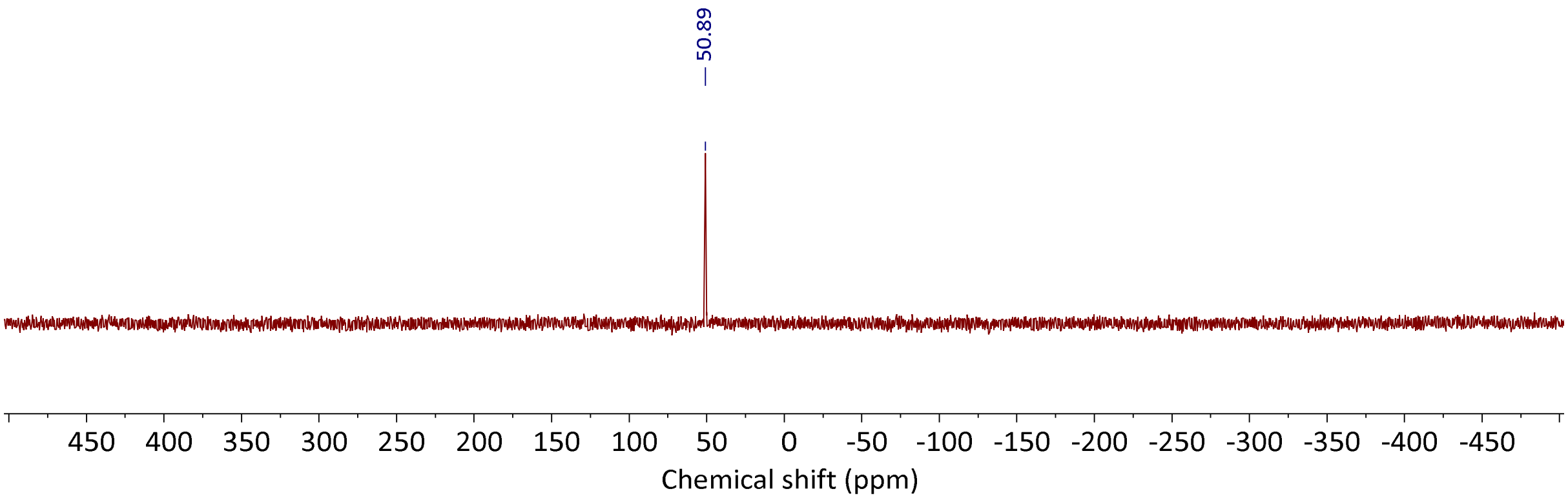

Figure S83. $^{31}P\{^1H\}$ NMR spectrum (CD_2Cl_2) of the Gutmann-Beckett test for [**1**][BArF20].

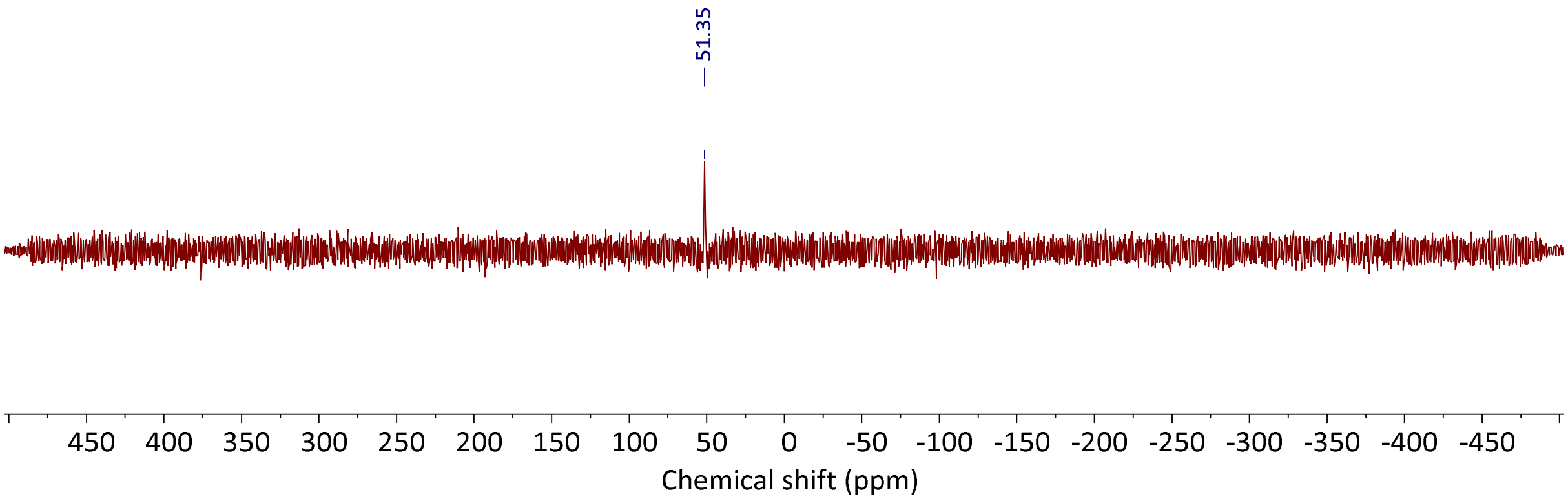

Figure S84. $^{31}P\{^1H\}$ NMR spectrum (CD_2Cl_2) of the Gutmann-Beckett test for [**2**][$BArF_{20}$].

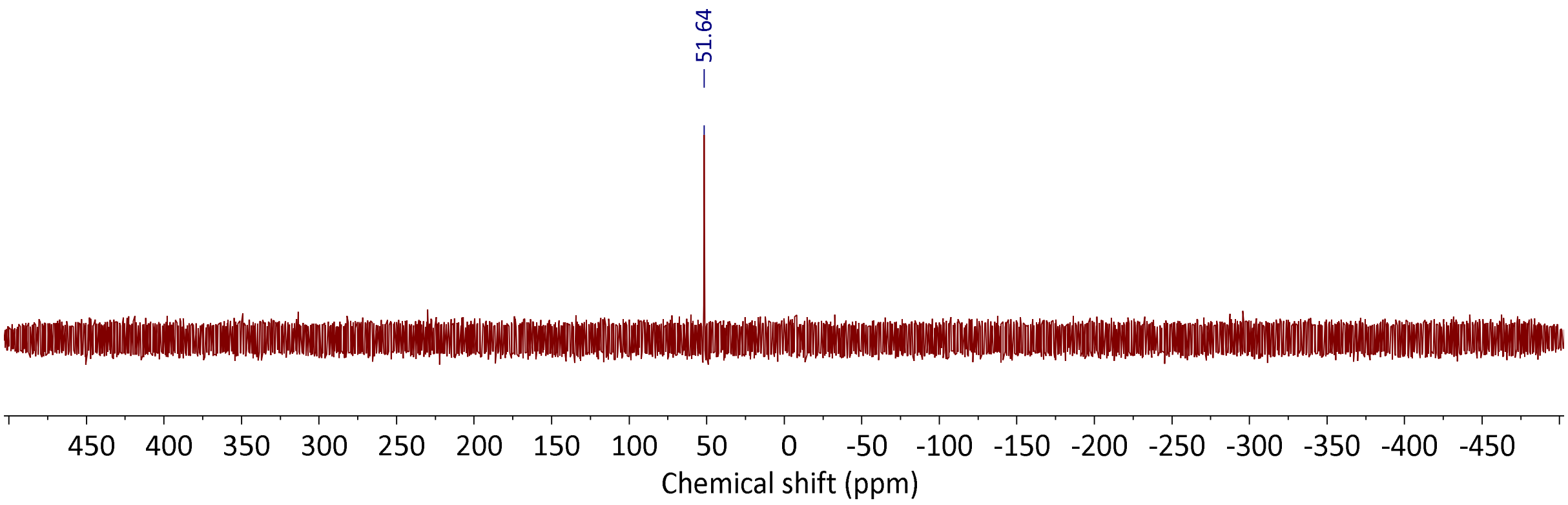

Figure S85. $^{31}P\{^1H\}$ NMR spectrum (CD_2Cl_2) of the Gutmann-Beckett test for [**3**][$BArF_{20}$].

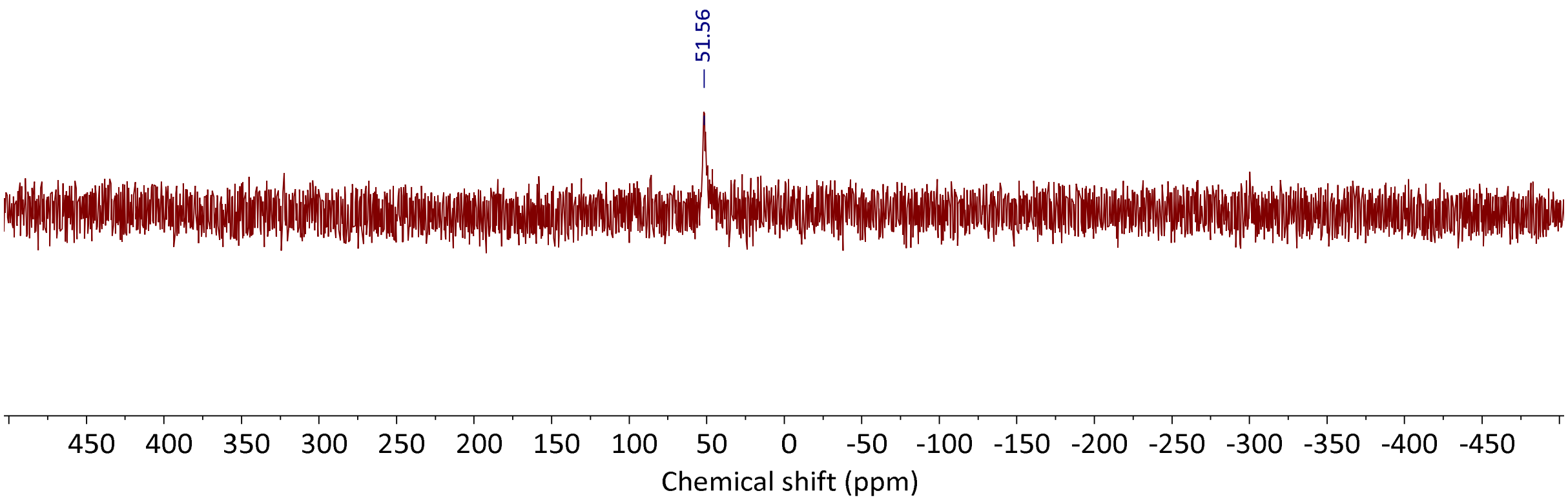

Figure S86. $^{31}P\{^1H\}$ NMR spectrum (CD_2Cl_2) of the Gutmann-Beckett test for [**4**][$BArF_{20}$].

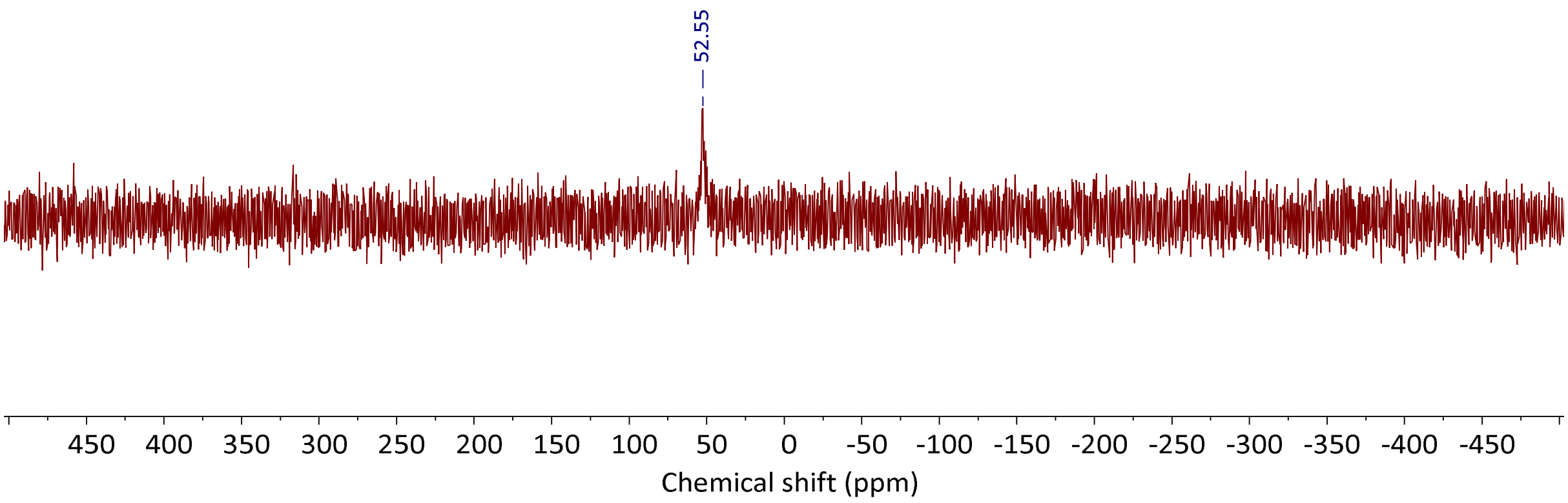

Figure S87. $^{31}P\{^1H\}$ NMR spectrum (CD_2Cl_2) of the Gutmann-Beckett test for [**5**][$BArF_{20}$]•$(DCM)_{0.5}$.

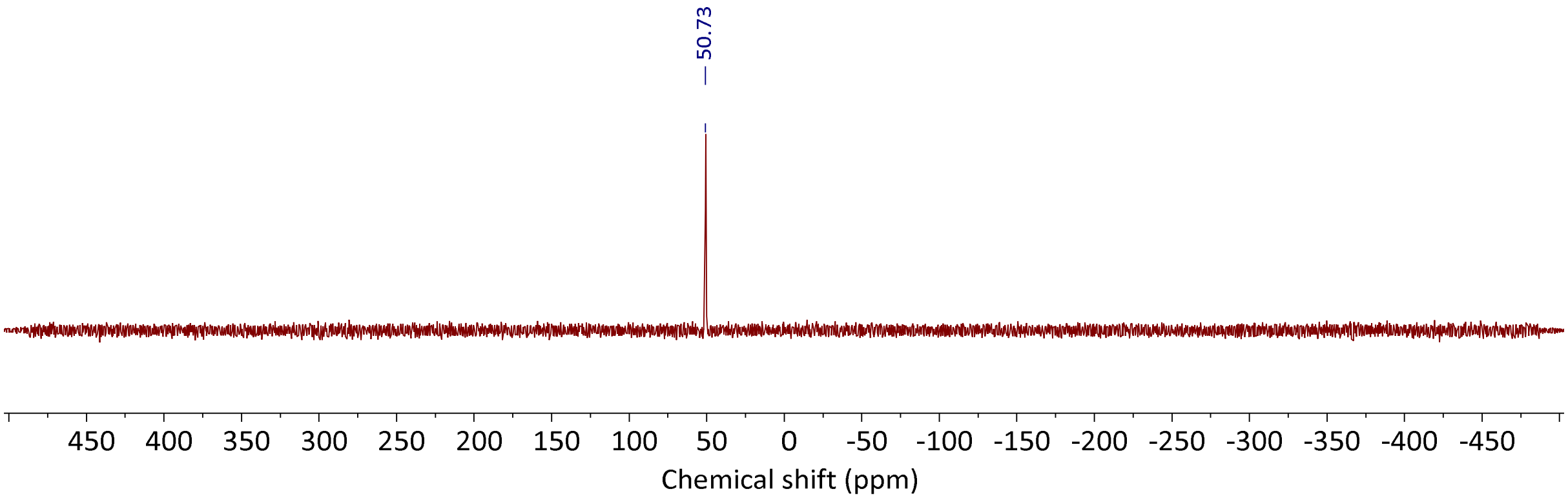

Figure S88. $^{31}P\{^{1}H\}$ NMR spectrum (CD_2Cl_2) of the Gutmann-Beckett test for [**6**][$BArF_{20}$].

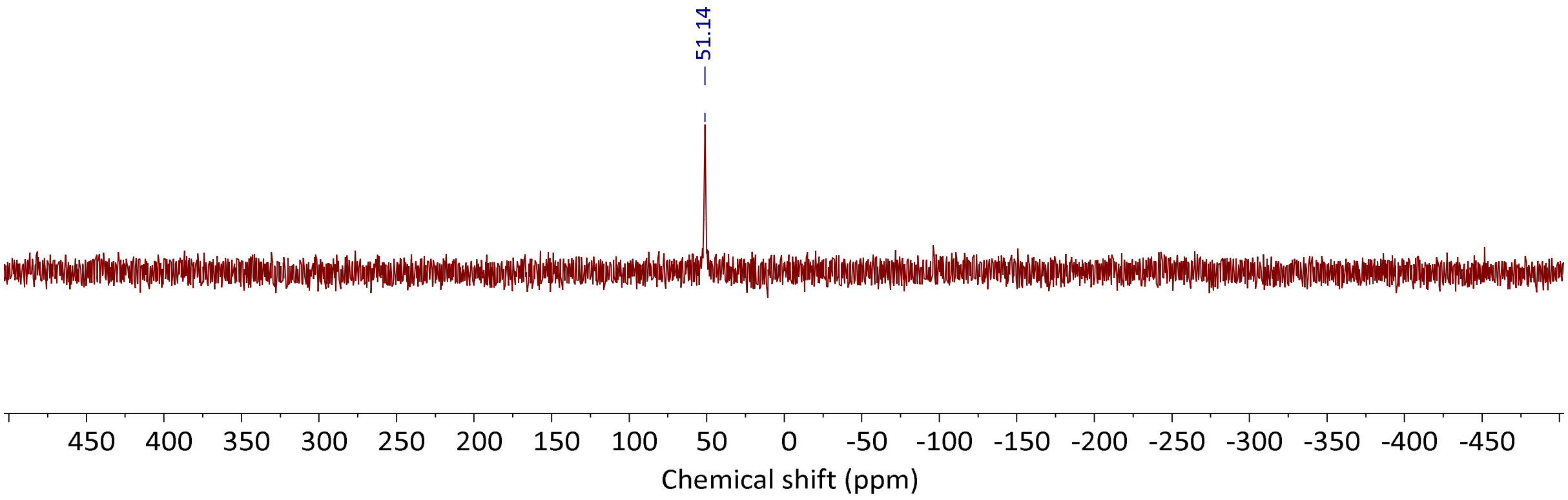

Figure S89. $^{31}P\{^{1}H\}$ NMR spectrum (CD_2Cl_2) of the Gutmann-Beckett test for [**7**][$BArF_{20}$].

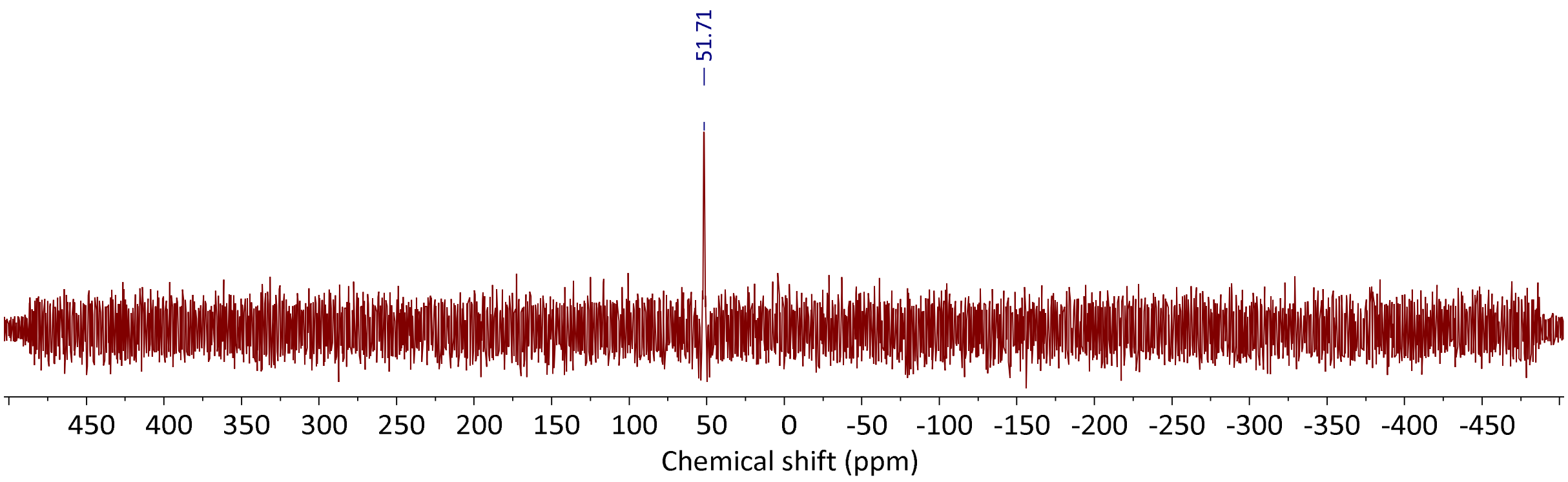

Figure S90. $^{31}P\{^{1}H\}$ NMR spectrum (CD_2Cl_2) of the Gutmann-Beckett test for [**A**][$BArF_{20}$].

3.8 Model catalytic reaction[9]

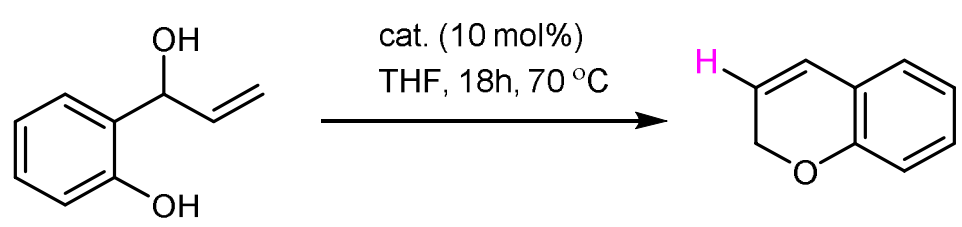

General Procedure for the Lewis Acid-Catalysed Allylic Cyclisation: In a J Young NMR tube 2-(1-hydroxyallyl)phenol (0.05 mmol, 1.0 eq.) and catalyst ([**1**][$BArF_{20}$], [**2**][$BArF_{20}$], [**3**][$BArF_{20}$], [**4**][$BArF_{20}$], [**5**][$BArF_{20}$]•$(DCM)_{0.5}$, or [**6**][$BArF_{20}$]; 10 mol% catalyst loading) were dissolved in THF (0.5 mL) under a

nitrogen atmosphere. The mixture was heated to 70 °C for 18 h. The reaction was cooled to room temperature, the solvent removed at 40 °C, 5 mbar. The residue was extracted with $CDCl_3$ (0.6 mL), filtered, and analysed by ^1H NMR spectroscopy with a 5 µL CH_2Br_2 internal standard. Conversions were determined by the ratio of the 5.77 ppm resonance (shown in pink above) to the internal standard resonance.

Table S16. Yields for the Lewis acid-catalysed allylic cyclisation of 2-(1-hydroxyallyl)phenol.

Catalyst	^1H NMR Yield (%, vs CH_2Br_2 Internal Standard)				
	Run 1	Run 2	Run 3	Average	esd
[**1**][$BArF_{20}$]	3	3.4	3.5	3.3	0.3
[**2**][$BArF_{20}$]	3.7	5.4	5.2	4.8	0.9
[**3**][$BArF_{20}$]	2.4	5.1	3.6	3.7	1.4
[**4**][$BArF_{20}$]	3.5	4.1	3.5	3.7	0.3
[**5**][$BArF_{20}$]•$(DCM)_{0.5}$	3.3	5.0	3.3	3.9	1
[**6**][$BArF_{20}$]	34.2	32.1	25.1	30.5	4.8

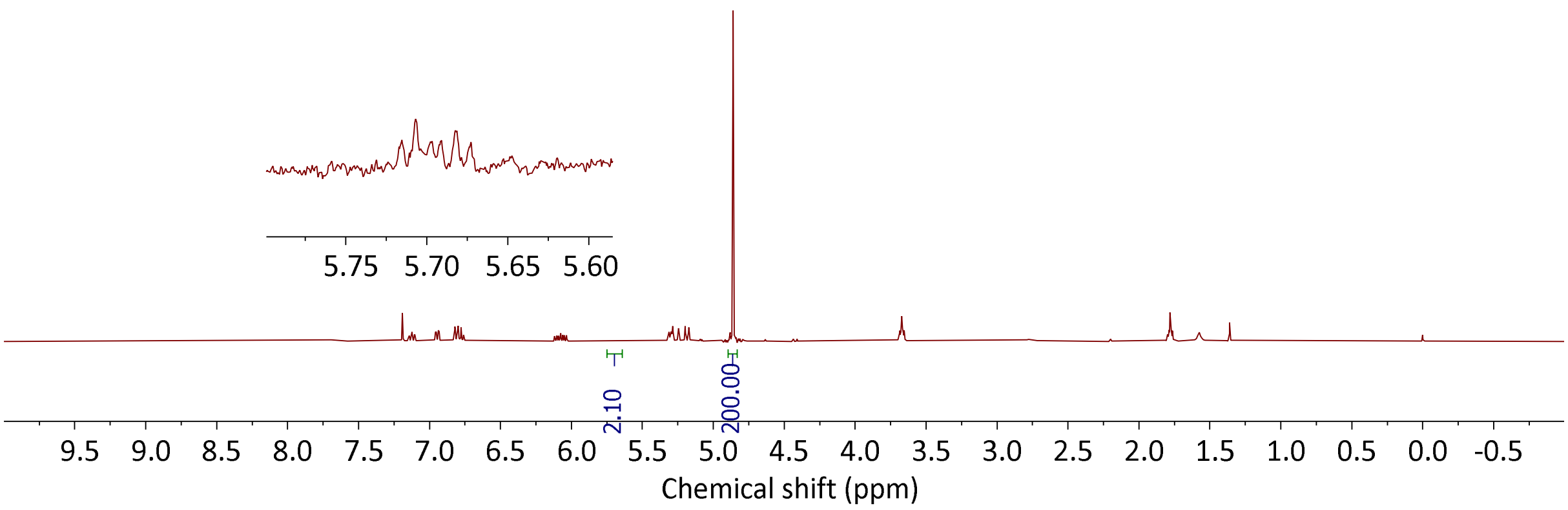

Figure S91. ^1H NMR spectrum of allylic cyclisation with [**1**][$BArF_{20}$] as the catalyst.

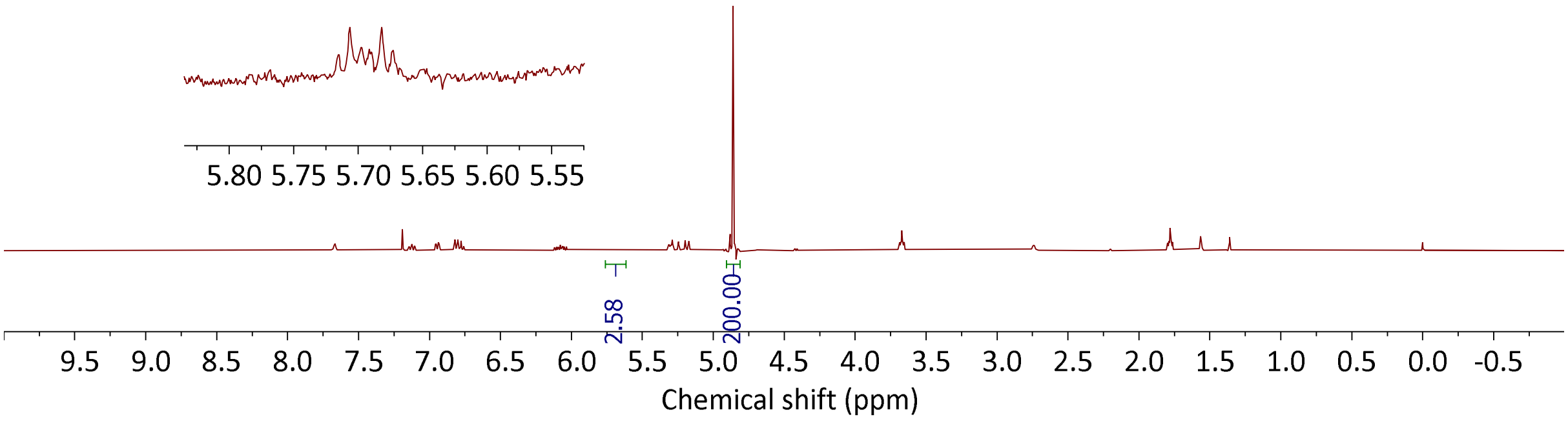

Figure S92. ^{1}H NMR spectrum of allylic cyclisation with [**2**][$BArF_{20}$] as the catalyst.

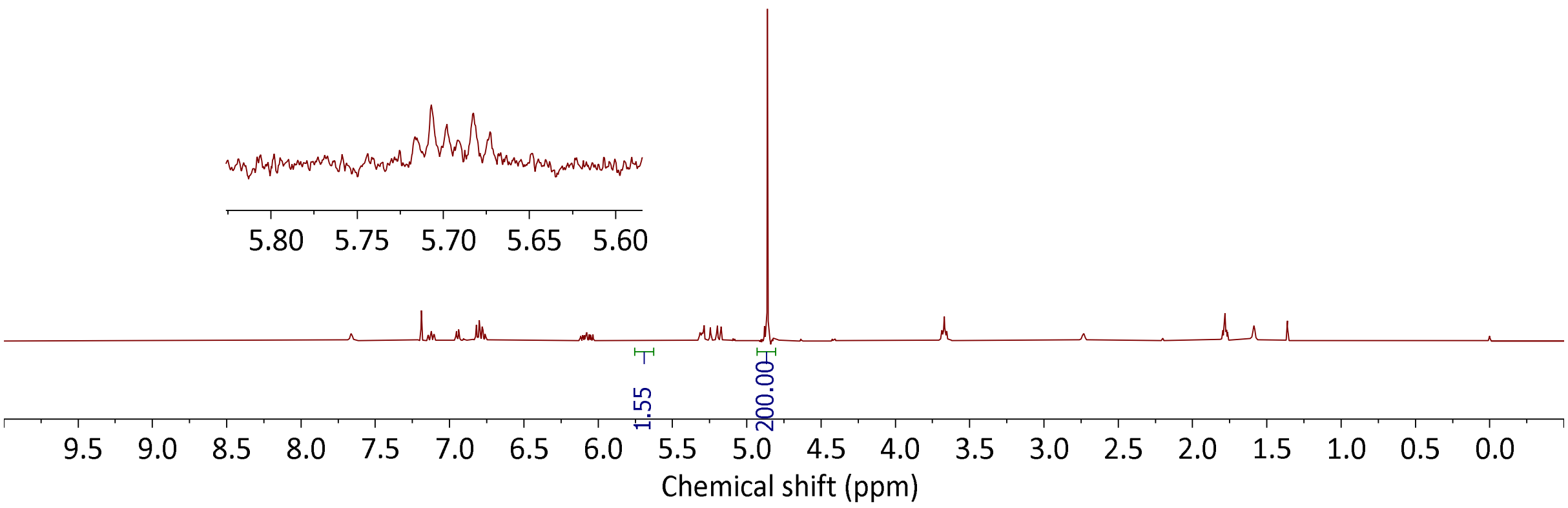

Figure S93. ^{1}H NMR spectrum of allylic cyclisation with [**3**][$BArF_{20}$] as the catalyst.

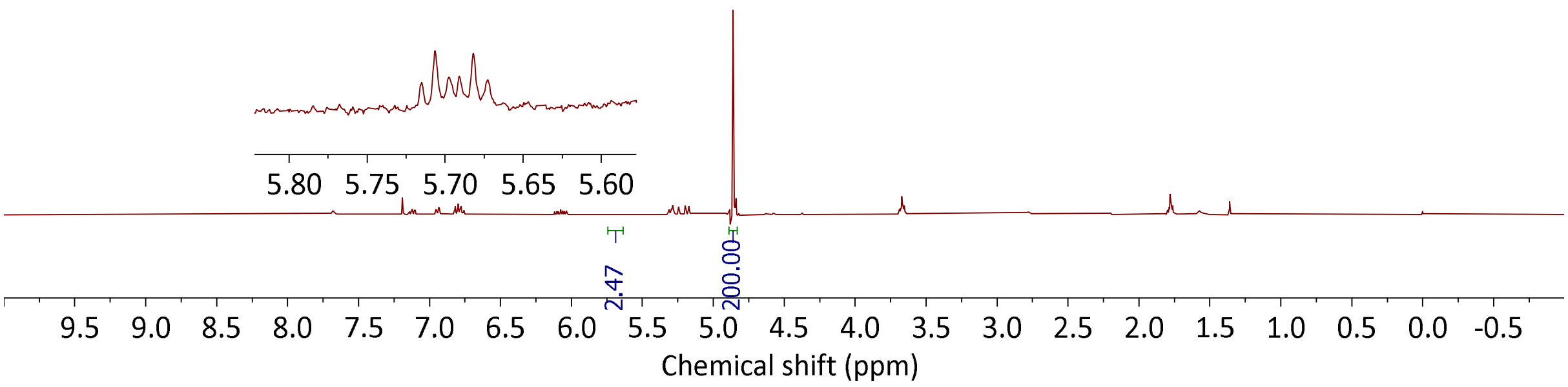

Figure S94. ^{1}H NMR spectrum of allylic cyclisation with [**4**][$BArF_{20}$] as the catalyst.

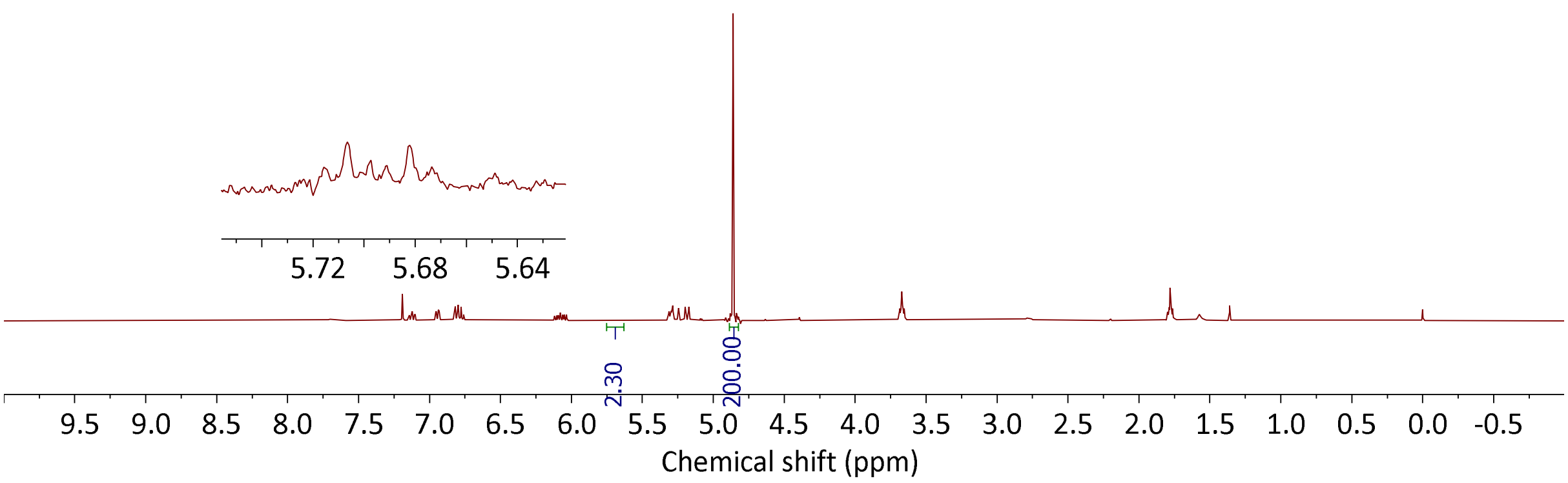

Figure S95. ^{1}H NMR spectrum of allylic cyclisation with [**5**][$BArF_{20}$]•$(DCM)_{0.5}$ as the catalyst.

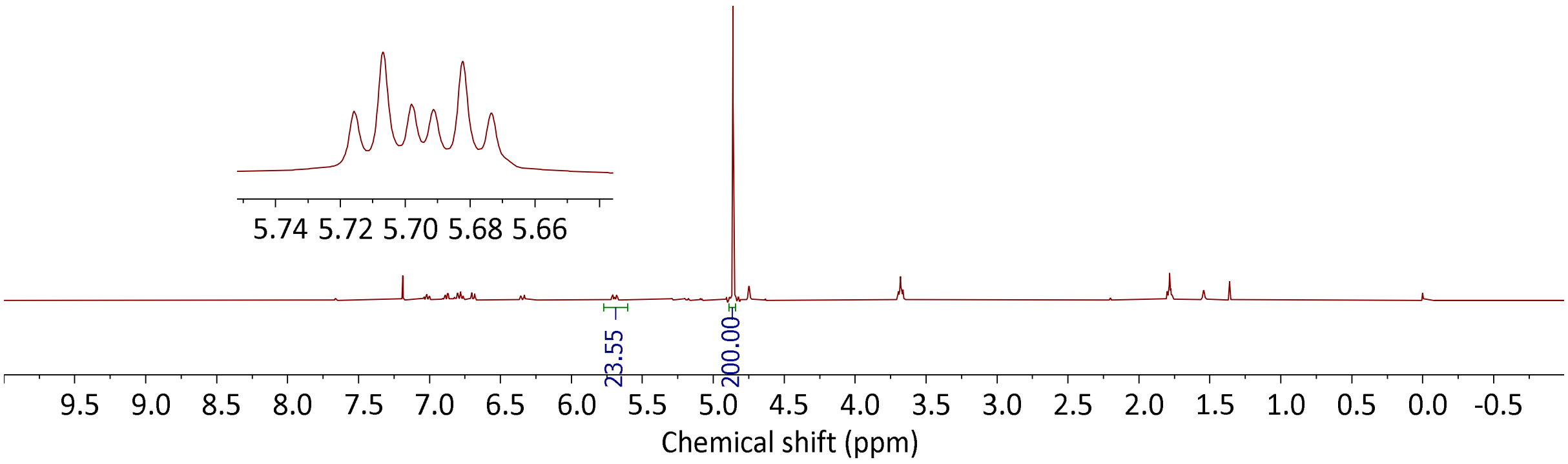

Figure S96. ^{1}H NMR spectrum of allylic cyclisation with [**6**][$BArF_{20}$] as the catalyst.

4. Crystallography Tables

Table S17. Crystallographic details for [**1**][$BArF_{20}$], [**2**][$BArF_{20}$], and [**3**][$BArF_{20}$].

**Compound**	[**1**][$BArF_{20}$]	[**2**][$BArF_{20}$]	[**3**][$BArF_{20}$]
**Empirical formula**	$C_{32}H_9BF_{21}N_3$	$C_{32}H_9BClF_{20}N_3$	$C_{32}H_9BBrF_{20}N_3$
**Formula Weight**	845.23	861.68	906.14
**Temperature (K)**	100.00(10)	100.15	100.00(10)
**Wavelength (Å)**	1.54184	1.54184	1.54184
**Crystal system**	Triclinic	Orthorhombic	Orthorhombic
**Space group**	*P*1	*Fdd*2	*Fdd*2
***a* (Å)**	10.48910(10)	34.77919(15)	34.87730(13)
***b* (Å)**	17.04420(10)	33.71990(12)	33.80660(13)
***c* (Å)**	17.75610(10)	10.39626(4)	10.37880(4)
**α (°)**	98.6690(10)		
**β (°)**	90.6240(10)		
**γ (°)**	93.6120(10)		
**Volume (Å^3)**	3131.24(4)	12192.23(9)	12237.47(8)
Z	4	16	16
**ρ_{calc} (Mg/m^3)**	1.793	1.878	1.967
**Crystal size (mm^3)**	0.224 × 0.181 × 0.07	0.125 × 0.103 × 0.055	0.25 × 0.23 × 0.06
**θ range (°)**	2.518 to 75.980	3.652 to 75.930	3.642 to 75.955
**Total reflections**	143095	68075	67143
**Unique reflections**	24188	6261	5954
**Parameters**	2172	516	516
**Completeness**	99.9	100.0	100.0
**R_{int}**	0.0391	0.0388	0.046
**R_1 (I > 2σ)**	0.0369	0.0225	0.0243
**R_1 (all data)**	0.0430	0.0228	0.0246
**wR_2 (I > 2σ)**	0.0914	0.0619	0.0661
**wR_2 (all data)**	0.0950	0.0622	0.0663
**Goodness of fit, *S***	1.034	1.054	1.041
**Deposition Number (CCDC)**	2564493	2564490	2564489

Table S18. Crystallographic details for [**4**][$BArF_{20}$], [**5**][$BArF_{20}$]•$(DCM)_{0.5}$, and [**6**][$BArF_{20}$]

**Compound**	[**4**][$BArF_{20}$]	[**5**][$BArF_{20}$]•$(DCM)_{0.5}$	[**6**][$BArF_{20}$]
**Empirical formula**	$C_{32}H_8BF_{22}N_3$	$C_{65}H_{18}B_2Cl_2F_{44}N_6$	$C_{32}H_6BF_{24}N_3$
**Formula Weight**	863.22	1811.37	899.21
**Temperature (K)**	100.00(10)	100.00(10)	99.9(6)
**Wavelength (Å)**	1.54184	1.54184	1.54184
**Crystal system**	Monoclinic	Triclinic	Orthorhombic
**Space group**	*Cc*	$P\bar{1}$	*Fdd*2
***a* (Å)**	9.67650(10)	9.98080(10)	34.7384(10)
***b* (Å)**	18.83700(10)	17.2138(2)	34.3715(10)
***c* (Å)**	17.02090(10)	20.1713(2)	10.3876(3)
**α (°)**		74.7390(10)	
**β (°)**	96.9730(10)	78.1840(10)	
**γ (°)**		81.5250(10)	
**Volume (Å^3)**	3079.56(4)	3256.45(6)	12402.9(6)
Z	4	2	16
**ρ_{calc} (Mg/m^3)**	1.862	1.847	1.926
**Crystal size (mm^3)**	0.33 × 0.25 × 0.14	0.35 × 0.24 × 0.19	0.183 × 0.056 × 0.016
**θ range (°)**	4.695 to 75.831	2.306 to 75.908	3.618 to 76.365
**Total reflections**	53647	127161	31113
**Unique reflections**	5984	13346	5247
**Parameters**	635	1196	549
**Completeness**	99.9	99.9	100.0
**R_{int}**	0.0672	0.0692	0.0332
**R_1 ($I > 2\sigma$)**	0.0327	0.0348	0.0261
**R_1 (all data)**	0.0337	0.0385	0.0285
**wR_2 ($I > 2\sigma$)**	0.0843	0.0887	0.0661
**wR_2 (all data)**	0.0852	0.0910	0.0671
**Goodness of fit, *S***	1.041	1.021	1.065
**Deposition Number (CCDC)**	2564488	2564492	2564487

Table S19. Crystallographic details for [**7**][$BArF_{20}$].

**Compound**	[**7**][$BArF_{20}$]
**Empirical formula**	$C_{33}H_{12}BF_{20}N_3$
**Formula Weight**	841.27
**Temperature (K)**	100.00(10)
**Wavelength (Å)**	1.54184
**Crystal system**	Orthorhombic
**Space group**	*Fdd*2
a **(Å)**	34.92676(15)
b **(Å)**	33.84930(14)
c **(Å)**	10.39118(4)
**α (°)**	
**β (°)**	
**γ (°)**	
**Volume ($Å^3$)**	12284.93(9)
Z	16
**ρ_{calc} (Mg/m^3)**	1.819
**Crystal size (mm^3)**	0.33 × 0.22 × 0.15
**θ range (°)**	3.637 to 76.097
**Total reflections**	81318
**Unique reflections**	6237
**Parameters**	517
**Completeness**	100.0
**R_{int}**	0.0468
**R_1 ($I > 2\sigma$)**	0.0263
**R_1 (all data)**	0.0268
**wR_2 ($I > 2\sigma$)**	0.0677
**wR_2 (all data)**	0.0680
**Goodness of fit, *S***	1.044
**Deposition Number (CCDC)**	2564491

5. Optimised coordinates

Table S20. Optimised coordinates (B3LYP/def2-TZVP) for [**1**]$^+$.

Atom	X	Y	Z
H	5.157719	2.580729	14.17849
F	1.489894	3.611696	11.81275
N	6.490227	1.615395	11.76572
N	5.494925	1.675643	9.912946
N	6.619023	1.3346	10.4961
C	4.596475	2.194771	10.81078
C	5.258938	2.154711	12.0445
C	3.297523	2.686738	10.67511
H	2.761218	2.729924	9.73846
C	4.654663	2.608344	13.2225
C	2.726248	3.126441	11.84759
C	3.374524	3.095669	13.10176
H	2.834775	3.466198	13.96255
C	5.331902	1.480815	8.473066
H	5.149663	2.443004	7.997031
H	4.494909	0.80819	8.292566
H	6.250083	1.044281	8.092592
C	7.592659	1.34326	12.68744
H	7.261679	0.634778	13.44495
H	7.908136	2.274218	13.15547
H	8.408816	0.919595	12.11063

Table S21. Optimised coordinates (B3LYP/def2-TZVP) for [**1**][$BArF_{20}$].

Atom	X	Y	Z
F	-1.83047	-3.14141	-4.08024
F	3.15553	0.611232	1.936392
F	2.888492	-2.2245	1.581329
F	1.257507	1.779907	-2.13803
F	-1.34639	0.747178	-1.5433
F	1.747737	-2.83003	-1.07843
F	6.943022	0.476836	-0.81333
F	2.7944	-0.68815	-2.61561
F	0.825552	4.397782	-2.00201
F	0.339902	1.42327	2.511572
F	-6.29615	-2.14161	-1.17049
F	0.396122	-4.20762	-2.90603
F	-0.55098	-3.58161	4.432478
F	-2.26934	-1.82724	3.219617
F	-2.656	-0.63674	-3.34688
F	-1.44422	-0.30586	1.211376
F	5.777619	0.953954	1.610381
F	2.031126	-3.73355	3.560545
F	5.395574	-0.34677	-2.90203

F	0.140607	5.567673	0.371512
F	-0.10259	4.014301	2.603144
H	-5.82907	-2.23512	1.32658
C	0.33102	-0.93887	-1.23286
C	1.609079	-2.05554	1.942186
C	0.788326	-1.13002	1.298359
C	-1.14107	-2.44509	-3.17193
C	3.669405	0.342848	0.717357
C	0.928415	2.282476	-0.93433
C	-0.98578	-1.92081	2.808562
C	-0.01075	-2.97905	-2.57201
C	3.473267	-0.30558	-1.51675
C	-0.51852	-1.13351	1.773036
C	2.826618	-0.10146	-0.30121
N	-3.59623	1.836635	1.257998
C	5.033137	0.536991	0.578065
C	0.479448	2.087476	1.343387
C	-5.17463	-0.09922	-1.24737
H	-5.31493	-0.03267	-2.31549
C	-0.12393	-2.80745	3.430809
C	-5.44375	-1.33897	0.860445
C	-1.54531	-1.17865	-2.79734
C	0.806087	1.427551	0.16304
C	0.679474	-2.23235	-1.6291
C	5.62926	0.2964	-0.64984
C	-0.82097	-0.47025	-1.84884
C	0.716234	3.651472	-0.89412
C	-4.76823	-0.39318	1.594621
H	-4.58515	-0.52368	2.650549
N	-3.35843	2.615132	0.237559
C	-4.49625	0.856734	-0.48959
C	-3.74976	2.654753	-2.131
H	-3.16757	3.562866	-2.01099
H	-4.73636	2.88849	-2.52867
H	-3.22606	1.960706	-2.78489
C	0.369695	4.251634	0.305769
C	-4.30085	0.719649	0.888888
C	4.839262	-0.12482	-1.70413
N	-3.89128	2.042476	-0.81375
C	0.253765	3.457171	1.430378
B	1.193838	-0.18734	-0.01787
C	-3.08374	2.185917	2.581196
H	-2.33984	1.449489	2.876987
H	-3.91054	2.202341	3.2892
H	-2.62457	3.16684	2.513835
C	1.184016	-2.87452	2.985408
C	-5.63415	-1.176	-0.52746

Table S22. Optimised coordinates (B3LYP/def2-TZVP) for **1˙**.

Atom	X	Y	Z
N	-1.07958	-0.91227	0.785958
N	-1.88337	-0.54893	-0.25933
N	-1.28088	0.592226	-0.7204
C	-1.84286	1.288168	-1.84035
C	-1.38518	-2.08742	1.544754
C	-0.17082	0.929508	-0.01568
C	0.724704	1.977826	-0.1306
H	0.644514	2.7494	-0.88232
C	1.76333	1.985507	0.799846
F	2.6561	2.988672	0.722174
C	1.922461	1.03393	1.781248
H	2.757176	1.115298	2.4628
C	1.00506	-0.02391	1.884797
H	1.114139	-0.78049	2.64933
C	-0.03244	-0.05533	0.976299
H	-1.12958	1.332167	-2.66721
H	-2.12724	2.30589	-1.5615
H	-2.72819	0.745768	-2.16151
H	-2.28411	-2.53317	1.126832
H	-1.56409	-1.83718	2.593943
H	-0.56717	-2.81066	1.487915

Table S23. Optimised coordinates (B3LYP/def2-TZVP) for [**1**][F] (FIA).

Atom	X	Y	Z
H	5.201038	2.656487	14.14419
F	1.465795	3.552839	11.80294
N	6.615909	1.885437	11.71688
N	5.61547	1.945925	9.855129
N	6.721256	1.54658	10.45234
C	4.678099	2.356631	10.77719
C	5.336758	2.317814	12.00938
C	3.356931	2.773463	10.66148
H	2.817444	2.803133	9.726552
C	4.701616	2.693858	13.18668
C	2.748676	3.142485	11.84497
C	3.383698	3.111692	13.08862
H	2.825778	3.411848	13.96436
C	5.381946	1.442892	8.51245
H	4.531997	1.96573	8.080831
H	5.201095	0.370854	8.599374
H	6.268963	1.624547	7.910651
C	7.588016	1.309365	12.62963
H	7.377613	0.240758	12.68829
H	7.498217	1.796095	13.59765

H	8.585012	1.471167	12.22757
F	6.069673	-0.4286	10.72984

Table S24. Optimised coordinates (B3LYP/def2-TZVP) for [**1**]$^+$ (triplet).

Atom	X	Y	Z
H	0.450419	-2.81173	0.000074
F	3.550066	0.445597	0.00004
N	-1.64875	-0.8545	-0.00036
N	-1.2269	1.25726	0.000161
N	-2.27005	0.406039	-3.4E-05
C	-0.01739	0.617956	0.000007
C	-0.32018	-0.76824	-0.00012
C	1.282455	1.060479	-8E-06
H	1.582771	2.098174	-1.8E-05
C	0.696719	-1.75996	-1.2E-05
C	2.308012	0.010391	0
C	2.035083	-1.33104	0.000007
H	2.849394	-2.04122	0.000061
C	-1.48375	2.689167	0.000003
H	-1.05554	3.143026	-0.89413
H	-1.0509	3.1439	0.891433
H	-2.56028	2.830276	0.00273
C	-2.50034	-2.03269	0.000189
H	-2.31569	-2.6243	0.89724
H	-2.30328	-2.63376	-0.88781
H	-3.53126	-1.69265	-0.0087

Table S25. Optimised coordinates (B3LYP/def2-TZVP) for [**1**(IMe)]$^+$.

Atom	X	Y	Z
C	2.427216	2.028997	-1.44915
N	2.803216	0.866725	-0.64681
C	4.087446	0.458576	-0.35426
C	3.996154	-0.69754	0.339548
N	2.651105	-0.98234	0.506265
C	2.158332	-2.07326	1.354334
C	1.933113	-0.01709	-0.10988
H	1.595101	1.75531	-2.09434
H	3.284182	2.321683	-2.04938
H	2.119745	2.853199	-0.80817
H	4.948593	1.016549	-0.67509
H	4.761583	-1.34215	0.732722
H	2.747546	-2.96526	1.152082
H	1.111393	-2.25568	1.136072
H	2.268363	-1.79539	2.40207
H	-2.44667	2.742677	0.565733
F	-5.12098	-0.78532	-0.22864

N	-0.0591	1.229995	0.377533
N	-0.27741	-1.07547	-0.23222
N	0.585604	0.114887	-0.28785
C	-1.55951	-0.46956	-0.1119
C	-1.43906	0.882627	0.187079
C	-2.79906	-1.06568	-0.25596
H	-2.93728	-2.1121	-0.487
C	-2.54812	1.689312	0.342289
C	-3.90516	-0.23908	-0.08829
C	-3.80931	1.112291	0.202397
H	-4.71104	1.69802	0.309831
C	-0.08584	-1.87868	-1.44716
H	-0.26006	-1.29271	-2.35436
H	-0.78261	-2.71301	-1.41019
H	0.922065	-2.2919	-1.46452
C	0.285145	1.315063	1.816667
H	0.040511	0.400468	2.366807
H	-0.2872	2.140981	2.233165
H	1.342497	1.554752	1.929545

Table S26. Optimised coordinates (B3LYP/def2-TZVP) for [**1**(PMe_3)]$^+$.

Atom	X	Y	Z
P	-2.20919	-0.2497	-0.57654
C	-3.36994	1.114031	-0.68016
H	-3.87408	1.244934	0.277066
H	-2.84097	2.028508	-0.9435
H	-4.11209	0.892791	-1.44864
C	-3.07369	-1.74945	-0.10583
H	-2.36673	-2.57366	-0.02554
H	-3.57562	-1.60301	0.850334
H	-3.81778	-1.9868	-0.86768
C	-1.41701	-0.48852	-2.17548
H	-0.87765	0.414701	-2.45842
H	-0.72133	-1.32526	-2.12275
H	-2.17839	-0.70273	-2.92773
H	1.831157	2.682342	-0.70203
F	4.415772	-0.99607	-0.64783
N	-0.43366	1.353876	0.399898
N	-0.19592	-0.97498	0.88249
N	-1.13879	0.115914	0.68507
C	1.017869	-0.45341	0.33781
C	0.880398	0.898908	0.047597
C	2.206767	-1.12595	0.116698
H	2.341417	-2.17708	0.330142
C	1.932634	1.629077	-0.47746
C	3.251767	-0.37633	-0.40964
C	3.139944	0.973773	-0.7089

H	3.99095	1.496377	-1.12236
C	-0.12208	-1.41589	2.287287
H	0.21658	-0.62184	2.95804
H	0.57765	-2.24852	2.337766
H	-1.10089	-1.76889	2.60357
C	-0.48388	2.299662	1.529732
H	-1.5202	2.558842	1.734999
H	0.044811	3.202798	1.229183
H	-0.01912	1.89745	2.434001

Table S27. Optimised coordinates (B3LYP/def2-TZVP) for [**1**][H] (HIA).

Atom	X	Y	Z
H	-0.54614	-2.77992	-0.0905
F	-3.56646	0.55367	0.175097
N	1.666201	-1.01348	-0.3852
N	1.203285	1.297165	-0.426
N	2.212828	0.290093	-0.71452
C	0.001839	0.574765	-0.26592
C	0.268538	-0.79593	-0.25662
C	-1.28779	1.057443	-0.11225
H	-1.5238	2.112411	-0.11598
C	-0.75095	-1.71752	-0.10051
C	-2.29406	0.11219	0.039456
C	-2.05935	-1.25142	0.048121
H	-2.88989	-1.93376	0.162189
C	1.58412	2.187605	0.671157
H	1.719476	1.65731	1.621672
H	0.804395	2.939191	0.794536
H	2.511589	2.691821	0.408287
C	2.32072	-1.60825	0.782566
H	1.888738	-2.59401	0.955316
H	2.20122	-1.00552	1.691542
H	3.380942	-1.72699	0.568619
H	2.277007	0.286709	-1.72752

Table S28. Optimised coordinates (B3LYP/def2-TZVP) for [**2**]$^{+}$.

Atom	X	Y	Z
H	5.10757	2.465227	14.18704
Cl	0.81732	2.859157	11.97161
N	6.536648	1.940523	11.69109
N	5.485676	1.881431	9.869183
N	6.676677	1.762147	10.40313
C	4.529344	2.144928	10.81874
C	5.229468	2.184044	12.03038
C	3.151092	2.349028	10.74429

H	2.602458	2.320875	9.814484
C	4.580486	2.431972	13.24412
C	2.518845	2.593128	11.94781
C	3.22154	2.634219	13.17926
H	2.661748	2.830642	14.08276
C	5.315655	1.731911	8.424945
H	4.910396	2.654376	8.012151
H	4.638841	0.902626	8.225499
H	6.29186	1.528354	7.996009
C	7.704866	1.866647	12.56756
H	7.561004	1.067046	13.2923
H	7.831635	2.819242	13.07923
H	8.570872	1.657475	11.94741

Table S29. Optimised coordinates (B3LYP/def2-TZVP) for [**2**][BArF$_{20}$].

Atom	X	Y	Z
F	-2.05191	-3.21529	-3.77403
F	3.389451	0.55898	1.82029
F	2.914225	-2.26949	1.666836
F	1.443603	1.590131	-2.26514
F	-1.20556	0.78895	-1.52874
F	1.641992	-2.96516	-0.90979
F	7.062692	-0.02786	-1.02347
F	2.784144	-1.00885	-2.61984
F	1.197253	4.235251	-2.29434
F	0.654833	1.604546	2.421687
Cl	-6.58007	-2.01625	-0.98591
F	0.133602	-4.35984	-2.59628
F	-0.51207	-3.1863	4.703313
F	-2.14467	-1.39685	3.424882
F	-2.67241	-0.61367	-3.19115
F	-1.28499	-0.07406	1.2945
F	6.016392	0.690585	1.395094
F	2.021969	-3.57976	3.768266
F	5.391573	-0.87454	-3.00489
F	0.670333	5.603418	0.012561
F	0.392752	4.2204	2.34988
H	-5.79489	-1.6706	1.686067
C	0.357744	-0.99051	-1.15192
C	1.662622	-1.98585	2.052674
C	0.886742	-1.04824	1.371764
C	-1.28368	-2.51178	-2.93726
C	3.841663	0.174026	0.608099
C	1.188867	2.192407	-1.08947
C	-0.88563	-1.60903	2.983642
C	-0.17363	-3.08528	-2.33606
C	3.52505	-0.60472	-1.56986

C	-0.40032	-0.92702	1.883716
C	2.935906	-0.27521	-0.35261
N	-3.2891	2.204695	1.249196
C	5.210065	0.260511	0.415966
C	0.80131	2.179108	1.207743
C	-5.08842	0.219476	-1.05988
H	-5.25343	0.240145	-2.12614
C	-0.06672	-2.51223	3.639187
C	-5.37	-0.84704	1.130349
C	-1.58395	-1.19699	-2.6394
C	1.043425	1.421887	0.066071
C	0.6	-2.32945	-1.46813
C	5.745886	-0.10268	-0.80958
C	-0.77866	-0.48129	-1.76457
C	1.072514	3.572639	-1.13602
C	-4.60313	0.093938	1.776129
H	-4.39458	0.02041	2.832968
N	-3.03482	2.892144	0.167754
C	-4.31724	1.173575	-0.39465
C	-3.50858	2.797294	-2.18338
H	-2.86279	3.668851	-2.14683
H	-4.49116	3.070917	-2.56519
H	-3.05705	2.02491	-2.80226
C	0.806486	4.272994	0.029621
C	-4.08178	1.1187	0.982236
C	4.893106	-0.53493	-1.80907
N	-3.64315	2.288445	-0.82208
C	0.672532	3.564052	1.208099
B	1.311842	-0.22503	-0.01644
C	-2.70662	2.607396	2.527503
H	-2.0051	1.842164	2.852231
H	-3.50447	2.731623	3.257682
H	-2.18385	3.546191	2.375988
C	1.217444	-2.70193	3.161169
C	-5.60802	-0.77983	-0.26426

Table S30. Optimised coordinates (B3LYP/def2-TZVP) for **2˙**.

Atom	X	Y	Z
N	-1.09217	-0.92423	0.785152
N	-1.89573	-0.56014	-0.26061
N	-1.29288	0.580134	-0.72057
C	-1.85305	1.277712	-1.84034
C	-1.40077	-2.10075	1.542403
C	-0.18125	0.916186	-0.01336
C	0.71471	1.962033	-0.12573
H	0.623637	2.727706	-0.88208
C	1.761012	1.97831	0.804075

Cl	2.920352	3.274281	0.709135
C	1.906393	1.015844	1.78268
H	2.735181	1.081089	2.473082
C	0.990669	-0.04054	1.88649
H	1.100862	-0.79649	2.651481
C	-0.0474	-0.07095	0.976575
H	-1.13859	1.32179	-2.66614
H	-2.13619	2.295484	-1.5605
H	-2.73876	0.736901	-2.16322
H	-2.29979	-2.54422	1.122407
H	-1.58064	-1.85078	2.591142
H	-0.58359	-2.82437	1.484936

Table S31. Optimised coordinates (B3LYP/def2-TZVP) for [**2**][F] (FIA).

Atom	X	Y	Z
H	5.133785	2.552546	14.15606
Cl	0.807026	2.788177	11.95745
N	6.597261	2.227545	11.65671
N	5.542338	2.165793	9.824406
N	6.727585	1.981058	10.37079
C	4.571645	2.318428	10.79213
C	5.26776	2.358625	12.0029
C	3.190048	2.448665	10.73316
H	2.63855	2.409449	9.80583
C	4.606977	2.530259	13.21264
C	2.542215	2.621182	11.94632
C	3.228023	2.661774	13.16815
H	2.665454	2.791358	14.08139
C	5.372183	1.701266	8.458896
H	4.424939	2.070897	8.073927
H	5.405619	0.611516	8.480933
H	6.189544	2.086264	7.854328
C	7.703948	1.834963	12.51159
H	7.740943	0.745006	12.50856
H	7.539153	2.23352	13.50954
H	8.625395	2.240446	12.10114
F	6.542611	-0.08574	10.54916

Table S32. Optimised coordinates (B3LYP/def2-TZVP) for [**2**]$^{+}$ (triplet).

Atom	X	Y	Z
H	0.130169	-2.86914	-8E-06
Cl	-3.59375	0.235482	0.000001
N	2.081069	-0.75595	0.000015
N	1.499654	1.312473	-1.7E-05
N	2.602319	0.539476	0

C	0.341616	0.584025	-6E-06
C	0.750313	-0.77281	0.000002
C	-0.98801	0.930619	0.000001
H	-1.34704	1.948645	0.000005
C	-0.19087	-1.83769	-2E-06
C	-1.95246	-0.18769	0
C	-1.54978	-1.50792	-1E-06
H	-2.29377	-2.29142	-3E-06
C	1.648638	2.759014	0.000005
H	1.185379	3.180131	0.892689
H	1.184913	3.18019	-0.89241
H	2.711396	2.981319	-0.00027
C	3.019216	-1.86784	-2E-06
H	2.874184	-2.47611	-0.89276
H	2.873802	-2.47645	0.892466
H	4.021377	-1.45058	0.000289

Table S33. Optimised coordinates (B3LYP/def2-TZVP) for [**2**(IMe)]$^+$.

Atom	X	Y	Z
C	2.831521	1.930686	-1.50066
N	3.160048	0.766809	-0.68009
C	4.42614	0.301829	-0.39348
C	4.286549	-0.83539	0.323291
N	2.93136	-1.05089	0.509686
C	2.395587	-2.10198	1.381742
C	2.254165	-0.06329	-0.11714
H	1.975599	1.687555	-2.1266
H	3.692199	2.166082	-2.1205
H	2.578252	2.782682	-0.87242
H	5.309772	0.810789	-0.73407
H	5.024138	-1.50938	0.720418
H	2.936215	-3.02582	1.186798
H	1.337934	-2.23425	1.179368
H	2.533704	-1.81347	2.423248
H	-1.98637	2.909204	0.567225
Cl	-5.21225	-0.64845	-0.18171
N	0.328012	1.285794	0.368597
N	-0.00528	-1.01639	-0.20047
N	0.912715	0.130207	-0.28388
C	-1.25662	-0.34756	-0.07657
C	-1.06772	1.000586	0.201859
C	-2.5243	-0.88528	-0.19654
H	-2.69581	-1.93055	-0.40831
C	-2.13655	1.858348	0.35933
C	-3.60366	-0.01557	-0.03044
C	-3.4237	1.336536	0.241186
H	-4.28499	1.978674	0.354399

C	0.133924	-1.84634	-1.40449
H	-0.0217	-1.26686	-2.31923
H	-0.60252	-2.64458	-1.34673
H	1.120083	-2.30899	-1.42561
C	0.694582	1.384117	1.801427
H	0.163836	2.242088	2.208106
H	1.762572	1.579815	1.896616
H	0.41788	0.491724	2.37235

Table S34. Optimised coordinates (B3LYP/def2-TZVP) for [**2**(PMe_3)]$^+$.

Atom	X	Y	Z
P	-2.46543	-0.44177	-0.58675
C	-3.72196	0.798694	-0.90352
H	-4.2704	1.011667	0.013785
H	-3.25414	1.710775	-1.27074
H	-4.41614	0.421876	-1.65616
C	-3.22971	-1.93691	0.044521
H	-2.46305	-2.68009	0.257895
H	-3.78664	-1.71289	0.953974
H	-3.91482	-2.33272	-0.70667
C	-1.58875	-0.8147	-2.11448
H	-1.12404	0.090066	-2.5046
H	-0.82213	-1.56573	-1.9271
H	-2.29591	-1.201	-2.85085
H	1.341438	2.787975	-0.92926
Cl	4.568275	-0.80323	-0.38659
N	-0.86454	1.414667	0.23962
N	-0.46694	-0.81093	1.014291
N	-1.48304	0.165506	0.652772
C	0.725347	-0.25887	0.449447
C	0.494433	1.033803	-0.00498
C	1.970462	-0.85188	0.349922
H	2.158705	-1.8573	0.698962
C	1.51043	1.779575	-0.57578
C	2.991751	-0.09717	-0.22705
C	2.772248	1.199759	-0.68475
H	3.586844	1.752034	-1.13079
C	-0.41819	-1.06916	2.46482
H	-0.18192	-0.17125	3.04193
H	0.349984	-1.82005	2.642158
H	-1.37496	-1.47157	2.789802
C	-1.045	2.487447	1.234839
H	-2.10698	2.687017	1.359505
H	-0.56861	3.385007	0.843892
H	-0.60122	2.239323	2.202765

Table S35. Optimised coordinates (B3LYP/def2-TZVP) for [**2**][H] (HIA).

Atom	X	Y	Z
H	0.055908	-2.85138	-0.0708
Cl	-3.62959	0.321448	0.121826
N	2.118329	-0.91004	-0.36573
N	1.470094	1.354448	-0.42619
N	2.565545	0.434846	-0.6852
C	0.328713	0.533651	-0.28773
C	0.710427	-0.80894	-0.25592
C	-0.99841	0.907374	-0.16537
H	-1.30473	1.943571	-0.18622
C	-0.23294	-1.80896	-0.10103
C	-1.93996	-0.11009	-0.01678
C	-1.57678	-1.44994	0.016326
H	-2.33763	-2.20923	0.126536
C	1.749378	2.271275	0.680263
H	1.910736	1.751339	1.632482
H	0.904055	2.950702	0.788224
H	2.634326	2.855693	0.437545
C	2.813266	-1.46975	0.795192
H	2.455537	-2.48695	0.954486
H	2.648216	-0.88825	1.710596
H	3.8796	-1.50717	0.582378
H	2.657012	0.434558	-1.69612

Table S36. Optimised coordinates (B3LYP/def2-TZVP) for [**3**]$^{+}$.

Atom	X	Y	Z
H	5.127351	2.496913	14.19015
Br	0.670868	2.993755	11.99699
N	6.528436	1.908415	11.69293
N	5.465524	1.85172	9.877904
N	6.656478	1.71105	10.40627
C	4.521192	2.149219	10.82982
C	5.229397	2.186667	12.03665
C	3.146946	2.384876	10.76122
H	2.59711	2.356967	9.832177
C	4.593951	2.46452	13.25073
C	2.528193	2.658332	11.96479
C	3.238985	2.697906	13.19094
H	2.694447	2.917959	14.09813
C	5.283384	1.688581	8.436697
H	4.897176	2.61516	8.014973
H	4.586213	0.872979	8.251496
H	6.251973	1.457061	8.004684
C	7.699784	1.817869	12.56353
H	7.541643	1.030877	13.29906
H	7.851988	2.773394	13.0627
H	8.556963	1.580782	11.94117

Table S37. Optimised coordinates (B3LYP/def2-TZVP) for [**3**][$BArF_{20}$].

Atom	X	Y	Z
F	-2.18583	-3.21334	-3.42584
F	3.778026	0.445944	1.691428
F	3.049329	-2.32891	1.770977
F	1.770962	1.349893	-2.39637
F	-0.90799	0.84807	-1.51159
F	1.623444	-3.09288	-0.70806
F	7.271688	-0.67534	-1.21739
F	2.864461	-1.37856	-2.59524
F	1.755886	3.997322	-2.61902
F	1.174343	1.777367	2.2993
Br	-6.59045	-1.51048	-0.65108
F	-0.06497	-4.46282	-2.23704
F	-0.32519	-2.70567	4.975817
F	-1.84206	-0.8749	3.616035
F	-2.55431	-0.53152	-3.01783
F	-0.9521	0.204126	1.364304
F	6.387827	0.308167	1.172531
F	2.126749	-3.39469	3.993977
F	5.456557	-1.50887	-3.07375
F	1.443112	5.572135	-0.40692
F	1.139539	4.394187	2.037037
H	-5.57123	-0.93696	2.02578
C	0.508052	-1.03307	-1.05515
C	1.843206	-1.90549	2.17329
C	1.1272	-0.95416	1.447321
C	-1.32761	-2.52383	-2.66868
C	4.14704	-0.06502	0.49778
C	1.616979	2.056946	-1.26226
C	-0.62448	-1.23218	3.15268
C	-0.24948	-3.15051	-2.06245
C	3.678401	-0.96779	-1.60355
C	-0.12376	-0.67975	1.988811
C	3.168471	-0.49873	-0.39636
N	-2.74544	2.646536	1.224281
C	5.509186	-0.1169	0.255389
C	1.321717	2.246405	1.040913
C	-4.80191	0.678445	-0.87617
H	-5.00121	0.6437	-1.93615
C	0.13649	-2.15497	3.849457
C	-5.09705	-0.20009	1.393734
C	-1.5005	-1.16959	-2.45969
C	1.450745	1.389171	-0.04715
C	0.620176	-2.40748	-1.27847
C	5.962753	-0.61497	-0.95588

C	-0.60274	-0.46886	-1.66656
C	1.620009	3.43496	-1.41002
C	-4.22433	0.709421	1.944946
H	-3.98449	0.691553	2.997595
N	-2.47068	3.229556	0.087711
C	-3.92313	1.602091	-0.30689
C	-3.03816	3.013805	-2.23434
H	-2.31794	3.824402	-2.28139
H	-4.00629	3.346581	-2.60591
H	-2.67828	2.162135	-2.80761
C	1.462974	4.239665	-0.29297
C	-3.64248	1.622684	1.062347
C	5.036921	-1.03962	-1.89158
N	-3.16741	2.616906	-0.83577
C	1.314804	3.633616	0.939948
B	1.569437	-0.27689	-0.01258
C	-2.08095	3.082087	2.450738
H	-1.44078	2.279059	2.809172
H	-2.83597	3.332119	3.194078
H	-1.48033	3.954386	2.214156
C	1.379723	-2.49478	3.347014
C	-5.37924	-0.20895	0.00653

Table S38. Optimised coordinates (B3LYP/def2-TZVP) for **3**$^{\bullet}$.

Atom	X	Y	Z
N	-1.09694	-0.92881	0.784887
N	-1.90031	-0.56439	-0.26099
N	-1.29732	0.575618	-0.7206
C	-1.857	1.273737	-1.84041
C	-1.40657	-2.10573	1.541547
C	-0.1856	0.91117	-0.01287
C	0.710932	1.956958	-0.12468
H	0.616224	2.720619	-0.88254
C	1.756758	1.971919	0.805864
Br	3.021388	3.383395	0.704231
C	1.901576	1.00917	1.784215
H	2.728304	1.069044	2.477486
C	0.985218	-0.04691	1.887181
H	1.094888	-0.80316	2.651973
C	-0.05259	-0.07631	0.976622
H	-1.14217	1.317765	-2.66585
H	-2.13978	2.291473	-1.56019
H	-2.74277	0.733256	-2.16363
H	-2.30563	-2.54821	1.120667
H	-1.58684	-1.85599	2.590182
H	-0.58976	-2.82962	1.483896

Table S39. Optimised coordinates (B3LYP/def2-TZVP) for [**3**][F] (FIA).

Atom	X	Y	Z
H	5.15442	2.581897	14.15865
Br	0.660638	2.918799	11.98232
N	6.594883	2.191109	11.65532
N	5.528402	2.133797	9.829324
N	6.711794	1.926083	10.37144
C	4.567378	2.321506	10.8008
C	5.271302	2.359058	12.00717
C	3.188567	2.485027	10.74781
H	2.635246	2.447865	9.821592
C	4.622089	2.56142	13.21827
C	2.553662	2.688149	11.96253
C	3.245858	2.726749	13.17996
H	2.696812	2.881355	14.0973
C	5.339226	1.657507	8.470303
H	4.395163	2.038957	8.08921
H	5.354041	0.567673	8.504702
H	6.157846	2.021789	7.854658
C	7.69758	1.784685	12.50879
H	7.708381	0.694291	12.52358
H	7.55031	2.203066	13.50138
H	8.625638	2.160644	12.08538
F	6.479763	-0.13043	10.57253

Table S40. Optimised coordinates (B3LYP/def2-TZVP) for $[\mathbf{3}]^{+}$ (triplet).

Atom	X	Y	Z
H	0.841476	-2.89589	-0.00016
Br	-3.13582	0.114195	0.000033
N	2.728923	-0.71374	-4.4E-05
N	2.070707	1.334161	0.000092
N	3.202604	0.603477	-1.2E-05
C	0.93955	0.561419	0.000061
C	1.399287	-0.78204	0.000004
C	-0.39872	0.862902	0.000031
H	-0.78298	1.871514	0.000034
C	0.492196	-1.87401	-0.00012
C	-1.32159	-0.28072	-4.2E-05
C	-0.87484	-1.58853	-0.00013
H	-1.58871	-2.39936	-0.00021
C	2.162552	2.784415	-0.00011
H	1.683289	3.18803	0.892409
H	1.68309	3.18782	-0.89262
H	3.215768	3.048406	-0.00026
C	3.727098	-1.76642	0.000122
H	3.231281	-2.73247	0.000066
H	4.350633	-1.66709	0.888425

H	4.350914	-1.66714	-0.88799

Table S41. Optimised coordinates (B3LYP/def2-TZVP) for [**3**(IMe)]$^+$.

Atom	X	Y	Z
C	-3.49982	1.839575	1.546165
N	-3.78672	0.675083	0.711023
C	-5.03449	0.159425	0.430936
C	-4.8536	-0.95908	-0.30588
N	-3.49267	-1.11174	-0.51078
C	-2.92123	-2.12615	-1.4033
C	-2.85203	-0.10532	0.124542
H	-2.62324	1.626753	2.154215
H	-4.36022	2.023898	2.183465
H	-3.29736	2.712869	0.928778
H	-5.93557	0.623381	0.789632
H	-5.56561	-1.65862	-0.70551
H	-3.41867	-3.07543	-1.21579
H	-1.85666	-2.21447	-1.21364
H	-3.0833	-1.82979	-2.43913
H	1.247495	3.057127	-0.56842
Br	4.787856	-0.41805	0.116211
N	-0.99143	1.332259	-0.36518
N	-0.55265	-0.96017	0.170623
N	-1.51843	0.144478	0.27909
C	0.667149	-0.23594	0.043054
C	0.416977	1.10584	-0.217
C	1.958794	-0.71936	0.143468
H	2.172365	-1.75968	0.340431
C	1.445374	2.011373	-0.37491
C	2.996443	0.198832	-0.02279
C	2.755851	1.544479	-0.27594
H	3.58366	2.228816	-0.39099
C	-0.64284	-1.81149	1.36424
H	-0.50064	-1.23882	2.285409
H	0.125455	-2.57745	1.286773
H	-1.60849	-2.31534	1.38977
C	-1.37788	1.432488	-1.79263
H	-0.89157	2.319349	-2.19294
H	-2.45468	1.580173	-1.87387
H	-1.06681	0.561519	-2.37876

Table S42. Optimised coordinates (B3LYP/def2-TZVP) for [**3**(PMe_3)]$^+$.

Atom	X	Y	Z
P	2.983488	-0.58017	-0.5877
C	3.653638	-2.08653	0.119837
H	4.249486	-1.84959	1.000958

H	2.840974	-2.75647	0.396214
H	4.288199	-2.57651	-0.62017
C	4.319252	0.540464	-1.00943
H	3.911091	1.464112	-1.41666
H	4.911274	0.76072	-0.12145
H	4.957621	0.068305	-1.75769
C	2.043073	-0.97943	-2.07036
H	1.2367	-1.66883	-1.82284
H	1.62485	-0.0691	-2.49839
H	2.704293	-1.44902	-2.80098
H	-0.60405	2.880625	-1.05207
Br	-4.20503	-0.49013	-0.19993
N	1.536671	1.432827	0.147747
N	1.01038	-0.70255	1.08301
N	2.079836	0.173518	0.63129
C	-0.15736	-0.10665	0.510366
C	0.148143	1.13428	-0.03506
C	-1.443	-0.616	0.481901
H	-1.68428	-1.58075	0.905002
C	-0.83076	1.911361	-0.62822
C	-2.42585	0.169407	-0.11904
C	-2.13243	1.41507	-0.66717
H	-2.91637	1.996972	-1.12987
C	0.985992	-0.8551	2.549067
H	1.920337	-1.30414	2.878317
H	0.170821	-1.53225	2.798554
H	0.833016	0.0961	3.065734
C	1.822744	2.556189	1.058592
H	1.393267	3.455392	0.620007
H	2.899316	2.690853	1.136203
H	1.398238	2.405698	2.054751

Table S43. Optimised coordinates (B3LYP/def2-TZVP) for [**3**][H] (HIA).

Atom	X	Y	Z
H	0.800189	-2.88333	-0.07191
Br	-3.17621	0.164804	0.070788
N	2.781913	-0.85755	-0.35035
N	2.040501	1.377319	-0.42843
N	3.177402	0.50406	-0.66774
C	0.933065	0.50967	-0.30118
C	1.370634	-0.81558	-0.25841
C	-0.41044	0.828095	-0.19697
H	-0.75482	1.851897	-0.22607
C	0.468511	-1.85399	-0.1107
C	-1.30834	-0.22842	-0.05552
C	-0.89131	-1.55149	-0.0119
H	-1.61687	-2.34516	0.092046

C	2.2658	2.310517	0.676821
H	2.434112	1.80232	1.634157
H	1.392147	2.955621	0.768548
H	3.129776	2.929039	0.443441
C	3.486566	-1.3881	0.818267
H	3.167798	-2.41849	0.975341
H	3.289093	-0.81239	1.730862
H	4.555647	-1.38338	0.616431
H	3.284493	0.504702	-1.67713

Table S44. Optimised coordinates (B3LYP/def2-TZVP) for [**4**]$^{+}$.

Atom	X	Y	Z
F	5.235602	2.414979	14.41626
F	1.280026	3.039352	11.97605
N	6.533761	1.837999	11.74328
N	5.495731	1.874105	9.911058
N	6.670996	1.669363	10.45621
C	4.545218	2.187798	10.85161
C	5.241577	2.162037	12.06678
C	3.185055	2.482431	10.76008
H	2.631753	2.506769	9.833308
C	4.570705	2.440694	13.2658
C	2.574045	2.74848	11.9658
C	3.234824	2.734164	13.21247
H	2.682003	2.954757	14.11557
C	5.331508	1.755045	8.462557
H	4.988547	2.706767	8.059916
H	4.607074	0.971679	8.245521
H	6.298006	1.497437	8.040807
C	7.692494	1.674916	12.62795
H	7.484249	0.886641	13.34815
H	7.881563	2.612454	13.14652
H	8.540263	1.407133	12.00532

Table S45. Optimised coordinates (B3LYP/def2-TZVP) for [**4**][BArF$_{20}$].

Atom	X	Y	Z
F	1.350833	-3.30501	4.210763
F	-3.04089	0.671727	-2.11689
F	-2.81221	-2.17184	-1.8134
F	-1.54205	1.708221	2.168883
F	1.102266	0.671888	1.777829
F	-1.96064	-2.85978	0.932358
F	-7.08147	0.484309	0.24163
F	-3.13398	-0.75154	2.411281
F	-1.11142	4.330194	2.152164

F	-0.19111	1.492502	-2.38233
F	6.054431	-2.18556	1.406866
F	-0.78024	-4.30736	2.823227
F	0.906436	-3.53216	-4.28645
F	2.495413	-1.8082	-2.87169
F	2.25637	-0.78622	3.629211
F	1.469735	-0.28374	-0.96435
F	-5.68152	1.01966	-2.04166
F	-1.75729	-3.66466	-3.70724
F	-5.74997	-0.40344	2.449407
F	-0.22303	5.573567	-0.11456
F	0.230038	4.090373	-2.35961
H	5.951398	-2.00812	-1.14576
C	-0.54954	-0.98808	1.269181
C	-1.5001	-2.00835	-2.03323
C	-0.74696	-1.09755	-1.2928
C	0.744107	-2.5716	3.273294
C	-3.6734	0.372792	-0.96258
C	-1.10491	2.247618	1.016879
C	1.175036	-1.88751	-2.611
C	-0.33751	-3.07309	2.56491
C	-3.70039	-0.33624	1.261752
C	0.602914	-1.10201	-1.62785
C	-2.93662	-0.10359	0.12153
N	3.59795	2.002134	-0.94753
C	-5.04366	0.570756	-0.95292
C	-0.44576	2.122032	-1.21451
C	4.89791	-0.16551	1.540293
H	4.895523	-0.20509	2.618581
C	0.378645	-2.76114	-3.33161
C	5.481108	-1.18599	-0.62499
C	1.187853	-1.29804	2.975688
C	-0.87821	1.426708	-0.08977
C	-0.94046	-2.28984	1.592098
C	-5.75858	0.300787	0.203388
C	0.547998	-0.5515	1.996475
C	-0.89718	3.61786	1.037287
C	4.889097	-0.16778	-1.32226
F	4.897759	-0.13766	-2.65087
N	3.225359	2.669501	0.110005
C	4.304084	0.855428	0.797479
C	3.380286	2.506993	2.50209
H	2.72253	3.365159	2.408442
H	4.321243	2.799669	2.966445
H	2.890574	1.726396	3.078665
C	-0.44789	4.255124	-0.10817
C	4.280861	0.867433	-0.60139
C	-5.07763	-0.15282	1.318576

N	3.642875	2.00182	1.156818
C	-0.22475	3.495058	-1.24035
B	-1.28431	-0.19099	0.000353
C	3.197956	2.459443	-2.28084
H	2.462033	1.766298	-2.68322
H	4.07614	2.495079	-2.92064
H	2.757614	3.445095	-2.17237
C	-0.97087	-2.81937	-3.03383
C	5.46724	-1.1632	0.784157

Table S46. Optimised coordinates (B3LYP/def2-TZVP) for **4•**.

Atom	X	Y	Z
F	5.180748	2.246864	14.43094
F	1.289304	3.066814	11.94012
N	6.518939	1.61204	11.78984
N	5.464032	1.734609	9.913893
N	6.719219	1.543163	10.43222
C	4.552543	2.084538	10.862
C	5.235076	2.016396	12.08348
C	3.215901	2.441088	10.7713
H	2.683492	2.512807	9.834751
C	4.554359	2.304466	13.24593
C	2.583407	2.71783	11.97473
C	3.21088	2.660271	13.20335
H	2.674475	2.889018	14.11223
C	5.317587	1.844223	8.49136
H	5.431193	2.878247	8.151593
H	4.334729	1.475783	8.197579
H	6.080451	1.227851	8.021088
C	7.688621	1.799219	12.60879
H	7.462514	1.506515	13.6311
H	8.027152	2.840223	12.5962
H	8.480377	1.163035	12.21851

Table S47. Optimised coordinates (B3LYP/def2-TZVP) for [**4**][F] (FIA).

Atom	X	Y	Z
F	5.253632	2.535899	14.40842
F	1.264535	2.975482	11.96174
N	6.619319	2.121249	11.7139
N	5.571025	2.158128	9.874185
N	6.737977	1.884606	10.42661
C	4.601872	2.353133	10.83387
C	5.295881	2.332302	12.04696
C	3.230354	2.570557	10.75382
H	2.67814	2.577271	9.826565
C	4.604466	2.542158	13.23353

C	2.592842	2.765613	11.96222
C	3.24326	2.758425	13.1984
H	2.6872	2.915602	14.11113
C	5.382439	1.725971	8.499744
H	4.457772	2.151779	8.118311
H	5.358643	0.635829	8.505947
H	6.221488	2.077887	7.904878
C	7.696716	1.636359	12.56631
H	7.657678	0.547002	12.54043
H	7.554729	2.024768	13.56987
H	8.641846	1.984359	12.15723
F	6.395186	-0.15838	10.55296

Table S48. Optimised coordinates (B3LYP/def2-TZVP) for [**4**]$^{+}$ (triplet).

Atom	X	Y	Z
F	-0.60772	2.726643	-0.00052
F	-3.4596	-0.99067	0.000098
N	1.650691	0.741288	0.000002
N	1.365287	-1.39999	0.000641
N	2.353473	-0.47903	0.000042
C	0.117813	-0.84649	0.000488
C	0.328025	0.554621	0.000282
C	-1.14691	-1.39234	0.00015
H	-1.35154	-2.45326	-0.00012
C	-0.79258	1.42025	-0.00015
C	-2.25864	-0.44913	0.000052
C	-2.10571	0.902576	-7.4E-05
H	-2.95515	1.570627	-5.2E-05
C	1.719034	-2.8115	-0.00074
H	1.32927	-3.29165	-0.89868
H	1.314039	-3.2968	0.887558
H	2.802709	-2.87754	0.008326
C	2.455143	1.950462	0.000205
H	3.088424	1.950049	0.887944
H	1.805504	2.818848	-0.00045
H	3.089386	1.949395	-0.88681

Table S49. Optimised coordinates (B3LYP/def2-TZVP) for [**4**(IMe)]$^{+}$.

Atom	X	Y	Z
C	2.438003	1.906661	-1.62825
N	2.885453	0.8456	-0.72723
C	4.191261	0.558069	-0.39141
C	4.173413	-0.53521	0.403259
N	2.85019	-0.89884	0.587155
C	2.424607	-1.94539	1.523123
C	2.074131	-0.04658	-0.11804

H	1.64067	1.518985	-2.25877
H	3.2818	2.21286	-2.24016
H	2.058529	2.754787	-1.06128
H	5.014811	1.145617	-0.75538
H	4.977702	-1.086	0.856962
H	3.056145	-2.81997	1.380984
H	1.386359	-2.19674	1.334534
H	2.53213	-1.58185	2.544454
F	-2.48074	2.66737	0.387136
F	-4.91092	-1.30316	-0.24783
N	-0.00303	1.093898	0.254711
N	-0.05991	-1.26393	-0.16052
N	0.72185	-0.02568	-0.31433
C	-1.3826	-0.7437	-0.10569
C	-1.34453	0.631958	0.09057
C	-2.57558	-1.43315	-0.22371
H	-2.63967	-2.49954	-0.38121
C	-2.52243	1.346105	0.178229
C	-3.73719	-0.6723	-0.13099
C	-3.74465	0.699113	0.068112
H	-4.67257	1.24834	0.134555
C	0.20729	-2.1613	-1.29238
H	0.005953	-1.67786	-2.25268
H	-0.4278	-3.03728	-1.18199
H	1.242375	-2.49924	-1.26099
C	0.3197	1.342418	1.679821
H	-0.30832	2.169774	2.003901
H	1.358278	1.659372	1.771317
H	0.128068	0.473397	2.317639

Table S50. Optimised coordinates (B3LYP/def2-TZVP) for [**4**(PMe_3)]$^+$.

Atom	X	Y	Z
P	2.335097	-0.12541	-0.60716
C	3.329884	-1.60024	-0.37779
H	3.839604	-1.55753	0.584457
H	2.695063	-2.48407	-0.4174
H	4.07323	-1.65585	-1.17444
C	3.369221	1.335318	-0.49722
H	2.757204	2.228095	-0.61615
H	3.872293	1.361451	0.469096
H	4.11788	1.303545	-1.29033
C	1.539198	-0.18118	-2.22098
H	0.896692	-1.05861	-2.28707
H	0.944338	0.718957	-2.37183
H	2.30356	-0.23866	-2.99818
F	-1.93705	2.649336	-0.26466
F	-4.21026	-1.4358	-0.74166

N	0.444791	1.145589	0.615629
N	0.414651	-1.24115	0.736972
N	1.257426	-0.05646	0.700777
C	-0.85233	-0.75715	0.28791
C	-0.82344	0.63008	0.208408
C	-1.98259	-1.49219	-0.02298
H	-2.02177	-2.57074	0.027301
C	-1.95778	1.311398	-0.19574
C	-3.0987	-0.76486	-0.42111
C	-3.11464	0.619443	-0.51791
H	-4.0023	1.14398	-0.84138
C	0.410057	-1.89874	2.057034
H	1.42105	-2.21609	2.301364
H	-0.22125	-2.78275	1.985762
H	0.02821	-1.24683	2.846933
C	0.430364	1.915503	1.87441
H	0.01694	1.343172	2.709094
H	-0.17919	2.801835	1.707373
H	1.444231	2.229122	2.111899

Table S51. Optimised coordinates (B3LYP/def2-TZVP) for [**4**][H] (HIA).

Atom	X	Y	Z
F	0.70211	2.712552	-0.04614
F	3.441458	-1.10801	0.177547
N	-1.64209	0.91314	-0.37213
N	-1.37236	-1.42718	-0.43066
N	-2.29245	-0.33782	-0.71549
C	-0.11289	-0.81625	-0.26349
C	-0.27342	0.570666	-0.24947
C	1.128567	-1.41478	-0.11177
H	1.270082	-2.48577	-0.12517
C	0.839088	1.372022	-0.08592
C	2.21314	-0.56281	0.045497
C	2.104695	0.817679	0.060183
H	2.975496	1.446465	0.173364
C	-1.8313	-2.30105	0.65014
H	-1.93132	-1.77531	1.607147
H	-1.11509	-3.11424	0.766413
H	-2.79378	-2.72515	0.372736
C	-2.23848	1.55261	0.804768
H	-1.72785	2.500288	0.972645
H	-2.1584	0.937114	1.709209
H	-3.28795	1.750707	0.598095
H	-2.35152	-0.32046	-1.72882

Table S52. Optimised coordinates (B3LYP/def2-TZVP) for [**5**]$^{+}$.

Atom	X	Y	Z
H	5.215668	2.290754	13.94047
F	1.371227	2.709334	11.67863
N	6.683443	1.834793	11.43758
N	5.661503	1.836815	9.598073
N	6.842965	1.695339	10.14519
C	4.693113	2.075265	10.53916
C	5.373018	2.073793	11.76304
C	3.314273	2.288958	10.45111
H	2.761399	2.295786	9.522839
C	4.719566	2.286057	12.98065
C	2.675076	2.497055	11.64757
C	3.366224	2.495677	12.89164
F	2.651221	2.7067	13.98264
C	5.51261	1.732888	8.146662
H	5.117213	2.669619	7.757174
H	4.836565	0.91235	7.911702
H	6.494856	1.539096	7.727203
C	7.836992	1.728316	12.33063
H	7.677324	0.908383	13.02901
H	7.961265	2.664882	12.87186
H	8.711479	1.532141	11.71818

Table S53. Optimised coordinates (B3LYP/def2-TZVP) for [**5**][$BArF_{20}$].

Atom	X	Y	Z
F	1.685237	-3.04513	4.204587
F	-3.20659	0.507465	-2.00602
F	-2.86498	-2.31651	-1.6091
F	-1.46204	1.779757	2.114123
F	1.178301	0.808423	1.618446
F	-1.80219	-2.85558	1.088474
F	-7.07351	0.295547	0.625621
F	-2.95178	-0.72392	2.571984
F	-1.08894	4.406023	1.959173
F	-0.41034	1.395476	-2.50479
F	6.140457	-1.6463	1.616913
F	-0.47383	-4.18009	2.971049
F	0.699964	-3.58113	-4.34682
F	2.323376	-1.75889	-3.09849
F	2.467104	-0.52569	3.473753
F	1.390761	-0.25604	-1.12702
F	-5.84649	0.776316	-1.76675
F	-1.8997	-3.81267	-3.54852
F	-5.56956	-0.45556	2.772522
F	-0.36907	5.566051	-0.40801
F	-0.02923	3.994101	-2.6152
F	5.976749	-2.13935	-0.96902

C	-0.4418	-0.9258	1.269541
C	-1.58128	-2.10993	-1.93266
C	-0.81063	-1.15329	-1.27269
C	1.007331	-2.37529	3.268553
C	-3.75082	0.238636	-0.80026
C	-1.11204	2.276455	0.914148
C	1.032432	-1.89633	-2.72345
C	-0.08896	-2.94435	2.638374
C	-3.60658	-0.37551	1.447514
C	0.50902	-1.11843	-1.70812
C	-2.92811	-0.16801	0.249925
N	3.416721	1.963108	-1.31686
C	-5.12335	0.394245	-0.70624
C	-0.59732	2.068132	-1.34846
C	4.906207	0.334376	1.447184
H	4.960783	0.493037	2.513615
C	0.219177	-2.81547	-3.36373
C	5.391863	-1.03424	-0.52213
C	1.390048	-1.10166	2.895261
C	-0.93748	1.412779	-0.16913
C	-0.76821	-2.22482	1.66691
C	-5.75069	0.151728	0.505631
C	0.676733	-0.42167	1.917937
C	-0.93289	3.649993	0.863014
C	4.728025	-0.20508	-1.38836
H	4.643033	-0.45141	-2.43603
N	3.062821	2.80848	-0.38341
C	4.237064	1.18721	0.565271
C	3.283663	3.068804	1.992012
H	2.676622	3.936328	1.75341
H	4.228136	3.379876	2.436023
H	2.740718	2.408116	2.664326
C	-0.56952	4.245577	-0.33427
C	4.151462	0.926372	-0.80571
C	-4.98246	-0.23262	1.589728
N	3.547285	2.35657	0.744949
C	-0.40421	3.441967	-1.44591
B	-1.28555	-0.21063	0.019698
C	2.980834	2.169889	-2.69626
H	2.332146	1.346572	-2.98674
H	3.855141	2.217343	-3.34343
H	2.429267	3.10383	-2.73505
C	-1.09851	-2.92245	-2.95574
C	5.479463	-0.76934	0.870504

Table S54. Optimised coordinates (B3LYP/def2-TZVP) for **5˙**.

Atom	X	Y	Z

H	5.226719	2.245055	13.9102
F	1.364855	2.675006	11.65379
N	6.687763	1.725675	11.4568
N	5.646304	1.724541	9.583878
N	6.908575	1.63479	10.10815
C	4.701547	1.988883	10.53243
C	5.383485	1.989004	11.7588
C	3.341968	2.222702	10.4598
H	2.791135	2.245029	9.530742
C	4.727949	2.222705	12.95218
C	2.682565	2.445194	11.66807
C	3.353637	2.445148	12.87488
F	2.67051	2.67487	14.00178
C	5.491332	1.811299	8.161489
H	5.469815	2.850826	7.819675
H	4.565017	1.318266	7.864941
H	6.329243	1.298752	7.694492
C	7.813465	1.80662	12.34043
H	7.577928	1.303478	13.27885
H	8.089414	2.844879	12.54962
H	8.653774	1.301278	11.87

Table S55. Optimised coordinates (B3LYP/def2-TZVP) for [**5**][F] (FIA).

Atom	X	Y	Z
H	5.237665	2.377634	13.91149
F	1.360823	2.646014	11.66289
N	6.748643	2.131115	11.4108
N	5.723318	2.13206	9.560677
N	6.897438	1.926752	10.11926
C	4.738036	2.254233	10.51766
C	5.414951	2.253815	11.73921
C	3.355996	2.386992	10.44376
H	2.802131	2.379165	9.516593
C	4.745111	2.386128	12.95035
C	2.693959	2.517847	11.64678
C	3.374112	2.517438	12.8741
F	2.653817	2.645155	13.99607
C	5.572491	1.700221	8.181542
H	4.63108	2.079214	7.791298
H	5.606184	0.610071	8.18167
H	6.398681	2.099018	7.598451
C	7.838274	1.699871	12.26979
H	7.857076	0.609758	12.24067
H	7.669345	2.077995	13.2751
H	8.77037	2.099905	11.87892
F	6.670501	-0.1534	10.24395

Table S56. Optimised coordinates (B3LYP/def2-TZVP) for [**5**]$^+$ (triplet).

Atom	X	Y	Z
H	0.716939	2.54965	0.000085
F	3.159994	-1.2774	-1.9E-05
N	-1.79795	1.036335	-0.00023
N	-1.67063	-1.11736	0.000126
N	-2.5912	-0.04837	-4.7E-05
C	-0.41077	-0.70356	0.000022
C	-0.46054	0.720067	-5.5E-05
C	0.814045	-1.42294	-1E-06
H	0.858775	-2.50226	-4.8E-05
C	0.68069	1.470099	0.000023
C	1.98313	-0.67038	0.000005
C	1.940898	0.716124	0.000021
F	3.048299	1.413107	0.000007
C	-2.20293	-2.47087	0.000011
H	-1.8669	-3.00186	0.890992
H	-1.87251	-2.99963	-0.89442
H	-3.28594	-2.39677	0.003472
C	-2.40061	2.359881	0.000095
H	-2.09319	2.907832	-0.89123
H	-2.09804	2.9054	0.894589
H	-3.47881	2.23146	-0.00302

Table S57. Optimised coordinates (B3LYP/def2-TZVP) for [**5**(IMe)]$^+$.

Atom	X	Y	Z
C	2.568891	2.067587	-1.50557
N	3.016238	0.9615	-0.66109
C	4.322733	0.657119	-0.34249
C	4.306329	-0.47896	0.389421
N	2.983627	-0.8541	0.552984
C	2.560163	-1.95264	1.428427
C	2.205636	0.035663	-0.10257
H	1.766061	1.715348	-2.14979
H	3.41042	2.400069	-2.10687
H	2.19744	2.889062	-0.89561
H	5.145465	1.264857	-0.67366
H	5.111421	-1.05342	0.811209
H	3.206343	-2.80969	1.250352
H	1.528098	-2.20754	1.212264
H	2.648865	-1.64098	2.468616
H	-2.38742	2.495205	0.457325
F	-4.78857	-1.22763	-0.27076
N	0.121651	1.148081	0.327653
N	0.079552	-1.18619	-0.21574
N	0.853708	0.063351	-0.29734
C	-1.24556	-0.66732	-0.12844

C	-1.22448	0.695294	0.135066
C	-2.44111	-1.35046	-0.26801
H	-2.50392	-2.41002	-0.47132
C	-2.38601	1.432024	0.26116
C	-3.61201	-0.61532	-0.1365
C	-3.58792	0.752378	0.12204
F	-4.74133	1.413711	0.230243
C	0.34093	-2.00008	-1.41103
H	0.12751	-1.45157	-2.33315
H	-0.28823	-2.88582	-1.35691
H	1.378045	-2.33365	-1.41313
C	0.442432	1.307549	1.766276
H	-0.19213	2.103758	2.149267
H	1.478185	1.627343	1.878293
H	0.259455	0.396794	2.345727

Table S58. Optimised coordinates (B3LYP/def2-TZVP) for [**5**(PMe_3)]$^+$.

Atom	X	Y	Z
P	-2.44442	0.000423	-0.66519
C	-3.46611	1.468817	-0.53478
H	-4.00887	1.459389	0.410156
H	-2.84128	2.358855	-0.58949
H	-4.18153	1.480221	-1.35848
C	-3.46714	-1.4672	-0.53479
H	-2.84323	-2.3578	-0.59052
H	-4.00965	-1.45797	0.410294
H	-4.18297	-1.47719	-1.35819
C	-1.58878	-4.2E-05	-2.24908
H	-0.96501	0.889197	-2.33283
H	-0.96618	-0.8901	-2.33285
H	-2.32305	0.000424	-3.05675
H	1.865952	-2.498	-0.01088
F	4.102305	1.343253	-0.62689
N	-0.58951	-1.19598	0.689203
N	-0.58952	1.195061	0.690687
N	-1.41729	0.000458	0.683436
C	0.696224	0.693923	0.310729
C	0.69626	-0.69428	0.31055
C	1.835476	1.41719	-0.00109
H	1.865255	2.498013	-0.0105
C	1.835787	-1.41719	-0.0012
C	2.979924	0.697306	-0.31093
C	2.980053	-0.69698	-0.31095
F	4.102601	-1.34268	-0.62689
C	-0.63057	1.903047	1.983878
H	-1.65185	2.216052	2.188243
H	-0.0089	2.792474	1.895148

H	-0.26181	1.288648	2.809537
C	-0.63175	-1.90537	1.981246
H	-0.0098	-2.79457	1.891814
H	-1.65308	-2.21913	2.184123
H	-0.26394	-1.29218	2.808257

Table S59. Optimised coordinates (B3LYP/def2-TZVP) for [**5**][H] (HIA).

Atom	X	Y	Z
H	-0.80449	-2.49524	-0.12955
F	-3.12246	1.348144	0.129524
N	1.727403	-1.17838	-0.40188
N	1.727096	1.179421	-0.4014
N	2.520902	0.000974	-0.70412
C	0.4019	0.697774	-0.27491
C	0.402314	-0.69735	-0.27464
C	-0.77494	1.414431	-0.13468
H	-0.80559	2.495229	-0.13123
C	-0.77423	-1.41444	-0.13367
C	-1.95377	0.693885	0.002876
C	-1.95339	-0.69434	0.003458
F	-3.12171	-1.34911	0.130642
C	2.249208	1.929107	0.743579
H	2.245522	1.344817	1.671895
H	1.635043	2.818553	0.88408
H	3.266948	2.242767	0.521707
C	2.25069	-1.92964	0.74136
H	1.636908	-2.81949	0.881075
H	2.247767	-1.34685	1.670641
H	3.268291	-2.24283	0.518174
H	2.59252	0.000966	-1.71671

Table S60. Optimised coordinates (B3LYP/def2-TZVP) for [**6**]$^{+}$.

Atom	X	Y	Z
F	5.600458	2.433383	14.41613
F	1.552352	2.649087	12.02538
N	6.904928	1.943328	11.71441
N	5.84751	1.854593	9.892561
N	7.039006	1.770518	10.42505
C	4.889438	2.089474	10.84643
C	5.592176	2.148519	12.05713
C	3.50268	2.256833	10.79926
F	2.836826	2.201106	9.654653
C	4.936898	2.377493	13.2702
C	2.857636	2.482627	11.99646
C	3.569112	2.542486	13.22221

F	2.888424	2.761469	14.32722
C	5.681406	1.698244	8.442378
H	5.240597	2.604782	8.033226
H	5.03398	0.846173	8.246207
H	6.667476	1.531246	8.020594
C	8.086396	1.903422	12.58482
H	7.9628	1.110232	13.31902
H	8.194446	2.863136	13.08583
H	8.946453	1.705847	11.95285

Table S61. Optimised coordinates (B3LYP/def2-TZVP) for [**6**][BArF$_{20}$].

Atom	X	Y	Z
F	1.595089	-2.94442	4.234755
F	-3.46432	0.467533	-1.91297
F	-3.26297	-2.32194	-1.31199
F	-1.37912	1.960223	1.98446
F	1.16057	0.754569	1.416101
F	-2.10016	-2.70544	1.373612
F	-7.16566	0.7336	0.942098
F	-2.99982	-0.38515	2.733921
F	-0.88132	4.538848	1.572374
F	-0.66743	1.12627	-2.64011
F	6.025116	-1.07159	2.101266
F	-0.7325	-3.98939	3.255313
F	0.06072	-4.0265	-4.114
F	1.858982	-2.21962	-3.09823
F	2.499313	-0.55558	3.256448
F	1.134579	-0.51552	-1.2123
F	-6.06321	0.927665	-1.54922
F	-2.49944	-4.02478	-3.1695
F	-5.57927	0.070971	3.058401
F	-0.27206	5.450549	-0.92824
F	-0.16702	3.677359	-3.00609
F	5.975552	-2.22142	-0.30164
C	-0.59703	-0.87633	1.312471
C	-1.9885	-2.22668	-1.71492
C	-1.12514	-1.27451	-1.174
C	0.894731	-2.28965	3.305008
C	-3.94815	0.336231	-0.65965
C	-1.09044	2.331866	0.724741
C	0.58654	-2.24712	-2.65051
C	-0.28666	-2.81381	2.803024
C	-3.70163	-0.09236	1.622244
C	0.167938	-1.36098	-1.67641
C	-3.08809	-0.03378	0.373997
N	3.23626	1.532931	-1.73314
C	-5.30019	0.589508	-0.50196

C	-0.74602	1.903977	-1.54021
C	4.71131	0.7239	1.375723
F	4.757591	1.305347	2.566085
C	-0.3162	-3.15756	-3.17277
C	5.325312	-1.08123	-0.14142
C	1.338724	-1.07867	2.809986
C	-1.03723	1.371653	-0.28759
C	-0.98481	-2.1148	1.830406
C	-5.86427	0.492885	0.760361
C	0.599258	-0.41382	1.841484
C	-0.84792	3.684006	0.538821
C	4.656548	-0.51083	-1.20166
F	4.65609	-1.07823	-2.39949
N	2.819153	2.564483	-1.04949
C	4.023488	1.299335	0.304892
C	2.91813	3.424421	1.191923
H	2.367232	4.218526	0.698634
H	3.82585	3.812919	1.647714
H	2.294706	2.938006	1.93867
C	-0.54119	4.154412	-0.7283
C	3.995652	0.695938	-0.95773
C	-5.05468	0.152665	1.829094
N	3.279115	2.441031	0.16732
C	-0.49283	3.251698	-1.77295
B	-1.4685	-0.20507	0.058765
C	2.85717	1.384504	-3.14203
H	2.433335	0.394196	-3.28264
H	3.740097	1.513743	-3.76549
H	2.116333	2.147036	-3.36113
C	-1.61344	-3.14608	-2.69151
C	5.35283	-0.47019	1.135766

Table S62. Optimised coordinates (B3LYP/def2-TZVP) for **6˙**.

Atom	X	Y	Z
F	5.580026	2.339098	14.41232
F	1.551575	2.643846	12.0058
N	6.898193	1.712121	11.76209
N	5.811731	1.620538	9.891952
N	7.086162	1.608343	10.405
C	4.888589	1.941044	10.85561
C	5.59072	2.000396	12.06426
C	3.524881	2.156122	10.82411
F	2.832815	2.107057	9.683245
C	4.932134	2.275045	13.24654
C	2.863308	2.433335	12.01572
C	3.558735	2.492127	13.21286
F	2.907378	2.758457	14.33974

C	5.685714	1.81872	8.470009
H	5.802189	2.872518	8.199554
H	4.712071	1.46581	8.140036
H	6.463904	1.237125	7.980664
C	8.060048	2.022016	12.55665
H	7.879767	1.734937	13.58935
H	8.304539	3.087595	12.512
H	8.896522	1.447751	12.16449

Table S63. Optimised coordinates (B3LYP/def2-TZVP) for [**6**][F] (FIA).

Atom	X	Y	Z
F	5.608763	2.544652	14.39977
F	1.547122	2.592566	12.01109
N	6.960069	2.234282	11.6787
N	5.898279	2.141841	9.844243
N	7.086195	1.980062	10.39173
C	4.92776	2.256258	10.81884
C	5.627251	2.317199	12.02738
C	3.542485	2.352833	10.78859
F	2.855387	2.304719	9.642742
C	4.957877	2.476185	13.23398
C	2.875931	2.502222	11.99168
C	3.577472	2.563355	13.20375
F	2.893572	2.709903	14.33737
C	5.742274	1.665132	8.475326
H	4.776662	1.986566	8.098153
H	5.829342	0.578852	8.502597
H	6.541683	2.088489	7.872738
C	8.0879	1.869927	12.52771
H	8.155268	0.78193	12.52134
H	7.91844	2.261122	13.52583
H	8.989805	2.302143	12.1021
F	6.944466	-0.05624	10.57634

Table S64. Optimised coordinates (B3LYP/def2-TZVP) for $[\mathbf{6}]^+$ (triplet).

Atom	X	Y	Z
F	0.69252	2.714859	-0.00377
F	3.012572	-1.38155	-0.00068
N	-1.86552	1.075318	0.009778
N	-1.86552	-1.07533	0.009151
N	-2.71469	0.000008	0.007701
C	-0.56872	-0.70372	0.006303
C	-0.56871	0.703671	0.006529
C	0.648148	-1.40499	0.001614
F	0.692632	-2.71489	-0.00397

C	0.648091	1.404966	0.00176
C	1.902152	-0.67799	-0.0001
C	1.902141	0.677977	-0.00001
F	3.012532	1.381571	-0.00051
C	-2.46838	-2.40263	-0.00959
H	-1.69281	-3.15202	0.105608
H	-2.99063	-2.54297	-0.956
H	-3.18262	-2.46909	0.809575
C	-2.46818	2.402695	-0.01016
H	-1.69397	3.151622	0.116756
H	-3.19114	2.46533	0.801551
H	-2.98025	2.547361	-0.96153

Table S65. Optimised coordinates (B3LYP/def2-TZVP) for [**6**(IMe)]$^+$.

Atom	X	Y	Z
C	2.84106	1.894873	-1.69559
N	3.270265	0.873732	-0.74064
C	4.569316	0.598123	-0.37182
C	4.534421	-0.4569	0.473151
N	3.207801	-0.8069	0.653557
C	2.765051	-1.81557	1.623629
C	2.446612	0.014431	-0.1021
H	2.048487	1.483673	-2.31697
H	3.693942	2.166331	-2.31124
H	2.462831	2.771579	-1.17297
H	5.400928	1.164937	-0.75017
H	5.329578	-0.9892	0.963639
H	3.421453	-2.67998	1.550446
H	1.74168	-2.10151	1.405367
H	2.822449	-1.39973	2.628943
F	-2.11567	2.757269	0.281264
F	-4.55604	-1.24875	-0.14762
N	0.362427	1.167898	0.197587
N	0.318142	-1.2103	-0.13466
N	1.095955	0.030169	-0.31996
C	-1.00078	-0.68284	-0.09307
C	-0.97634	0.701344	0.044609
C	-2.20716	-1.35362	-0.14528
F	-2.2674	-2.68379	-0.26251
C	-2.14164	1.434319	0.1231
C	-3.39135	-0.61928	-0.07581
C	-3.36249	0.764223	0.059008
F	-4.49839	1.44596	0.127378
C	0.587948	-2.12054	-1.26181
H	0.377889	-1.64669	-2.22473
H	-0.0381	-2.99987	-1.13666
H	1.628867	-2.43941	-1.22925

C	0.673791	1.486082	1.612154
H	0.483722	0.646387	2.28824
H	0.040988	2.325243	1.89376
H	1.71012	1.81195	1.692766

Table S66. Optimised coordinates (B3LYP/def2-TZVP) for [**6**(PMe_3)]$^+$.

Atom	X	Y	Z
P	-2.64175	-0.00017	-0.66915
C	-3.65935	1.469766	-0.53322
H	-4.20009	1.460336	0.41291
H	-3.03306	2.358955	-0.58914
H	-4.37675	1.483274	-1.35525
C	-3.65886	-1.47052	-0.53346
H	-3.03233	-2.35954	-0.58932
H	-4.19973	-1.46123	0.41259
H	-4.37609	-1.48422	-1.35564
C	-1.78723	0.000238	-2.25338
H	-1.16631	0.891331	-2.34017
H	-1.16561	-0.89034	-2.3403
H	-2.52359	0.00006	-3.05929
F	1.653953	-2.73609	-0.00082
F	3.909068	1.352977	-0.63117
N	-0.78353	-1.19471	0.694695
N	-0.78348	1.194877	0.694236
N	-1.61044	-0.00014	0.680303
C	0.497167	0.695137	0.315307
C	0.497184	-0.6951	0.315334
C	1.642169	1.402021	0.001661
F	1.654098	2.736045	-0.0009
C	1.642102	-1.40207	0.001651
C	2.802502	0.696335	-0.31164
C	2.802482	-0.69646	-0.31166
F	3.909019	-1.35314	-0.63118
C	-0.82245	1.911293	1.984567
H	-1.84434	2.22347	2.1855
H	-0.20023	2.798934	1.884567
H	-0.45452	1.299967	2.812405
C	-0.82192	-1.91031	1.985577
H	-0.45396	-1.29822	2.812858
H	-0.19942	-2.79779	1.886022
H	-1.84366	-2.22262	2.187017

Table S67. Optimised coordinates (B3LYP/def2-TZVP) for [**6**][H] (HIA).

Atom	X	Y	Z
F	-0.68206	-2.73944	-0.09494
F	-3.00914	1.355734	0.150128

N	1.843068	-1.1779	-0.39032
N	1.842714	1.178741	-0.39002
N	2.627351	0.000694	-0.71152
C	0.521085	0.698342	-0.25957
C	0.521414	-0.69804	-0.25951
C	-0.6606	1.398289	-0.11268
F	-0.68308	2.739311	-0.09537
C	-0.66003	-1.3984	-0.11241
C	-1.85395	0.694265	0.031241
C	-1.85365	-0.69481	0.031387
F	-3.00855	-1.35674	0.150381
C	2.37066	1.926716	0.755777
H	2.364598	1.341484	1.682833
H	1.760875	2.819101	0.891592
H	3.390242	2.229648	0.529451
C	2.371746	-1.92656	0.754599
H	1.761998	-2.81898	0.890322
H	2.366408	-1.3419	1.682028
H	3.391145	-2.22948	0.527423
H	2.678122	0.000752	-1.72554

Table S68. Optimised coordinates (B3LYP/def2-TZVP) for [**A**]$^+$.

Atom	X	Y	Z
H	1.408891	-2.52097	-0.00017
H	3.519132	1.226685	-9E-06
N	-1.10989	-1.05191	-0.00019
N	-1.11038	1.051636	0.000119
N	-1.88867	-0.00044	0.000028
C	0.217607	0.700562	0.000189
C	0.217971	-0.70015	0.000003
C	1.4028	1.44082	0.000182
H	1.408749	2.52149	0.000268
C	1.403258	-1.4403	-0.00013
C	2.570172	0.707605	0.000031
C	2.57036	-0.70681	-0.00011
H	3.519478	-1.22544	-0.00022
C	-1.69599	2.39102	-0.0002
H	-1.37591	2.92407	-0.894
H	-1.37213	2.925967	0.891088
H	-2.77586	2.280506	0.002219
C	-1.69463	-2.39179	0.000135
H	-1.37527	-2.92404	0.894654
H	-1.3692	-2.92693	-0.89044
H	-2.77457	-2.28204	-0.00372

Table S69. Optimised coordinates (B3LYP/def2-TZVP) for [**A**]$^+$ (triplet).

Atom	X	Y	Z
H	1.402297	-2.51296	0.000039
H	3.560828	1.227847	-2E-06
N	-1.10971	-1.06913	-0.00023
N	-1.11162	1.067733	0.000139
N	-1.96096	-0.00112	-4.1E-05
C	0.187145	0.702838	0.000037
C	0.188069	-0.70233	-7.2E-05
C	1.387279	1.43442	0.000039
H	1.39872	2.514913	0.000074
C	1.389721	-1.43246	-1.2E-05
C	2.630884	0.677386	0.000007
C	2.631919	-0.6747	-7E-06
H	3.562781	-1.22372	-2.1E-05
C	-1.68262	2.406043	-3.2E-05
H	-1.36606	2.943319	-0.89409
H	-1.3609	2.945618	0.890763
H	-2.76315	2.301999	0.0032
C	-1.67909	-2.40814	0.000119
H	-1.36326	-2.94437	0.895069
H	-1.35535	-2.948	-0.88976
H	-2.75973	-2.30539	-0.00484

Table S70. Optimised coordinates (B3LYP/def2-TZVP) for [**A**(IMe)]$^+$.

Atom	X	Y	Z
C	2.012119	2.125678	-1.39103
N	2.445516	0.974846	-0.60182
C	3.74827	0.636878	-0.29925
C	3.717103	-0.53768	0.367701
N	2.389094	-0.90601	0.508618
C	1.94984	-2.04022	1.328243
C	1.622458	0.028347	-0.09715
H	1.223962	1.809043	-2.07116
H	2.864885	2.493944	-1.95447
H	1.623647	2.910163	-0.7442
H	4.578627	1.252176	-0.59538
H	4.514393	-1.14403	0.758315
H	2.592788	-2.89235	1.117747
H	0.918104	-2.27772	1.090763
H	2.028793	-1.7776	2.38277
H	-2.91657	2.520703	0.540991
H	-5.17054	-1.03982	-0.29275
N	-0.44893	1.154085	0.360494
N	-0.51827	-1.15781	-0.26826
N	0.273805	0.079508	-0.29176
C	-1.8392	-0.6288	-0.15848
C	-1.80242	0.724162	0.154618

C	-3.04403	-1.28924	-0.3211
H	-3.08802	-2.34281	-0.56077
C	-2.95664	1.465474	0.306048
C	-4.21572	-0.54797	-0.16456
C	-4.17601	0.808067	0.141927
H	-5.09849	1.36178	0.250498
C	-0.26651	-1.9209	-1.4981
H	-0.46683	-1.3268	-2.39469
H	-0.91312	-2.79538	-1.48652
H	0.764472	-2.27325	-1.51341
C	-0.12863	1.266451	1.80296
H	-0.75816	2.054826	2.209463
H	0.909725	1.573662	1.92749
H	-0.32002	0.33947	2.353551

Table S71. Optimised coordinates (B3LYP/def2-TZVP) for [**A**(PMe_3)]$^+$.

Atom	X	Y	Z
P	-1.97432	0.000133	-0.50333
C	-2.97522	-1.46777	-0.25422
H	-2.36173	-2.35773	-0.38473
H	-3.40075	-1.46014	0.748937
H	-3.78379	-1.47699	-0.98654
C	-1.31738	0.001159	-2.1797
H	-0.70885	0.89082	-2.33757
H	-0.70756	-0.88752	-2.33817
H	-2.14457	0.000792	-2.89189
C	-2.97573	1.46742	-0.25263
H	-3.40163	1.458301	0.750353
H	-2.36251	2.357762	-0.38176
H	-3.78403	1.47725	-0.98524
H	2.353417	2.495935	-0.37996
H	4.320759	-1.23132	-1.20763
N	0.029209	1.194882	0.609119
N	0.029311	-1.19508	0.608643
N	-0.79346	-0.00025	0.709932
C	1.25967	-0.6949	0.071581
C	1.259649	0.694893	0.071794
C	2.351885	-1.41374	-0.38143
H	2.353665	-2.49569	-0.38066
C	2.351756	1.413983	-0.38103
C	3.455451	-0.69596	-0.84095
C	3.45539	0.696437	-0.84078
H	4.32064	1.231973	-1.20732
C	0.149622	-1.90731	1.893355
H	0.762047	-2.79157	1.725092
H	-0.83688	-2.22814	2.220706
H	0.613448	-1.29377	2.670441

C	0.149687	1.906122	1.894392
H	-0.83667	2.227481	2.221677
H	0.762835	2.790022	1.726876
H	0.612844	1.291669	2.671161

Table S72. Optimised coordinates (B3LYP/def2-TZVP) for [**A**][H] (HIA).

Atom	X	Y	Z
H	1.403772	-2.4967	-0.01961
H	3.527673	1.23097	0.188213
N	-1.10653	-1.11818	-0.35153
N	-1.10655	1.118187	-0.35154
N	-1.93505	-3.7E-05	0.040652
C	0.219309	0.701369	-0.13649
C	0.219307	-0.70135	-0.13651
C	1.396737	1.414717	-0.02058
H	1.403736	2.496689	-0.0193
C	1.396767	-1.41475	-0.02072
C	2.593026	0.693752	0.091837
C	2.592869	-0.69372	0.091716
H	3.527469	-1.23088	0.188022
C	-1.5914	2.368013	0.17954
H	-0.96931	3.178933	-0.19578
H	-1.57525	2.394295	1.281531
H	-2.61239	2.526813	-0.16443
C	-1.59134	-2.36801	0.179649
H	-1.57529	-2.39409	1.281643
H	-0.96913	-3.17892	-0.19547
H	-2.61228	-2.52695	-0.1644
H	-1.92379	-8.7E-05	1.085841

Table S73. Optimised coordinates (B3LYP/def2-TZVP) for [**A**][F] (FIA).

Atom	X	Y	Z
H	1.62715	-2.5093	0.015016
H	3.710466	1.228113	0.413557
N	-0.84114	-1.05658	-0.4971
N	-0.84108	1.056686	-0.4972
N	-1.62351	0.00013	-0.53482
C	0.46276	0.698759	-0.21954
C	0.462635	-0.6988	-0.21952
C	1.624442	1.428396	0.004312
H	1.627468	2.5092	0.01473
C	1.624277	-1.42847	0.004506
C	2.783851	0.701396	0.228047
C	2.783779	-0.7016	0.228136
H	3.710339	-1.22835	0.413794
C	-1.48548	2.334288	-0.24791

H	-0.75962	3.131067	-0.38956
H	-1.8655	2.304586	0.774177
H	-2.30768	2.456491	-0.9488
C	-1.48567	-2.33413	-0.2482
H	-1.86523	-2.30501	0.774077
H	-0.76008	-3.13103	-0.39067
H	-2.30824	-2.45572	-0.94873
F	-2.03251	-8.1E-05	1.533038

Table S74. Optimised coordinates (B3LYP/def2-TZVP) for PMe_3.

Atom	X	Y	Z
P	0	0	0
C	0	1.830346	0
H	-1.05139	2.124815	0
H	0.462492	2.260157	0.88969
H	0.462492	2.260157	-0.88969
C	-1.58513	-0.91517	0
H	-1.31445	-1.97294	0
H	-2.1886	-0.72955	0.88969
H	-2.1886	-0.72955	-0.88969
C	1.585126	-0.91517	0
H	2.36584	-0.15187	0
H	1.726107	-1.53061	0.88969
H	1.726107	-1.53061	-0.88969

Table S75. Optimised coordinates (B3LYP/def2-TZVP) for IMe.

Atom	X	Y	Z
C	2.439418	-0.57127	-5E-06
N	1.06085	-0.12132	-1.5E-05
C	0.675792	1.210623	0.000032
C	-0.67579	1.210623	0.000028
N	-1.06085	-0.12132	-1.9E-05
C	-2.43942	-0.57127	-3E-06
C	0	-0.97644	0.000011
H	2.431251	-1.65765	0.000964
H	2.964433	-0.21522	-0.8888
H	2.964875	-0.21362	0.88788
H	1.37505	2.028942	-0.00011
H	-1.37505	2.028942	-0.00012
H	-2.96445	-0.21518	-0.88878
H	-2.43125	-1.65765	0.000924
H	-2.96486	-0.21365	0.887904

Table S76. Optimised coordinates (B3LYP/def2-TZVP) for $[Me_3Si]^+$.

Atom	X	Y	Z

Si	0	0	0
C	0	1.829394	0
H	-1.00241	2.255862	0
H	0.548727	2.196251	0.875332
H	0.548727	2.196251	-0.87533
C	-1.5843	-0.9147	0
H	-1.45243	-1.99604	0
H	-2.17637	-0.62291	0.875332
H	-2.17637	-0.62291	-0.87533
C	1.584301	-0.9147	0
H	2.454839	-0.25982	0
H	1.627645	-1.57334	0.875332
H	1.627645	-1.57334	-0.87533

Table S77. Optimised coordinates (B3LYP/def2-TZVP) for Me_3SiH.

Atom	X	Y	Z
Si	-0.00012	-0.00016	0.373373
C	0.374625	1.745357	-0.22097
H	1.349197	2.08401	0.13809
H	0.386158	1.797462	-1.31275
H	-0.37518	2.454294	0.137628
C	1.324541	-1.19692	-0.22106
H	1.13136	-2.21034	0.138163
H	1.363811	-1.233	-1.31283
H	2.31321	-0.90123	0.137408
C	-1.69906	-0.54827	-0.22111
H	-2.47954	0.126431	0.138085
H	-1.7498	-0.56412	-1.31288
H	-1.93812	-1.55211	0.137389
H	-2.1E-05	-0.00016	1.863343

Table S78. Optimised coordinates (B3LYP/def2-TZVP) for CF_2O (FIA).

Atom	X	Y	Z
C	0	0	0.08618
O	0	0	1.256803
F	1.064942	0	-0.69149
F	-1.06494	0	-0.69149

Table S79. Optimised coordinates (B3LYP/def2-TZVP) for $[CF_3O]^-$ (FIA).

Atom	X	Y	Z
C	-0.002	0	0.159196
O	0.001106	0	1.372333
F	1.273054	0	-0.50057
F	-0.64248	1.106269	-0.4956
F	-0.64248	-1.10627	-0.4956

Table S80: Optimised coordinates (B3LYP/def2-TZVP) for [**A**][$BArF_{20}$].

Atom	X	Y	Z
F	1.553007	-3.07867	4.361748
F	-2.9012	0.69107	-2.05113
F	-2.87614	-2.13679	-1.54284
F	-1.09667	1.946828	2.009366
F	1.444497	0.694444	1.615935
F	-1.8621	-2.69157	1.183397
F	-6.79084	0.985451	0.538224
F	-2.81952	-0.40024	2.568806
F	-0.44928	4.509293	1.74015
F	0.007689	1.227378	-2.55747
H	6.065838	-2.30504	1.591716
F	-0.68676	-4.05755	3.138088
F	0.552614	-3.91831	-4.16461
F	2.357891	-2.23902	-2.97245
F	2.574954	-0.6742	3.547774
F	1.579362	-0.54785	-1.08379
F	-5.49871	1.248744	-1.85384
F	-2.06557	-3.81799	-3.40031
F	-5.39491	0.152508	2.727299
F	0.441501	5.469232	-0.6585
F	0.66331	3.762547	-2.77885
H	5.848017	-2.76756	-0.80817
C	-0.32778	-0.89215	1.309784
C	-1.57332	-2.09107	-1.85441
C	-0.70603	-1.19952	-1.22415
C	0.952045	-2.38647	3.388796
C	-3.48076	0.52548	-0.84345
C	-0.67134	2.344398	0.796505
C	1.056657	-2.20971	-2.61172
C	-0.18412	-2.87676	2.763306
C	-3.42257	-0.02236	1.424963
C	0.614293	-1.33193	-1.63984
C	-2.71492	0.07129	0.230042
N	3.893397	1.403754	-1.25625
C	-4.82952	0.829571	-0.77196
C	-0.13118	1.971214	-1.43848
C	5.190345	-0.36917	1.515885
H	5.259636	-0.18094	2.577012
C	0.150667	-3.05868	-3.22381
C	5.50175	-1.81587	-0.42849
C	1.456752	-1.17012	2.972897
C	-0.56523	1.414123	-0.23966
C	-0.78034	-2.13714	1.753238
C	-5.48975	0.697394	0.439412
C	0.82464	-0.46745	1.956374
C	-0.3478	3.687521	0.685886

C	4.935985	-0.9116	-1.30041
H	4.809201	-1.13167	-2.34988
N	3.648307	2.294836	-0.33177
C	4.623547	0.55288	0.632694
C	3.908197	2.554811	2.037626
H	3.405595	3.484591	1.790766
H	4.885035	2.757923	2.474129
H	3.295958	1.971272	2.721574
C	0.103485	4.18168	-0.52744
C	4.500579	0.288956	-0.73564
C	-4.77662	0.270878	1.54501
N	4.079342	1.801082	0.799762
C	0.20789	3.310931	-1.59503
B	-1.08444	-0.1527	0.019101
C	3.483376	1.648943	-2.63662
H	2.731828	0.914299	-2.91706
H	4.354234	1.572272	-3.28559
H	3.059893	2.646919	-2.68801
C	-1.17503	-2.99773	-2.83363
C	5.626351	-1.54967	0.954431

Table S81. Optimised coordinates (B3LYP/def2-TZVP) for [**A**•NH_3]$^+$.

Atom	X	Y	Z
N	5.447478	-1.119826	11.047262
H	5.476613	-1.579456	10.141724
H	6.121305	-1.614517	11.624668
H	4.534991	-1.332203	11.439990
N	6.867786	1.892177	10.376432
N	6.692687	2.019571	11.665207
C	5.383670	2.288903	11.975640
C	4.710669	2.505047	13.180885
C	3.358978	2.759587	13.080238
C	2.693177	2.800104	11.833317
C	3.355210	2.587497	10.642294
C	4.724645	2.328951	10.741735
N	5.703422	2.079429	9.813243
C	7.821189	1.843197	12.573412
C	5.572503	1.981536	8.363149
H	7.641860	0.974874	13.205718
H	8.711393	1.689161	11.971335
H	7.935201	2.735818	13.186577
H	6.562130	1.810314	7.950822
H	4.914794	1.149261	8.116729
H	5.161436	2.911390	7.972963
H	5.214562	2.476112	14.136763
H	2.786394	2.935847	13.980984
H	1.631350	3.006305	11.817799

H	2.841386	2.620692	9.691857

Table S82. Optimised coordinates (B3LYP/def2-TZVP) for [**A**•OH_2]$^+$.

Atom	X	Y	Z
O	5.340933	-0.917209	10.957167
H	5.901295	-1.613207	10.594433
H	4.597417	-1.384081	11.355939
N	6.793678	1.732200	10.379917
N	6.629063	1.885422	11.667322
C	5.344945	2.259340	11.973559
C	4.689730	2.536369	13.176421
C	3.361730	2.892895	13.071779
C	2.701280	2.973237	11.823722
C	3.345414	2.700096	10.635364
C	4.691451	2.339164	10.738288
N	5.648244	2.005233	9.813285
C	7.732568	1.605532	12.580129
C	5.502767	1.878684	8.366842
H	7.895779	2.467930	13.224398
H	7.488467	0.728773	13.178507
H	8.618697	1.415246	11.982409
H	6.485907	1.684782	7.948960
H	4.831535	1.049692	8.146139
H	5.102219	2.806562	7.961990
H	5.189486	2.476234	14.132982
H	2.803550	3.120035	13.970192
H	1.658107	3.259095	11.805425
H	2.835808	2.762227	9.684166

Table S83. Optimised coordinates (B3LYP/def2-TZVP) for [**1**•NH_3]$^+$.

Atom	X	Y	Z
N	5.425955	-1.113869	11.064061
H	6.100988	-1.608691	11.639959
H	4.513927	-1.333511	11.454096
H	5.457690	-1.568943	10.156278
N	6.883867	1.888145	10.374085
N	6.709073	2.014432	11.661174
C	5.401436	2.285914	11.972225
C	4.726358	2.501806	13.178575
C	3.377474	2.756855	13.086082
C	2.732551	2.793549	11.830973
C	3.373830	2.585912	10.630945
C	4.741161	2.327913	10.739112
N	5.717169	2.078759	9.810026
C	7.837567	1.834230	12.569611
C	5.584244	1.982944	8.359752

H	5.227934	2.472163	14.135486
H	2.781160	2.936016	13.970542
H	2.837403	2.624742	9.693976
H	7.657065	0.963567	13.198360
H	8.727415	1.680896	11.966873
H	7.951874	2.725046	13.185224
H	4.929873	1.148212	8.112340
H	6.573971	1.816001	7.945883
H	5.169350	2.912134	7.971984
F	1.425499	3.045548	11.823122

Table S84. Optimised coordinates (B3LYP/def2-TZVP) for [**1**•OH_2]$^+$.

Atom	X	Y	Z
O	5.320782	-0.923528	10.945825
H	5.899643	-1.614349	10.602652
H	4.564804	-1.396221	11.313156
N	6.806143	1.725404	10.382041
N	6.642420	1.879471	11.667647
C	5.360406	2.257089	11.974560
C	4.704015	2.537591	13.178094
C	3.379010	2.895420	13.081921
C	2.738585	2.967744	11.825967
C	3.361155	2.697427	10.628657
C	4.704946	2.336461	10.740101
N	5.658224	2.000833	9.814773
C	7.748108	1.604253	12.580831
C	5.509466	1.870425	8.368495
H	5.201864	2.478905	14.135606
H	2.798481	3.129729	13.963979
H	2.828647	2.764954	9.691136
H	7.920104	2.474367	13.212216
H	7.499412	0.737099	13.191047
H	8.629782	1.399054	11.981504
H	6.490636	1.666100	7.950983
H	4.831078	1.046186	8.152210
H	5.117320	2.800506	7.960319
F	1.454651	3.318929	11.814601

Table S85. Optimised coordinates (B3LYP/def2-TZVP) for [**2**•NH_3]$^+$.

Atom	X	Y	Z
N	6.054423	-0.909407	10.869212
H	6.149305	-1.304291	9.937969
H	6.837134	-1.277822	11.401912
H	5.222100	-1.333603	11.268548
N	6.815384	2.365046	10.305990
N	6.664017	2.382015	11.603211

C	5.340993	2.348849	11.959768
C	4.675812	2.349004	13.189409
C	3.300988	2.312433	13.141005
C	2.600356	2.276847	11.908832
C	3.249201	2.277637	10.689525
C	4.642824	2.314155	10.748636
N	5.616135	2.330289	9.782485
C	7.836842	2.399389	12.472154
C	5.455129	2.286698	8.332679
H	5.202414	2.376454	14.132972
H	2.727912	2.311625	14.057684
H	2.701310	2.251301	9.758973
H	7.782005	3.260244	13.136671
H	7.867874	1.478405	13.052490
H	8.716800	2.472206	11.840455
H	6.442600	2.354872	7.886585
H	4.981607	1.347288	8.050780
H	4.841492	3.126799	8.010812
Cl	0.876034	2.232109	11.951633

Table S86. Optimised coordinates (B3LYP/def2-TZVP) for [**2**•OH_2]$^+$.

Atom	X	Y	Z
O	5.963959	-0.745794	10.863730
H	5.393157	-1.410118	11.266730
H	6.646522	-1.251737	10.407673
N	6.760790	2.187351	10.287979
N	6.617935	2.226095	11.585651
C	5.298932	2.304862	11.948448
C	4.642021	2.366712	13.181233
C	3.268677	2.442388	13.139486
C	2.561107	2.455762	11.910708
C	3.200935	2.394485	10.688360
C	4.593493	2.318464	10.740356
N	5.559563	2.246998	9.769725
C	7.789811	2.124957	12.450368
C	5.386451	2.186403	8.321694
H	5.173885	2.355551	14.122148
H	2.702202	2.494204	14.058780
H	2.647705	2.404036	9.760700
H	7.782580	2.946356	13.164634
H	7.765328	1.168837	12.971513
H	8.673005	2.185747	11.821907
H	6.371556	2.229147	7.867200
H	4.892062	1.252151	8.058525
H	4.788359	3.034955	7.993582
Cl	0.839057	2.551921	11.961961

Table S88. Optimised coordinates (B3LYP/def2-TZVP) for [**3**•NH_3]$^+$.

Atom	X	Y	Z
N	5.970570	-0.937760	10.907809
H	6.747601	-1.321074	11.438281
H	6.046663	-1.345724	9.980495
H	5.130395	-1.334297	11.318933
N	6.809978	2.308704	10.300646
N	6.666717	2.344535	11.598554
C	5.345403	2.348693	11.962890
C	4.687861	2.379559	13.195951
C	3.311475	2.376503	13.155682
C	2.603535	2.344022	11.928454
C	3.244357	2.314674	10.705932
C	4.639471	2.317367	10.756248
N	5.607390	2.297819	9.784425
C	7.844449	2.342983	12.460964
C	5.436518	2.241133	8.336225
H	5.220443	2.404850	14.136270
H	2.749005	2.400576	14.078434
H	2.695258	2.291031	9.776063
H	7.815134	3.213524	13.114376
H	7.854618	1.429272	13.053416
H	8.722564	2.384550	11.823916
H	6.422468	2.281558	7.883425
H	4.940006	1.309580	8.068074
H	4.840148	3.091295	8.008415
Br	0.712996	2.342479	11.984875

Table 89. Optimised coordinates (B3LYP/def2-TZVP) for [**3**•OH_2]$^+$.

Atom	X	Y	Z
O	5.883577	-0.773080	10.899643
H	6.550159	-1.301770	10.445756
H	5.297553	-1.417566	11.312815
N	6.750803	2.132415	10.285689
N	6.616500	2.190464	11.583781
C	5.301887	2.305484	11.952797
C	4.653880	2.398202	13.188092
C	3.281741	2.506515	13.152865
C	2.568135	2.521971	11.928388
C	3.198737	2.430352	10.703818
C	4.590009	2.321375	10.748636
N	5.548562	2.214684	9.773481
C	7.790606	2.071610	12.443162
C	5.365537	2.140865	8.327319
H	5.190570	2.385640	14.126297
H	2.727030	2.582779	14.077461
H	2.645283	2.441422	9.776350

H	7.807280	2.901616	13.147239
H	7.746102	1.122880	12.976332
H	8.671364	2.103307	11.809161
H	6.348721	2.153763	7.866859
H	4.846813	1.215985	8.078310
H	4.786593	2.999881	7.992168
Br	0.683958	2.673907	11.991965

Table S90: Optimised coordinates (B3LYP/def2-TZVP) for [**4**•NH_3]$^+$.

Atom	X	Y	Z
N	5.698379	-0.974590	10.585109
H	6.358953	-1.358160	11.255030
H	4.796334	-1.376188	10.824486
H	5.954975	-1.379841	9.689292
N	6.885611	2.249598	10.435191
N	6.714870	2.298452	11.728114
C	5.384566	2.350289	12.050866
C	4.668081	2.410759	13.253365
C	3.300435	2.449741	13.198689
C	2.655050	2.426927	11.944244
C	3.311005	2.367756	10.734872
C	4.702353	2.329571	10.829069
N	5.697022	2.273880	9.887496
C	7.879259	2.296445	12.617169
C	5.559292	2.179053	8.437772
H	2.712144	2.498746	14.105044
H	2.767031	2.351105	9.802258
H	7.881585	3.208589	13.210578
H	7.828684	1.429143	13.272253
H	8.765058	2.248817	11.991453
H	6.554424	2.223780	8.006025
H	5.083444	1.231992	8.187502
H	4.958080	3.012206	8.077378
F	5.321692	2.428952	14.412990
F	1.326583	2.464699	11.951417

Table S91. Optimised coordinates (B3LYP/def2-TZVP) for [**4**•OH_2]$^+$.

Atom	X	Y	Z
O	5.668041	-0.765013	10.680610
H	6.356863	-1.320463	10.297106
H	5.007050	-1.385681	11.009006
N	6.804567	2.097748	10.379022
N	6.650509	2.135458	11.673592
C	5.332259	2.285936	12.012048
C	4.634487	2.378810	13.223714
C	3.273708	2.527067	13.184605

C	2.615832	2.581048	11.937690
C	3.253122	2.494562	10.719877
C	4.638147	2.345046	10.797898
N	5.616159	2.227826	9.844036
C	7.813419	1.987908	12.551753
C	5.457208	2.160025	8.394167
H	2.700683	2.604123	14.098791
H	2.700706	2.539119	9.793170
H	7.871562	2.845224	13.218802
H	7.708146	1.071297	13.128855
H	8.694040	1.938904	11.918867
H	6.448298	2.165320	7.950868
H	4.935615	1.239066	8.136759
H	4.891768	3.025402	8.052801
F	5.297678	2.322538	14.376072
F	1.294868	2.724107	11.961004

Table S92. Optimised coordinates (B3LYP/def2-TZVP) for [**5**•NH_3]$^+$.

Atom	X	Y	Z
N	6.176035	-0.989797	10.527867
H	6.291931	-1.353082	9.586107
H	6.956242	-1.363624	11.060626
H	5.345420	-1.441761	10.899583
N	6.984746	2.300007	10.068682
N	6.813146	2.281644	11.365322
C	5.485650	2.243002	11.699437
C	4.814566	2.209810	12.925308
C	3.444768	2.177649	12.844646
C	2.755050	2.178857	11.600820
C	3.412120	2.212649	10.396274
C	4.807435	2.244537	10.476363
N	5.794027	2.283806	9.527367
C	7.972019	2.273408	12.253490
C	5.654116	2.281610	8.073997
H	5.309360	2.208235	13.885981
H	2.859380	2.213291	9.467710
H	7.913476	3.122149	12.933258
H	7.987332	1.341129	12.816218
H	8.862213	2.351585	11.636961
H	6.648863	2.354198	7.645196
H	5.178132	1.353838	7.759808
H	5.052201	3.135551	7.766765
F	2.711277	2.144774	13.946262
F	1.432116	2.146101	11.639170

Table S93. Optimised coordinates (B3LYP/def2-TZVP) for [**5**•OH_2]$^+$.

Atom	X	Y	Z
O	6.024470	-0.814870	10.420837
H	6.734129	-1.337472	10.029098
H	5.392985	-1.465699	10.748949
N	6.944251	2.125320	10.063478
N	6.777814	2.136190	11.360976
C	5.452789	2.205541	11.700093
C	4.786011	2.237673	12.928526
C	3.417420	2.309359	12.852704
C	2.724537	2.348695	11.611255
C	3.377024	2.317498	10.404163
C	4.771237	2.244315	10.478874
N	5.753784	2.195071	9.526287
C	7.934820	2.031242	12.246011
C	5.605371	2.153318	8.074122
H	5.283144	2.207753	13.887525
H	2.821847	2.346840	9.477550
H	7.932019	2.866232	12.944736
H	7.887595	1.086079	12.785170
H	8.827598	2.065340	11.629212
H	6.598156	2.206187	7.637873
H	5.120932	1.219517	7.791828
H	5.009455	3.003934	7.747577
F	2.688298	2.342503	13.957150
F	1.403096	2.416127	11.654268

Table S94. Optimised coordinates (B3LYP/def2-TZVP) for [**6**•NH_3]$^+$.

Atom	X	Y	Z
N	6.421494	-0.891428	10.941922
H	6.541592	-1.275502	10.008724
H	7.228919	-1.210563	11.470167
H	5.623357	-1.378815	11.339653
N	7.168871	2.390019	10.318887
N	7.027610	2.399208	11.617635
C	5.708311	2.318495	11.977189
C	5.044571	2.282311	13.206008
C	3.669765	2.189900	13.174620
C	2.959692	2.132812	11.948534
C	3.613925	2.167254	10.735941
C	5.007508	2.262004	10.767041
N	5.973520	2.313856	9.797073
C	8.209685	2.461497	12.481063
C	5.812601	2.270033	8.341307
H	8.136004	3.331241	13.130647
H	8.263351	1.553554	13.078496
H	9.078775	2.543286	11.835751
H	6.800796	2.366806	7.902494

H	5.362642	1.319931	8.060205
H	5.174715	3.092690	8.024870
F	5.707707	2.331880	14.354694
F	2.979811	2.151745	14.297576
F	1.644877	2.044025	11.992219
F	2.947113	2.110605	9.589720

Table S95. Optimised coordinates (B3LYP/def2-TZVP) for [**6**•OH_2]$^+$.

Atom	X	Y	Z
O	6.251359	-0.729618	10.833308
H	6.955797	-1.240151	10.416706
H	5.656264	-1.392690	11.202793
N	7.134969	2.216211	10.322320
N	6.997842	2.257367	11.620959
C	5.676520	2.281512	11.982444
C	5.013983	2.316282	13.212445
C	3.636090	2.325465	13.183012
C	2.921523	2.300539	11.958234
C	3.574000	2.265959	10.744712
C	4.971183	2.256929	10.773452
N	5.936669	2.220748	9.802159
C	8.182231	2.227101	12.483642
C	5.769384	2.132165	8.349047
H	8.158500	3.081443	13.156511
H	8.182390	1.299741	13.053404
H	9.054212	2.276310	11.838882
H	6.756618	2.206148	7.903560
H	5.312170	1.175448	8.103161
H	5.137239	2.949642	8.009804
F	5.680859	2.337782	14.359459
F	2.946818	2.356459	14.306512
F	1.603918	2.310035	12.004580
F	2.902492	2.241083	9.600561

6. References

(1) Maier, T. M.; Coburger, P.; van Leest, N. P.; Hey-Hawkins, E.; Wolf, R. Direct Synthesis of an Anionic 13-Vertex Closo-Cobaltacarborane Cluster. *Dalton Trans.* **2019**, *48* (42), 15772–15777. DOI: 10.1039/C9DT03111A.

(2) Zhang, Y.; Santos, A. M.; Herdtweck, E.; Mink, J.; Kühn, F. E. Organonitrile Ligated Silver Complexes with Perfluorinated Weakly Coordinating Anions and Their Catalytic Application for Coupling Reactions. *New J. Chem.* **2005**, *29* (2), 366–370. DOI: 10.1039/B414060E.

(3) Gaussian 16 C.01. Frisch, M. J.; Trucks, G. W.; Schlegel, H. B.; Scuseria, G. E.; Robb, M. A.; Cheeseman, J. R.; Scalmani, G.; Barone, V.; Petersson, G. A.; Nakatsuji, H.; *et al.* Gaussian 16, Revision C.01; Gaussian, Inc.: Wallingford, CT, 2016.
(4) Perdew, J. P.; Ernzerhof, M.; Burke, K. Rationale for Mixing Exact Exchange with Density Functional Approximations. *J. Chem. Phys.* **1996**, *105* (22), 9982–9985. DOI: 10.1063/1.472933.
(5) Krishnan, R.; Binkley, J. S.; Seeger, R.; Pople, J. A. Self-consistent Molecular Orbital Methods. XX. A Basis Set for Correlated Wave Functions. *J. Chem. Phys.* **1980**, *72* (1), 650–654. DOI: 10.1063/1.438955.
(6) Perdew, J. P.; Burke, K.; Ernzerhof, M. Generalized Gradient Approximation Made Simple. *Phys. Rev. Lett.* **1996**, *77* (18), 3865–3868. DOI: 10.1103/PhysRevLett.77.3865.
(7) F. Neese, Software Update: The ORCA Program System—Version 6.0. *Wiley Interdiscip. Rev.: Comput. Mol. Sci.*, 2025, **15**(*2*), e70019.
(8) R. C. Sabando, C. Riplinger, F. Wennmohs, F. Neese and G. Bistoni, Broadening the Scope of the ETS-NOCV Scheme: A Versatile Implementation in ORCA. *J. Chem. Theory Comput.*, 2025, **21**(*16*), 7920–7934.
(9) Danelzik, T.; Joseph, S.; Mück-Lichtenfeld, C.; Daniliuc, C. G.; García Mancheño, O. Benzotriazolium Salts: Emergent Readily Accessible Bench-Stable Lewis Acid Catalysts. *Org. Lett.* **2022**, *24* (33), 6105–6110. DOI: 10.1021/acs.orglett.2c01697.
(10) Christe, K. O.; Dixon, D. A.; McLemore, D.; Wilson, W. W.; Sheehy, J. A.; Boatz, J. A. On a Quantitative Scale for Lewis Acidity and Recent Progress in Polynitrogen Chemistry. *J. Fluor. Chem.* **2000**, *101* (2), 151–153. DOI: 10.1016/S0022-1139(99)00151-7.
(11) Erdmann, P.; Greb, L. Multidimensional Lewis Acidity: A Consistent Data Set of Chloride, Hydride, Methide, Water and Ammonia Affinities for 183 p-Block Element Lewis Acids. *ChemPhysChem* **2021**, *22* (10), 935–943. DOI: 10.1002/cphc.202100150.
(12) Jupp, A. R.; Johnstone, T. C.; Stephan, D. W. Improving the Global Electrophilicity Index (GEI) as a Measure of Lewis Acidity. *Inorg. Chem.* **2018**, *57* (23), 14764–14771. DOI: 10.1021/acs.inorgchem.8b02517.
